# Multi‐Omics Reveals STAT1 as a Central Regulator of Oxidative Stress‐Induced Senescence in the Aging Retina

**DOI:** 10.1111/acel.70792

**Published:** 2026-11-07

**Authors:** Yiran Fan, Ying Li, Xueying Wu, Lin Cai, Ruotong Yang, Zhenhui Liu, Yan Li, Bingtao Hao, Bo Lei, Xiuxiu Jin

**Affiliations:** ^1^ Henan Eye Hospital, Henan Provincial People's Hospital Henan University People's Hospital Zhengzhou Henan China; ^2^ Eye Institute, Henan Academy of Innovations in Medical Science Zhengzhou Henan China; ^3^ Department of Immunology, School of Basic Medical Sciences Zhengzhou University Zhengzhou Henan China

**Keywords:** cellular senescence, horizontal cells, multi‐omics integration, retinal aging, STAT1

## Abstract

Aging is the dominant risk factor for retinal neurodegeneration, yet the upstream regulators that coordinate senescence‐associated programs across retinal cell types remain incompletely defined. Here we combined bulk RNA‐seq and quantitative proteomics of retinas from naturally aged (24‐month) and young (4‐month) mice to define age‐associated molecular programs and infer upstream regulators. We identified coordinated but imperfect transcript‐protein coupling, with prominent immune and metabolic activation and concomitant repression of visual and structural pathways. Network‐based integration nominated Stat1 as a convergent hub linking oxidative stress, inflammatory signaling, and cell fate regulation. Functionally, STAT1 induction accompanied oxidative stress‐driven senescence in human ARPE‐19 cells; ectopic STAT1 expression was sufficient to elicit senescence‐like transcriptional changes, increase reactive oxygen species, and activate inflammasome‐associated signaling, whereas STAT1 knockdown attenuated TBHP‐induced senescence, reduced p53 and ASC upregulation, and blocked stress‐induced nuclear accumulation of STAT1. Re‐analysis of aging retina single‐cell RNA‐seq data further localized Stat1 expression primarily to horizontal cells and glial populations, while Stat1 target programs were engaged across multiple retinal cell types, consistent with compartmentalized initiation and multicellular propagation of inflammatory aging cues. Together, these data establish STAT1 as a mechanistic driver and cell‐context‐dependent marker of retinal aging, providing a rationale for targeting STAT1‐centered pathways to delay or mitigate age‐related retinal degeneration.

## Graphical abstract

Integrated multi‐omics identifies STAT1 as a central regulator of retinal aging, linking oxidative stress, inflammation, and senescence at neuro‐glial interfaces.

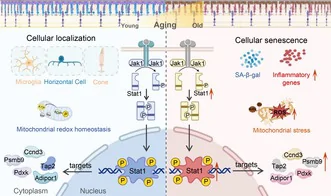

## 1 Introduction

Aging is a well‐recognized risk factor for neurodegenerative diseases, including those affecting the retina (López‐Luppo et al. 2017). The retina, a highly specialized neural tissue with structured layers of photoreceptors, interneurons, and ganglion cells (Masland 2001), is particularly vulnerable to oxidative stress (Barot et al. 2011; Abokyi et al. 2020), mitochondrial dysfunction (Eells 2019), and chronic inflammation with advancing age (Chen and Xu 2015; Xu et al. 2009). These pathological changes contribute to progressive functional decline and increased susceptibility to retinal disorders such as age‐related macular degeneration (AMD), glaucoma, and inherited retinal degenerations (Abokyi et al. 2020; Van Bergen et al. 2011; Ambati and Fowler 2012). Understanding the molecular mechanisms of retinal aging is therefore critical for developing interventions that preserve vision in the aging population (Jarrett and Boulton 2012; Terao et al. 2022).

Retinal aging is characterized by multilayered perturbations in gene expression, protein homeostasis, and intracellular signaling networks (Terluk et al. 2015). Among these, mitochondrial dysfunction is a central feature, leading to impaired oxidative phosphorylation and excessive accumulation of reactive oxygen species (ROS), thereby exacerbating oxidative stress and triggering inflammatory responses (Zhao et al. 2011). Concurrently, defective mitochondrial autophagy (mitophagy) promotes the persistence of damaged mitochondria, driving cellular senescence and inflammasome activation (Sebastián et al. 2017; Palikaras et al. 2018). Additional hallmarks include lipid metabolism disorders, defects in protein quality control, and remodeling of the extracellular matrix, reflecting the multifactorial nature of the aging retina (Wang et al. 2009; Datta et al. 2017). Chronic low‐grade inflammation, referred to as inflammaging, is widely recognized as a key driver of age‐related retinal degeneration (Franceschi et al. 2018; Kauppinen et al. 2016). The interplay among oxidative stress, impaired autophagy, and aberrant cell death pathways often forms a vicious cycle that accelerates retinal functional decline (Campisi 2016; López‐Otín et al. 2013). These changes not only align with the established hallmarks of aging but also underscore the retina's heightened vulnerability to oxidative damage and its substantial metabolic demands (Kushwah et al. 2023). Furthermore, recent evidence highlights the importance of epigenetic remodeling and metabolic reprogramming in retinal aging (Pal and Tyler 2016; Sen et al. 2016), where the bidirectional interaction between metabolic states and epigenetic landscapes profoundly influences transcriptional programs and long‐term cellular function.

Given the multifactorial nature of retinal aging and the complex interactions among oxidative, inflammatory, and structural processes, unraveling the underlying molecular mechanisms remains a significant challenge. Recent advances in high‐throughput omics technologies now enable systematic characterization of age‐associated molecular alterations in ocular tissues. For example, integrated transcriptomic and proteomic analyses of the retinal pigment epithelium (RPE) in patients with geographic atrophy have revealed significant disruptions in mitochondrial metabolism, lipid synthesis, and extracellular matrix remodeling (Senabouth et al. 2022). Quantitative proteomic profiling of intracellular and secreted RPE proteins across a broad donor age spectrum implicated RNF123‐ and RNF149‐mediated ubiquitination as a key protective mechanism against oxidative stress in human RPE during aging (Jin et al. 2022). Similarly, transcriptomic profiling in aged mouse RPE shows upregulation of genes involved in inflammation, immune response, and oxidative stress, coupled with downregulation of genes related to visual perception and extracellular matrix formation (Dubey et al. 2025). Collectively, these studies underscore the utility of multi‐omics approaches yet also expose a knowledge gap: the absence of an integrative molecular map of the naturally aged retina and the identification of its central regulatory hubs.

In this study, we address this gap by constructing an integrated molecular map of the naturally aged mouse retina using matched transcriptomic and proteomic profiling. By coupling multi‐omics integration with regulator‐ and network‐centric inference, and by resolving cell‐type programs using single‐cell transcriptomic context, we identify STAT1 as a central upstream hub of retinal aging. We further demonstrate that STAT1 mechanistically links oxidative stress to senescence and inflammasome‐related activation, positioning STAT1 as a candidate master regulator that coordinates oxidative and inflammatory trajectories across retinal cell types. Together, our work provides a framework for prioritizing upstream regulatory targets in retinal aging and suggests STAT1 as a tractable entry point for therapeutic intervention in age‐associated retinal degeneration.

## 2 Results

### 2.1 Age‐Dependent Activation of SASP‐Related and Oxidative Stress Pathways in the Retina

To comprehensively investigate age‐related molecular changes in the retina, we conducted integrated TMT‐based quantitative proteomics and RNA‐seq on retinal tissues from young (4 months, *n* = 8) and aged (24 months, *n* = 8) mice. We quantified 7019 proteins and detected 39,325 genes, with 6763 molecules identified across both platforms (Figure 1A, Table S1). Principal component analysis (PCA) demonstrated a clear distinction between young and aged retinas, with one aged sample (old‐2) excluded due to outlier behavior (Figure 1B). These data indicate extensive and coordinated remodeling of the retinal proteome and transcriptome with age.

**FIGURE 1:**
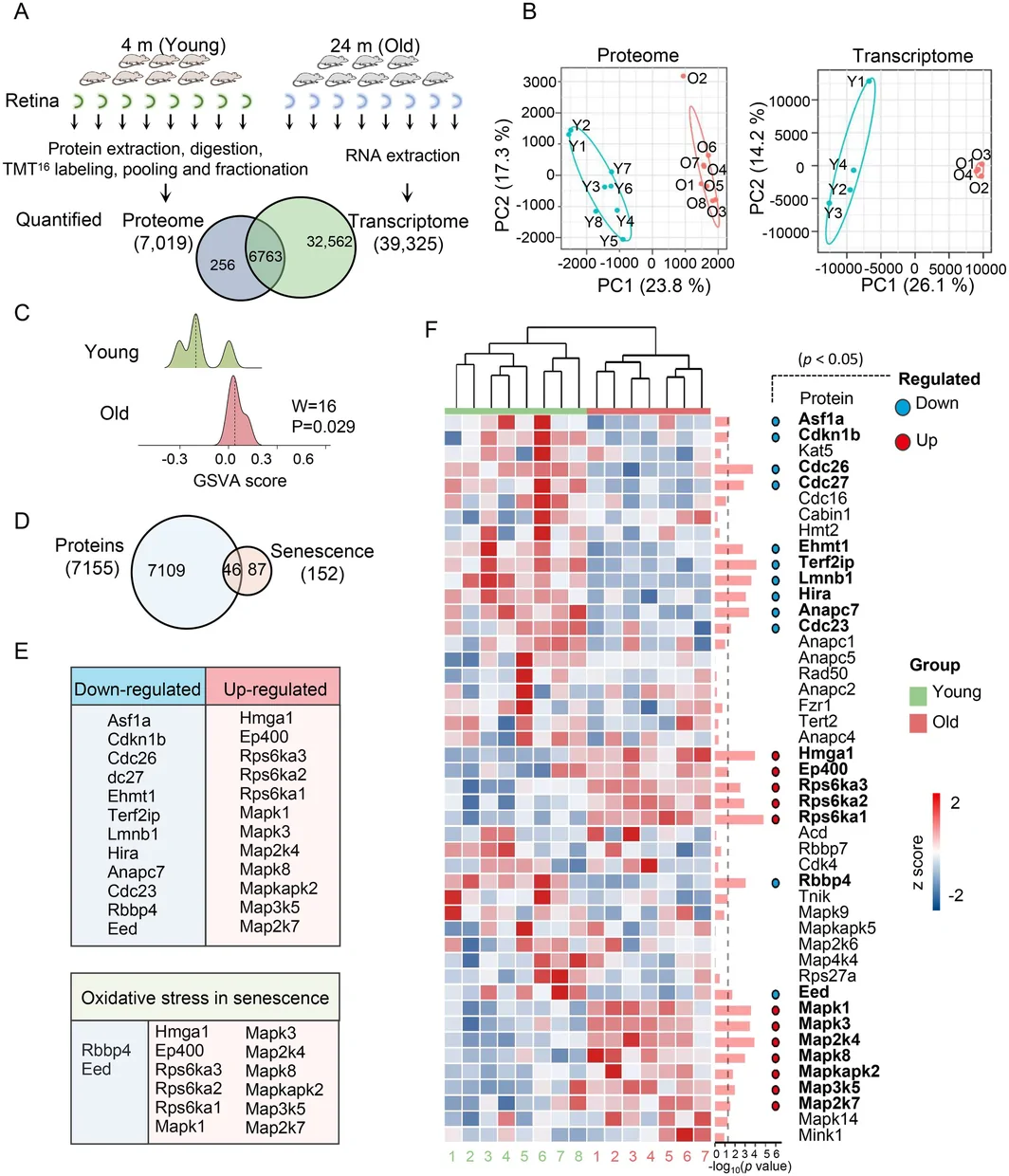
Integrated proteomic and transcriptomic profiling of retinal aging. (A) Schematic of the experimental design comparing retinas from young (4‐month‐old) and aged (24‐month‐old) C57BL/6J mice (*n* = 8 per group) using tandem mass tag (TMT)‐based proteomics and RNA‐seq. Integrated analysis quantified 7019 proteins and 39,325 transcripts, with 6763 targets consistently detected across both omics layers. A total of 7155 proteins were identified, of which 7019 were quantitatively analyzed. (B) Principal component analysis (PCA) revealed clear age‐dependent separation in proteomic (PC1 = 23.8%) and transcriptomic profiles (PC1 = 26.1%). (C) Gene set variation analysis (GSVA) scores of the transcriptome for the senescence‐associated secretory phenotype (SASP) gene set displayed as a ridge plot. Group differences were assessed using a two‐sided exact Wilcoxon rank‐sum test (W = 16, *p* = 0.029). (D) A Venn diagram illustrates the overlap between the proteome (7155 proteins) and the SASP‐related protein set (152 proteins), with 46 proteins overlapping in senescence. (E) A table summarizes proteins down‐regulated, up‐regulated, or associated with oxidative stress during aging. (F) *Z*‐score normalized expression levels of differentially regulated proteins are shown in a heatmap, with rows representing proteins and columns representing samples. Hierarchical clustering of samples is indicated at the top, and proteins significantly down‐ or up‐regulated (*p* < 0.05) are highlighted in blue and red, respectively. The −log_10_(*p*‐value) is shown along the bottom axis.

Senescence‐associated secretory phenotype (SASP) factors are established drivers of age‐related inflammation and tissue degeneration (Li et al. 2023). Transcriptomic scoring of a curated SASP gene set (Liberzon et al. 2015) revealed significantly higher SASP activity in aged retinas compared with young controls (*W* = 16, *p* < 0.05) (Figure 1C). Notably, elevated SASP scores were also detected in a subset of young samples, indicating variation in senescence‐related activity across samples. Targeted interrogation of the proteomic dataset identified 46 SASP‐related proteins (Figure 1D, Table S1), of which 12 were down‐regulated (e.g., ASF1A, EED, CDKN1B), and 12 were up‐regulated, encompassing seven MAPK family kinases (e.g., MAPK1, MAPK3, MAP2K4) (Figure 1E,F). Fourteen differentially expressed SASP proteins were annotated to oxidative stress pathways (Figure 1E), implicating stress‐response signaling in SASP activation during retinal aging.

To evaluate longevity‐associated networks, we cross‐referenced the proteome with 137 murine aging‐related genes curated in GenAge (Tacutu et al. 2018) (Figure S1A). Sixty‐seven corresponding proteins were detected (Figure S1B), with 31 significantly altered in aged retinas: 11 down‐regulated proteins (e.g., ARNTL, EEF1E1, HNRNPD, RAE1, BUB3) that are associated with reduced lifespan and 20 up‐regulated proteins (e.g., GSTA4, MIF, COQ7, CTF1, AKT1) previously associated with extended longevity (Figure S1C). Together, these results indicate progressive activation of SASP‐associated pathways during retinal aging, accompanied by oxidative stress‐related changes and modulation of longevity‐associated proteins, while also revealing substantial heterogeneity in senescence‐associated activity across individuals.

### 2.2 Aging Induces Distinct Yet Correlated Retinal Proteomic and Transcriptomic Expression Changes

Comparative proteomics identified 292 differentially expressed proteins (DEPs; *p* < 0.05, fold change ≥ 1.2) between young and aged mouse retinas, including 99 down‐regulated and 193 up‐regulated proteins (Figure 2A, Table S2). RNA‐seq detected 1002 differentially expressed genes (DEGs; FDR < 0.05, fold change ≥ 2), consisting of 369 up‐regulated and 633 down‐regulated transcripts (Figure 2B, Table S3). To assess concordance between layers, we compared fold changes for significantly changed features and observed a positive correlation between proteomic and transcriptomic effects (Pearson's *r* = 0.603; *p* < 0.001; *n* = 432) (Figure 2C). Twenty‐three DEPs were also differentially expressed at the mRNA level (FDR < 0.05, fold change ≥ 2), including 10 consistently up‐regulated, 10 consistently down‐regulated, and 3 discordant features (Figure 2D). The discordant group, including COL4A1, COL4A2, and NID2, suggests regulation beyond transcript abundance, consistent with post‐transcriptional or post‐translational control.

**FIGURE 2:**
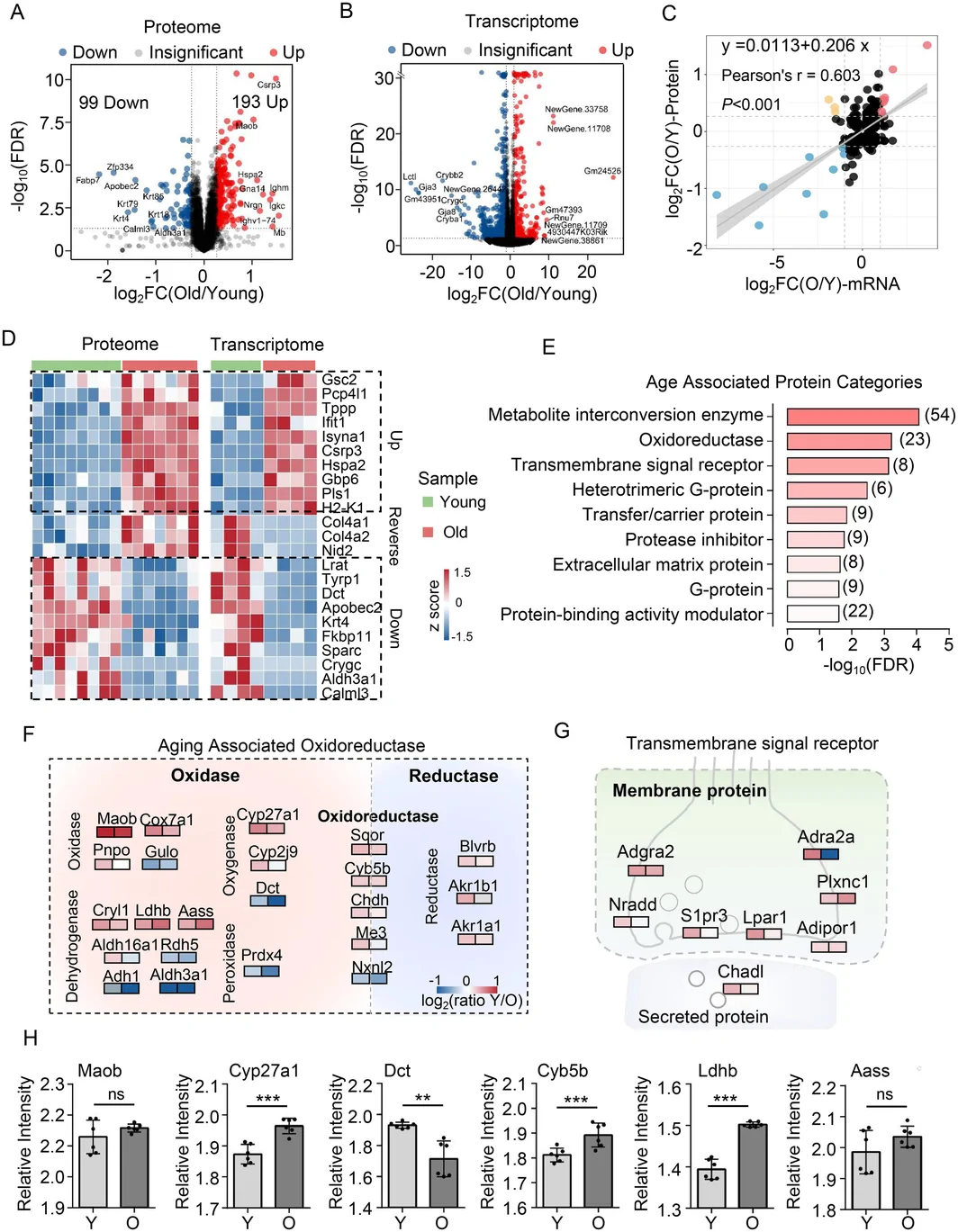
Age‐associated retinal protein and gene expression changes. (A) Volcano plot of differentially expressed proteins (DEPs) between young and aged mouse retinas. A total of 292 DEPs were identified as significantly altered (*p* < 0.05; fold change ≥ 1.2), including 99 down‐regulated and 193 up‐regulated proteins. (B) Volcano plot of transcriptomic changes in aging retinas, revealing widespread gene expression alterations. Differentially expressed genes (DEGs) were defined by FDR < 0.05 and fold change ≥ 2, identifying 369 up‐regulated and 633 down‐regulated transcripts. (C) Correlation analysis of age‐associated changes between proteomic and transcriptomic datasets. Each data point represents a gene–protein pair, revealing a significant positive correlation between protein abundance and corresponding mRNA expression (Pearson's *r* = 0.603; *p* < 0.001; *n* = 432). (D) Heatmap showing proteins with significant consistent or divergent regulation at both the transcript and protein levels. Among these, 10 were concordantly up‐regulated (red‐colored in Figure C), 10 were concordantly down‐regulated (blue‐colored in Figure C), and 3 displayed discordant trends (up‐regulated at the protein level but down‐regulated at the transcript level, yellow‐colored in Figure C). (E) Functional classification of DEPs using the PANTHER system (v17.0). Enriched protein classes included metabolite interconversion enzymes (*n* = 54), oxidoreductases (*n* = 23), protein‐binding activity modulators (*n* = 22), transmembrane signal receptors (*n* = 8), transfer/carrier proteins (*n* = 9), protease inhibitors (*n* = 9), extracellular matrix proteins (*n* = 9), and heterotrimeric G proteins (*n* = 6). (F) Subclassification of oxidoreductases revealed predominant age‐related upregulation (e.g., Maob, Cyp27a1, Cyb5b) versus selective downregulation (e.g., Gulo, Dct, Adh1) proteins, as illustrated by a heatmap of representative targets. (G) Age‐associated transmembrane signal receptors and their expression patterns at both the protein and transcriptomic levels. (H) RT–qPCR validation of targets, including Maob, Cyp27a1, Dct, Cyb5b, Ldhb, and Aass, in six biological replicates. Statistical significance was determined using two‐tailed tests and is indicated as ns, not significant; **p < 0.01; ***p < 0.001.

### 2.3 Aging Predominantly Rewires Retinal Metabolic, Redox, and Receptor‐Mediated Proteins

PANTHER‐based functional classification indicated that DEPs were predominantly metabolite interconversion enzymes (54 DEPs) and oxidoreductases (23 DEPs), with additional groups including transmembrane signal receptors, heterotrimeric G‐proteins, transfer/carrier proteins, protease inhibitors, extracellular matrix proteins, G‐proteins, and protein‐binding activity modulators (Figure 2E). Given the retina's high metabolic demand, alterations in metabolic enzymes and oxidoreductases indicate age‐related remodeling in energy metabolism and possibly perturbed redox homeostasis (Tsantilas et al. 2021). Redox‐related enzymes were further categorized as oxidases (including oxygenases, peroxidases, and dehydrogenases), reductases, and oxidoreductases, showing predominantly up‐regulated expression at both the protein and transcript levels (Figure 2F). Notable up‐regulated examples included CYP27A1 and CYB5B, while GULO, DCT, and ADH1 were down‐regulated. Within the transmembrane signal receptor category, proteins were further classified as membrane proteins (ADGRA2, ADRA2A, NRADD, S1PR3, LPAR1, ADIPOR1, PLXNC1) and a secreted factor (CHADL). All showed consistent upregulation across omics layers except ADRA2A, which exhibited opposing directions (Figure 2G). Validation of representative genes (Maob, Cyp27a1, Dct, Cyb5b, Ldhb, and Aass) in six independent samples by RT‐qPCR confirmed expression trends consistent with multi‐omics analyses (Figure 2H), supporting the robustness of these findings.

### 2.4 Pathway Enrichment Reveals Immune Activation and Metabolic Reprogramming During Retinal Aging

Proteome‐based enrichment analysis (Metascape) identified 12 pathways significantly up‐regulated (*p* < 0.05) in aged retinas, dominated by immune regulation and metabolic processes (Figure 3A), including antigen processing and presentation, negative regulation of extrinsic apoptotic signaling pathway via death domain receptors, monocarboxylic acid metabolic process, and negative regulation of cellular component organization. Six pathways were significantly down‐regulated (Figure 3B), including sarcomere organization, intermediate filament cytoskeleton organization, visual perception, retinol metabolism, tyrosine metabolism, and cellular response to vascular endothelial growth factor stimulus.

**FIGURE 3:**
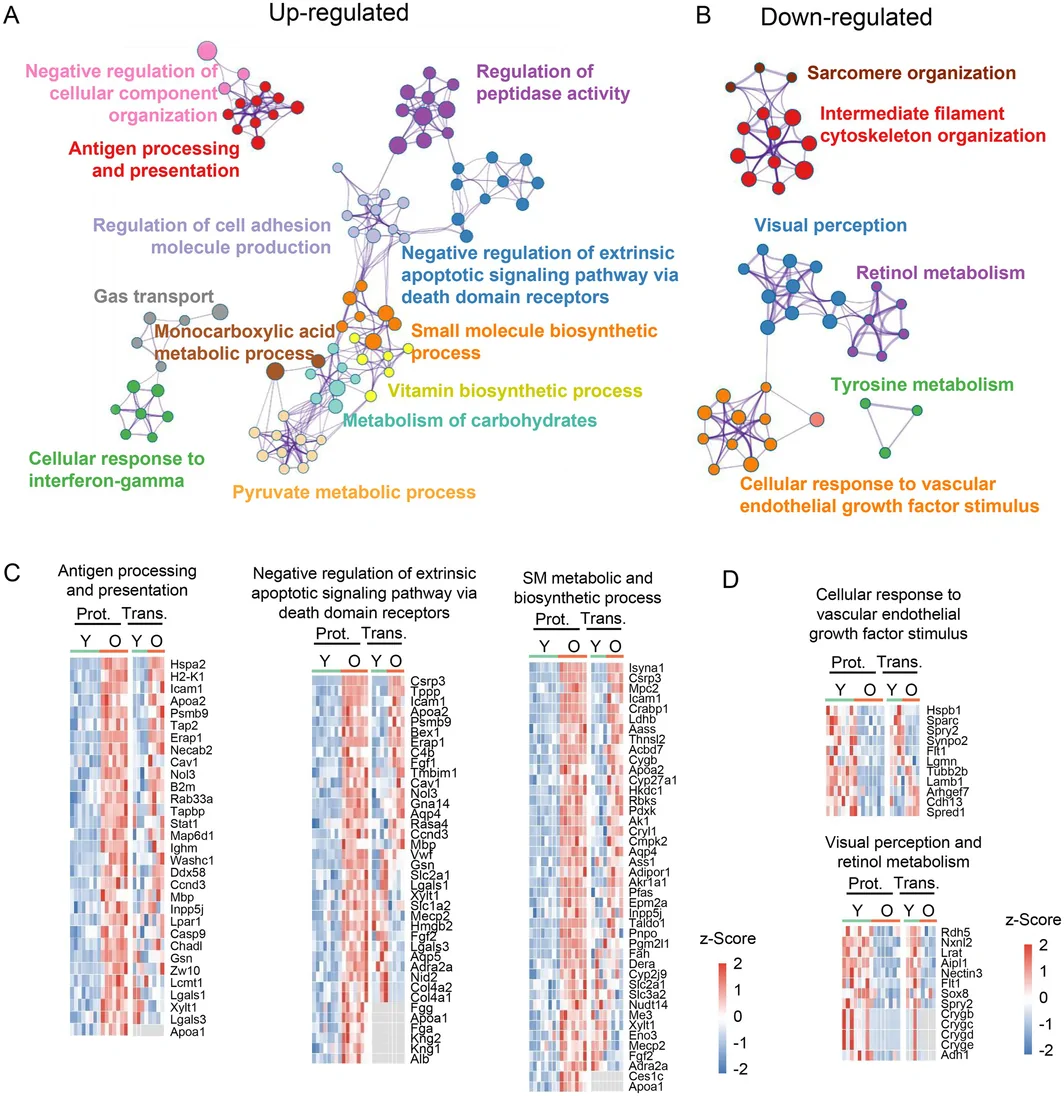
Differential expression of key pathways in retinal aging. (A) Upregulated pathways predominantly reflect immunometabolic adaptation, encompassing antigen processing and presentation, regulation of cell adhesion molecule production, modulation of peptidase activity, suppression of death receptor–mediated apoptosis, and diverse biosynthetic processes, including vitamin, carbohydrate, pyruvate, and monocarboxylic acid metabolism, as well as oxygen transport. (B) Down‐regulated pathways indicate structural and functional decline, including sarcomere and intermediate filament organization, visual perception, tyrosine and retinol metabolism, and cellular responses to vascular endothelial growth factor (VEGF) stimulation. (C) Representative gene and protein profiles of the activation of antigen processing, apoptosis suppression, and small‐molecule biosynthesis in aged tissue. (D) Expression landscapes of the downregulation of VEGF‐responsive signaling, visual perception pathways, and retinal metabolic processes in aged tissue.

Cross‐omics comparison revealed pathway‐specific coupling between transcriptional and proteomic changes. For antigen processing and presentation, 31 proteins were detected and showed generally high cross‐platform consistency; Stat1, Ccnd3, Psmb9, and Tap2 were increased in both datasets, whereas Lcmt1, Lgals1, and Xylt1 were discordant. For negative regulation of the extrinsic apoptotic signaling pathway via death domain receptors, 38 proteins were identified and exhibited lower concordance overall; Apoa2 and Psmb9 were consistently up‐regulated, while Slc2a1 and Lgals1 displayed inverse changes, suggesting post‐transcriptional regulation. For small‐molecular metabolic and biosynthetic processes, 42 proteins were identified, while proteins such as Slc2a1, Slc3a2, and Nudt14 showed mild inconsistency (Figure 3C). Down‐regulated pathways showed higher concordance. In cellular response to vascular endothelial growth factor stimulus, 11 proteins were identified, with discordance limited to Arhgef7, Cdh13, and Spred1. In visual perception and retinol metabolism, 13 proteins were detected, with only Sox8 showing an opposing change direction (Figure 3D). Together, these integrated analyses define coordinated immune activation and metabolic reconfiguration alongside attenuation of sensory and growth‐factor response programs during retinal aging, while highlighting pathway‐dependent divergence between mRNA and protein regulation.

### 2.5 Stat1 Functions as a Central Transcriptional Regulator of Retinal Aging

To explore upstream regulators of retinal aging, we annotated DEPs using AnimalTFDB and HOCOMOCO, which yielded five candidate transcription factors, including Zfp41, Sox8, Stat1, Tfam, and Hmga1 (Figure 4A; Table S4). Target gene sets for each candidate were then compiled from TFlink and the Cistrome Data Browser and intersected with DEGs from the identified aged retina transcriptomic dataset. Among these candidates, Stat1 showed the greatest regulatory coverage and the strongest enrichment of its target genes (Tables S5, S6). Therefore, Stat1 was prioritized as a candidate core upstream regulator of retinal aging (Figure S2). A Stat1‐centered regulatory network was subsequently constructed using overlapping Stat1 target genes that were differentially expressed in the aged retina, including Psmb9, Tap2, and Adipor1 (Figure 4B).

**FIGURE 4:**
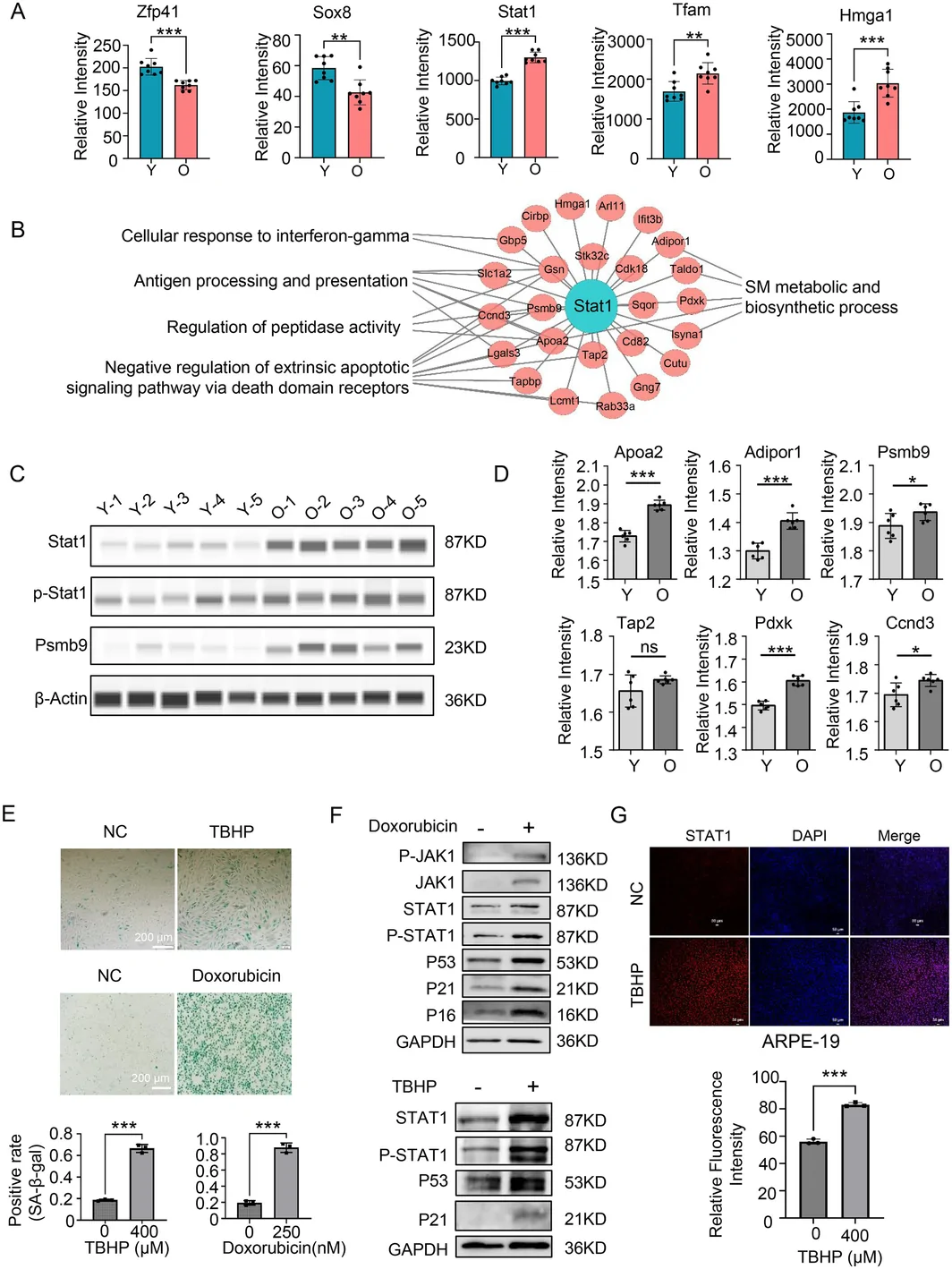
Stat1‐centered regulatory network in retinal aging. (A) Identification of candidate transcription factors associated with retinal aging. Differentially expressed proteins (DEPs) were annotated using AnimalTFDB and HOCOMOCO, yielding 5 candidate transcription factors potentially involved in retinal aging. (B) Functional pathway mapping of Stat1 target genes demonstrated broad regulation of aging‐relevant processes, including antigen processing and presentation, interferon‐γ signaling, regulation of peptidase activity, suppression of death receptor–mediated apoptosis, and small‐molecule biosynthesis and metabolism. (C) Representative Jess Simple Western analysis of Stat1 protein expression in retinal lysates from young (4 months) and naturally aged (24 months) mice. The native PDF reports have been uploaded as Raw Data S1. (D) RT–qPCR validation (*n* = 6) confirms age‐dependent expression changes in Stat1‐responsive genes, including Apoa2, Adipor1, Psmb9, Tap2, Pdxk, and Ccnd3. (E) Senescence‐associated β‐galactosidase (SA‐β‐gal) staining of ARPE‐19 cells following TBHP (400 μM, 2 h) or Doxorubicin (250 nM, 3 day) treatment. Quantification of SA‐β‐gal‐positive cells is shown. Data are presented as the mean ± SEM. (F) Western blot analysis of STAT1, p21, p53, and downstream targets in ARPE‐19 cells following TBHP and Doxorubicin treatment. (G) Immunofluorescence analysis of STAT1 in ARPE‐19 cells 2 h after TBHP treatment. Nuclei were counterstained with DAPI. Scale bar: 50 μm.

Re‐analysis of published ChIP‐seq datasets revealed pronounced Stat1 occupancy at the transcriptional start sites of these target genes (Figure S3) (Ng et al. 2011). Functional enrichment linked the Stat1 network to interferon‐γ responses, antigen processing, and sphingomyelin metabolism (Figure 4B). We next validated these findings in vivo. Jess Simple Western analysis showed a marked accumulation of total Stat1 protein in aged (24 months) mouse retinas compared with young (4 months) controls (Figure 4C). Quantitative analysis further confirmed increased Stat1 protein abundance and elevated Psmb9 protein levels in aged retinas, whereas p‐Stat1 did not show a statistically significant difference between groups (Figure S4). Consistently, age‐stratified RT‐qPCR confirmed increased expression of representative Stat1 targets, including Apoa2, Adipor1, Psmb9, Tap2, Pdxk, and Ccnd3 (Figure 4D). Together, these data support an age‐associated enhancement of the Stat1‐related inflammatory and antigen‐processing program in the retina. To test whether Stat1 is induced in cellular senescence, we established stress‐induced premature senescence in ARPE‐19 cells using oxidative stress (TBHP) or DNA damage (Doxorubicin). Both interventions recapitulated canonical senescence phenotypes, evidenced by robust SA‐β‐gal staining (Figure 4E). In both TBHP‐ and Doxorubicin‐induced senescent ARPE‐19 cells, STAT1 abundance increased, accompanied by activation of the phosphorylated JAK1–STAT1 axis and elevated p21 and p53 levels (Figure 4F, Figure S5). Immunofluorescence further corroborated increased STAT1 signal in senescent cells (Figure 4G).

### 2.6 STAT1 Overexpression Recapitulates Transcriptomic and Phenotypic Hallmarks of Retinal Cell Senescence

To test whether STAT1 activation is sufficient to elicit a senescence‐like program, we performed RNA‐seq in STAT1‐overexpressing (OE) and control (NC) ARPE‐19 cells. We identified 2029 DEGs (fold change ≥ 2, *p* < 0.05), including 1202 down‐regulated and 827 up‐regulated genes. In parallel, RNA‐seq comparing TBHP‐induced senescent and NC cells identified 747 DEGs, including 597 down‐regulated and 150 up‐regulated genes (Figure 5A, Table S7). Comparison of log2 fold changes for significantly altered genes (FDR < 0.05) revealed a significant positive correlation between the STAT1‐OE and senescence models (Pearson's *r* = 0.589, *p* < 0.001; Figure 5B), indicating that STAT1 activation captures a substantial fraction of the senescence‐associated transcriptional response.

**FIGURE 5:**
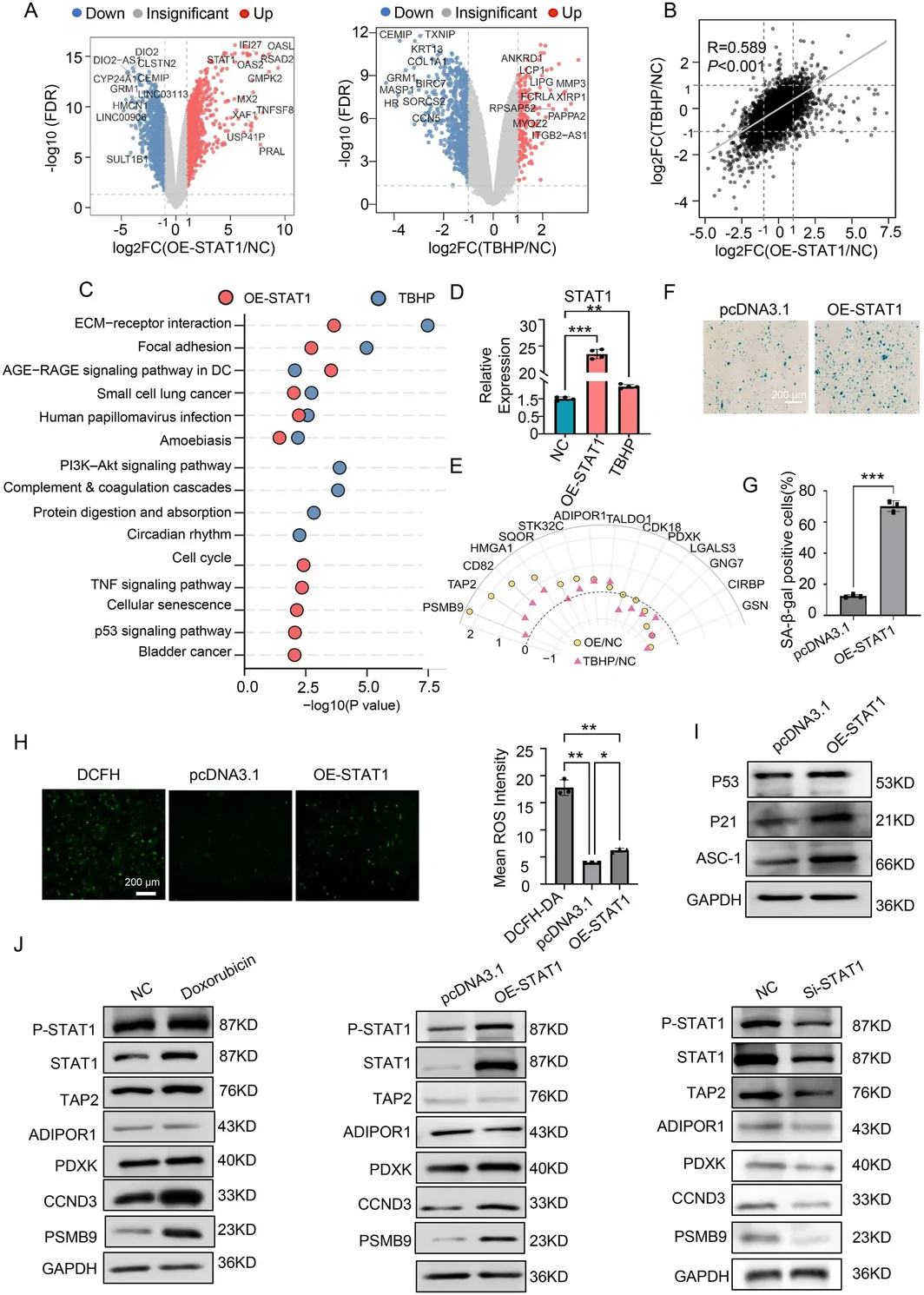
STAT1 regulation of aging‐associated transcriptional programs. (A) Volcano plots of differentially expressed genes (DEGs) from RNA‐seq analysis. Left: STAT1‐overexpression (OE) versus control (NC) cells (2029 DEGs, 1202 down‐regulated, 827 up‐regulated; |log_2_FC| > 1, FDR < 0.05). Right: Stress‐induced senescent cells (TBHP or Doxorubicin) versus NC (747 DEGs; |log_2_FC| > 1, FDR < 0.05). (B) Correlation of log_2_ fold changes for shared genes between STAT1‐OE and stress‐induced senescent cells (Pearson's *r* = 0.589, FDR < 0.05). (C) KEGG pathway enrichment analysis was performed based on DEGs identified in STAT1‐OE ARPE‐19 cells and TBHP‐induced senescent cells. Shared enriched pathways between the two models included ECM–receptor interaction, focal adhesion, AGE–RAGE signaling pathway in diabetic complications, small cell lung cancer (SCLC), human papillomavirus infection (HPV infection), and amoebiasis. Dot plots display significantly enriched pathways ranked by −log_10_ (*p*‐value). Blue dots indicate pathways enriched following TBHP treatment, including the PI3K–Akt signaling pathway, the complement and coagulation cascades, protein digestion and absorption, and the circadian rhythm. Red dots represent pathways enriched in the STAT1 overexpression group, including the Cell cycle, TNF signaling pathway, Cellular senescence, p53 signaling pathway, and Bladder cancer. (D) STAT1 expression in RNA‐seq data from Control, STAT1‐OE, and senescent cells (*n* = 4 per group); one‐way ANOVA was used for statistical analysis. (E) Fourteen core STAT1 downstream targets (e.g., PSMB9, TAP2, CD82, HMGA1, CCND3, GSN, GBP5) identified in aged mouse retina by multi‐omics analysis, showing consistent regulation in STAT1‐OE and TBHP‐induced senescent cells. (F, G) Representative SA‐β‐gal staining images (F) and quantification (G), showing increased senescence in STAT1‐OE cells. Data are presented as mean ± SD and were analyzed by one‐way ANOVA. (H) Intracellular ROS levels in STAT1‐OE and NC cells; data are mean ± SD, analyzed by one‐way ANOVA. (I) Western blots of senescence markers p53, p21, and inflammasome adaptor ASC in STAT1‐OE and NC cells. (J) Western blot validation of STAT1 and target proteins in untreated control (NC), STAT1‐OE, Doxorubicin (Dox)‐treated, and STAT1‐knockdown (si‐STAT1) ARPE‐19 cells.

Pathway enrichment analysis further supported convergence between the two perturbations. Six pathways were commonly enriched, including the ECM–receptor interaction, focal adhesion, AGE–RAGE signaling pathway in diabetic complications, small cell lung cancer, human papillomavirus infection, and amoebiasis, consistent with shared stress‐ and inflammation‐related programs (Figure 5C). Despite this overlap, each model retained distinct signatures. STAT1 overexpression preferentially enriched pathways related to cell‐cycle control and damage responses, including Cell cycle, TNF signaling, Cellular senescence, and p53 signaling, whereas TBHP‐induced senescence selectively enriched PI3K–Akt signaling, the complement and coagulation cascades, protein digestion and absorption, and circadian rhythm (Figure 5C). Gene Ontology (GO) analysis highlighted a clear functional divergence between models (Figure S6): TBHP‐induced senescence was dominated by extracellular matrix remodeling and adhesion terms, whereas STAT1 overexpression was dominated by interferon/antiviral response programs, indicating preferential activation of immune signaling by STAT1.

Based on integrative multi‐omics analyses of the senescent mouse retina, we identified Stat1 as a central regulatory hub and delineated a set of 14 core downstream Stat1 target genes, including Tap2, CD82, Hmga1, Ccnd3, Gsn, and Gbp5, defined by their consistent differential regulation and predicted Stat1 binding. These genes exhibited concordant regulation in both STAT1‐OE cells and TBHP‐induced senescence models (Figure 5D,E), supporting their shared positioning within a STAT1‐centered senescence network. Consistent with these transcriptional changes, ectopic STAT1 expression induced canonical senescence phenotypes: increased SA‐β‐gal positivity (Figure 5F,G, Figure S7), and elevated intracellular reactive oxygen species (Figure 5H, Figure S8). STAT1 overexpression also increased p53 and p21 abundance, together with the inflammasome adaptor ASC, consistent with amplification of senescence‐associated inflammatory signaling. Moreover, expression of representative STAT1 targets (CCND3, ADIPOR1, and PSMB9) tracked with STAT1 activity in both Doxorubicin‐induced senescence and STAT1 knockdown conditions, reinforcing STAT1 as a functional regulator of the senescence‐associated transcriptional network (Figure 5I,J, Figures S9, S10).

### 2.7 STAT1 Knockdown Mitigates Oxidative Stress‐Induced Senescence and Inflammasome Activation

To determine the essential role of STAT1 in oxidative stress‐induced senescence, we depleted STAT1 in ARPE‐19 cells using siRNA and then induced senescence with TBHP (Figure 6A, Figure S11). The results showed that STAT1 depletion significantly attenuated TBHP‐driven senescence phenotypes (Figure 6B), indicating that STAT1 is critically involved in driving cells into senescence under oxidative stress, and its suppression can partially mitigate stress‐induced senescence. Consistent with this, immunoblot analysis showed that STAT1 depletion suppressed TBHP‐induced upregulation of p53 and reduced expression of the inflammasome adaptor ASC (Figure 6C,D). To further assess inflammasome activation, intracellular cleaved caspase‐1 and mature IL‐1β were examined by Western blot, and IL‐1β secretion in culture supernatants was quantified by ELISA. TBHP treatment increased both intracellular inflammasome activation markers and IL‐1β secretion, whereas STAT1 knockdown attenuated these effects (Figure 6D,E). We next examined STAT1 subcellular dynamics. In TBHP‐induced senescent cells, STAT1 exhibited nuclear translocation, shifting from a predominantly cytoplasmic pattern in controls to pronounced nuclear accumulation. This TBHP‐induced nuclear localization was largely abolished by STAT1 knockdown (Figure 6F), consistent with impaired STAT1‐dependent transcriptional activation and attenuated downstream senescence‐ and inflammasome‐associated signaling.

**FIGURE 6:**
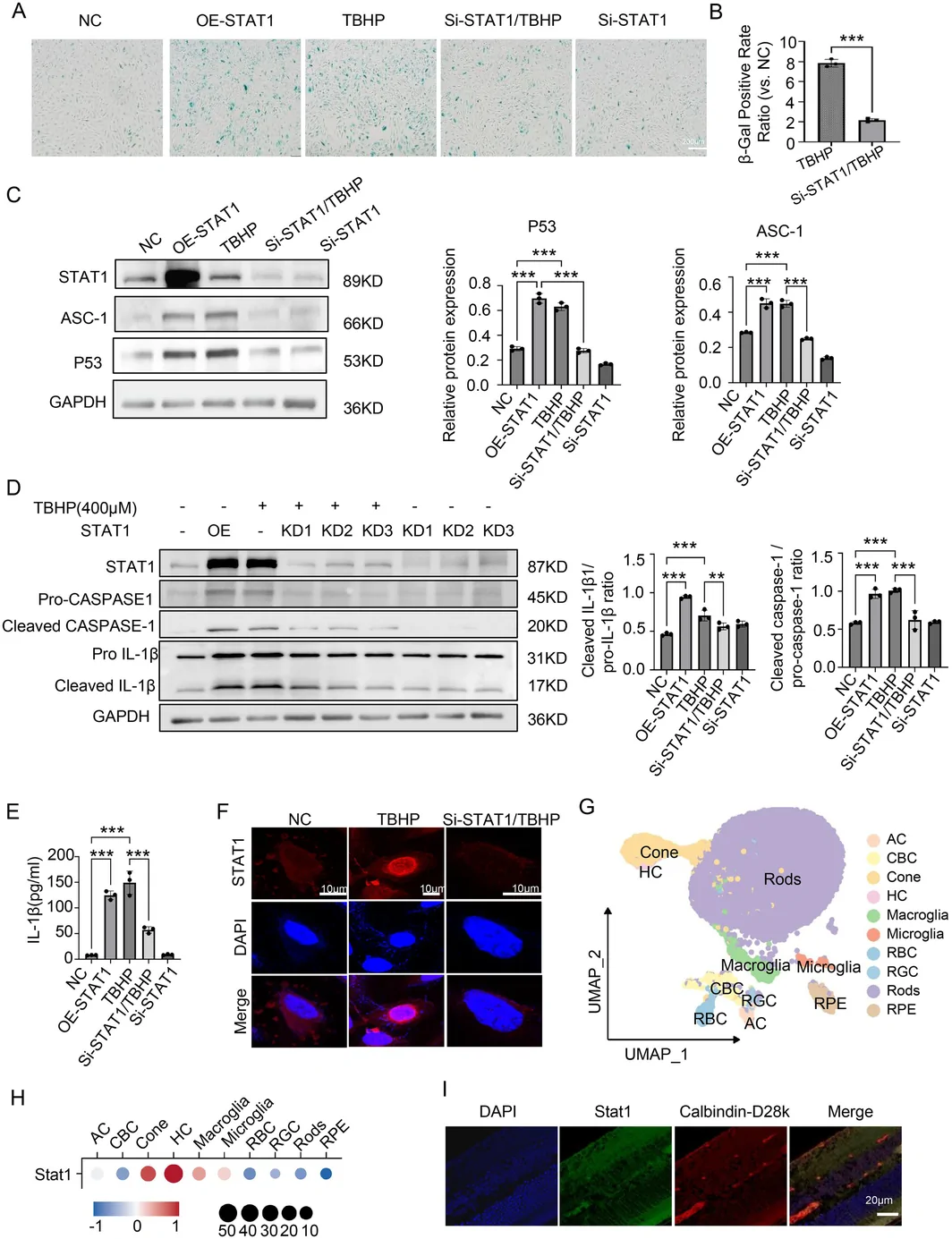
STAT1 modulates oxidative stress‐induced senescence and shows cell type–specific expression in the aging retina. (A) Representative senescence‐associated β‐galactosidase (SA‐β‐gal) staining of ARPE‐19 cells under different conditions: Control (NC), TBHP‐induced senescence, STAT1 overexpression (OE‐STAT1), STAT1 knockdown (si‐STAT1), and STAT1 knockdown combined with TBHP treatment (si‐STAT1 + TBHP). Scale bars: 100 μm. (B) The proportion of SA‐β‐gal–positive (blue‐stained) cells was quantified and normalized to the NC group for both the TBHP and si‐STAT1/TBHP groups. Data are presented as the mean ± SD from three independent experiments. (C) Western blot analysis of STAT1, p53, and the inflammasome adaptor ASC in ARPE‐19 cells following TBHP treatment. Knockdown of STAT1 suppresses stress‐induced upregulation of p53 and ASC. (D) Representative Western blot analysis of intracellular inflammasome‐associated proteins in ARPE‐19 cells under the indicated conditions. Quantitative analysis of the cleaved caspase‐1/pro‐caspase‐1 ratio and mature IL‐1β/pro‐IL‐1β ratio is shown in the accompanying bar graphs. Protein expression levels were normalized to GAPDH and are presented as the mean ± SEM from three independent biological replicates. Statistical significance was determined using a two‐tailed unpaired Student's *t*‐test (*p < 0.05, **p < 0.01, ***p < 0.001). (E) ELISA quantification of secreted IL‐1β levels in culture supernatants collected from ARPE‐19 cells following TBHP treatment and STAT1 knockdown. Data are presented as the mean ± SEM from three independent biological replicates. Statistical significance was determined using a two‐tailed unpaired Student's *t*‐test. (F) Immunofluorescence analysis of STAT1 subcellular localization in ARPE‐19 cells. Under TBHP‐induced senescence, STAT1 exhibits pronounced nuclear translocation compared with NC cells. In contrast, STAT1 knockdown abrogates TBHP‐induced nuclear accumulation of STAT1, with the protein remaining predominantly cytoplasmic. Nuclei were counterstained with DAPI. Scale bar: 10 μm. (G) UMAP visualization of mouse retinal cells, identifying major populations including rods, cones, rod bipolar cells (RBC), cone bipolar cells (CBC), horizontal cells (HC), amacrine cells (AC), retinal ganglion cells (RGC), microglia, macroglia, and retinal pigment epithelium (RPE). (H) Dot plot showing cell type–specific Stat1 expression, with the highest expression in horizontal cells and moderate expression in microglia, macroglia, and cone photoreceptors. (I) Representative images showing nuclear staining (blue), the horizontal cell marker Calbindin‐D28k (red), and STAT1 (green), with the merged image on the right. STAT1 signal is enriched in horizontal cells, as indicated by the overlap between STAT1 and the horizontal cell marker. Scale bar: 20 μm.

### 2.8 Stat1 Signaling Is Preferentially Activated in Horizontal and Glial Cells of the Aging Retina

To define the cellular context of Stat1 activation in vivo, we analyzed a published aging retina single‐cell RNA‐seq dataset from (Liu et al. 2025). UMAP projection identified 10 major cell clusters—rods, cones, rod bipolar cells, cone bipolar cells, amacrine cells, horizontal cells, retinal ganglion cells, microglia, macroglia, and retinal pigment epithelium cells—with clear separation and confident annotation (Figure 6G, Figure S12A). Mapping Stat1 transcripts revealed peak expression in horizontal cells, with moderate levels in microglia, macroglia, and cones, and low abundance in other populations (Figure 6H). Immunofluorescence co‐staining in mouse retinal sections confirmed strong Stat1 signal in horizontal cells (Figure 6I). In contrast to Stat1 itself, expression of Stat1 target genes was most prominently elevated in microglial, macroglial, and retinal pigment epithelial cells, with additional activation in specific neuronal populations, including cones, bipolar cells, and rods (Figure S12B). Together, these data suggest that Stat1 induction is concentrated in horizontal and glial compartments during retinal aging, whereas its downstream transcriptional program is engaged across multiple retinal cell types.

## 3 Discussion

By integrating proteomic, transcriptomic, and single‐cell transcriptomic analyses, we delineate a comprehensive molecular framework of natural retinal aging and identify Stat1 as a central upstream regulator linking oxidative stress, immune activation, and senescence programs (Figure 7). Our findings move beyond descriptive catalogs of age‐related changes and instead define a regulatory node that coordinates multi‐layer molecular reprogramming across retinal cell types.

**FIGURE 7:**
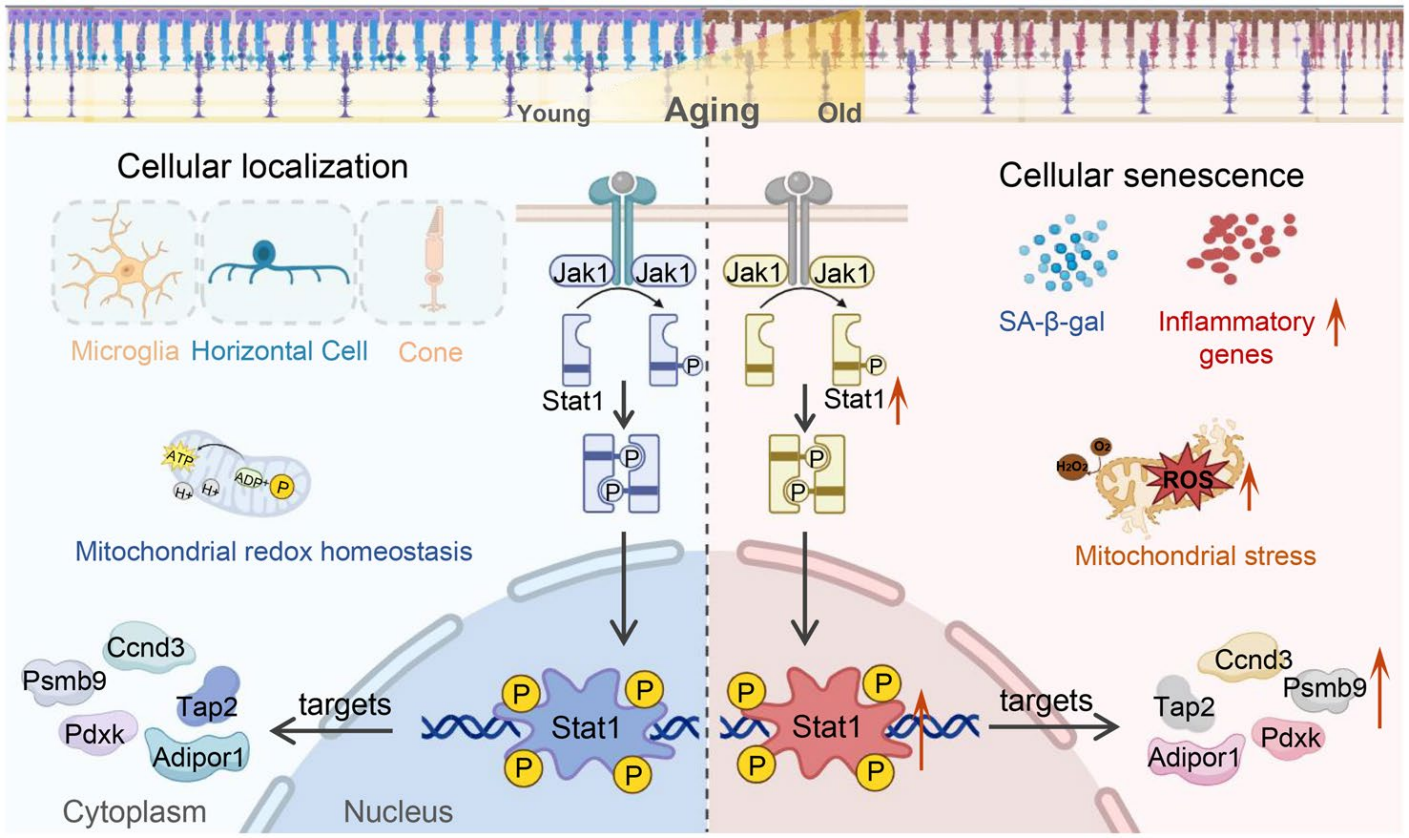
Age‐associated activation of STAT1 signaling in retinal aging. In young retinas, STAT1 displays low basal activity with limited nuclear translocation and is predominantly localized in microglia and horizontal cells, thereby maintaining retinal cellular homeostasis. With aging, STAT1 phosphorylation progressively increases, accompanied by enhanced nuclear accumulation. In aged retinas, sustained STAT1 activation promotes the transcription of downstream target genes, including Psmb9, Tap2, Adipor1, Ccnd3, and Pdxk, leading to increased inflammatory responses, elevated reactive oxygen species (ROS) production, and mitochondrial dysfunction. These molecular alterations ultimately drive cellular senescence, as evidenced by increased SA‐β‐gal activity, upregulation of canonical senescence markers (p53 and p21), and elevated expression of inflammation‐associated genes (ASC), collectively contributing to retinal aging and degeneration.

The aging retina exhibited widespread molecular remodeling, encompassing immune activation, metabolic reprogramming, and progressive loss of structural and functional integrity. The identification of 292 DEPs and 1002 DEGs highlights pervasive dysregulation across regulatory layers. Notably, the moderate correlation between proteomic and transcriptomic changes (Pearson's *r* = 0.603) underscores a key principle of aging biology: RNA‐level alterations alone do not fully capture functional states in aged tissues. This divergence is consistent with emerging evidence from large‐scale aging mouse proteome atlases, which revealed that post‐transcriptional dysregulation becomes pronounced in late life—particularly beyond 24 months—manifesting as the accumulation of core extracellular matrix proteins and depletion of mitochondrial membrane proteins across multiple tissues (Takasugi et al. 2024). In line with this, increased intron retention has been recognized as a conserved post‐transcriptional hallmark of aging and Alzheimer's disease (Adusumalli et al. 2019). In the retina, our observation of opposite RNA‐protein trends for extracellular matrix components (Col4a1, Col4a2, Nid2) provides tissue‐specific evidence for age‐associated proteostatic imbalance, reinforcing the utility of integrative proteogenomic analysis in resolving the complex regulatory architecture of retinal aging.

Functionally, aging was characterized by coordinated activation of antigen processing and presentation, interferon signaling, apoptosis, and small‐molecule biosynthesis, alongside repression of phototransduction, retinol metabolism, and cytoskeletal organization. These reciprocal shifts between immune–metabolic activation and visual function mirror trends reported in aged human retina (Butler et al. 2021) and RPE (Wang et al. 2023), but here are captured with molecular and pathway‐level resolution in a naturally aged murine model. Importantly, SASP signatures were not uniformly activated, suggesting marked cellular heterogeneity in aging responses, consistent with tissue‐specific aging dynamics described in other organ systems (Cohn et al. 2023). Such variation may explain why broad SASP suppression has yielded mixed results in age‐related interventions (Saliev and Singh 2025).

Metabolic rewiring emerged as a prominent feature of retinal aging. The observed upregulation of oxidoreductases such as Maob and Cyp27a1, together with reduced expression of enzymes including Gulo and Dct, suggests a shift in redox homeostasis and mitochondrial metabolism. Similar age‐dependent metabolic remodeling has been reported in human RPE and aged retinas, including increased oxidative stress (Dubey et al. 2025), dysregulated visual cycle genes (Butler et al. 2021), and enhanced immune signatures (Lee et al. 2021). While such changes may represent compensatory responses to chronic oxidative stress, they could paradoxically intensify ROS accumulation and lipid peroxidation (Zhao et al. 2023; Schumann et al. 2025). The concurrent dysregulation of transmembrane receptors (e.g., S1pr3, Lpar1, Adipor1) further indicates impaired lipid and sphingolipid signaling, processes known to influence both photoreceptor survival and glial activation during aging (Simon et al. 2021; Lewandowski et al. 2022; Yung et al. 2015; Jung et al. 2025).

A central advance of this study is the identification of STAT1 as a regulatory hub that integrates these age‐dependent stress pathways. STAT1 is a canonical effector of interferon signaling and inflammation and has been implicated in multiple age‐related pathologies. While previous retinal studies noted increased interferon‐responsive transcriptional signatures with age, the regulatory status, activation dynamics, and functional relevance of STAT1 at the protein level remained unresolved. Our multi‐omics data demonstrate concordant upregulation of STAT1 at both the transcript and protein levels in the aged retina, supported by increased STAT1 abundance in oxidative stress‐induced senescent human RPE cells. This convergence across molecular layers distinguishes STAT1 from downstream inflammatory markers and supports its role as an upstream driver of retinal aging.

Mechanistically, our data reveal that STAT1 function during oxidative stress–induced senescence is governed not only by abundance but also by subcellular localization. Immunofluorescence analysis revealed a pronounced nuclear translocation of STAT1 in ARPE‐19 cells following TBHP‐induced senescence, whereas STAT1 knockdown effectively abolished this stress‐induced nuclear accumulation. These findings suggest that oxidative stress not only elevates STAT1 expression but also promotes its functional activation through nuclear translocation, thereby enabling STAT1 to exert transcriptional control over senescence‐ and inflammation‐related gene programs. Importantly, the suppression of p53 upregulation and ASC expression upon STAT1 knockdown supports a model in which STAT1 nuclear activity acts upstream of both senescence signaling and inflammasome priming. This spatial regulation of STAT1 provides mechanistic insight into how oxidative stress drives a coordinated senescence–inflammatory response and reinforces the notion that STAT1 functions as an active driver rather than a passive marker in retinal aging‐associated cellular stress responses.

Single‐cell transcriptomic analysis further contextualized Stat1 activity within the cellular architecture of the aging retina (Lukowski et al. 2019). Stat1 expression was enriched in horizontal cells and was also detectable in cones, microglia, and Miller glia, suggesting that its involvement extends beyond canonical immune cell populations. Notably, the cellular distribution of Stat1 transcripts did not fully overlap with that of its downstream target genes, which were more prominently upregulated in microglial, macroglial, and retinal pigment epithelial cells. This pattern suggests that Stat1 transcript abundance and downstream transcriptional output may be dissociated across retinal cell types. Such divergence may reflect differences in cell‐type‐specific sensitivity to STAT1‐associated signaling, or indirect, non‐cell‐autonomous propagation of inflammatory responses (Gautam et al. 2021) within the aging retina. Together, these findings support a model in which Stat1‐related retinal inflammaging is spatially organized and shaped by distinct cellular contexts.

In summary, our study provides an integrated, multi‐layered view of retinal aging and identifies STAT1 as a convergent upstream regulator linking oxidative stress, metabolic dysregulation, senescence, and inflammation. By coupling systems‐level mapping with mechanistic validation, we establish STAT1 as a driver of retinal aging rather than a correlative marker. This work offers a conceptual framework for dissecting complex aging networks in neural tissues and nominates STAT1 signaling as a promising, cell‐context‐dependent therapeutic target to mitigate retinal inflammaging and preserve visual function in later life.

## 4 Methods

### 4.1 Experimental Mice

C57BL/6J wild‐type mice were obtained from Beijing Vital River Laboratory Animal Technology Co. Ltd. and maintained under controlled conditions in our laboratory. Animals were housed under specific pathogen‐free (SPF) conditions in a temperature‐controlled environment (22°C ± 2°C) with a 12‐h light/dark cycle and had *ad libitum* access to standard chow and water. Both male and female mice were used unless otherwise specified. All animal experiments were conducted in accordance with the Association for Research in Vision and Ophthalmology (ARVO) Statement for the Use of Animals in Ophthalmic and Vision Research, and all procedures were approved by the Animal Ethics Committee of Henan Eye Hospital/Henan Eye Institute (Approval No. HNEEC‐2020).

### 4.2 Tissue Collection

Mice were euthanized by cervical dislocation. The bilateral eyeballs were carefully enucleated with fine forceps and immediately transferred into pre‐cooled 1× PBS. Under a dissecting microscope, a small incision was made at the corneal margin with a scalpel, and the globe was opened along the limbus. The cornea and lens were removed using ophthalmic forceps, and the retina was gently dissected to minimize mechanical damage.

### 4.3 Cellular Senescence Models

Human retinal pigment epithelial cells (ARPE‐19) were cultured in DMEM/F12 medium supplemented with 10% fetal bovine serum (FBS) under standard conditions (37°C, 5% CO_2_). For oxidative stress‐induced senescence, cells were seeded at a density of 1.2 × 10^5^ cells/mL and serum‐starved for 24 h prior to a 2 h exposure to 400 μM tert‐butyl hydroperoxide (t‐BHP) (B802372; Macklin) in serum‐free medium, followed by a 48 h recovery period in complete medium. For genotoxic stress‐induced senescence, cells were plated at a density of 1.0 × 10^5^ cells/mL and treated with 250 nM Doxorubicin (HY‐15142; MedChemExpress) in complete medium for 72 h, after which they were maintained in fresh medium for an additional 7 days. Senescence was validated by senescence‐associated β‐galactosidase (SA‐β‐gal) staining using a Senescence β‐Galactosidase Staining Kit (C0602; Beyotime) according to the manufacturer's instructions. These concentrations were selected based on preliminary dose–response experiments and previous reports demonstrating robust senescence induction without excessive cytotoxicity.

### 4.4 Protein Extraction and Mass Spectrometry Analysis

Retinal tissues were homogenized in lysis buffer (8 M urea, 25 mM TEAB) using a high‐throughput tissue grinder (HF‐48; Shanghai Hefan). Protein concentration was determined using a Bradford assay (HXJ5137; Huaxingbio) with BSA standard. For digestion, proteins were reduced with 5 mM DTT (M109‐5G; Amresco) at 37°C for 1 h, alkylated with 10 mM iodoacetamide (M216‐30G; Amresco) in the dark for 45 min, and digested with trypsin (V5280, Promega) at a 1:50 enzyme‐to‐protein ratio overnight at 37°C. Peptides were desalted using C18 ZipTips (ZTC18M096; Millipore), activated with 100% acetonitrile and equilibrated with 0.1% formic acid (T79708; Sigma‐Aldrich).

### 4.5 TMT Labeling and LC–MS/MS Analysis

Peptides were labeled using TMT16plex reagents (A44520; Thermo Fisher) according to the manufacturer's protocol. Briefly, 100 μg of peptides were incubated with a TMT reagent dissolved in 41 μL of acetonitrile for 1 h at room temperature, followed by quenching with 5% hydroxylamine. Labeled peptides were fractionated using an XBridge Peptide BEH C18 column (186,003,581; Waters) on an L‐3000 HPLC system (RIGOL) with a 72‐min gradient (5%–95% acetonitrile in 0.1% formic acid). Fractions were analyzed on an Orbitrap Exploris 480 mass spectrometer equipped with a FAIMS Pro interface, using an 80‐min gradient (7%–100% acetonitrile) at a flow rate of 600 nL/min. MS spectra were acquired at a resolution of 60,000 (m/z 350–1500) with data‐dependent acquisition (TopSpeed mode, 22 ms injection time, 36% collision energy). Raw files were processed using Proteome Discoverer 2.4 with the *Mus musculus* database, allowing two missed cleavages, 15 ppm precursor and 0.02 Da fragment mass tolerances, including carbamidomethylation (C) as a fixed modification and oxidation (M), TMT labeling (K and N‐terminus), and acetylation (N‐terminus) as variable modifications.

### 4.6 Transcriptomics Analysis

Total RNA was extracted from retinal tissue samples using TRIzol reagent. RNA yield and purity were assessed using a NanoDrop 2000 spectrophotometer (Thermo Scientific), and integrity was evaluated by an Agilent 2100 Bioanalyzer (5067–1511, RNA 6000 Nano Kit). Sequencing libraries were constructed using the TruSeq Stranded mRNA LT Sample Prep Kit (Illumina) and sequenced on the Illumina NovaSeq 6000 platform for mouse retinal samples, generating 150 bp paired‐end reads. Raw reads were aligned to the mm10 mouse reference genome. Differentially expressed genes were identified using DESeq2 with an FDR < 0.05 and |log_2_ fold change| ≥ 1. All RNA‐seq analyses were based on biological replicates.

### 4.7 UID‐Based RNA Sequencing and Data Processing of ARPE‐19 Cells

Total RNA from ARPE‐19 cells was extracted using TRIzol reagent followed by DNase I treatment. RNA quality and quantity were assessed using the NanoDrop, Qubit fluorometry, and the LabChip GX Touch system. UID‐based stranded mRNA‐seq libraries were prepared using the KC‐Digital mRNA‐seq Library Prep Kit and sequenced on a DNBSEQ‐T7 platform in paired‐end 150 bp mode. Raw reads were quality‐filtered and adapter‐trimmed, followed by UMI extraction and UMI‐based deduplication to generate consensus reads. Clean reads were aligned to the human reference genome (GRCh38). Gene‐level counts were generated, and differential expression analysis was performed using edgeR with *p* < 0.05 and |fold change| ≥ 2.

### 4.8 SASP Gene Set and GenAge Lifespan‐Related Protein Analysis

Senescence‐associated secretory phenotype (SASP) gene sets were obtained from MSigDB (www.gsea‐msigdb.org/gsea/index.jsp). Gene set scores were calculated using the AddModuleScore function in Seurat (v4.1.0) for transcriptomic datasets, including bulk and single‐cell RNA‐seq data. Early‐onset senescence subpopulations were identified based on elevated SASP scores. Mouse lifespan‐related genes (*n* = 137) were downloaded from the GenAge database (GenAge Build 21, https://genomics.senescence.info/genes/models.html). Protein identifiers were matched to gene symbols, and DEPs with overlapping GenAge genes were identified. Significant changes were determined with *p* ≤ 0.05 and fold change ≥ 1.2.

### 4.9 Single‐Cell RNA‐Seq Analysis

Publicly available aging mouse retina scRNA‐seq datasets (Liu et al. 2025) were analyzed using Seurat v4.1.0. Low‐quality cells were filtered, and data were normalized and scaled. PCA and UMAP dimensionality reduction were performed. Cell clusters were annotated based on canonical markers (e.g., Gnat1 for rods, Rpe65 for RPE, Hexb for microglia). The expression of Stat1 and its 25 interacting genes was evaluated by scaled expression and AddModuleScore to infer cell‐type‐specific regulatory patterns.

### 4.10 Identification of Candidate Transcription Factors and Regulatory Network Construction

Differentially expressed proteins between young and aged retinas were annotated using public regulatory resources (AnimalTFDB v4.0 (Shen et al. 2023) and HOCOMOCO v14 (Vorontsov et al. 2024)) to identify transcription factors or proteins with DNA‐binding motif evidence. Proteins annotated as transcription factors in both databases were retained as candidate upstream regulators. For each candidate transcription factor, target genes were obtained from the Cistrome Data Browser (Taing et al. 2024) and TFlink (Liska et al. 2022). In TFlink, only transcription factor–target interactions supported by curated experimental evidence or literature annotation were retained. In the Cistrome Data Browser, candidate target genes were selected based on transcription factor‐binding predictions with a regulatory score > 0. Target genes from different resources were merged after gene symbol harmonization, and duplicated entries were removed to generate a unified target gene set for each transcription factor. To evaluate the relevance of each candidate transcription factor to retinal aging, each transcription factor‐specific target‐gene set was intersected with the DEGs identified in the aged retina transcriptomic dataset. Regulatory coverage was defined as the number of overlapping genes between the target‐gene set and aging‐associated DEGs vs. aging‐associated DEGs.

Candidate transcription factors were ranked according to regulatory coverage and enrichment significance. STAT1 was prioritized because it showed the highest regulatory coverage, significant enrichment among aging‐associated DEGs, and increased abundance in the aged retina proteomic dataset. The STAT1‐centered regulatory network was constructed and visualized using Cytoscape v3.9.1 based on overlapping STAT1 target genes differentially expressed in the aged retina.

### 4.11 Automated Capillary‐Based Western Immunoblotting

Retinal tissues were collected from 4‐month‐old (young group) and 24‐month‐old (aged group) C57BL/6J mice, with five independent biological replicates per group. Total retinal proteins were extracted using lysis buffer supplemented with protease and phosphatase inhibitors. Protein concentrations were determined using a BCA protein assay. Automated capillary‐based Western immunoblotting was performed using the Jess Simple Western system (ProteinSimple, Bio‐Techne, San Jose, CA, USA) according to the manufacturer's instructions.

Protein lysates were diluted with 0.1× sample buffer and mixed with fluorescent 5× master mix containing molecular weight markers and dithiothreitol to obtain a final protein concentration of 0.5 μg/μL. Samples were denatured at 95°C for 5 min and loaded into a 12–230‐kDa Jess separation module. The assay plate was loaded with the samples, a biotinylated molecular weight ladder, a blocking reagent, wash buffer, primary antibodies, horseradish peroxidase‐conjugated secondary antibodies, a chemiluminescent substrate, and a peroxide reagent. Proteins were separated by size within the capillaries and immobilized using the instrument's photoactivated capture chemistry. Capillaries were then sequentially incubated with primary antibodies against p‐Stat1 (ET1611‐20; HUABIO, 1:100), Stat1 (#14994; Cell Signaling Technology, 1:50), Psmb9 (ET7107‐24; HUABIO, 1:100), and β‐actin (AB2287; Abcam, 1:200), followed by the corresponding HRP‐conjugated secondary antibodies.

Chemiluminescent signals were acquired and analyzed using Compass for Simple Western software. Results were visualized as electropherograms and virtual blot images. Peak areas were automatically calculated by the software, and target protein abundance was quantified based on chemiluminescence peak areas normalized to the corresponding β‐actin signal. Native, non‐editable PDF reports generated directly by the Jess instrument/software system are provided as supporting raw data.

### 4.12 Western Blotting

Proteins were separated using 10% SDS‐PAGE precast gels and transferred electrophoretically onto polyvinylidene difluoride (PVDF) membranes (Millipore). Membranes were blocked with 5% skim milk in TBST for 1 h at room temperature, followed by incubation with primary antibodies JAK1 (ET1705‐84, HUABIO), P‐JAK1 (#YP0154, Immuno way), STAT1 (#14994, Cell Signaling Technology), P‐STAT1 (ET1611‐20, HUABIO), TAP2 (HA723859, HUABIO), p53 (ET1601‐13, HUABIO), ADIPOR1 (14361–1‐AP, Proteintech), PDXK (15309–1‐AP, Proteintech), CCND3 (ET1612‐4, HUABIO), APOA2 (ET7107‐47, HUABIO), PSMB9 (ET7107‐24, HUABIO), and GAPDH (ET1601‐4, HUABIO) overnight at 4°C. After three washes with TBST, membranes were incubated with HRP‐conjugated secondary antibodies (EM2001‐04, HUABIO) for 1 h at room temperature. Protein bands were detected using the Bio‐Rad enhanced chemiluminescence detection system (GelView 6000Plus, Biolight), and signal intensities were quantified by densitometric analysis with ImageJ software.

### 4.13 Measurement of IL‐1β Secretion by ELISA

After establishing different cell models, the cells were transferred to serum‐free DMEM/F12 medium and cultured for 24 h. The cell supernatants were collected and ELISA assays were performed according to the kit instructions (Elabscience, E‐EL‐H0149, Wuhan, China). All experiments were independently repeated three times, and the data were statistically analyzed by two‐tailed unpaired *t*‐test.

### 4.14 Immunofluorescence

ARPE‐19 cells were fixed with 4% paraformaldehyde (PFA) for 15 min and permeabilized with 0.1% Triton X‐100 for 10 min. Blocking was performed with 5% bovine serum albumin (BSA) in PBS for 1 h at room temperature. Cells were then incubated overnight at 4°C with primary antibodies against STAT1 (ET1606‐39, HUABIO), p53 (ET1601‐13, HUABIO), and Calbindin‐D28k (66394‐1‐Ig; Proteintech). Following three washes with PBS, cells were incubated with Alexa Fluor–conjugated secondary antibodies (ab150077; Abcam) for 1 h at room temperature. Nuclei were counterstained with DAPI (F6057; Sigma). Fluorescence images were captured using a confocal laser scanning microscope (Nikon system, 20× objective), with identical acquisition parameters applied consistently across all experimental groups.

### 4.15 Detection of Intracellular ROS

Intracellular reactive oxygen species (ROS) levels were measured using a ROS Assay Kit (CM‐H2DCFDA, Beyotime) according to the manufacturer's protocol. Briefly, cells were seeded into 6‐well plates and, following model establishment, incubated with a 10 μM DCFH‐DA probe diluted in serum‐free medium (1:1000) for 20 min at 37°C. Excess probe was removed by washing cells three times with serum‐free medium. As a positive control, Rosup (Beyotime) was added to induce ROS production for 20–30 min. Fluorescence signals were then visualized using an inverted fluorescence microscope with excitation at 488 nm and emission at 525 nm.

### 4.16 RNA Extraction and cDNA Synthesis

Total RNA was extracted using RNA Extraction Reagent (G3013; Servicebio) according to the manufacturer's protocol. Briefly, 1 mL TRIzol was added to each sample, followed by 0.2 mL chloroform and incubation for 15 min at room temperature. Samples were centrifuged at 12,000 × ***g*** for 15 min at 4°C, and the aqueous phase was collected. An equal volume of isopropanol was added, and the mixture was centrifuged again at 12,000 × ***g*** for 15 min at 4°C. The resulting RNA pellet was washed with 75% ethanol, air‐dried, and dissolved in RNase‐free water. Complementary DNA (cDNA) was synthesized from total RNA using the ABScript III RT Master Mix with gDNA Remover (RK20433; ABclonal) following the manufacturer's instructions.

### 4.17 Quantitative Real‐Time PCR (RT–qPCR)

Quantitative real‐time PCR was performed on a Roche LightCycler 96 system using 2× Universal SYBR Green Fast qPCR Mix (RK21203, ABclonal). Relative gene expression was analyzed using the ΔΔC_t_ method with GAPDH as the internal control. The primers used were as follows: *Apoa2*‐Forward: GCAGACGGACCGGATATGC; *Apoa2*‐Reverse: GCTGCTCGTGTGTCTTCTCA; *Adipor1*‐Forward: AATGGGGCTCCTTCTGGTAAC; *Adipor1*‐Reverse: GGATGACTCTCCAACGTCCCT; *Psmb9*‐Forward: GGGACAACCATCATGGCAGT; *Psmb9*‐Reverse: CAGCAGCGGAACCTGAGAG; *Tap2*‐Forward: GACATGGCTTTACTTGGGTTGC; *Tap2*‐Reverse: GTGCCCTCTATCCAGAGTCC; *Pdxk*‐Forward: TCCGTGGCTACGTGGGTAA; *Pdxk*‐Reverse: CATCGTTCACCTTGAGACCCT *Ccnd3*‐Forward: TGCGTGCAAAAGGAGATCAAG; *Ccnd3*‐Reverse: GGACAGGTAGCGATCCAGGT; *Gapdh*‐Forward: AGGTCGGTGTGAACGGATTTG; *Gapdh*‐Reverse: GGGGTCGTTGATGGCAACA.

### 4.18 Bioinformatics and Statistical Analysis

Functional Enrichment: GO/KEGG analyses via Metascape (v3.5). Protein classification by PANTHER (v17.0). Proteomics/transcriptomics: FDR‐adjusted *t*‐test (FDR < 0.05). All experiments were conducted with at least three independent biological replicates. Data are presented as the mean ± SEM. Statistical analyses were performed using GraphPad Prism (v9.0) or R. Two‐tailed Student's *t*‐tests were used for comparisons between two groups, and one‐way ANOVA followed by appropriate post hoc tests was used for multiple‐group comparisons. For omics data, statistical significance was determined using FDR‐adjusted tests as specified above. Data are presented as mean ± SEM unless otherwise indicated. *p* < 0.05 was considered statistically significant.

## Author Contributions

B.L., B.H., and X.J. conceived the study of the subject and designed the experiments; Y.F., YI. L., and Y.L. performed the experiments; Y.F., L.C., X.J., R.Y., and X.W. analyzed the results; X.J. and Y.F. wrote the manuscript; B.L., B.H., Z.L., and X.J. reviewed and revised the article. All authors have read and approved the final version of this article.

## Funding

This work was supported by Henan Province Youth Health Science and Technology Innovation Talent Training Program, YQRC2023007. National Natural Science Foundation of China grants, 82271084, 82004001. Medical Science and Technology Program of Health Commission of Henan Province, SBGJ202302097. Henan Provincial Science and Technology Research and Development Program Joint Fund, 242301420067. Henan Academy of Innovations in Medical Science, JBKY250311, Eye Institute 20250203. Project of Basic Research of Henan Eye Hospital, 23JCZD001.

## Conflicts of Interest

The authors declare no conflicts of interest.

## Supporting information


**Data S1:** acel70792‐sup‐0001‐Supinfo.pdf.

**Figure S1:** Proteomic analysis of lifespan‐related genes in aging mouse retina. (A) Heatmap of *Z*‐score‐normalized expression for differentially regulated lifespan‐related proteins in young and aged mouse retinas. Rows represent proteins, columns represent samples, and hierarchical clustering of samples is indicated at the top. Proteins significantly altered with aging (*p* < 0.05) are indicated, and statistical significance is summarized by −log_10_(*p*‐values). Lifespan‐associated annotations were derived from the GenAge database, including proteins annotated as lifespan‐decreasing (e.g., Arntl, Eef1e1, Hnrnpd, Rae1, Bub3, Clock, and Efemp1) and lifespan‐increasing (e.g., Gsta4, Mif, Coq7, Ctf1, Akt1, Akt2, Gpx4, Surf1, and Igf1r). (B) Venn diagram illustrating the overlap between the total proteome (7155 proteins) and the GenAge protein set (136 proteins), identifying 67 overlapping proteins. (C) Differentially expressed proteins (DEPs) among the 67 overlapping GenAge‐associated proteins in aging retinas. Thirty‐one proteins exhibited significant age‐dependent changes, including 11 down‐regulated and 20 up‐regulated proteins.
**Figure S2:** Workflow of screening and candidate transcription factor ranking analysis. Differentially expressed proteins (DEPs) identified from proteomic analysis of young (4 M) and aged (24 M) mouse retinas were annotated using AnimalTFDB and HOCOMOCO to identify candidate transcription factors. Target‐gene sets for candidate transcription factors were obtained from TFlink and the Cistrome Data Browser. Candidate target genes were intersected with aging‐associated differentially expressed genes (DEGs) identified from transcriptomic analysis. Regulatory coverage was calculated as the proportion of overlapping genes between transcription factor target‐gene sets and aging‐associated DEGs. Based on this workflow, STAT1 was identified as the top‐ranked candidate transcription factor associated with retinal aging.
**Figure S3:** Genome browser visualization of Stat1 occupancy at representative aging‐associated genes. Genome browser tracks showing enrichment of Stat1 binding at transcription start sites (TSS) of representative aging‐associated genes (Psmb9, Tap2, Apoa2, and Ccnd3), based on publicly available Stat1 ChIP‐seq datasets obtained from the Cistrome database.
**Figure S4:** Quantification of Stat1, p‐Stat1, and Psmb9 protein expression in young and aged mouse retinas. Retinal tissues from 4‐month‐old mice (young group, Y‐1 to Y‐5) and 24‐month‐old mice (aged group, O‐1 to O‐5) were analyzed by capillary‐based Simple Western assay. Protein abundance was quantified based on integrated chemiluminescence peak areas generated by Compass for Simple Western software and normalized to the corresponding β‐actin signal. Data are presented as mean ± SEM (*n* = 5 biological replicates per group). Statistical significance was determined using a two‐tailed unpaired Student's *t*‐test. (*ns, not significant; **p < 0.01; ***p < 0.001).
**Figure S5:** Quantitative analysis of protein expression in different senescence models. (A) Quantification of protein levels of P‐JAK1, JAK1, STAT1, P‐STAT1, P53, P21, and P16 in control (NC) and doxorubicin‐induced senescent cells. (B) Quantification of protein levels of STAT1, P53, P21, and P‐STAT1 in control (NC) and TBHP‐treated cells. Protein expression levels were normalized to GAPDH and are presented as mean ± SEM from independent biological replicates. Statistical significance is indicated (*p < 0.05, **p < 0.01, ***p < 0.001).
**Figure S6:** Distinct GO biological process enrichment patterns in STAT1 overexpression and oxidative stress–induced senescence models. Gene Ontology (GO) biological process enrichment analysis was performed using differentially expressed genes (DEGs) identified in STAT1‐overexpressing (OE) ARPE‐19 cells and TBHP‐induced senescent cells. Significantly enriched GO terms are ranked by −log_10_(*p‐*value). GO terms enriched in the STAT1 overexpression group (red) were predominantly associated with interferon signaling, defense response to virus, and interferon‐gamma–mediated signaling pathway, whereas GO terms enriched in the TBHP‐treated group (blue) were mainly related to extracellular matrix organization, cell adhesion, and extracellular structural components, reflecting distinct regulatory programs underlying transcription factor–driven versus oxidative stress–induced senescence.
**Figure S7:** Senescence‐associated β‐galactosidase (SA‐β‐gal) staining of ARPE‐19 cells under STAT1 overexpression conditions. Representative SA‐β‐gal staining images of ARPE‐19 cells transfected with pcDNA3.1 or STAT1 overexpression (OE‐STAT1). Vector control cells exhibited sparse and weak SA‐β‐gal staining, whereas OE‐STAT1 cells showed markedly increased staining intensity and a higher proportion of SA‐β‐gal–positive cells, indicating enhanced senescence induction upon STAT1 overexpression. Scale bar: 200 μm. Figure S7 includes SA‐beta‐gal staining images used for quantification, including the representative images shown in Figure 5F.
**Figure S8:** Intracellular ROS staining in ARPE‐19 cells following STAT1 overexpression. Replicate DCFH/ROS images are shown, including the representative image presented in Figure 5H.
**Figure S9:** Integrated validation of STAT1‐mediated regulation of senescence markers and downstream target genes. (A) Quantification of protein levels of senescence‐associated markers, including P53, P21, and ASC, in control (PC DNA3.1) and STAT1 overexpression (OE‐STAT1) cells. (B) Quantification of protein levels of STAT1 signaling components and downstream targets in control (NC) and doxorubicin‐induced senescent cells, including P‐STAT1, STAT1, TAP2, ADIPOR1, PDXK, CCND3, and PSMB9. Protein expression levels were normalized to GAPDH and are presented as mean ± SEM from independent biological replicates. Statistical significance is indicated (*p < 0.05, **p < 0.01, ***p < 0.001; ns, not significant). (C) RT‐qPCR analysis of gene expression changes in the STAT1 overexpression model and TBHP‐induced senescent cells. Data are presented as mean ± SEM from three independent biological replicates. Statistical significance was determined using a two‐tailed unpaired Student's *t*‐test (*p < 0.05, **p < 0.01, ***p < 0.001).
**Figure S10:** Quantitative analysis of STAT1‐regulated target protein expression. (A) Quantification of protein levels of STAT1 signaling components and downstream targets, including P‐STAT1, STAT1, TAP2, ADIPOR1, PDXK, CCND3, and PSMB9, in control (PC DNA3.1) and STAT1 knockdown (si‐STAT1) cells.(B) Quantification of the same set of proteins in control (PC DNA3.1) and STAT1 overexpression (OE‐STAT1) cells. Protein expression levels were normalized to GAPDH and are presented as mean ± SEM from independent biological replicates. Statistical significance is indicated (*p < 0.05, **p < 0.01, ***p < 0.001).
**Figure S11:** Validation of STAT1 knockdown and its effects on downstream gene expression. (A) ARPE‐19 cells were transfected with three independent siRNA sequences targeting STAT1 or a non‐targeting control siRNA. STAT1 knockdown efficiency was confirmed at the mRNA level by RT‐qPCR and at the protein level by Western blot. (B) The downstream molecular effects of STAT1 depletion were assessed by RT‐qPCR analysis of six STAT1‐associated target genes. Data are presented as mean ± SEM from three independent experiments. Statistical significance was determined by GraphPad Prism 10 (*p < 0.05, **p < 0.01, ***p < 0.001).
**Figure S12:** Marker gene mapping across retinal cell types and intercellular expression correlations of STAT1 downstream targets. (A) Dot plot showing the expression of canonical marker genes used for retinal cell‐type annotation. Representative marker genes (e.g., Nrl, Opn1mw) are displayed across major retinal cell populations, including rods, cones, rod bipolar cells (RBC), cone bipolar cells (CBC), amacrine cells (AC), horizontal cells (HC), retinal ganglion cells (RGC), macroglia, microglia, and retinal pigment epithelium (RPE). Dot Color indicates normalized gene expression levels (scaled from −1 to 2), while dot size represents the proportion of cells expressing each gene within the corresponding cell type (0%–60%). (B) Heatmap illustrating expression correlations between retinal cell types and STAT1 downstream target genes. The y‐axis denotes retinal cell types, and the x‐axis shows STAT1‐associated genes (e.g., Ifit3b, Gsn). Color intensity represents Pearson correlation coefficients (ranging from −1 to 1), with blue indicating negative correlations and red indicating positive correlations. Distinct correlation patterns highlight cell‐type–specific activation profiles of STAT1‐regulated transcriptional programs across the aging retina.

**Table S1:** Quantified transcriptomic profiles and gene expression data.

**Table S2:** Differentially expressed proteins (DEPs) identified in aging mouse retina.

**Table S3:** Differentially expressed genes identified across experimental conditions.

**Table S4:** Candidate transcription factors identified from DEPS using AnimalTFDB and HOCOMOCO annotation.

**Table S5:** Overlap and integration of STAT1 target genes from TFlink and Cistrome with aging‐associated DEGs.

**Table S6:** STAT1 target genes identified by ChIP‐seq analysis from publicly available datasets.

**Table S7:** RNA‐seq–derived differentially expressed genes (DEGs).

## Data Availability

The mass spectrometry proteomics data have been deposited to the ProteomeXchange Consortium (https://proteomecentral.proteomexchange.org) via the iProX partner repository with the dataset identifier PXD072682 (iProX project IPX0014845000) (Ma et al. 2019; Chen et al. 2022). RNA sequencing data have been deposited in the NCBI BioProject database under accession number PRJNA1398564, corresponding to the ARPE‐19 cellular senescence model. Proteomic data from naturally aged mouse retinas are available in the NCBI BioProject database under accession number PRJNA1395546. Other data generated or analyzed during this study are included in this published article and its Supplementary Information Files.
