# Supplementary material for: Multi‐Omics Reveals STAT1 as a Central Regulator of Oxidative Stress‐Induced Senescence in the Aging Retina
Source: Aging Cell. 2026 Nov 7;25(11):e70792. doi: 10.1111/acel.70792 (PMC13744170; doi:10.1111/acel.70792)
Supplement: Supplementary file 1 [file ACEL-25-e70792-s005.pdf]

File: P-Stat1_Jess

# Run

| | |
|---|---|
| | 2026-07-14_19-45-56_Jess |
| | C:\Users\\OneDrive\Desktop |
| | Jess |
| | Regular: 12-230 kDa |
| | |
| | Jess : Jess JS5549 - js5549 |
| | 2.1.2021.09.01.00.26.14.bc9a11c63 |
| | |
| | 1144502115 |
| | 2026067640901030 |
| | 6-2026 |
| | 7:41  7 14, 2026 CST |
| | 10:26  7 14, 2026 CST |
| | None |



proteinsimple a biotechne brand

File: P-Stat1_Jess

## Protocol

| | |
|---|---|
| Separation Matrix | |
| Well Row | |
| Load Time (sec) | |
| Stacking Matrix | |
| Well Row | |
| Load Time (sec) | |
| Sample | |
| Well Row | |
| Load Time (sec) | |
| Separation Time (min) | |
| Separation Voltage (volts) | |
| Standards Exposure (sec) | |
| EE Immobilization Time (sec) | |
| Antibody Diluent Time (min) | |
| Well Row | |
| Primary Antibody Time (min) | |
| Well Row | |
| Secondary Antibody Time (min) | |
| Well Row | |
| Detection | |
| Well Row | |
| CHEMI Detection Profile | |
| Exposure 1 (sec) | |
| Exposure 2 (sec) | |
| Exposure 3 (sec) | |
| Exposure 4 (sec) | |
| Exposure 5 (sec) | |
| Exposure 6 (sec) | |
| Exposure 7 (sec) | |
| Exposure 8 (sec) | |
| Exposure 9 (sec) | |
| Ladder Channel | |

File: P-Stat1_Jess

## Plate Layout

| | 1 | 2 | 3 | 4 | 5 | 6 | 7 | 8 | 9 | 10 | 11 | 12 | 13 | 14 | 15 | 16 | 17 | 18 | 19 | 20 | 21 | 22 | 23 | 24 | 25 |
|---|---|---|---|---|---|---|---|---|---|---|---|---|---|---|---|---|---|---|---|---|---|---|---|---|---|
| A | B... | Y... | Y... | Y... | Y... | Y... | O... | O... | O... | O... | O... | Y... | Y... | Y... | Y... | Y... | O... | O... | O... | O... | O... | Sample | | | |
| B | Antibody Diluent | | | | | | | | | | | | | | | | | | | | | | | | |
| C | A... | Actin | | | | | | | | | | P-Stat1 | | | | | | | | | | Primary | | | |
| D | S... | Secondary | | | | | | | | | | | | | | | | | | | | | | | |
| E | Luminol/Peroxide | | | | | | | | | | | | | | | | | | | | | | | | |

File: P-Stat1_Jess

# Analysis Settings

| | |
|---|---|
| Hidden Capillaries | None |
| Images | |
| CHEMI Luminescence | High Dynamic Range 4.0 |
| Lane Contrast | Slider |
| CHEMI White Level | 24.5 |
| CHEMI Black Level | 10103.7 |
| Peak Fit | fit |
| Apply To | Default, Biot. Ladder |
| Range Min (MW (kDa)) | 1.0 |
| Range Max (MW (kDa)) | 250.0 |
| Range View | Analysis |
| Baseline Type | Spline |
| Baseline Threshold | 1.0 |
| Baseline Window (pixels) | 15.0 |
| Baseline Stiffness | 1.0 |
| Peak Find Threshold | 10.0 |
| Peak Find Width (pixels) | 9.0 |
| Peak Find Area Calculation | Gaussian Fit |
| Ladder Settings | Cap: 1, Chemiluminescent/Fluorescent Channel: CHEMI |
| Apply To | Default |
| Ladder Peak 1 (MW (kDa)) | 12.0 |
| Ladder Peak 2 (MW (kDa)) | 40.0 |
| Ladder Peak 3 (MW (kDa)) | 66.0 |
| Ladder Peak 4 (MW (kDa)) | 116.0 |
| Ladder Peak 5 (MW (kDa)) | 180.0 |
| Ladder Peak 6 (MW (kDa)) | 230.0 |
| Signal To Noise | |
| Algorithm | Signal-to-Noise Ratio |
| Region Spectrum | Full |
| Region Units | kDa |
| Standards | Biotinylated Ladder (12kDa-230kDa) |
| Apply To | Default |
| Standard 1 (MW (kDa)) | 1 |
| Standard 1 Position (pixels) | 120 |
| Standard 1 Fit | no |
| Standard 1 Registration | yes |
| Standard 2 (MW (kDa)) | 29 |
| Standard 2 Position (pixels) | 270 |
| Standard 2 Fit | yes |
| Standard 2 Registration | no |
| Standard 3 (MW (kDa)) | 230 |
| Standard 3 Position (pixels) | 530 |
| Standard 3 Fit | yes |
| Standard 3 Registration | no |
| Advanced | Advanced |
| Apply To | Default |
| Standards Peak Width (pixels) | 15 |
| Standards Allowable Drift (pixels) | 100 |

# File: P-Stat1_Jess

| | |
|---|---|
| Sample Peak Fit Starting Width Ratio | 0.5 |
| Image Median Filter Threshold Ratio | 0.5 |
| Image Median Filter Threshold Limit (counts) | 1000 |

File: P-Stat1_Jess

CHEMI

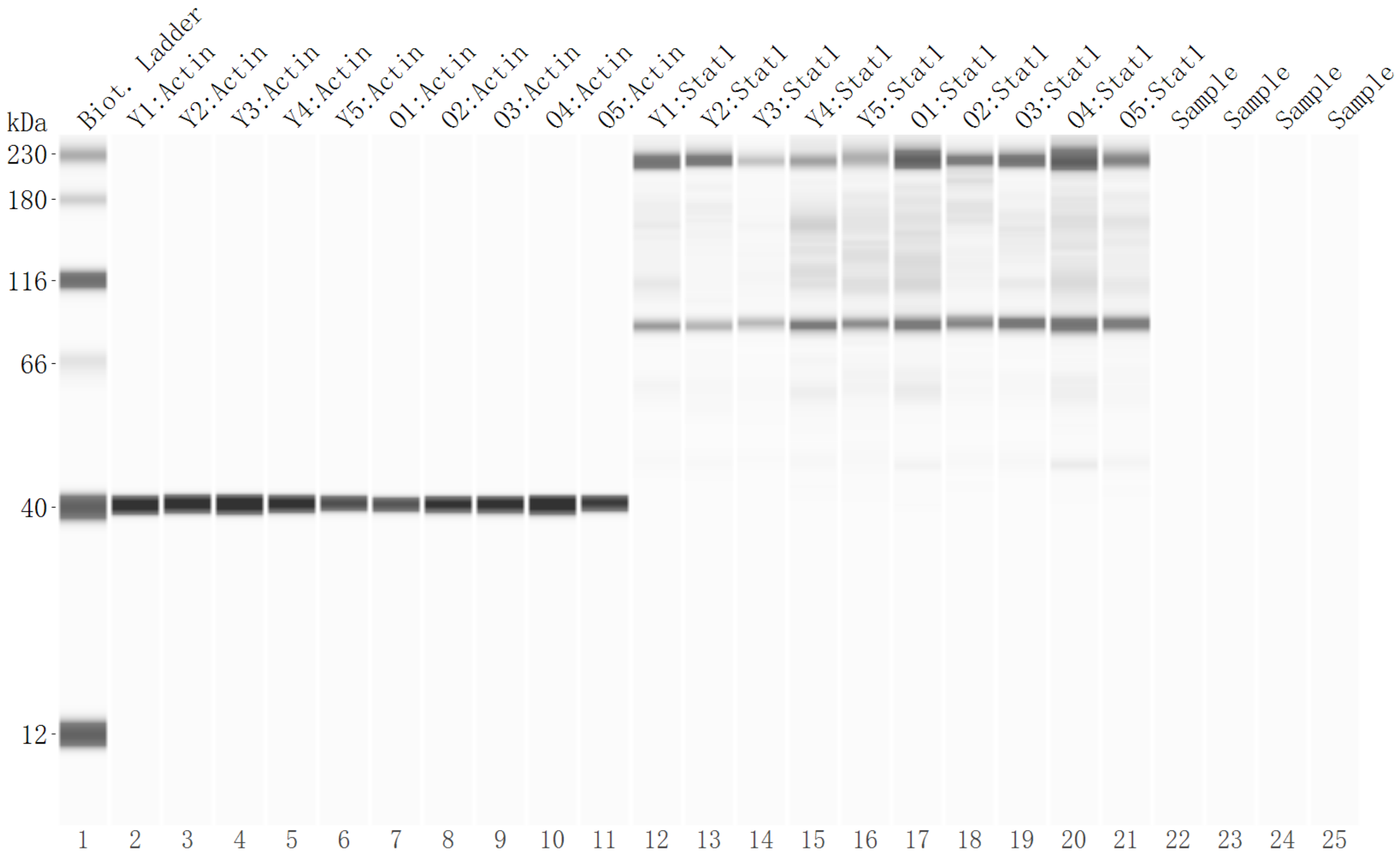


C:\Users\\OneDrive\Desktop\P-Stat1_Jess.cbz

File: P-Stat1_Jess

## Standard

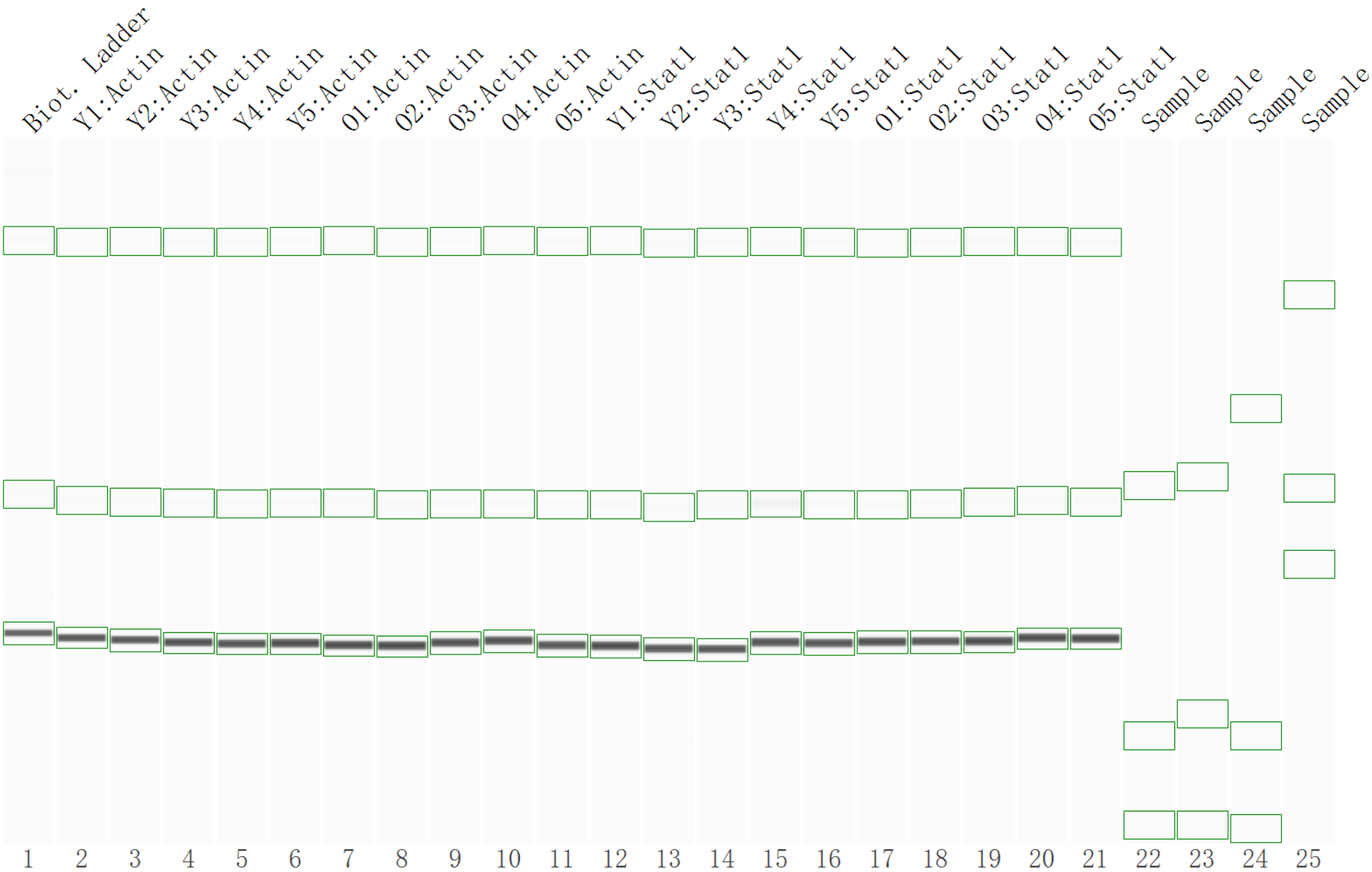

File: P-Stat1_Jess

# IV Plot

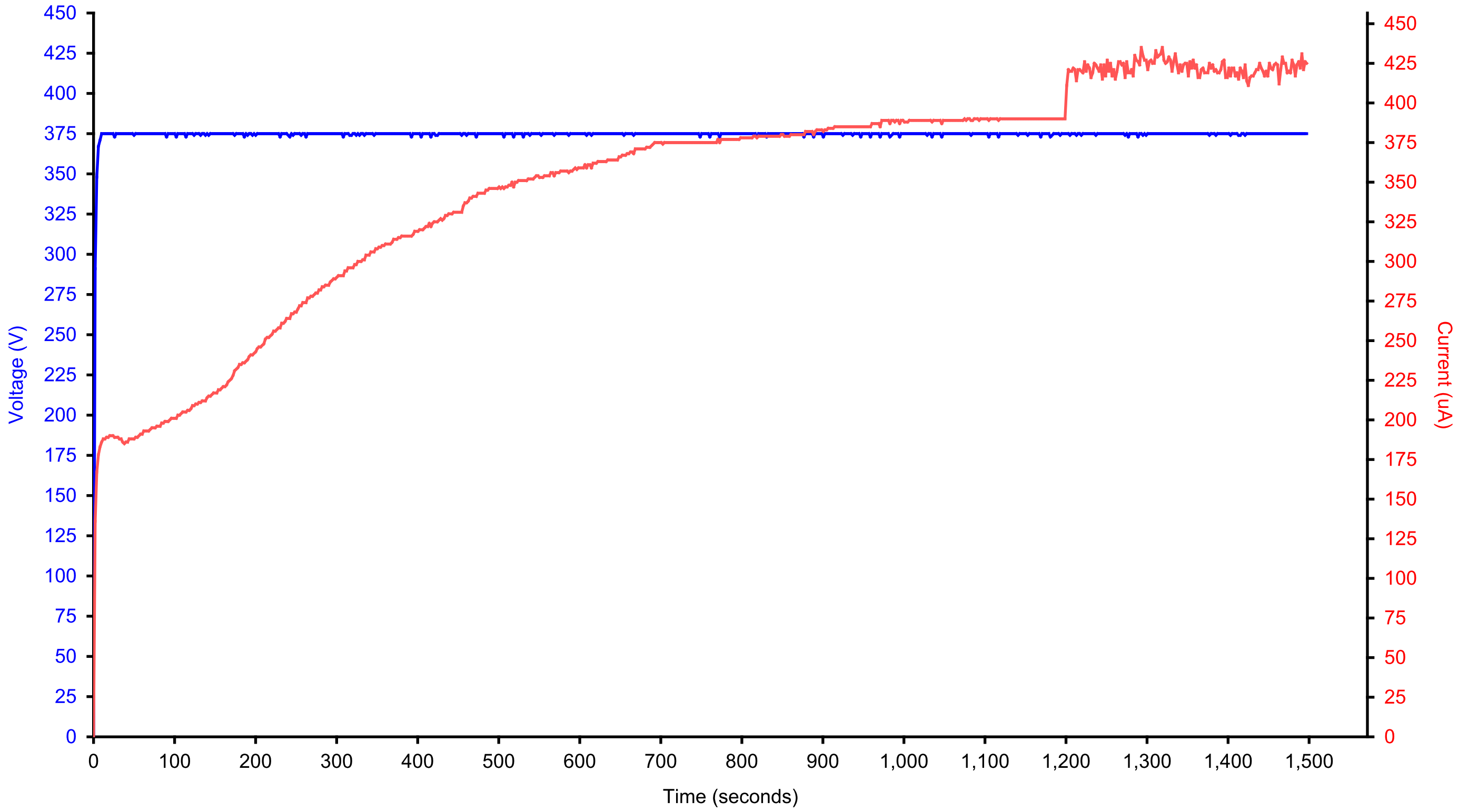

File: P-Stat1_Jess

# CHEMI Sample Plots

Biot. Ladder, Cap:1

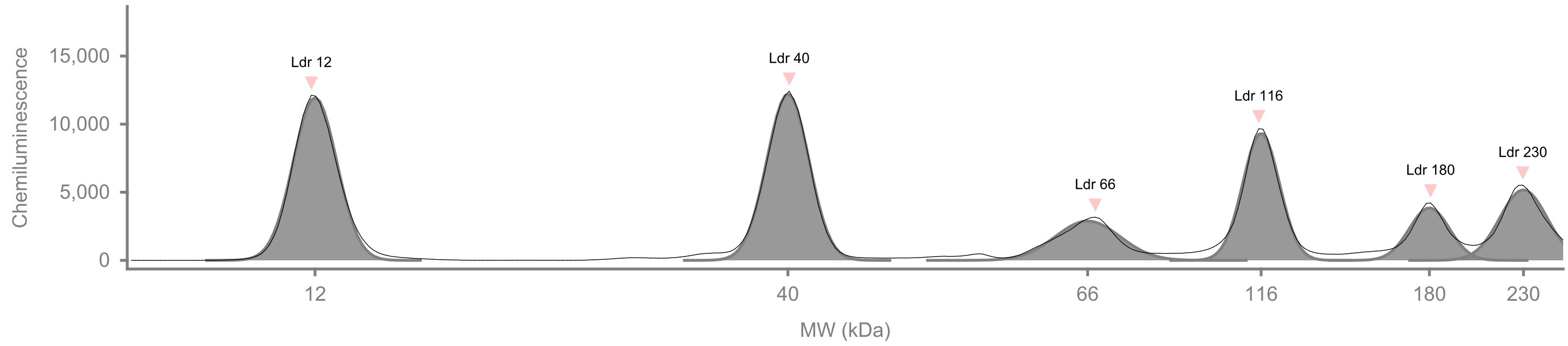


Y1:Actin, Cap:2

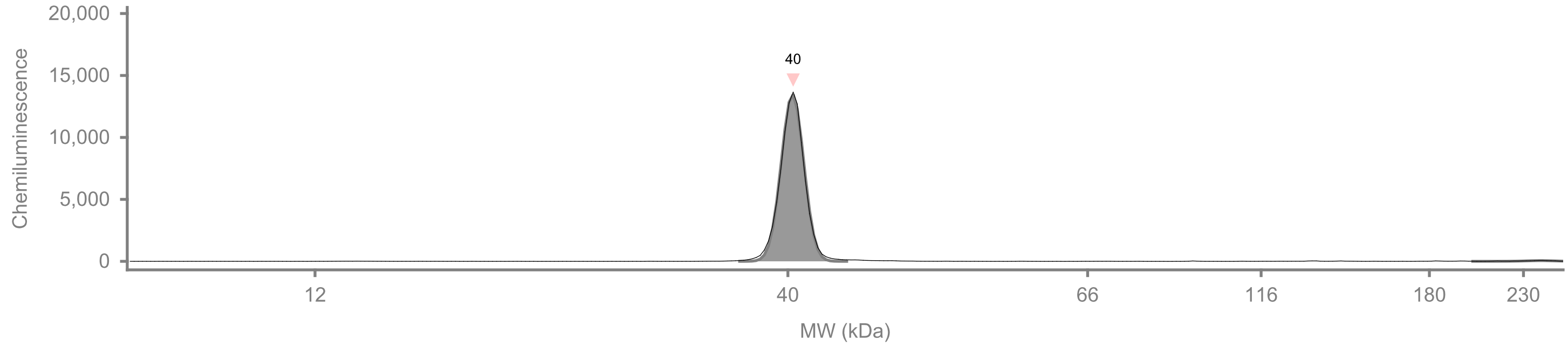


Y2:Actin, Cap:3

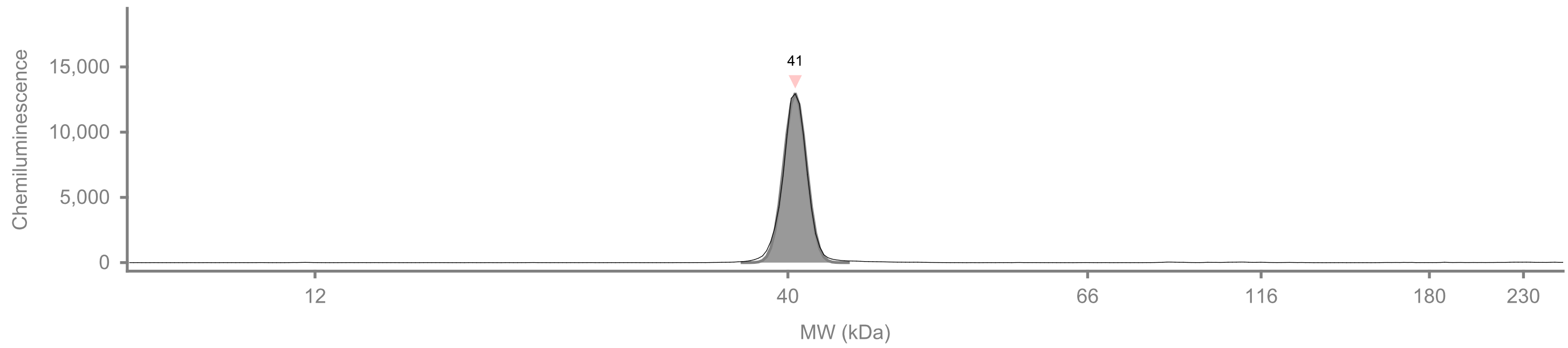


Y3:Actin, Cap:4

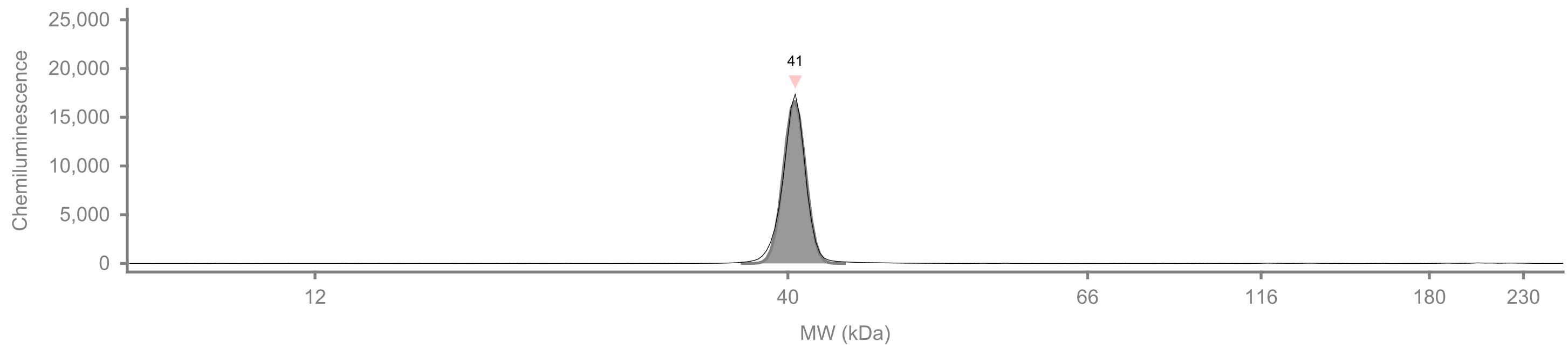




proteinsimple a biotechne brand

# File: P-Stat1_Jess

Y4:Actin, Cap:5

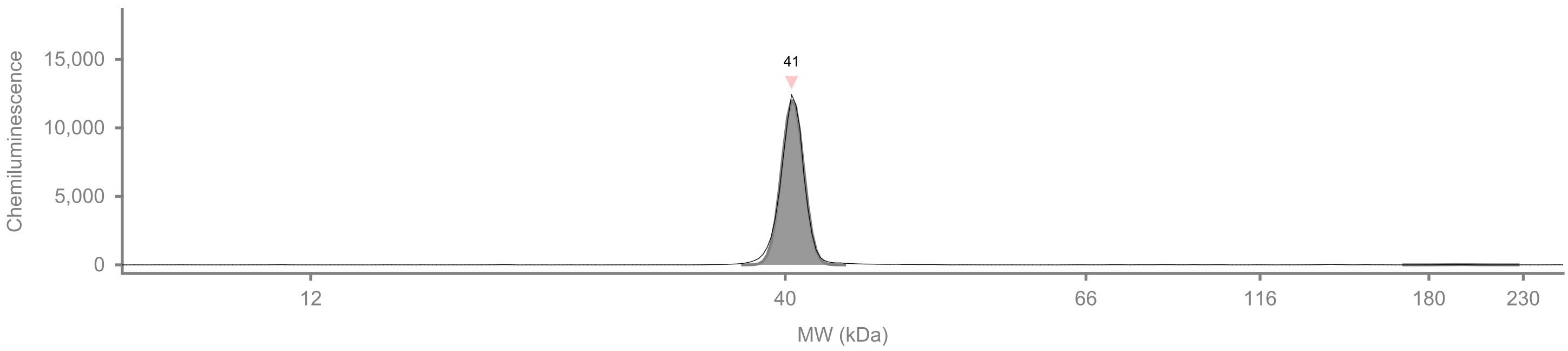


Y5:Actin, Cap:6

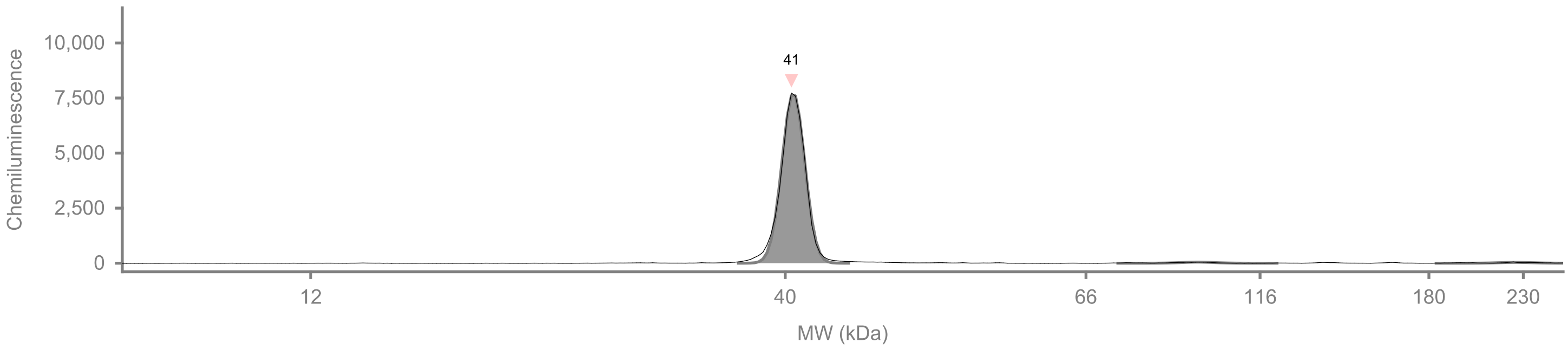


O1:Actin, Cap:7

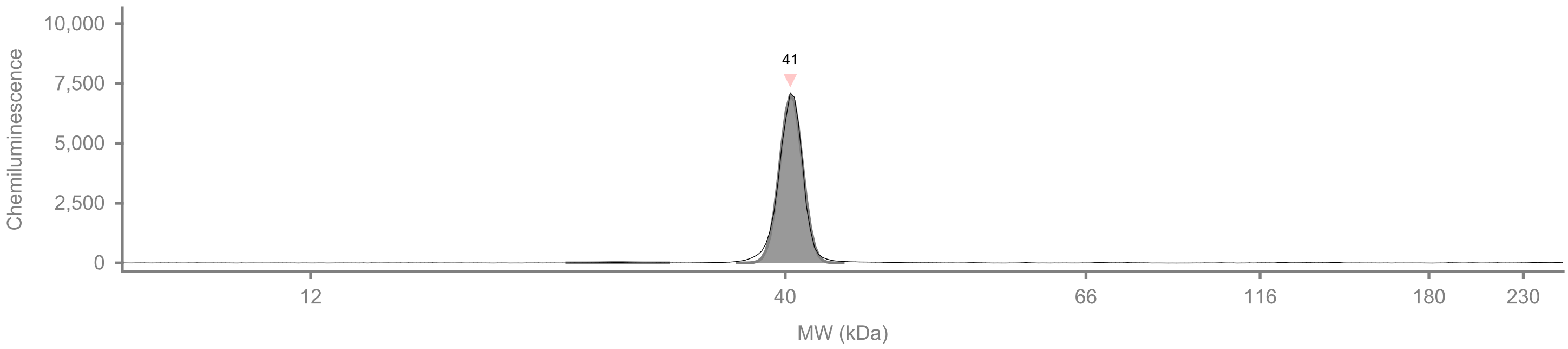


O2:Actin, Cap:8

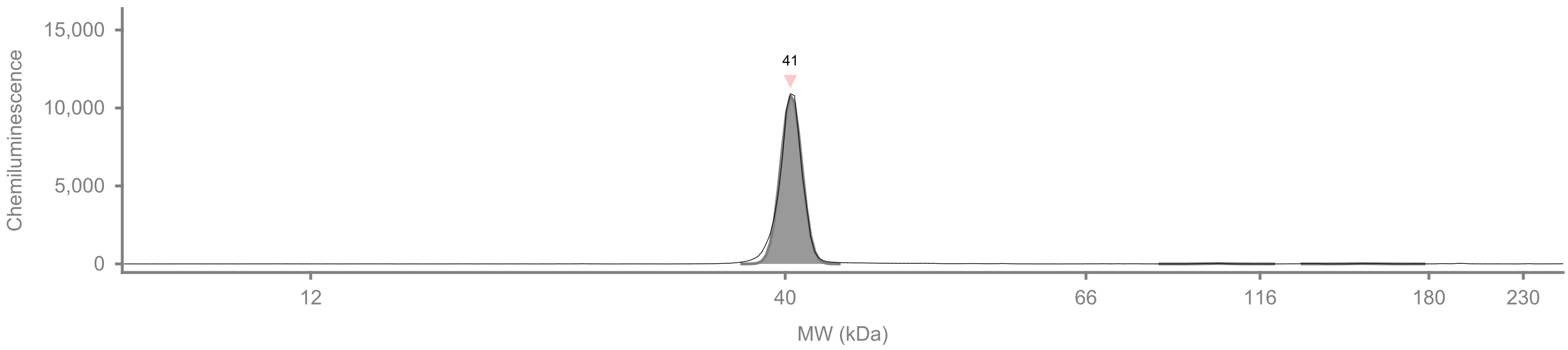

# File: P-Stat1_Jess

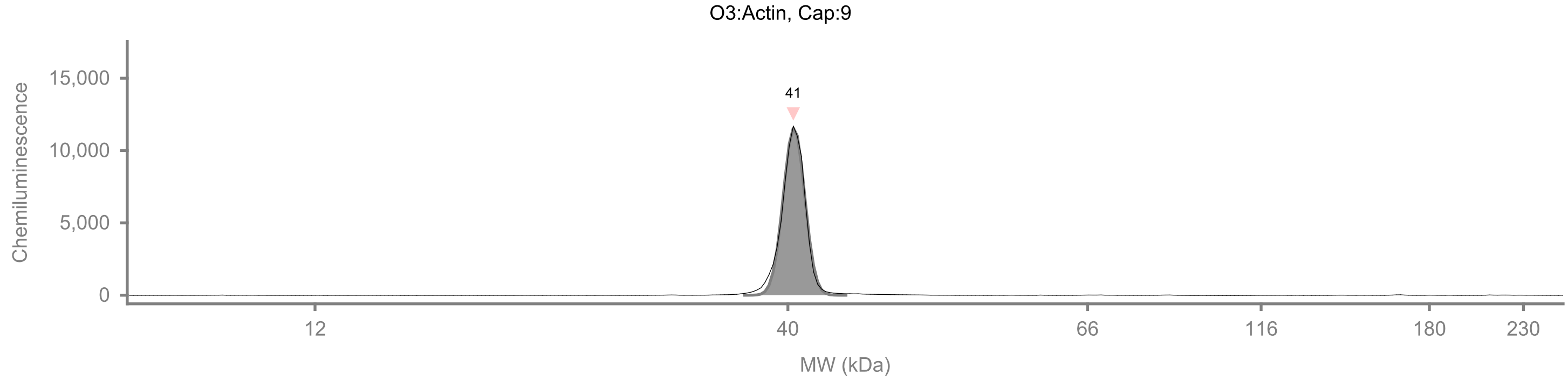


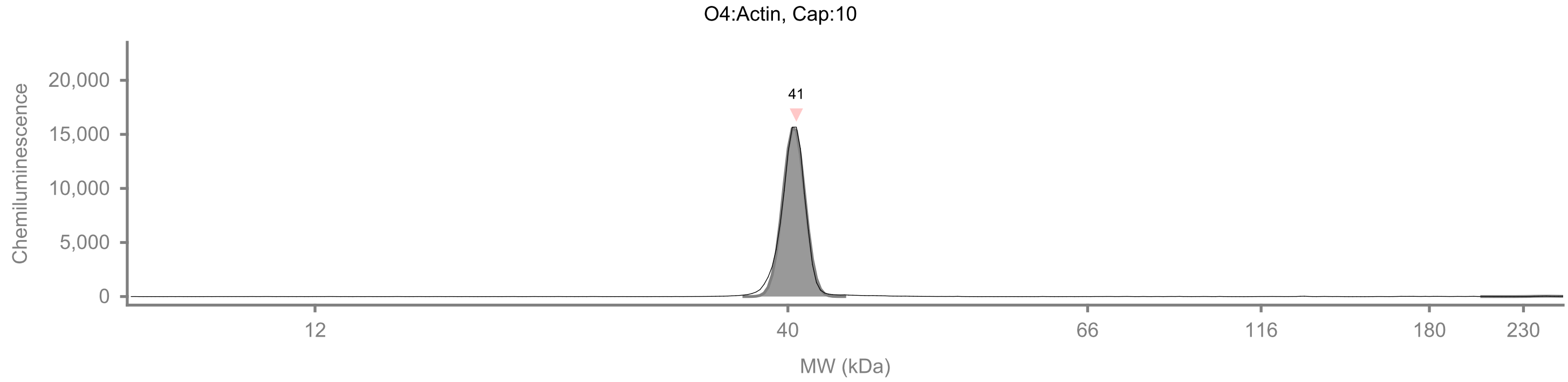


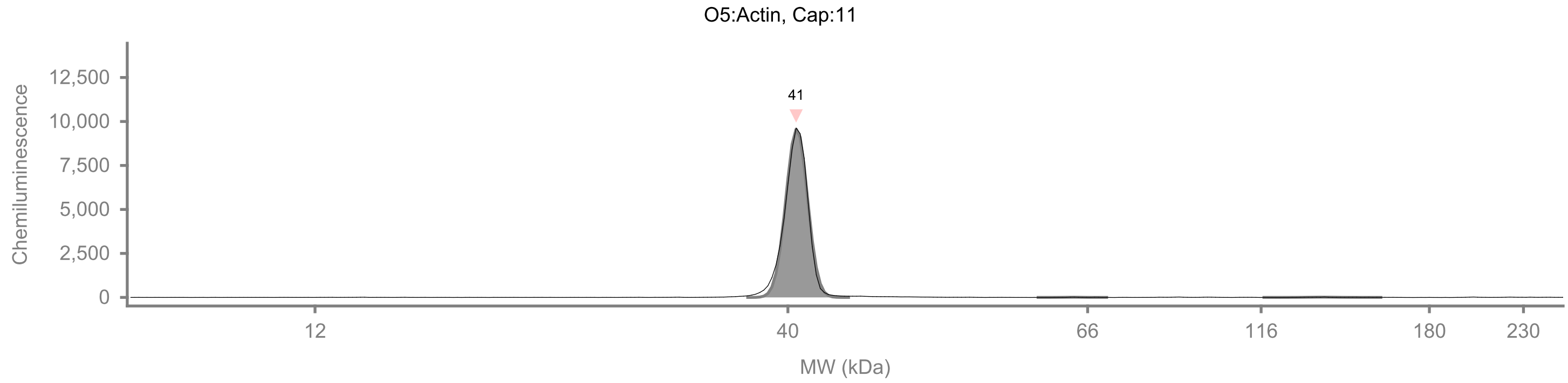


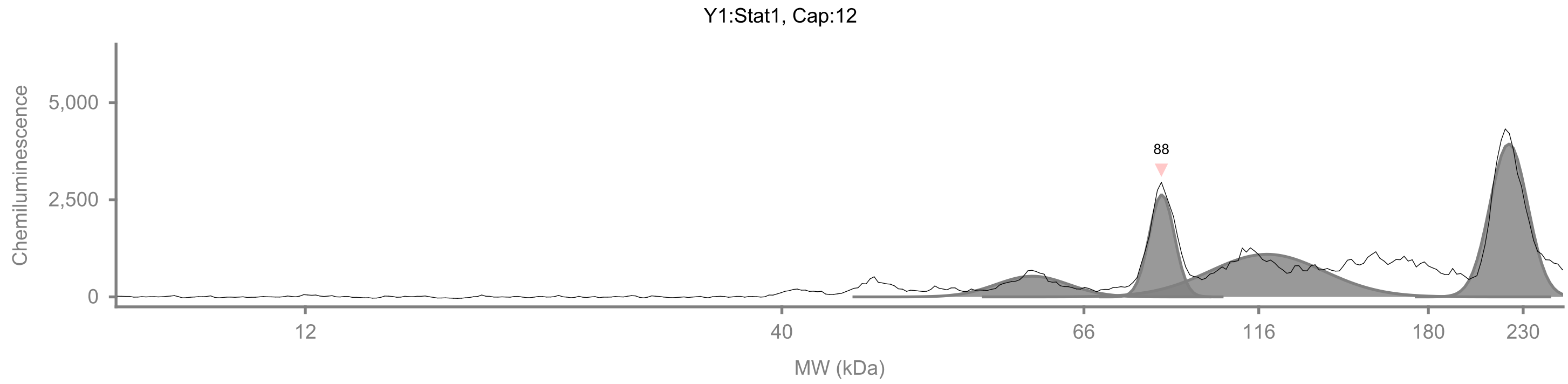

# File: P-Stat1_Jess

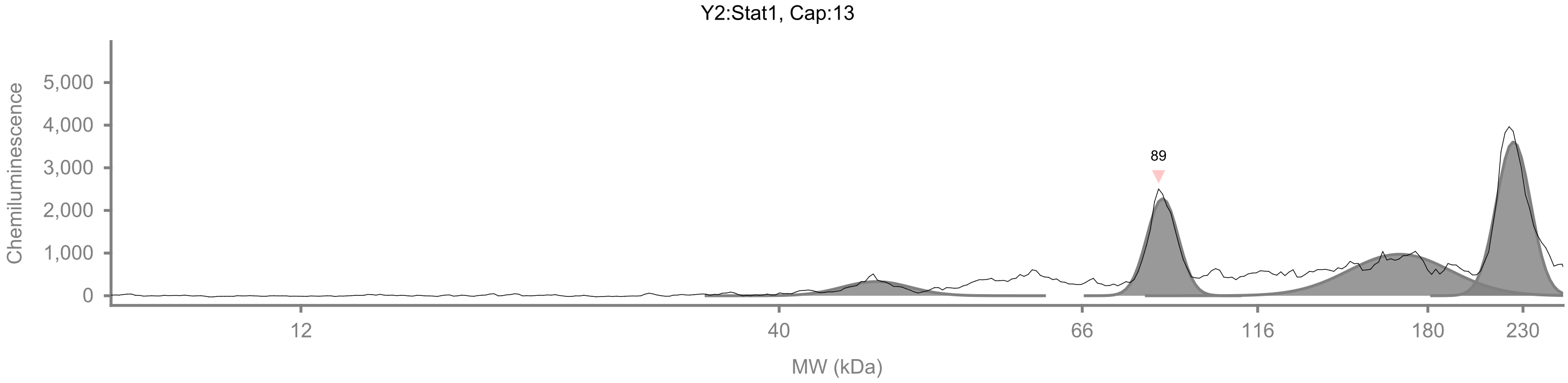


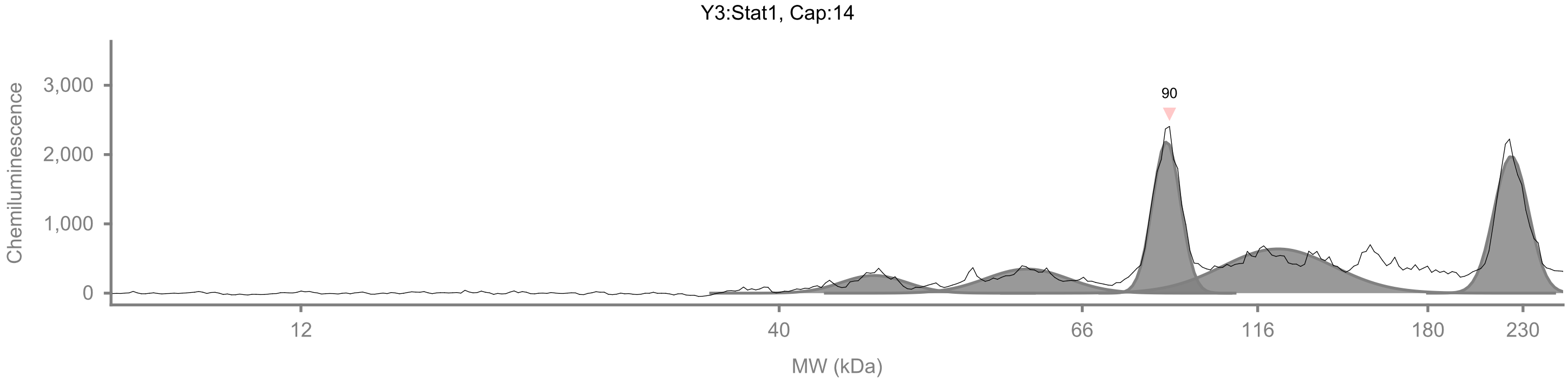


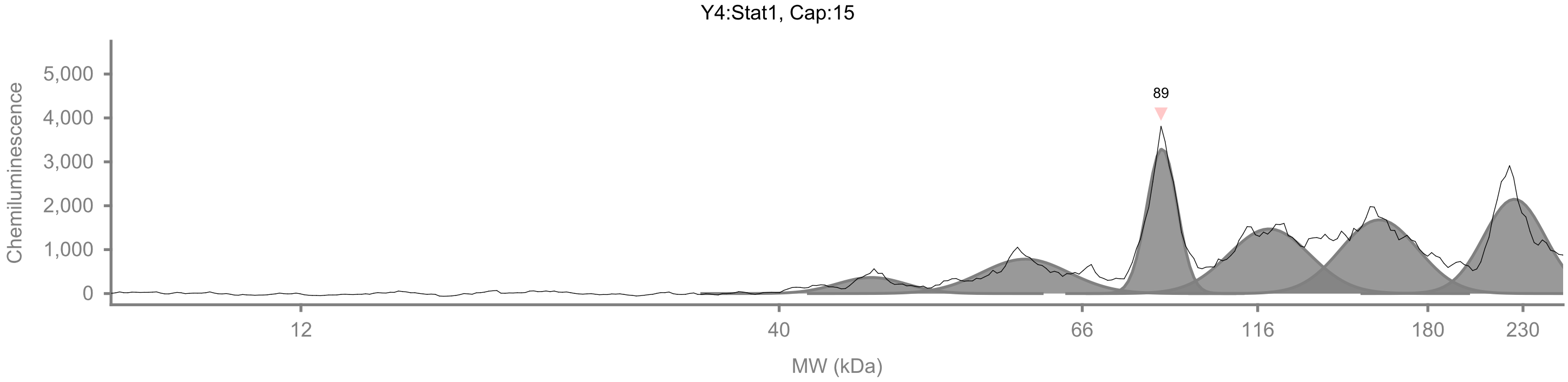


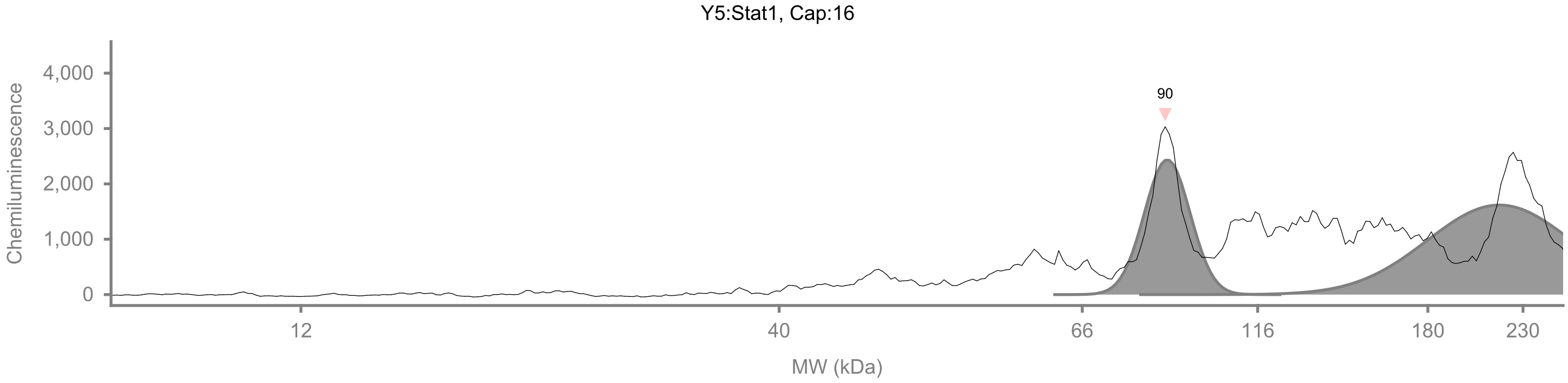

# File: P-Stat1_Jess

O1:Stat1, Cap:17

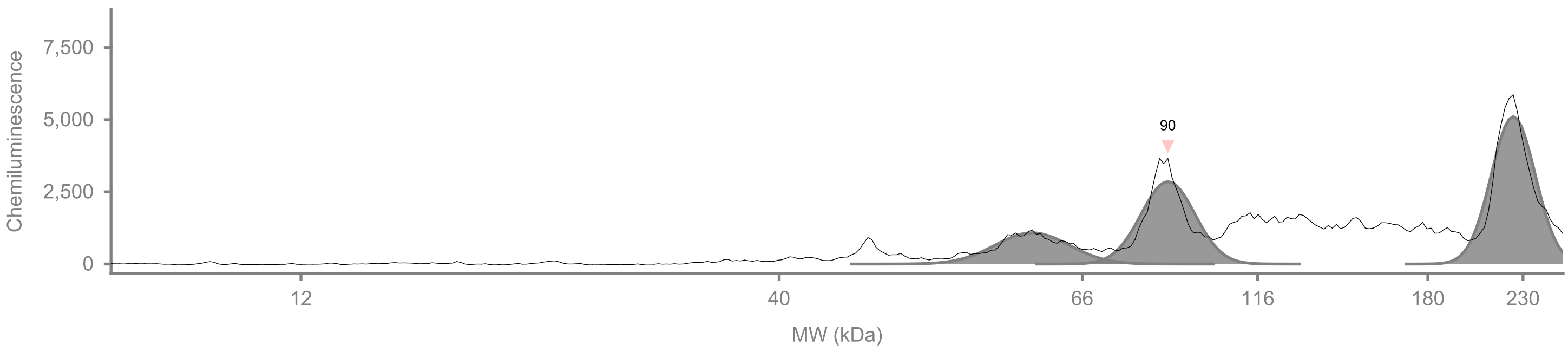


O2:Stat1, Cap:18

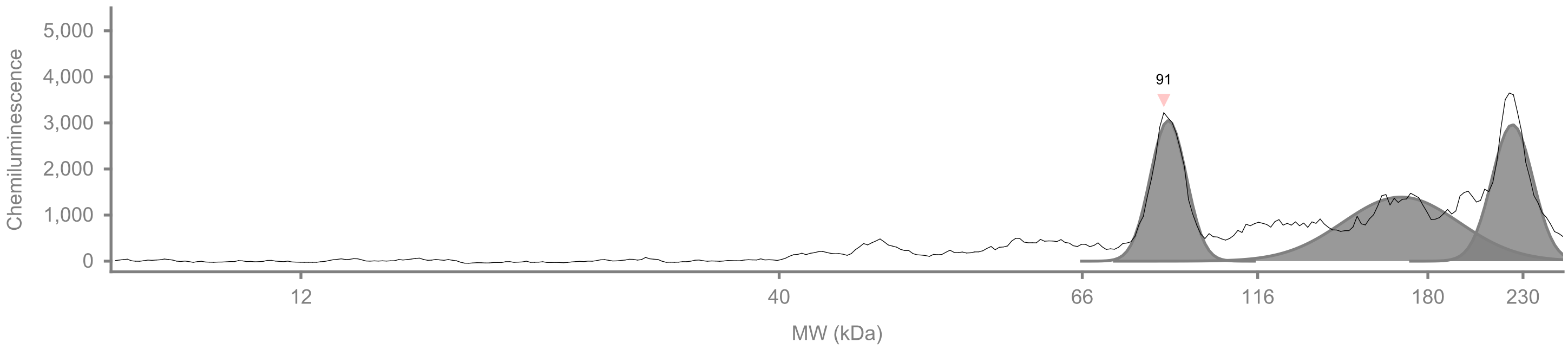


O3:Stat1, Cap:19

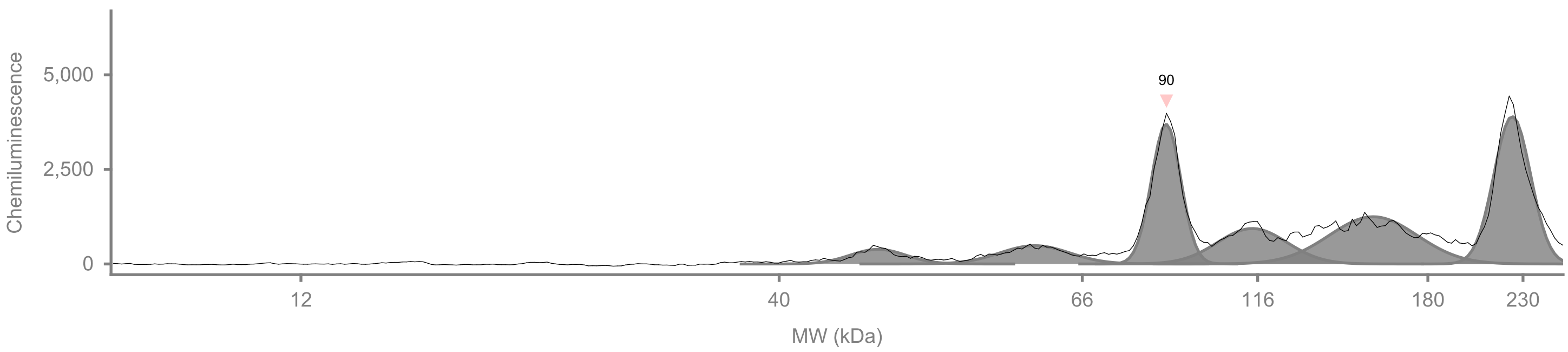


O4:Stat1, Cap:20

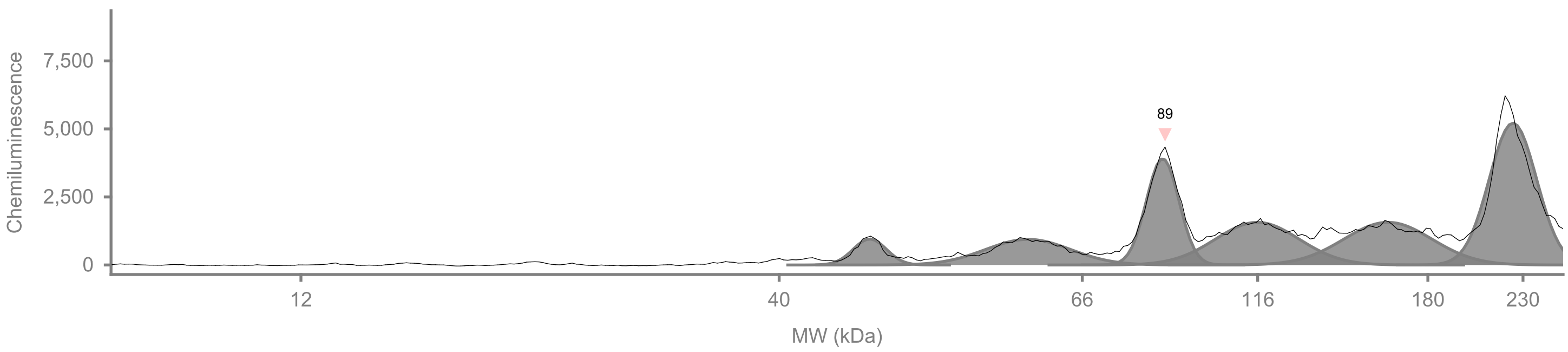

# File: P-Stat1_Jess

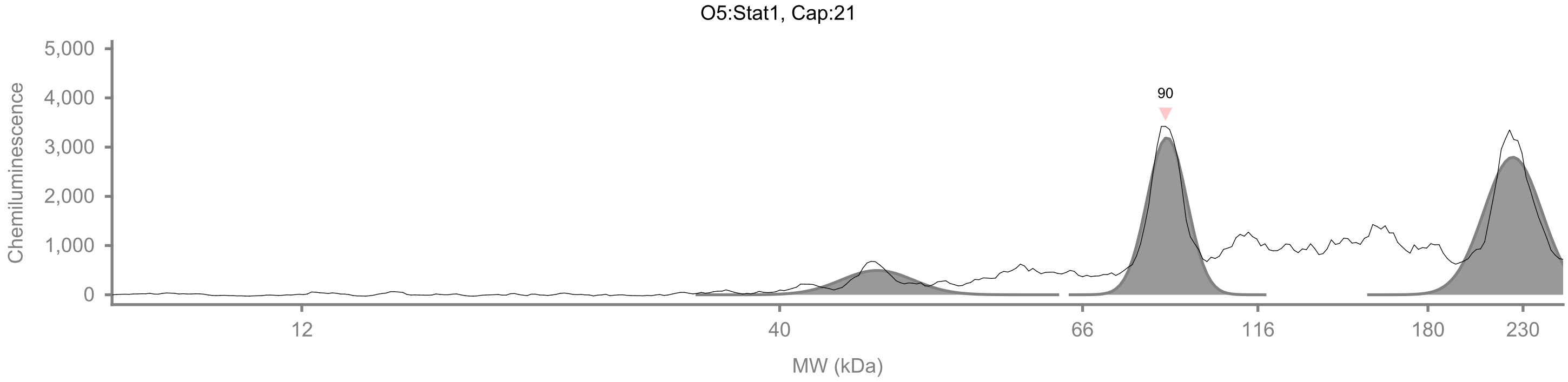


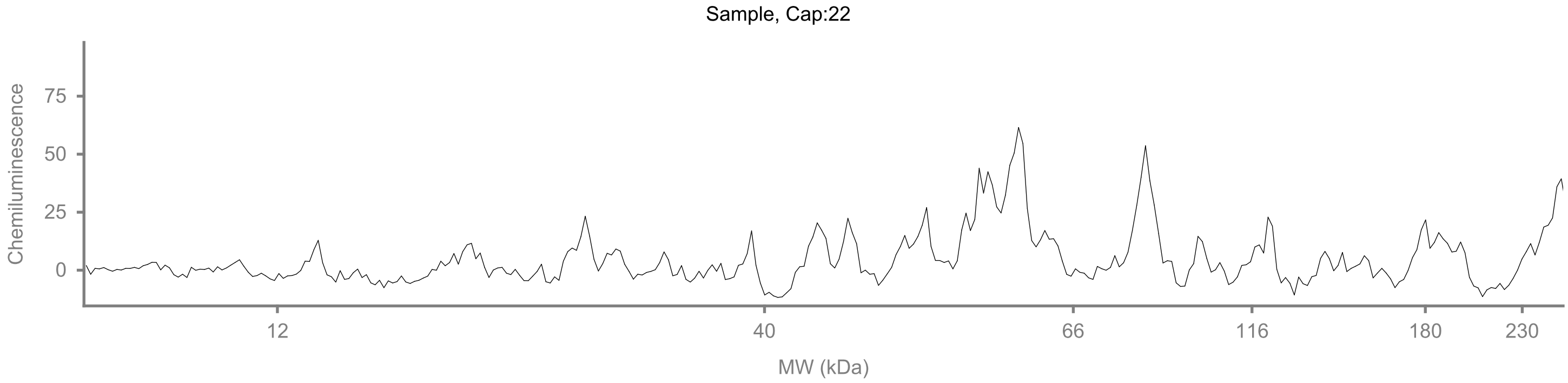


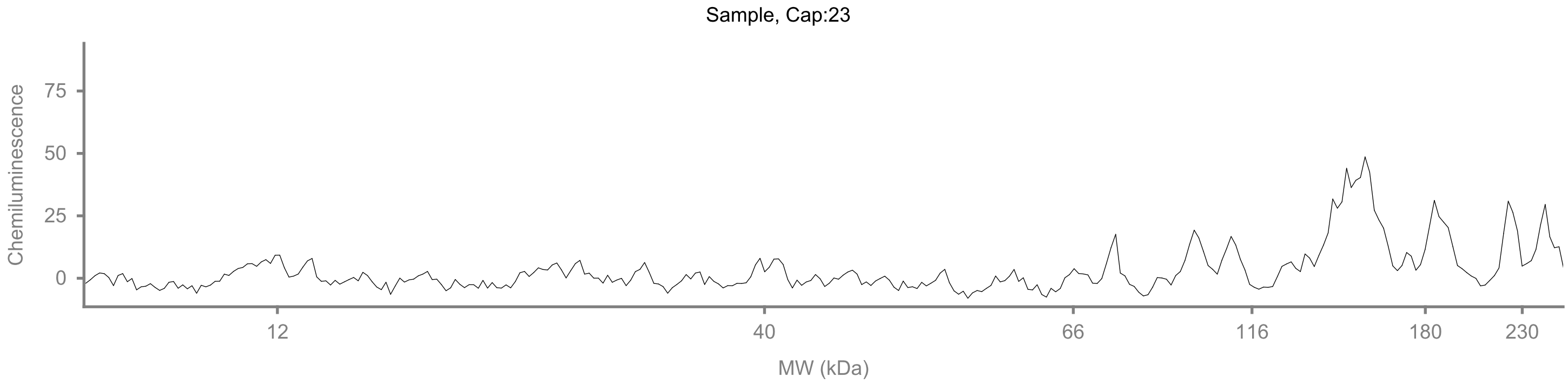


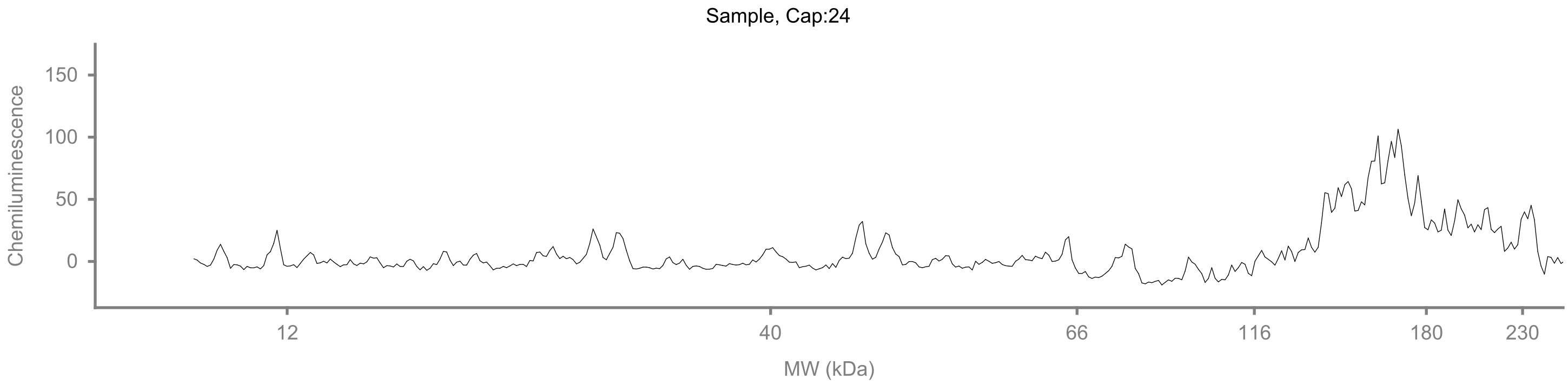




proteinsimple a biotechne brand

# File: P-Stat1_Jess

Sample, Cap:25

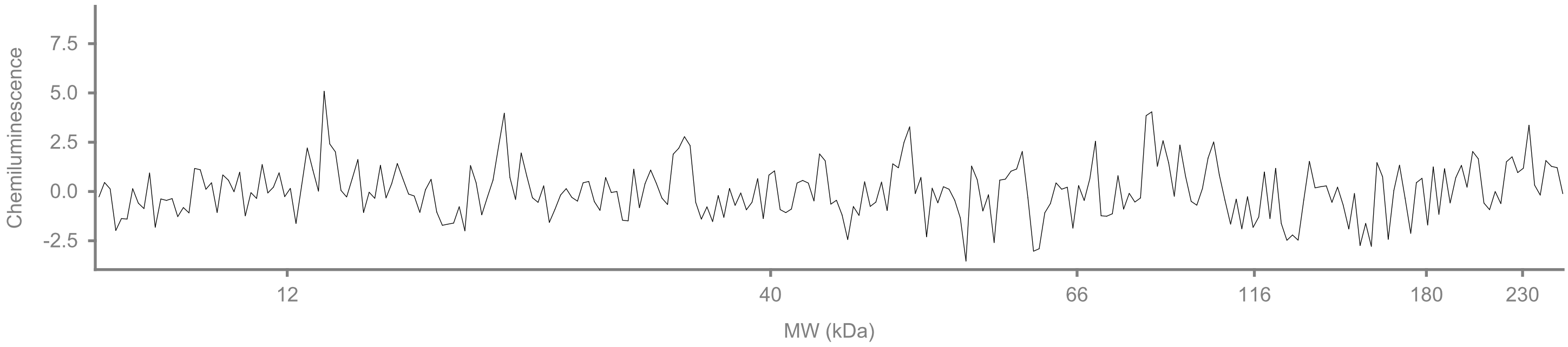




proteinsimple a biotechne brand

File: P-Stat1_Jess

## CHEMI Sample Peaks

| | | | | | | | | | | | | | |
|---|---|---|---|---|---|---|---|---|---|---|---|---|---|
| Biot. Ladder | Antibody Diluent | Streptavidin HRP | 1 | 1 | Ldr 12 | 265 | 12 | 11951.8 | 155978.1 | | 12.3 | 3544.4 | 71.3 |
| Biot. Ladder | Antibody Diluent | Streptavidin HRP | 1 | 2 | Ldr 40 | 378 | 40 | 12213.3 | 153433.9 | | 11.8 | 3622.0 | 101.7 |
| Biot. Ladder | Antibody Diluent | Streptavidin HRP | 1 | 3 | Ldr 66 | 450 | 66 | 2883.6 | 55711.4 | | 18.2 | 855.2 | 109.8 |
| Biot. Ladder | Antibody Diluent | Streptavidin HRP | 1 | 4 | Ldr 116 | 491 | 116 | 9344.5 | 102620.7 | | 10.3 | 2771.2 | 111.3 |
| Biot. Ladder | Antibody Diluent | Streptavidin HRP | 1 | 5 | Ldr 180 | 531 | 180 | 3851.2 | 46156.7 | | 11.3 | 1142.1 | 110.9 |
| Biot. Ladder | Antibody Diluent | Streptavidin HRP | 1 | 6 | Ldr 230 | 553 | 230 | 5176.8 | 72940.4 | | 13.2 | 1535.2 | 110.1 |
| Y1:Actin | Actin | Secondary | 2 | 1 | | 374 | 40 | 13489.5 | 90549.1 | | 6.3 | 5600.2 | 15.1 |
| Y2:Actin | Actin | Secondary | 3 | 1 | | 374 | 41 | 12992.5 | 87915.2 | | 6.4 | 5138.3 | 14.2 |
| Y3:Actin | Actin | Secondary | 4 | 1 | | 373 | 41 | 16735.0 | 108326.4 | | 6.1 | 6501.4 | 16.2 |
| Y4:Actin | Actin | Secondary | 5 | 1 | | 372 | 41 | 12089.5 | 79359.0 | | 6.2 | 5319.5 | 15.0 |
| Y5:Actin | Actin | Secondary | 6 | 1 | | 373 | 41 | 7746.9 | 53123.4 | | 6.4 | 3625.4 | 16.4 |
| O1:Actin | Actin | Secondary | 7 | 2 | | 373 | 41 | 7057.9 | 47941.1 | | 6.4 | 3539.6 | 19.0 |
| O2:Actin | Actin | Secondary | 8 | 1 | | 371 | 41 | 10832.8 | 68891.1 | | 6.0 | 5295.9 | 18.0 |
| O3:Actin | Actin | Secondary | 9 | 1 | | 372 | 41 | 11589.0 | 75433.8 | | 6.1 | 4862.9 | 19.6 |
| O4:Actin | Actin | Secondary | 10 | 1 | | 372 | 41 | 15768.3 | 104678.2 | | 6.2 | 6613.4 | 19.7 |
| O5:Actin | Actin | Secondary | 11 | 1 | | 372 | 41 | 9598.5 | 62777.6 | | 6.1 | 4474.1 | 19.5 |
| Y1:Stat1 | P-Stat1 | Secondary | 12 | 2 | | 464 | 88 | 2637.4 | 20425.5 | | 7.3 | 195.8 | 143.4 |
| Y2:Stat1 | P-Stat1 | Secondary | 13 | 2 | | 462 | 89 | 2277.9 | 22066.6 | | 9.1 | 163.7 | 128.6 |
| Y3:Stat1 | P-Stat1 | Secondary | 14 | 3 | | 464 | 90 | 2191.2 | 18535.5 | | 7.9 | 143.9 | 157.7 |
| Y4:Stat1 | P-Stat1 | Secondary | 15 | 3 | | 464 | 89 | 3300.5 | 30632.9 | | 8.7 | 180.6 | 182.3 |
| Y5:Stat1 | P-Stat1 | Secondary | 16 | 1 | | 464 | 90 | 2436.6 | 34021.7 | | 13.1 | 178.7 | 202.8 |
| O1:Stat1 | P-Stat1 | Secondary | 17 | 2 | | 463 | 90 | 2855.0 | 46770.0 | | 15.4 | 219.8 | 137.9 |
| O2:Stat1 | P-Stat1 | Secondary | 18 | 1 | | 465 | 91 | 3053.8 | 33409.6 | | 10.3 | 291.0 | 120.7 |
| O3:Stat1 | P-Stat1 | Secondary | 19 | 3 | | 466 | 90 | 3701.7 | 32961.4 | | 8.4 | 261.4 | 163.6 |
| O4:Stat1 | P-Stat1 | Secondary | 20 | 3 | | 466 | 89 | 3910.8 | 38435.1 | | 9.2 | 197.5 | 161.7 |
| O5:Stat1 | P-Stat1 | Secondary | 21 | 2 | | 465 | 90 | 3193.9 | 38396.2 | | 11.3 | 239.7 | 123.5 |

File: P-Stat1_Jess

# Standard Plots

C1:1

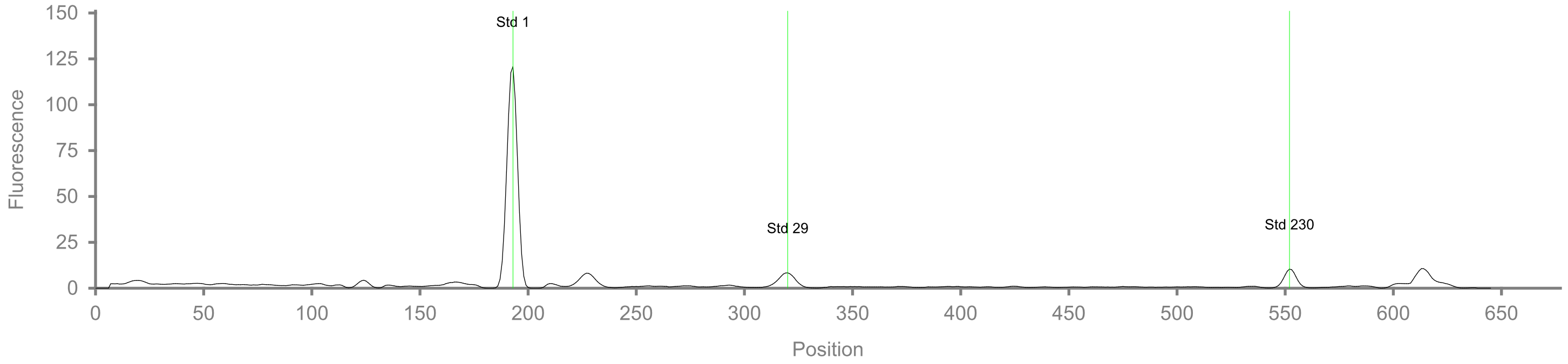


C1:2

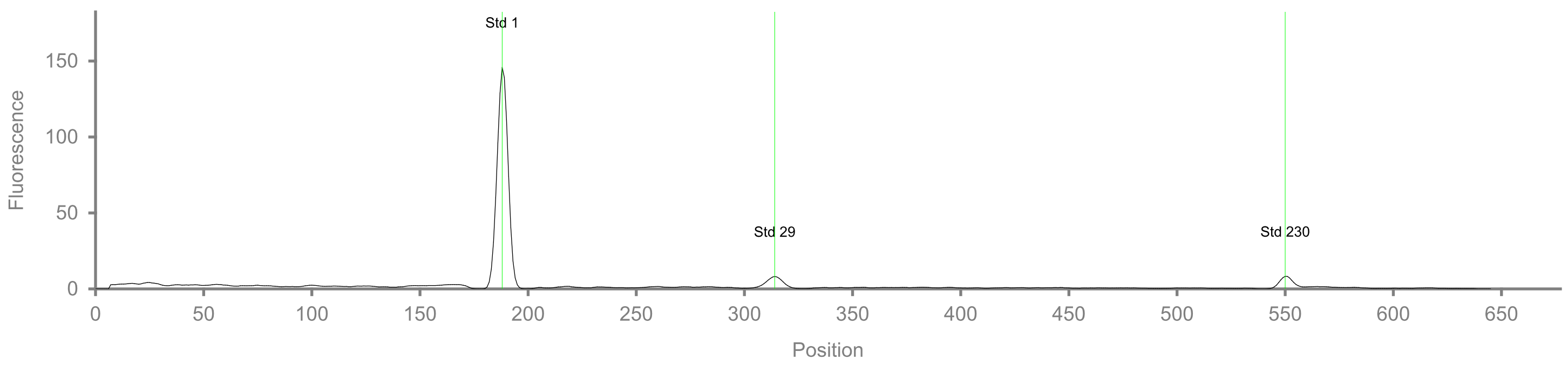


C1:3

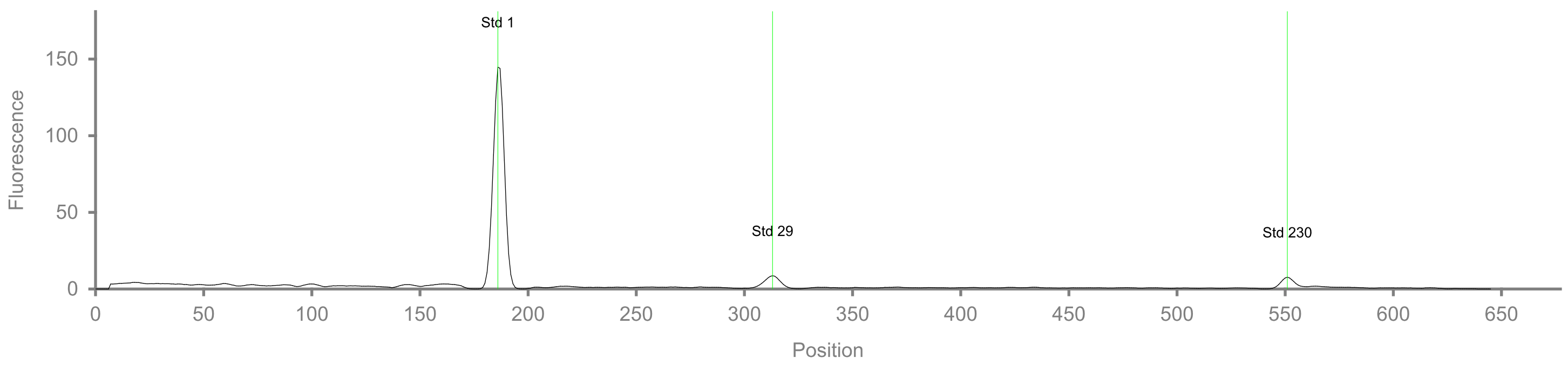


C1:4

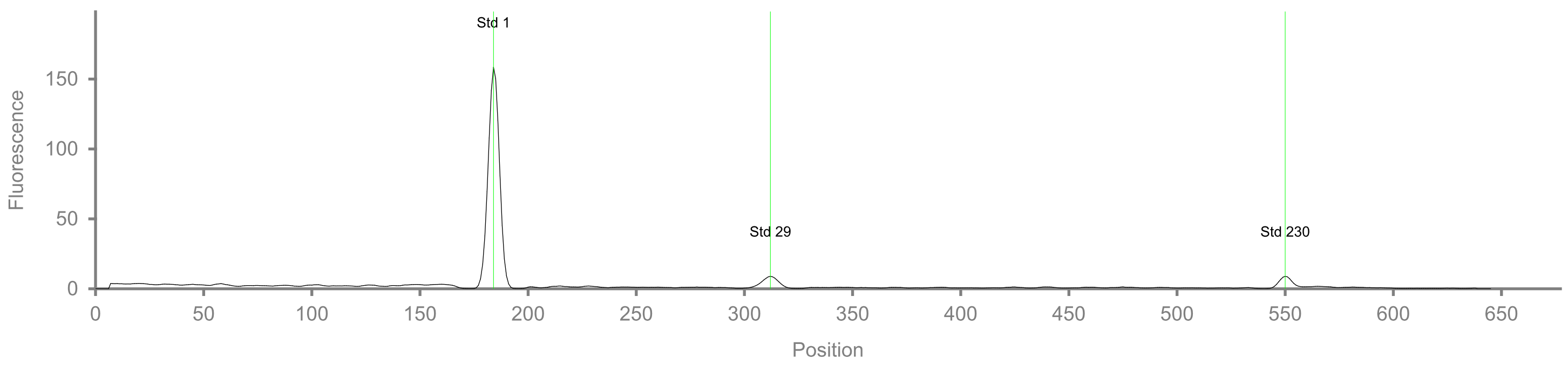

# File: P-Stat1_Jess

C1:5

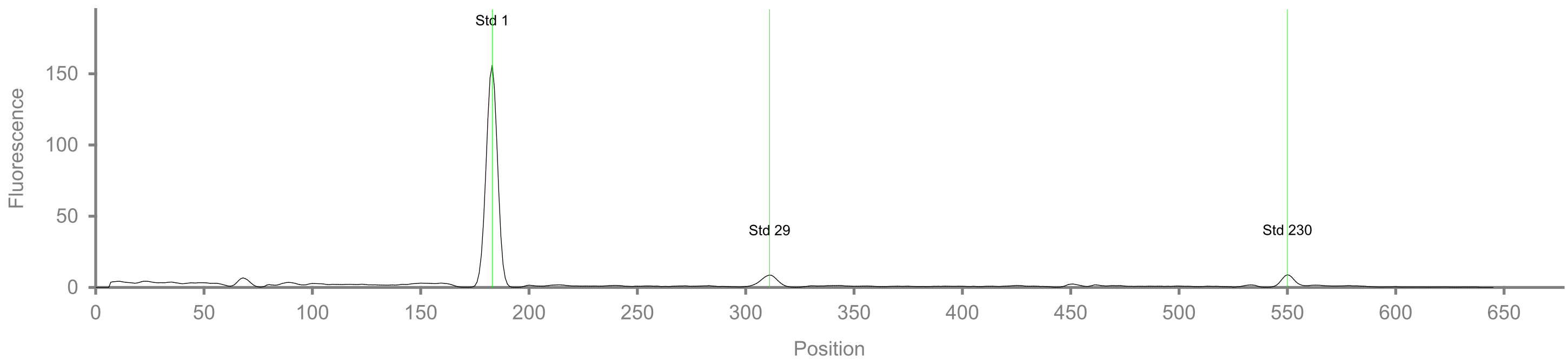


C1:6

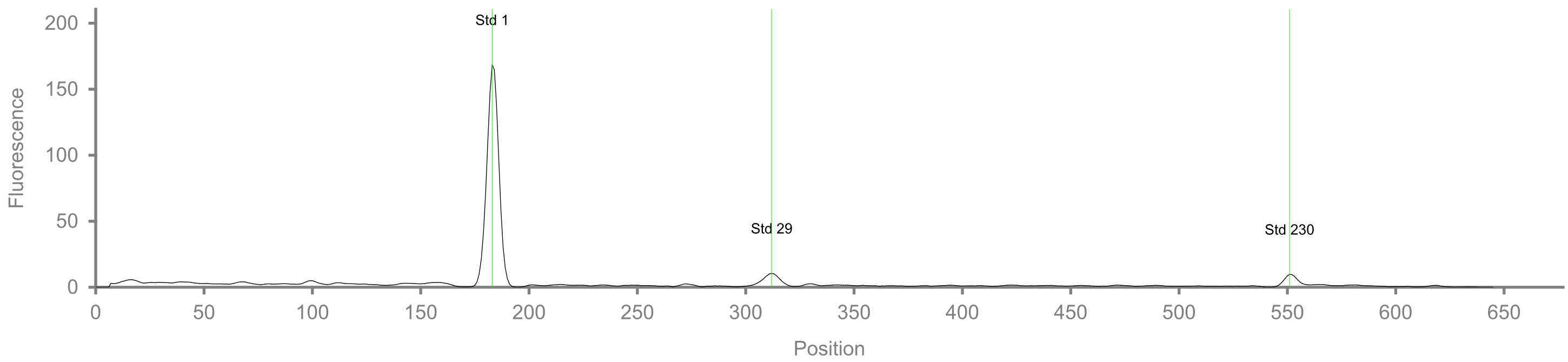


C1:7

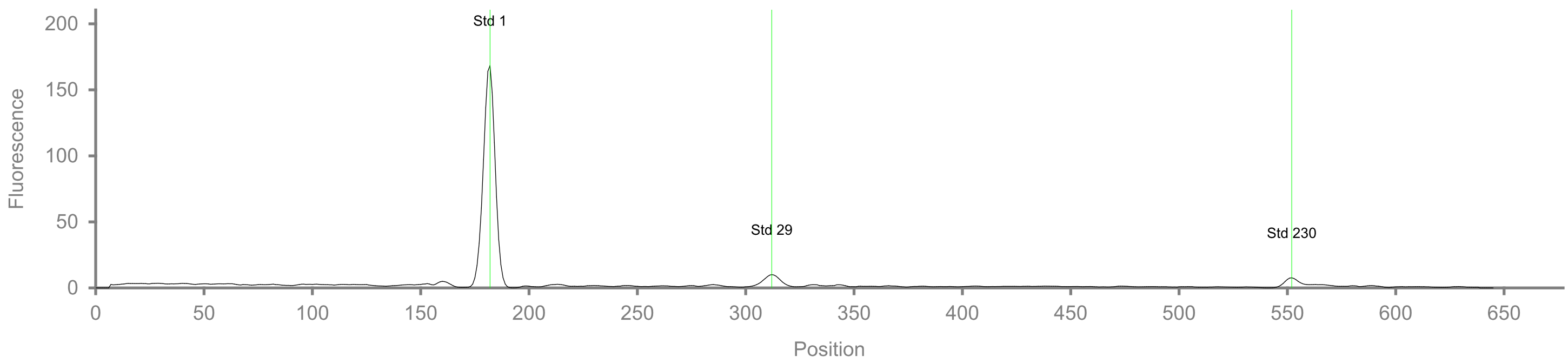


C1:8

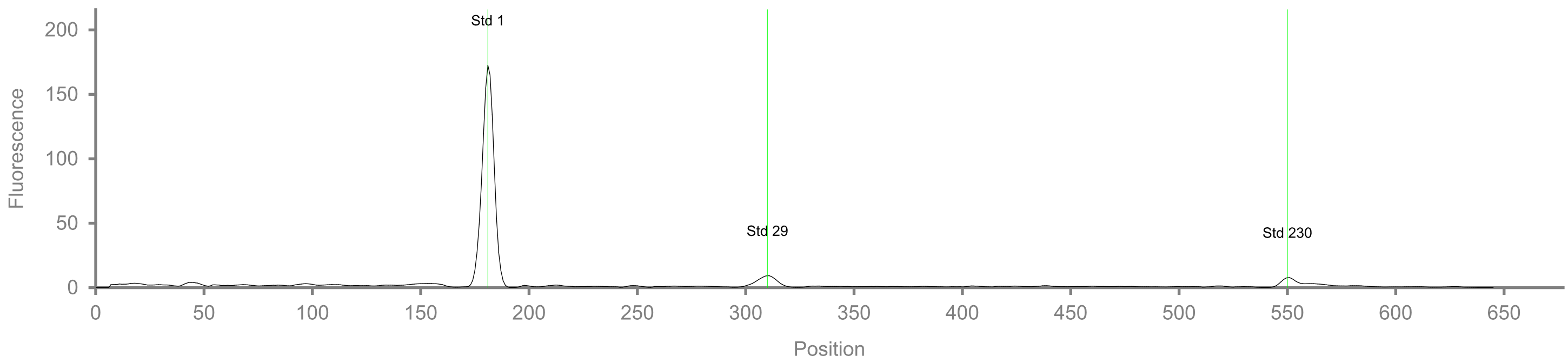

# File: P-Stat1_Jess

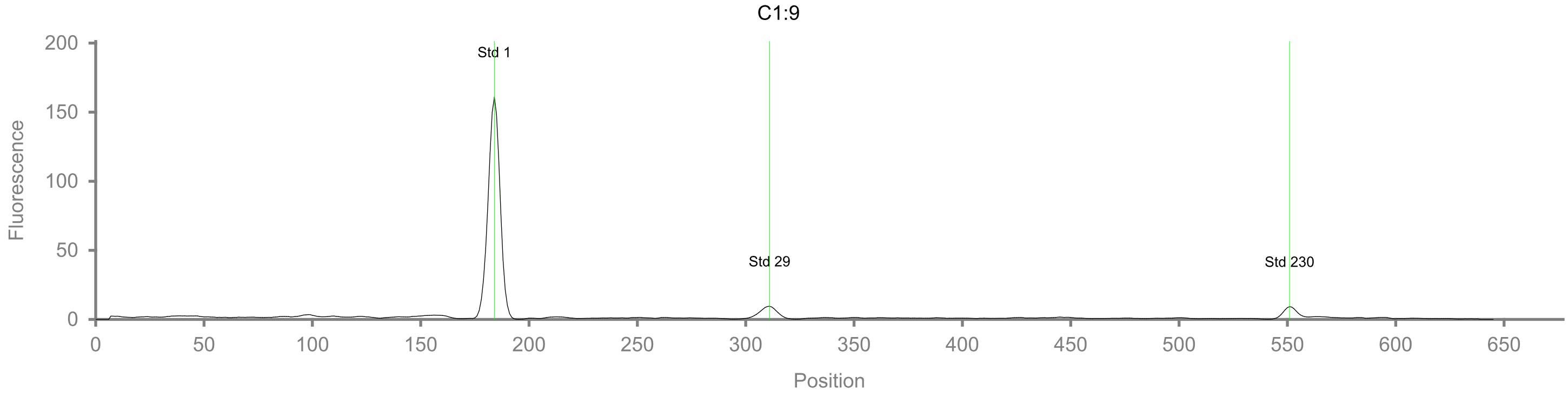


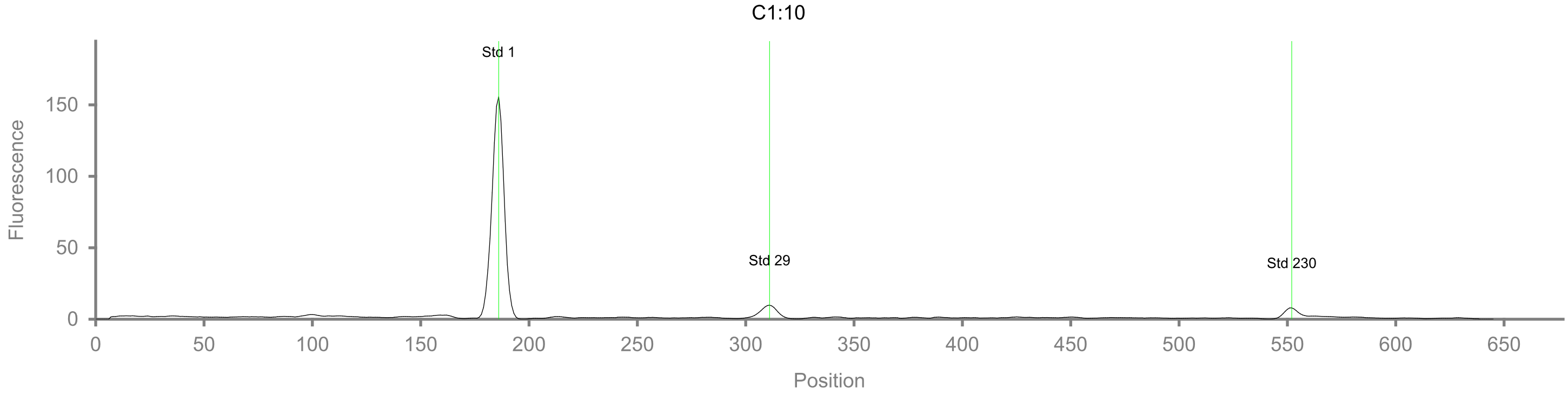


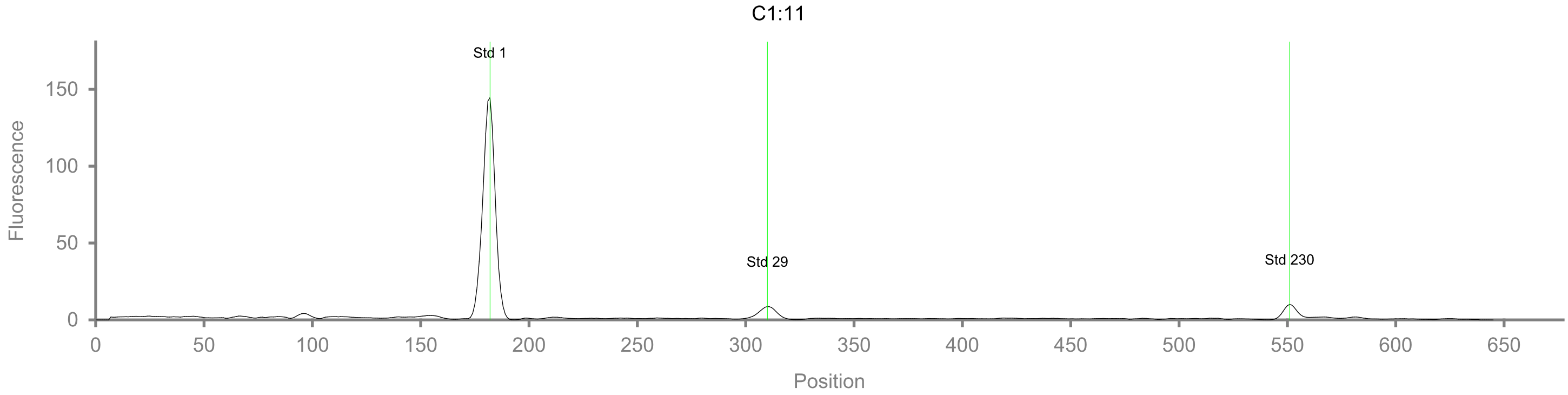


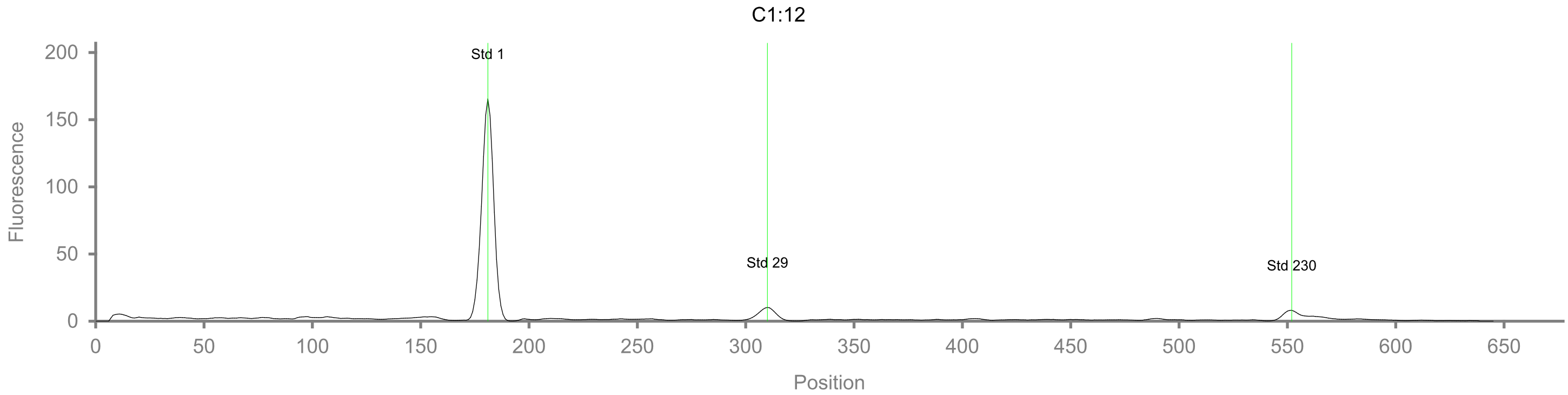

# File: P-Stat1_Jess

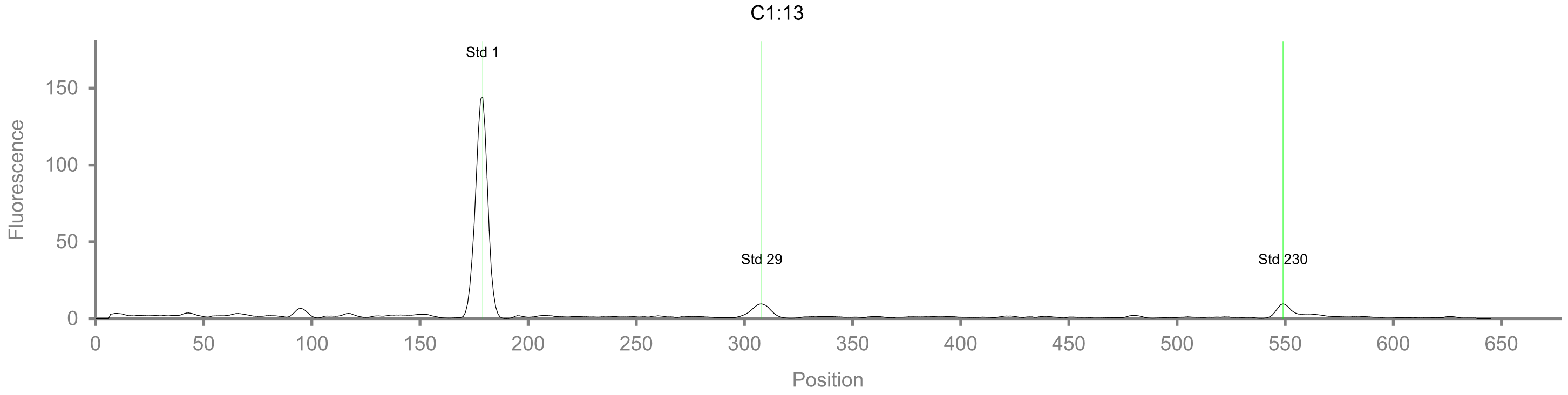


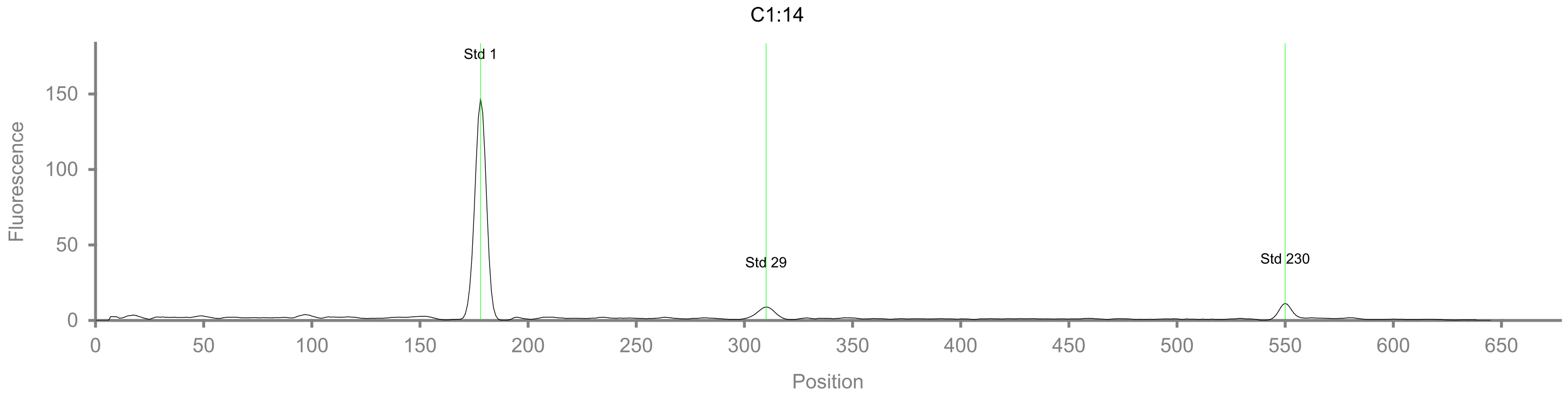


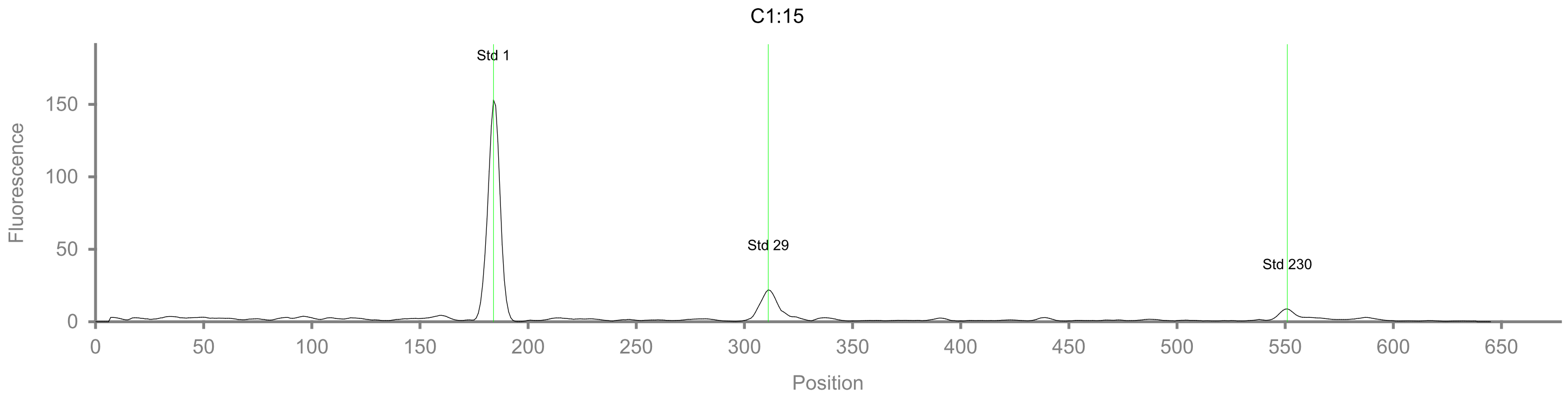


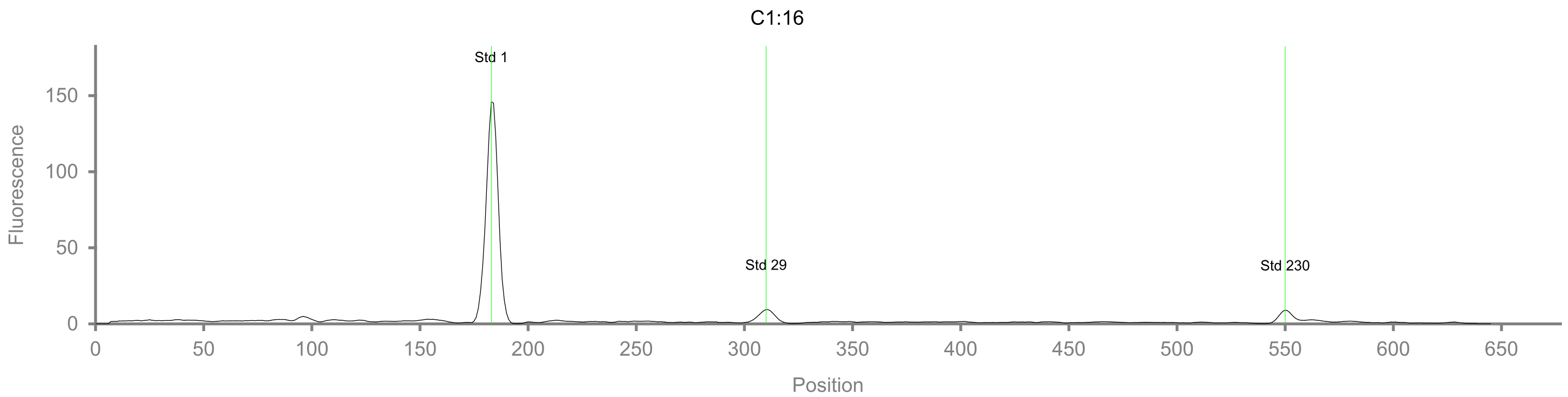

# File: P-Stat1_Jess

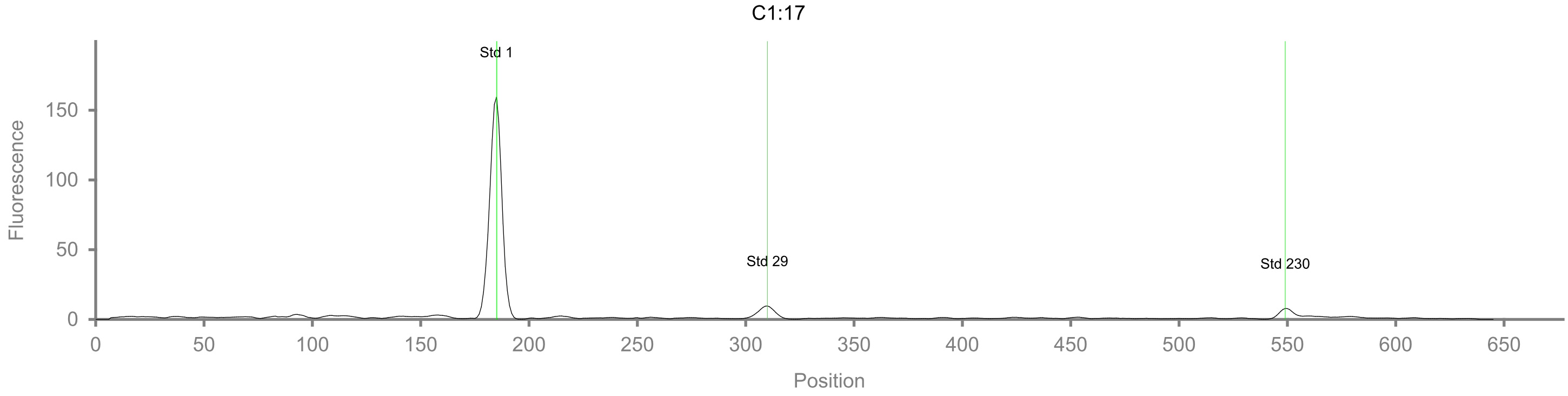


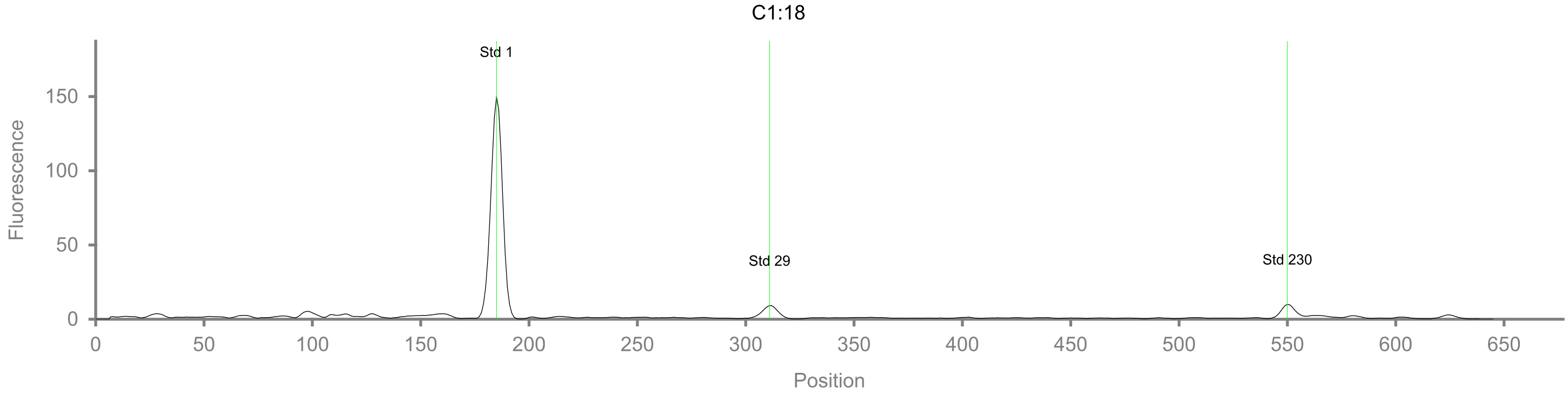


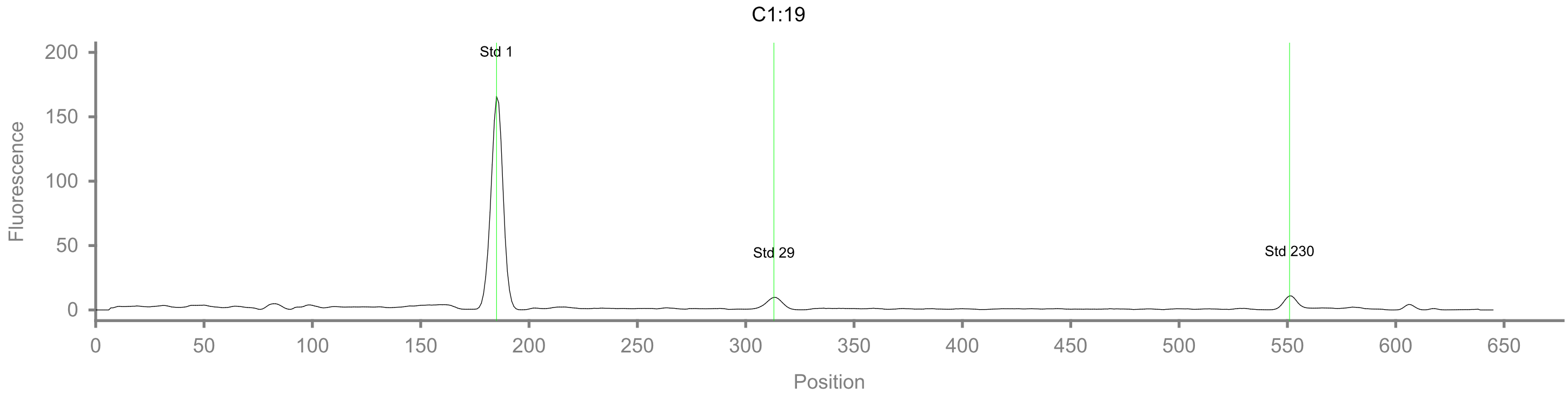


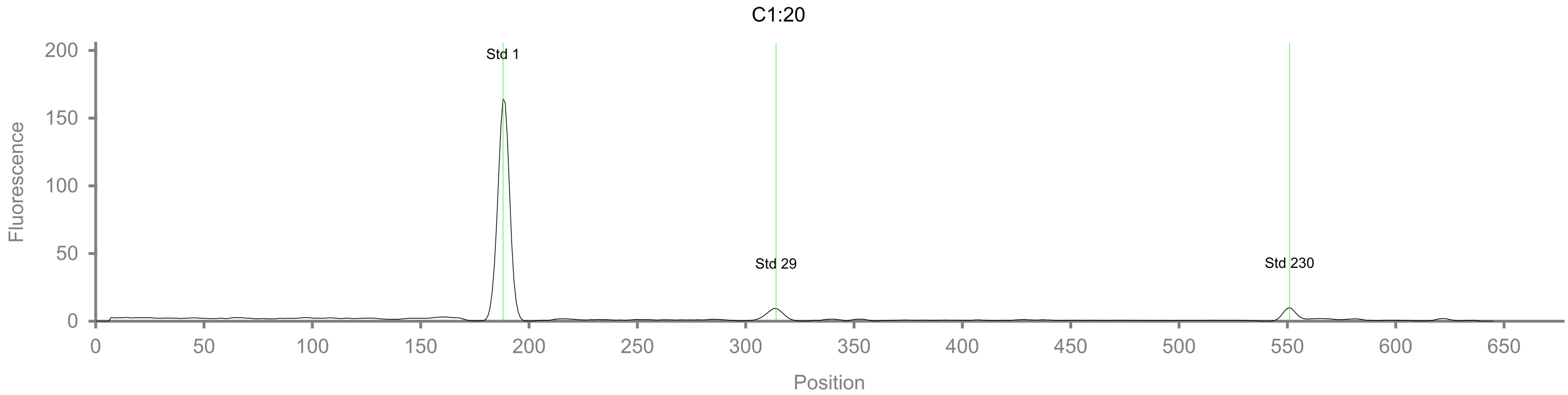


C:\Users\\OneDrive\Desktop\P-Stat1_Jess.cbz

# File: P-Stat1_Jess

C1:21

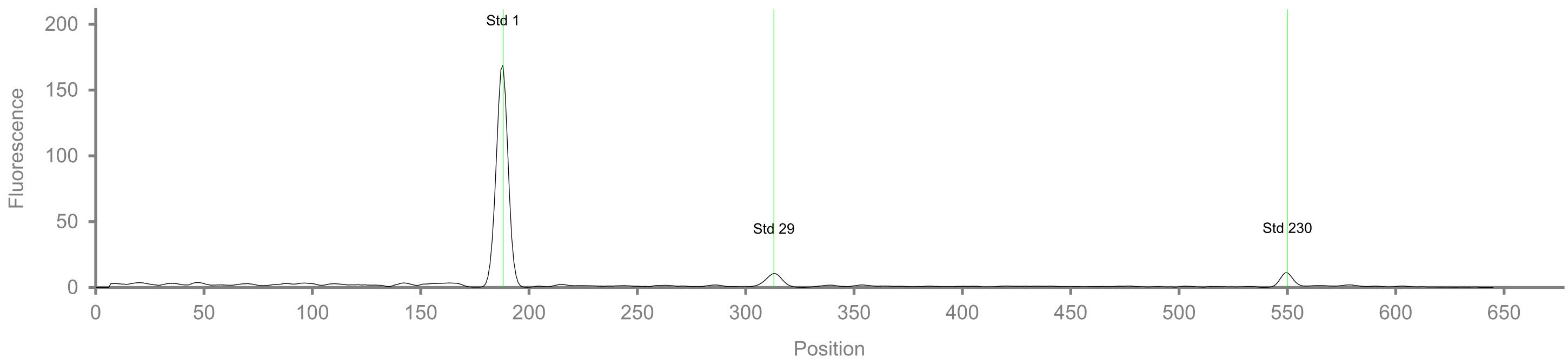


C1:22

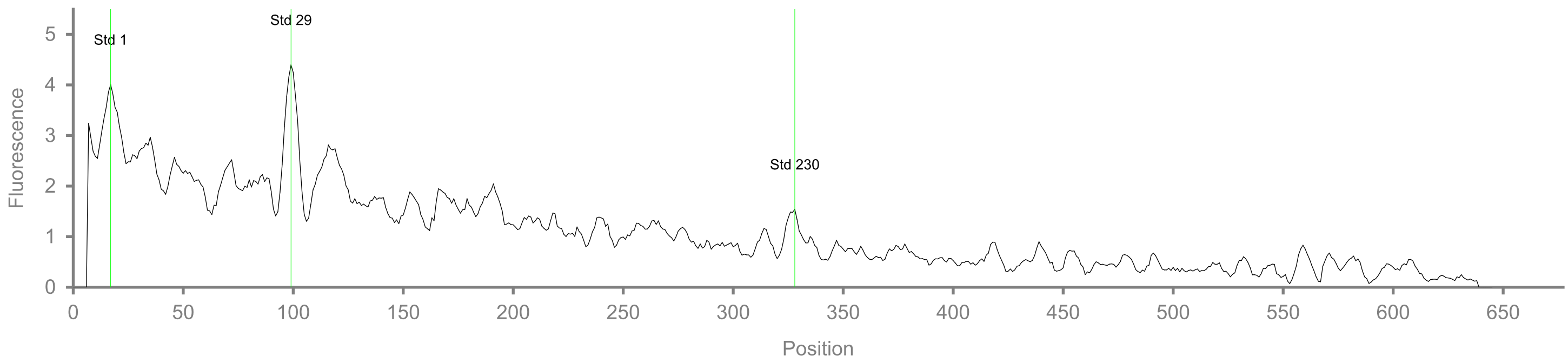


C1:23

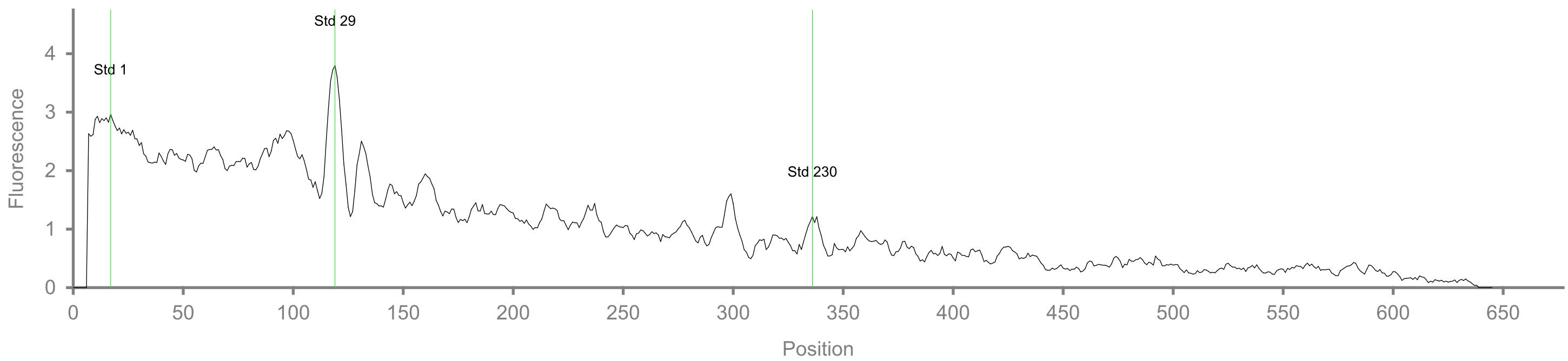


C1:24

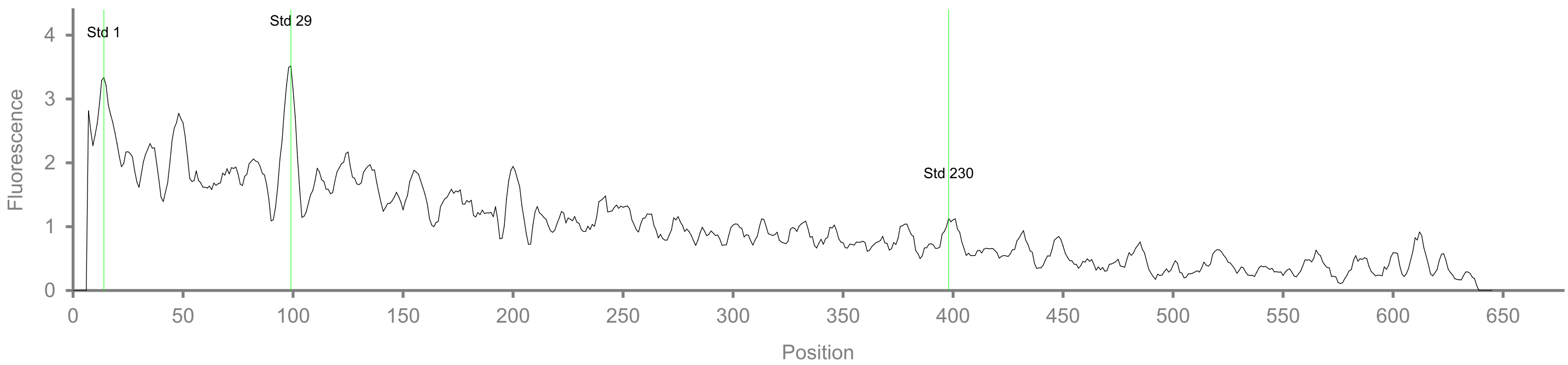


C:\Users\\OneDrive\Desktop\P-Stat1_Jess.cbz

# File: P-Stat1_Jess

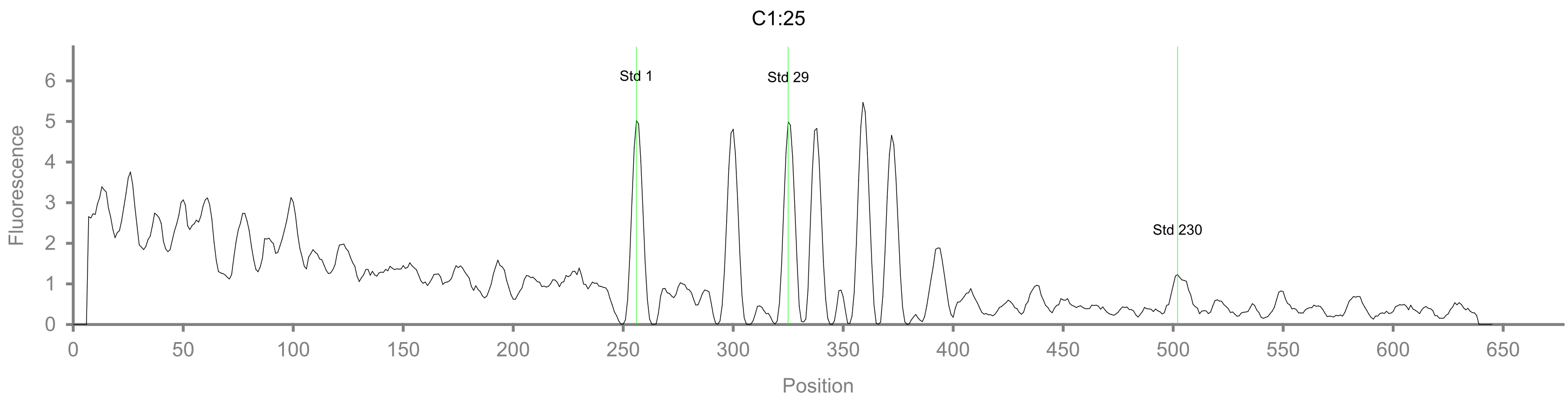

File: P-Stat1_Jess

## Standard Peaks (pixels)

| | | | | Std 1 | Std 29 | Std 230 |
|---|---|---|---|---|---|---|
| Biot. Ladder | Antibody Diluent | Streptavidin HRP | C1:1 | 192.8 | 319.9 | 552.0 |
| Y1:Actin | Actin | Secondary | C1:2 | 188.1 | 314.0 | 550.2 |
| Y2:Actin | Actin | Secondary | C1:3 | 186.2 | 312.9 | 551.2 |
| Y3:Actin | Actin | Secondary | C1:4 | 184.0 | 311.9 | 550.2 |
| Y4:Actin | Actin | Secondary | C1:5 | 182.8 | 310.9 | 550.1 |
| Y5:Actin | Actin | Secondary | C1:6 | 183.0 | 311.9 | 551.2 |
| O1:Actin | Actin | Secondary | C1:7 | 181.6 | 312.0 | 552.2 |
| O2:Actin | Actin | Secondary | C1:8 | 180.8 | 309.9 | 550.3 |
| O3:Actin | Actin | Secondary | C1:9 | 183.6 | 310.9 | 551.2 |
| O4:Actin | Actin | Secondary | C1:10 | 185.5 | 310.9 | 552.2 |
| O5:Actin | Actin | Secondary | C1:11 | 181.4 | 309.9 | 551.2 |
| Y1:Stat1 | P-Stat1 | Secondary | C1:12 | 180.5 | 309.9 | 552.2 |
| Y2:Stat1 | P-Stat1 | Secondary | C1:13 | 178.3 | 307.9 | 549.2 |
| Y3:Stat1 | P-Stat1 | Secondary | C1:14 | 177.6 | 310.0 | 550.1 |
| Y4:Stat1 | P-Stat1 | Secondary | C1:15 | 183.7 | 311.2 | 551.1 |
| Y5:Stat1 | P-Stat1 | Secondary | C1:16 | 182.8 | 309.9 | 550.2 |
| O1:Stat1 | P-Stat1 | Secondary | C1:17 | 184.5 | 309.9 | 549.2 |
| O2:Stat1 | P-Stat1 | Secondary | C1:18 | 184.8 | 311.0 | 550.2 |
| O3:Stat1 | P-Stat1 | Secondary | C1:19 | 184.9 | 312.9 | 551.2 |
| O4:Stat1 | P-Stat1 | Secondary | C1:20 | 188.0 | 313.9 | 551.2 |
| O5:Stat1 | P-Stat1 | Secondary | C1:21 | 187.7 | 312.9 | 550.1 |
| Sample | Primary | Secondary | C1:22 | 16.9 | 99.0 | 328.0 |
| Sample | Primary | Secondary | C1:23 | 17.0 | 119.0 | 336.0 |
| Sample | Primary | Secondary | C1:24 | 14.0 | 99.0 | 398.0 |
| Sample | Primary | Secondary | C1:25 | 256.0 | 325.4 | 502.0 |

File: P-Stat1_Jess

## Sample Images

CHEMI, 1s, Sample

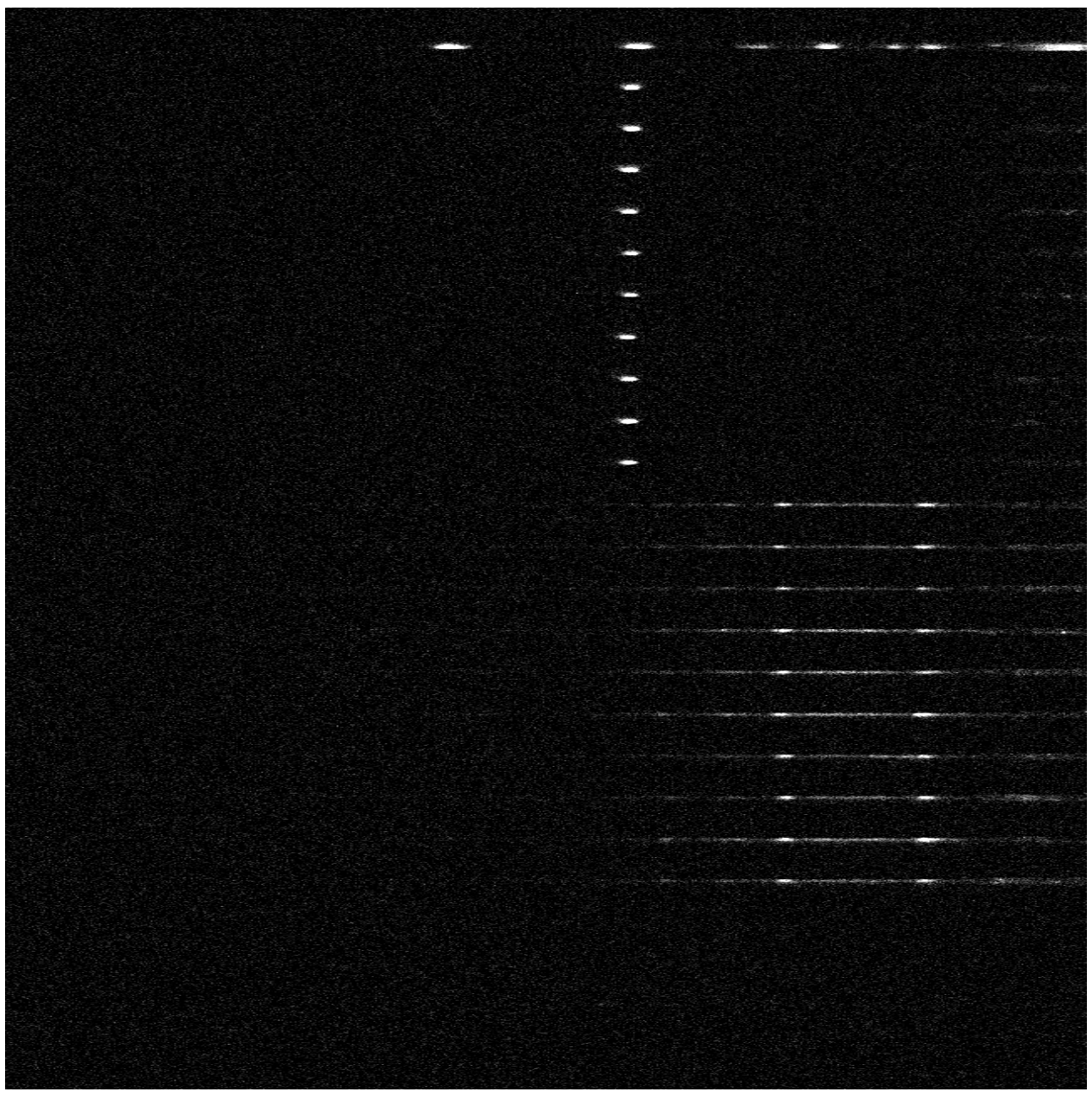

File: P-Stat1_Jess

## Standard Image

Std, 4s

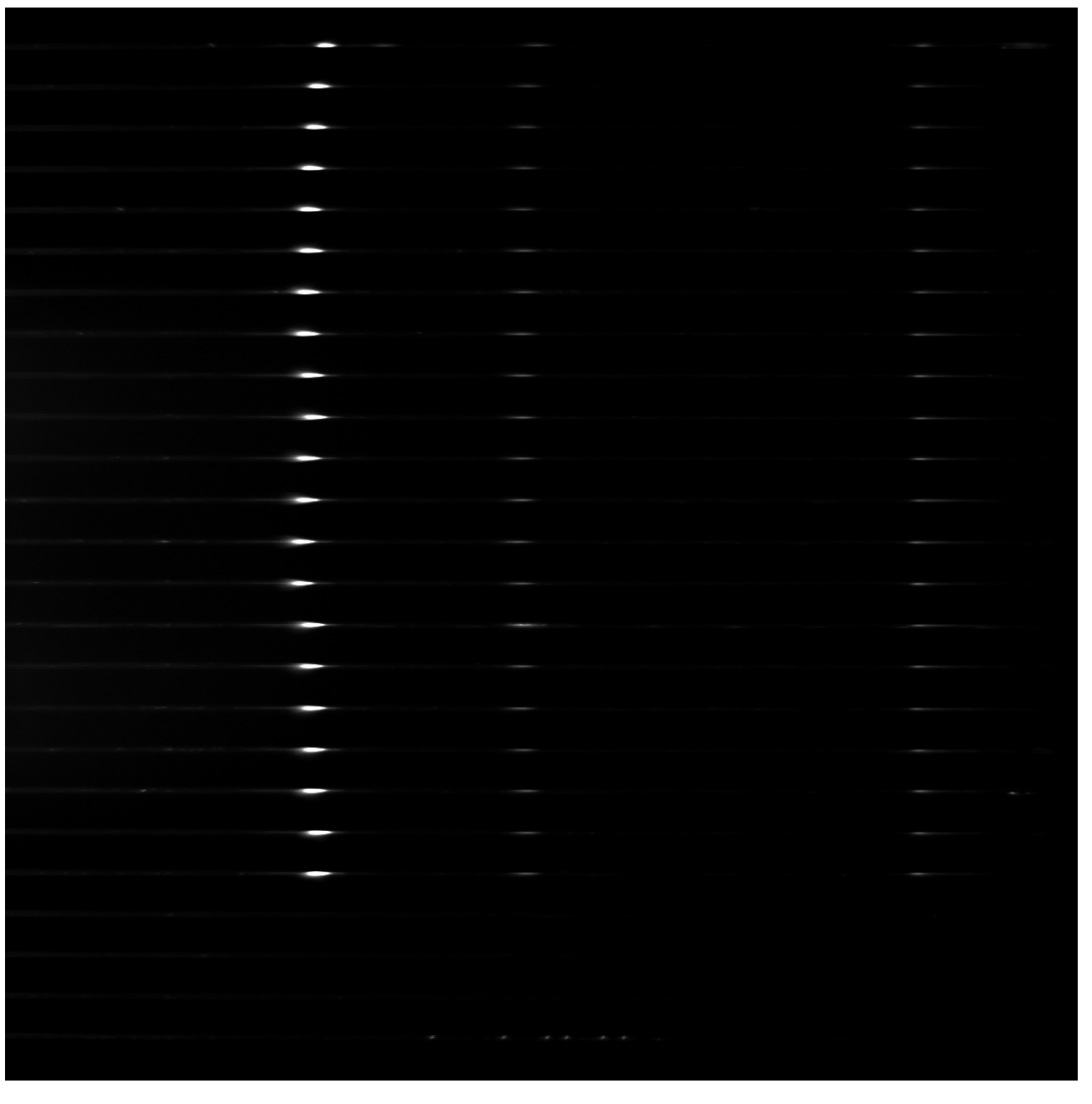

File: P-Stat1_Jess

# Analysis Log

| | | | |
|---|---|---|---|
| 2026-07-14 19:41:26 | | Started run: 2026-07-14_19-45-56_Jess Assay: Jess.assay from Compass for SW v6.3.0-0719 | |
| 2026-07-15 09:42:15 | | Saved template changes from Compass for SW v6.3.0-0719 | |
| | | Changed well annotation at row A column 2: Name from Sample to Y1:Actin | |
| | | Changed well annotation at row A column 3: Name from Sample to Y2:Actin | |
| | | Changed well annotation at row A column 4: Name from Sample to Y3:Actin | |
| | | Changed well annotation at row A column 5: Name from Sample to Y4:Actin | |
| | | Changed well annotation at row A column 6: Name from Sample to Y5:Actin | |
| | | Changed well annotation at row A column 7: Name from Sample to O1:Actin | |
| | | Changed well annotation at row A column 8: Name from Sample to O2:Actin | |
| | | Changed well annotation at row A column 9: Name from Sample to O3:Actin | |
| | | Changed well annotation at row A column 10: Name from Sample to O4:Actin | |
| | | Changed well annotation at row A column 11: Name from Sample to O5:Actin | |
| | | Changed well annotation at row A column 12: Name from Sample to Y1:Stat1 | |
| | | Changed well annotation at row A column 13: Name from Sample to Y2:Stat1 | |
| | | Changed well annotation at row A column 14: Name from Sample to Y3:Stat1 | |
| | | Changed well annotation at row A column 15: Name from Sample to Y4:Stat1 | |
| | | Changed well annotation at row A column 16: Name from Sample to Y5:Stat1 | |
| | | Changed well annotation at row A column 17: Name from Sample to O1:Stat1 | |
| | | Changed well annotation at row A column 18: Name from Sample to O2:Stat1 | |
| | | Changed well annotation at row A column 19: Name from Sample to O3:Stat1 | |
| | | Changed well annotation at row A column 20: Name from Sample to O4:Stat1 | |
| | | Changed well annotation at row A column 21: Name from Sample to O5:Stat1 | |
| | | Changed well annotation at row C column 2: Name from Primary to Actin | |

File: P-Stat1_Jess

| | | | |
|---|---|---|---|
| | | Changed well annotation at row C column 3: Name from Primary to Actin | |
| | | Changed well annotation at row C column 4: Name from Primary to Actin | |
| | | Changed well annotation at row C column 5: Name from Primary to Actin | |
| | | Changed well annotation at row C column 6: Name from Primary to Actin | |
| | | Changed well annotation at row C column 7: Name from Primary to Actin | |
| | | Changed well annotation at row C column 8: Name from Primary to Actin | |
| | | Changed well annotation at row C column 9: Name from Primary to Actin | |
| | | Changed well annotation at row C column 10: Name from Primary to Actin | |
| | | Changed well annotation at row C column 11: Name from Primary to Actin | |
| | | Changed well annotation at row C column 12: Name from Primary to P-Stat1 | |
| | | Changed well annotation at row C column 13: Name from Primary to P-Stat1 | |
| | | Changed well annotation at row C column 14: Name from Primary to P-Stat1 | |
| | | Changed well annotation at row C column 15: Name from Primary to P-Stat1 | |
| | | Changed well annotation at row C column 16: Name from Primary to P-Stat1 | |
| | | Changed well annotation at row C column 17: Name from Primary to P-Stat1 | |
| | | Changed well annotation at row C column 18: Name from Primary to P-Stat1 | |
| | | Changed well annotation at row C column 19: Name from Primary to P-Stat1 | |
| | | Changed well annotation at row C column 20: Name from Primary to P-Stat1 | |
| | | Changed well annotation at row C column 21: Name from Primary to P-Stat1 | |
| 2026-07-15 09:43:35 | | Saved analysis and template changes from Compass for SW v6.3.0-0719 | |
| | | Added Peak Fit Apply Override "apply fit to Biot. Ladder" | |
| 2026-07-15 09:52:21 | | Saved analysis changes from Compass for SW v6.3.0-0719 | |
| | | Hide peak at position 555 in manual settings for capillary 2 CHEMI | |
| | | Hide peak at position 534 in manual settings for capillary 5 CHEMI | |
| | | Hide peak at position 472 in manual settings for capillary 6 CHEMI | |
| | | Hide peak at position 552 in manual settings for capillary 6 CHEMI | |
| | | Hide peak at position 330 in manual settings for capillary 7 CHEMI | |
| | | Hide peak at position 475 in manual settings for capillary 8 CHEMI | |
| | | Hide peak at position 513 in manual settings for capillary 8 CHEMI | |
| | | Hide peak at position 561 in manual settings for capillary 10 CHEMI | |
| | | Hide peak at position 442 in manual settings for capillary 11 CHEMI | |

# File: P-Stat1_Jess

| | | | |
|---|---|---|---|
| | | Hide peak at position 501 in manual settings for capillary 11 CHEMI | |
| | | Hide peak at position 432 in manual settings for capillary 12 CHEMI | |
| | | Hide peak at position 486 in manual settings for capillary 12 CHEMI | |
| | | Hide peak at position 550 in manual settings for capillary 12 CHEMI | |
| | | Hide peak at position 391 in manual settings for capillary 13 CHEMI | |
| | | Hide peak at position 521 in manual settings for capillary 13 CHEMI | |
| | | Hide peak at position 548 in manual settings for capillary 13 CHEMI | |
| | | Hide peak at position 393 in manual settings for capillary 14 CHEMI | |
| | | Hide peak at position 431 in manual settings for capillary 14 CHEMI | |
| | | Hide peak at position 488 in manual settings for capillary 14 CHEMI | |
| | | Hide peak at position 549 in manual settings for capillary 14 CHEMI | |
| | | Hide peak at position 394 in manual settings for capillary 15 CHEMI | |
| | | Hide peak at position 429 in manual settings for capillary 15 CHEMI | |
| | | Hide peak at position 489 in manual settings for capillary 15 CHEMI | |
| | | Hide peak at position 518 in manual settings for capillary 15 CHEMI | |
| | | Hide peak at position 549 in manual settings for capillary 15 CHEMI | |
| | | Hide peak at position 550 in manual settings for capillary 16 CHEMI | |
| | | Hide peak at position 428 in manual settings for capillary 17 CHEMI | |
| | | Hide peak at position 548 in manual settings for capillary 17 CHEMI | |
| | | Hide peak at position 521 in manual settings for capillary 18 CHEMI | |
| | | Hide peak at position 549 in manual settings for capillary 18 CHEMI | |
| | | Hide peak at position 395 in manual settings for capillary 19 CHEMI | |
| | | Hide peak at position 435 in manual settings for capillary 19 CHEMI | |
| | | Hide peak at position 486 in manual settings for capillary 19 CHEMI | |
| | | Hide peak at position 517 in manual settings for capillary 19 CHEMI | |
| | | Hide peak at position 550 in manual settings for capillary 19 CHEMI | |
| | | Hide peak at position 394 in manual settings for capillary 20 CHEMI | |
| | | Hide peak at position 431 in manual settings for capillary 20 CHEMI | |
| | | Hide peak at position 487 in manual settings for capillary 20 CHEMI | |
| | | Hide peak at position 518 in manual settings for capillary 20 CHEMI | |
| | | Hide peak at position 549 in manual settings for capillary 20 CHEMI | |
| | | Hide peak at position 394 in manual settings for capillary 21 CHEMI | |
| | | Hide peak at position 549 in manual settings for capillary 21 CHEMI | |

File: Stat1_Jess

# Run

| | |
|---|---|
| | 2026-07-14_15-57-16_Jess |
| | C:\Users\\OneDrive\Desktop |
| | Jess |
| | Regular: 12-230 kDa |
| | |
| | Jess : Jess JS5549 - js5549 |
| | 2.1.2021.09.01.00.26.14.bc9a11c63 |
| | |
| | 1144502075 |
| | 2026067630401030 |
| | 6-2026 |
| | 3:52 7 14, 2026 CST |
| | 6:37 7 14, 2026 CST |
| | None |

File: Stat1_Jess

# Protocol

| | |
|---|---|
| Separation Matrix | |
| Well Row | |
| Load Time (sec) | |
| Stacking Matrix | |
| Well Row | |
| Load Time (sec) | |
| Sample | |
| Well Row | |
| Load Time (sec) | |
| Separation Time (min) | |
| Separation Voltage (volts) | |
| Standards Exposure (sec) | |
| EE Immobilization Time (sec) | |
| Antibody Diluent Time (min) | |
| Well Row | |
| Primary Antibody Time (min) | |
| Well Row | |
| Secondary Antibody Time (min) | |
| Well Row | |
| Detection | |
| Well Row | |
| CHEMI Detection Profile | |
| Exposure 1 (sec) | |
| Exposure 2 (sec) | |
| Exposure 3 (sec) | |
| Exposure 4 (sec) | |
| Exposure 5 (sec) | |
| Exposure 6 (sec) | |
| Exposure 7 (sec) | |
| Exposure 8 (sec) | |
| Exposure 9 (sec) | |
| Ladder Channel | |

File: Stat1_Jess

## Plate Layout

| | 1 | 2 | 3 | 4 | 5 | 6 | 7 | 8 | 9 | 10 | 11 | 12 | 13 | 14 | 15 | 16 | 17 | 18 | 19 | 20 | 21 | 22 | 23 | 24 | 25 |
|---|---|---|---|---|---|---|---|---|---|---|---|---|---|---|---|---|---|---|---|---|---|---|---|---|---|
| A | B... | Y... | Y... | Y... | Y... | Y... | O... | O... | O... | O... | O... | Y... | Y... | Y... | Y... | Y... | O... | O... | O... | O... | O... | Sample | | | |
| B | Antibody Diluent | | | | | | | | | | | | | | | | | | | | | | | | |
| C | A... | Actin | | | | | | | | | | Stat1 | | | | | | | | | | Primary | | | |
| D | S... | Secondary | | | | | | | | | | | | | | | | | | | | | | | |
| E | Luminol/Peroxide | | | | | | | | | | | | | | | | | | | | | | | | |



proteinsimple a biotechne brand

File: Stat1_Jess

## Analysis Settings

| | |
|---|---|
| Hidden Capillaries | None |
| Images | |
| CHEMI Luminescence | High Dynamic Range 4.0 |
| Lane Contrast | Slider |
| CHEMI White Level | 18.3 |
| CHEMI Black Level | 13209.1 |
| Peak Fit | fit |
| Apply To | Default, Biot. Ladder |
| Range Min (MW (kDa)) | 1.0 |
| Range Max (MW (kDa)) | 250.0 |
| Range View | Analysis |
| Baseline Type | Spline |
| Baseline Threshold | 1.0 |
| Baseline Window (pixels) | 15.0 |
| Baseline Stiffness | 1.0 |
| Peak Find Threshold | 10.0 |
| Peak Find Width (pixels) | 9.0 |
| Peak Find Area Calculation | Gaussian Fit |
| Ladder Settings | Cap: 1, Chemiluminescent/Fluorescent Channel: CHEMI |
| Apply To | Default |
| Ladder Peak 1 (MW (kDa)) | 12.0 |
| Ladder Peak 2 (MW (kDa)) | 40.0 |
| Ladder Peak 3 (MW (kDa)) | 66.0 |
| Ladder Peak 4 (MW (kDa)) | 116.0 |
| Ladder Peak 5 (MW (kDa)) | 180.0 |
| Ladder Peak 6 (MW (kDa)) | 230.0 |
| Signal To Noise | |
| Algorithm | Signal-to-Noise Ratio |
| Region Spectrum | Full |
| Region Units | kDa |
| Standards | Biotinylated Ladder (12kDa-230kDa) |
| Apply To | Default |
| Standard 1 (MW (kDa)) | 1 |
| Standard 1 Position (pixels) | 120 |
| Standard 1 Fit | no |
| Standard 1 Registration | yes |
| Standard 2 (MW (kDa)) | 29 |
| Standard 2 Position (pixels) | 270 |
| Standard 2 Fit | yes |
| Standard 2 Registration | no |
| Standard 3 (MW (kDa)) | 230 |
| Standard 3 Position (pixels) | 530 |
| Standard 3 Fit | yes |
| Standard 3 Registration | no |
| Advanced | Advanced |
| Apply To | Default |
| Standards Peak Width (pixels) | 15 |
| Standards Allowable Drift (pixels) | 100 |

File: Stat1_Jess

| | |
|---|---|
| Sample Peak Fit Starting Width Ratio | 0.5 |
| Image Median Filter Threshold Ratio | 0.5 |
| Image Median Filter Threshold Limit (counts) | 1000 |

File: Stat1_Jess

CHEMI

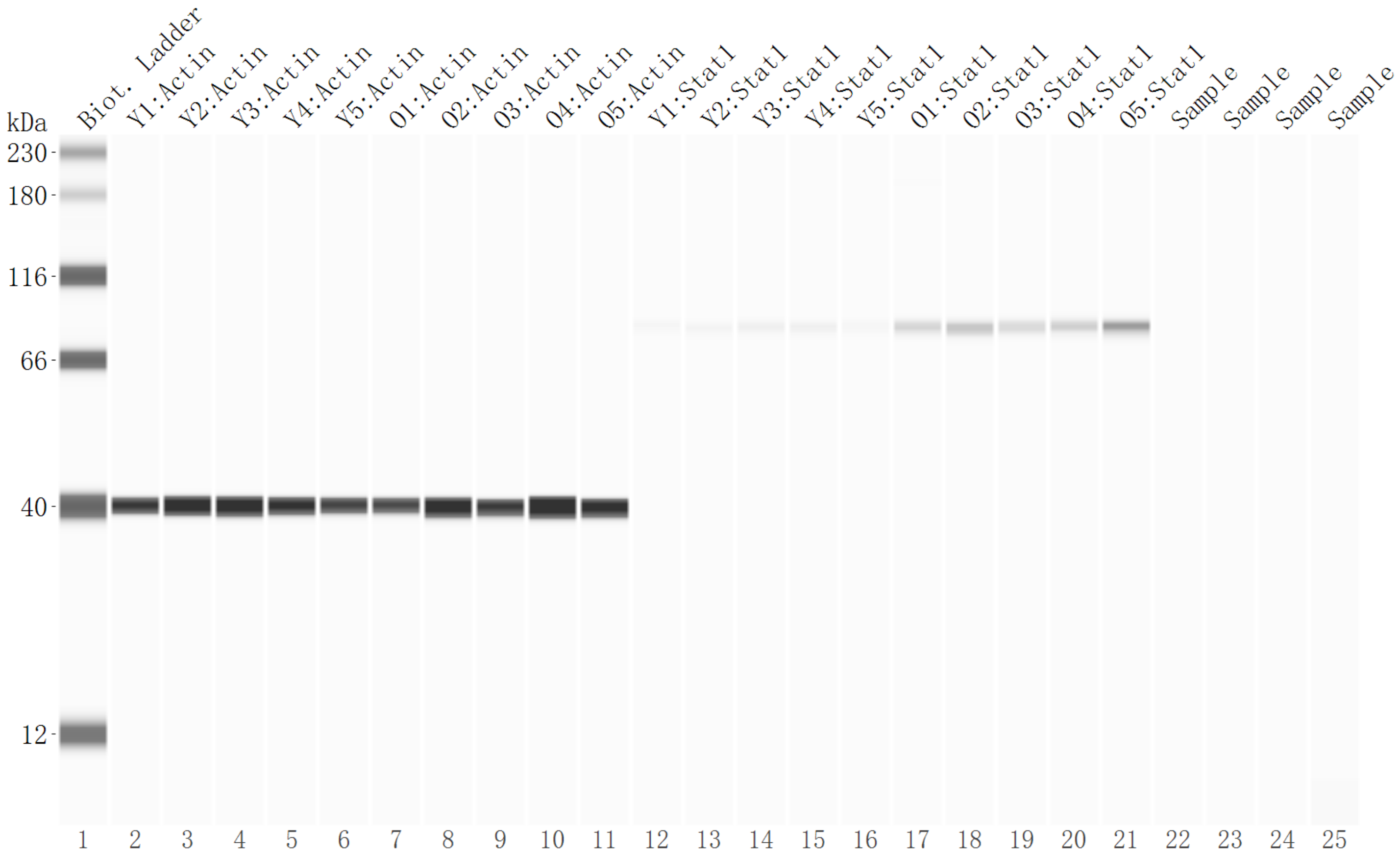


C:\Users\\OneDrive\Desktop\Stat1_Jess.cbz

File: Stat1_Jess

## Standard

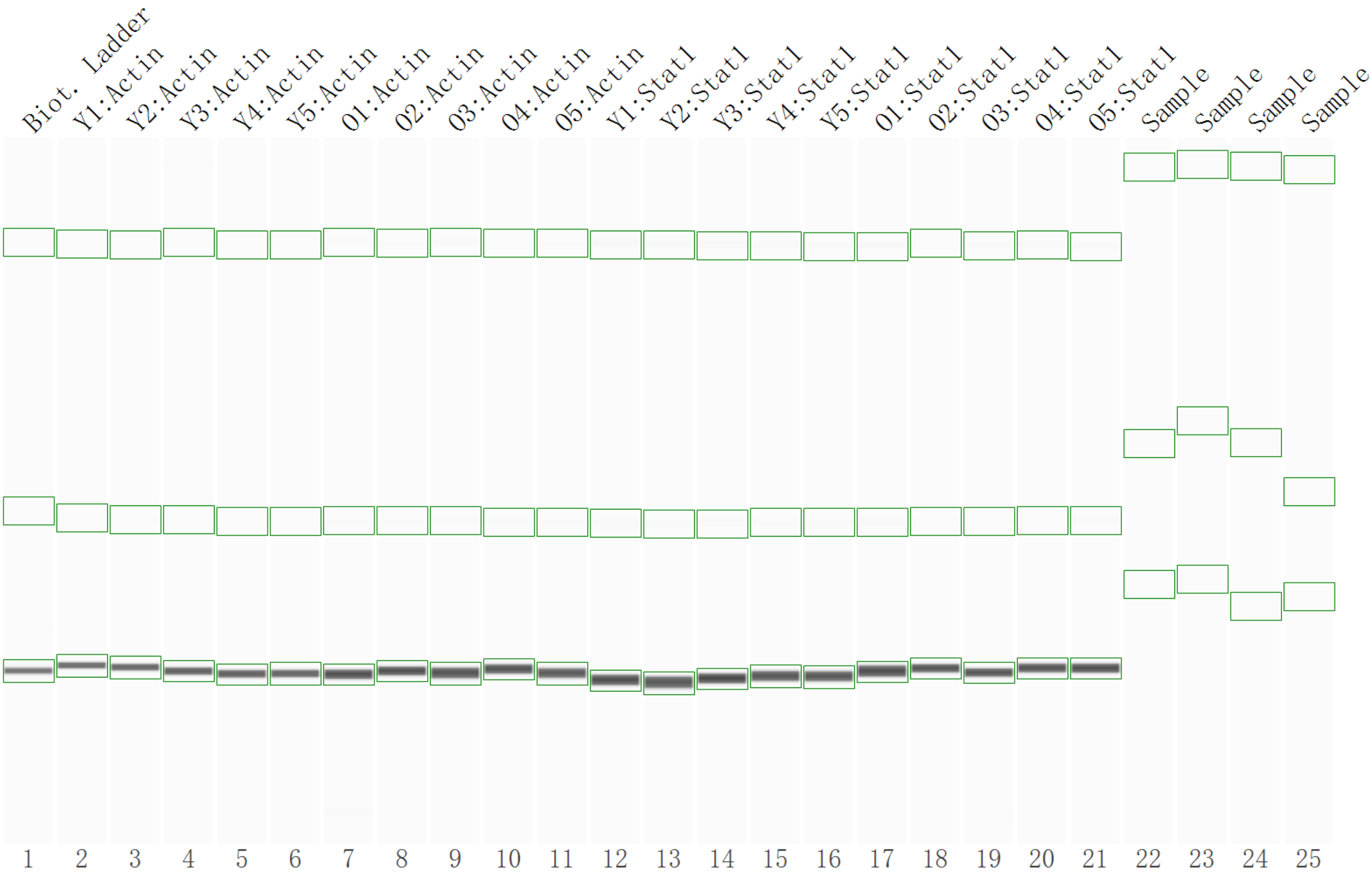

File: Stat1_Jess

# IV Plot

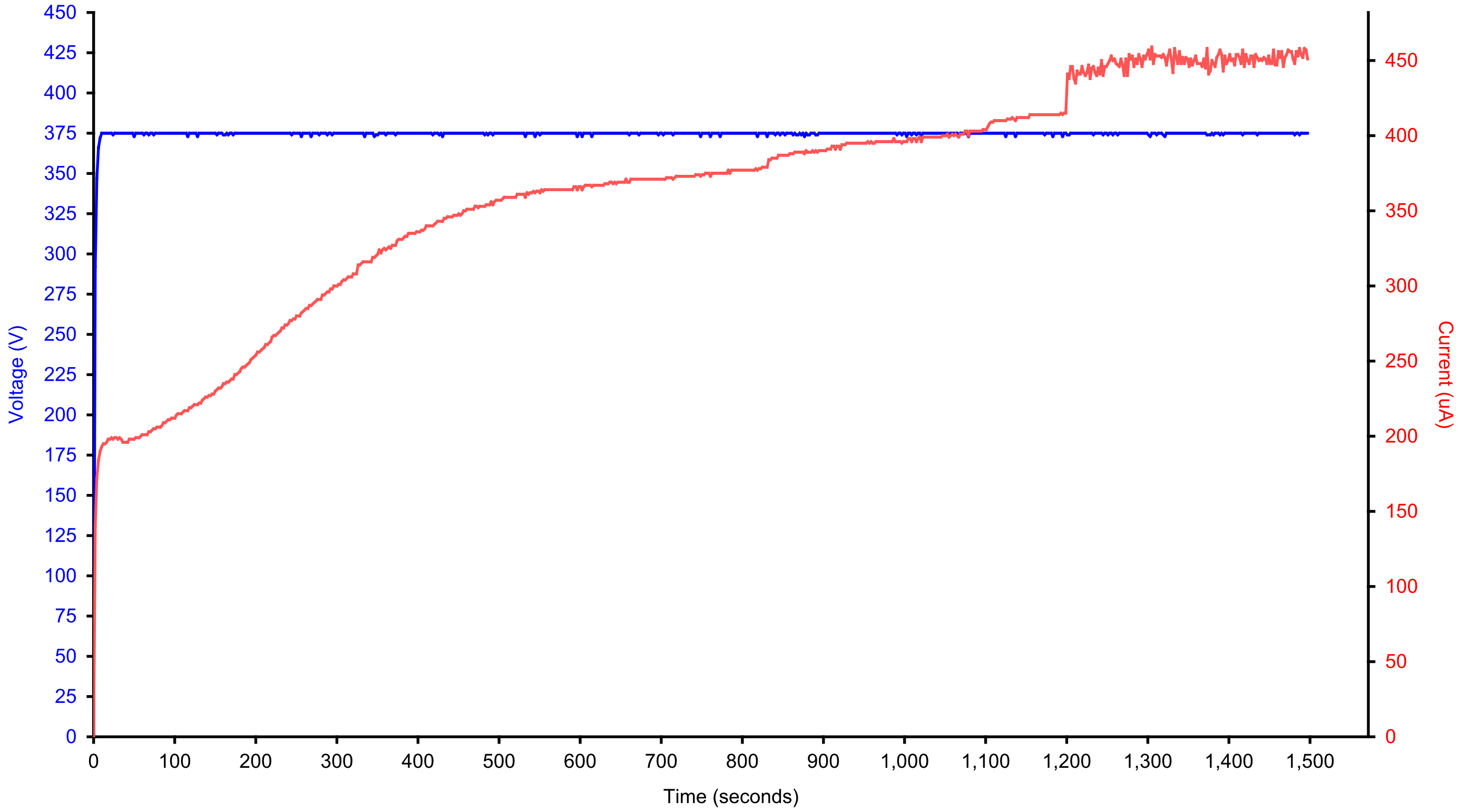

File: Stat1_Jess

## CHEMI Sample Plots

Biot. Ladder, Cap:1

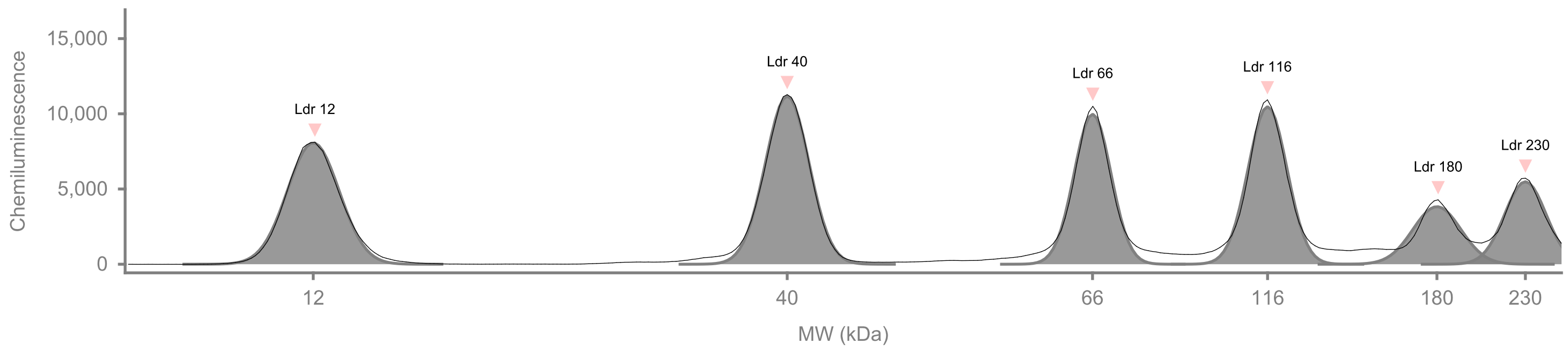


Y1:Actin, Cap:2

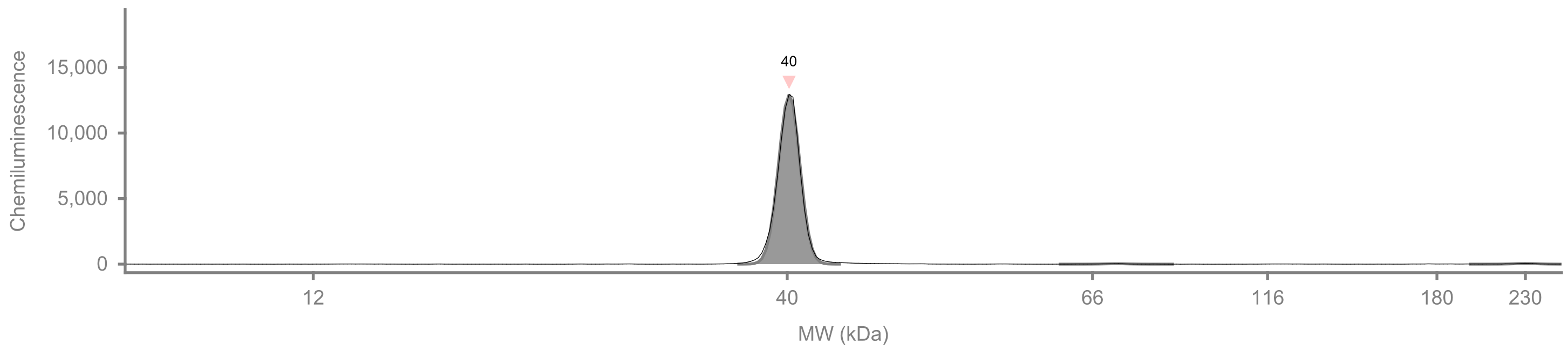


Y2:Actin, Cap:3

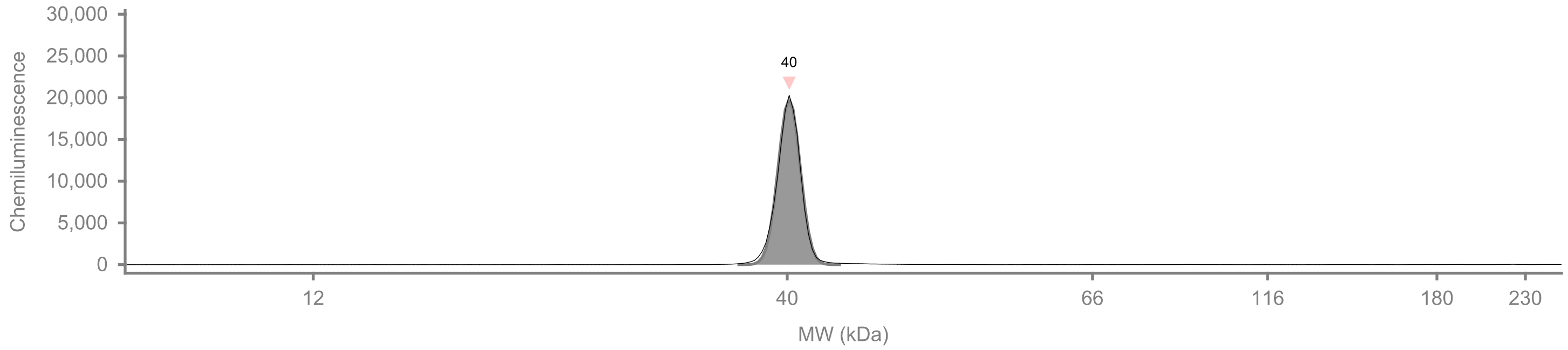


Y3:Actin, Cap:4

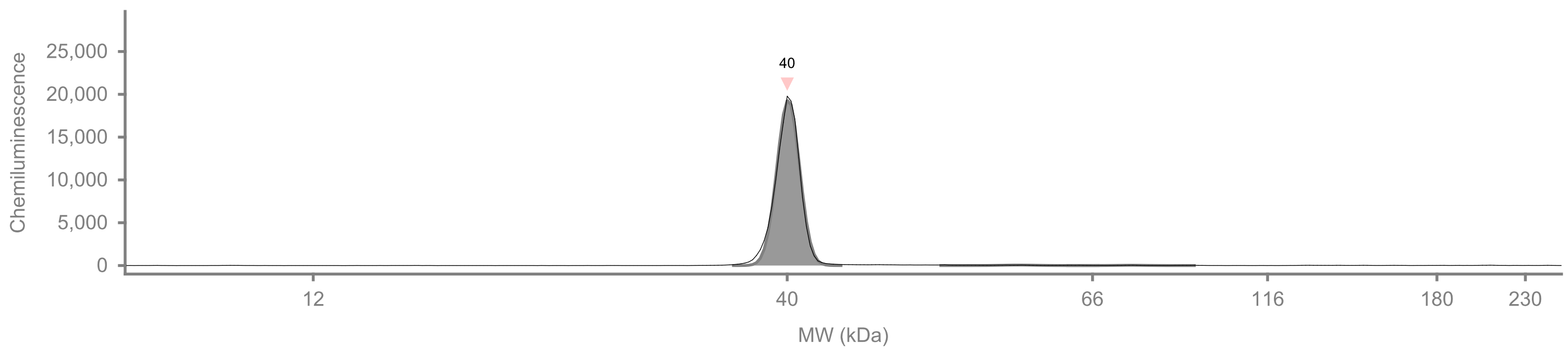




proteinsimple a biotechne brand

# File: Stat1_Jess

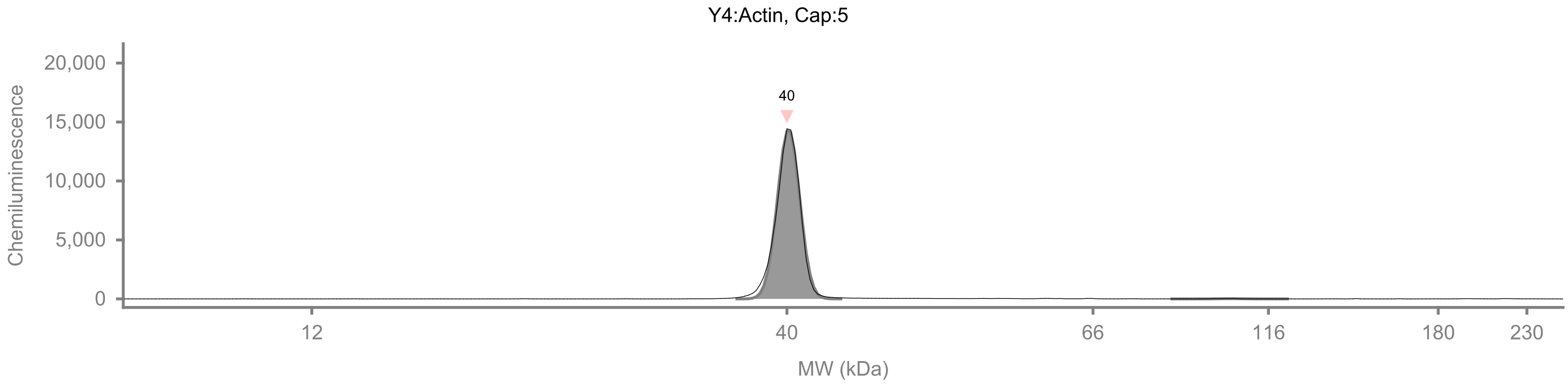


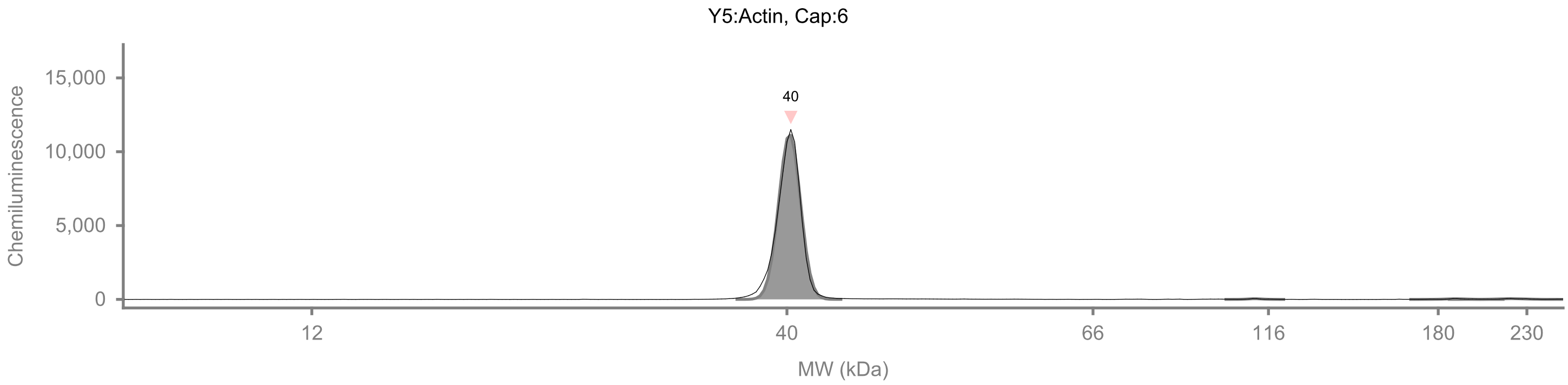


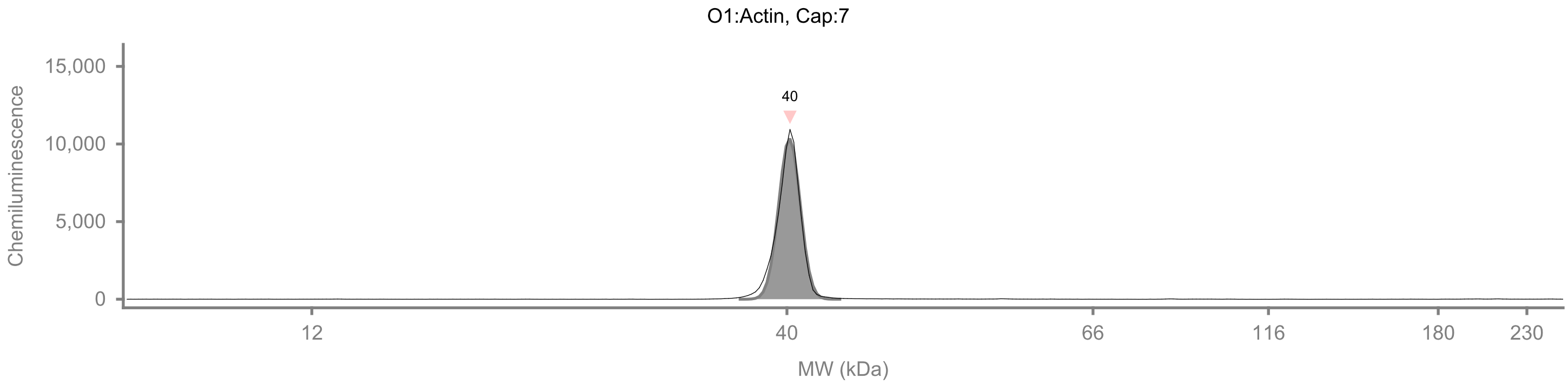


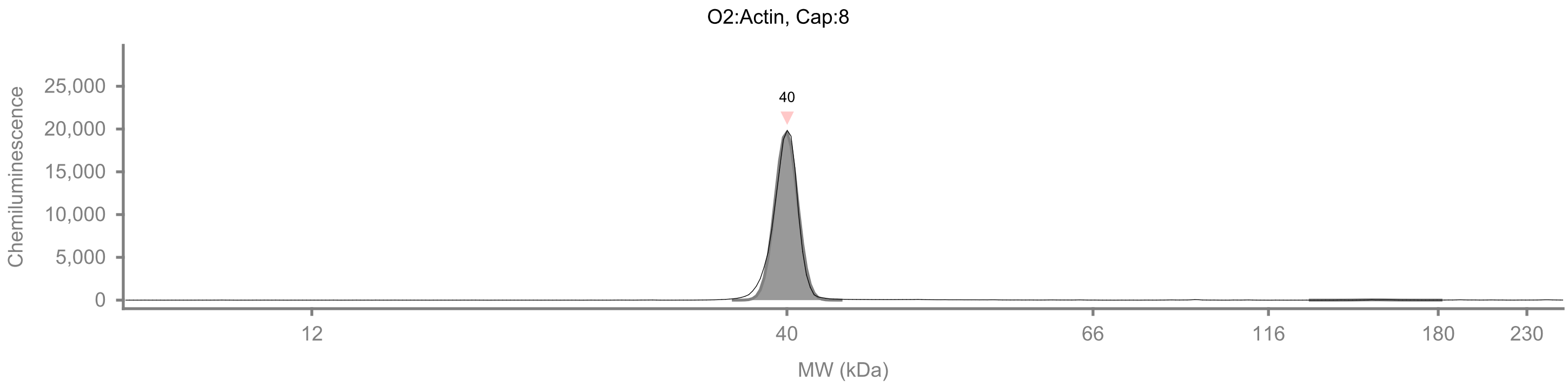

# File: Stat1_Jess

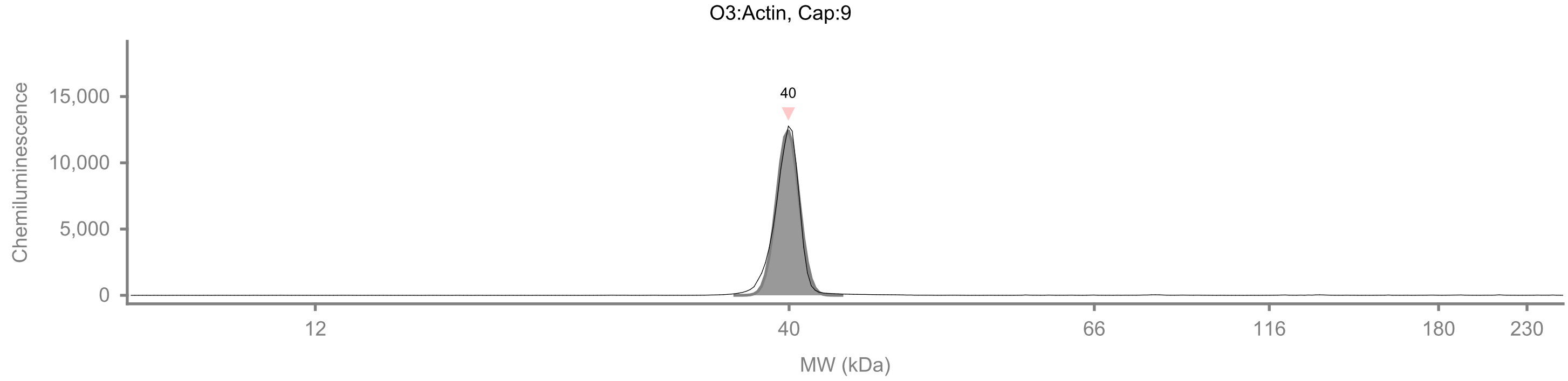


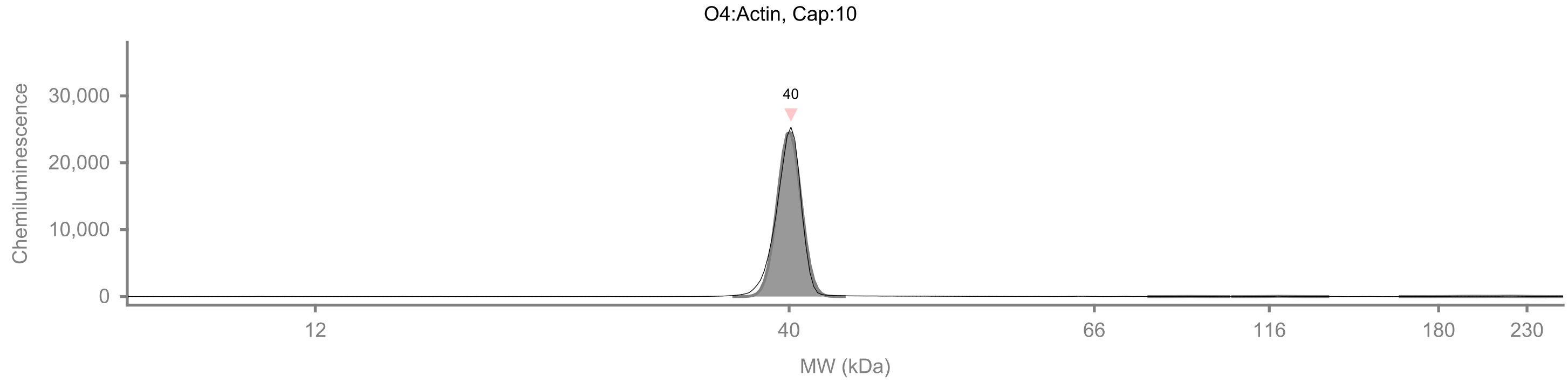


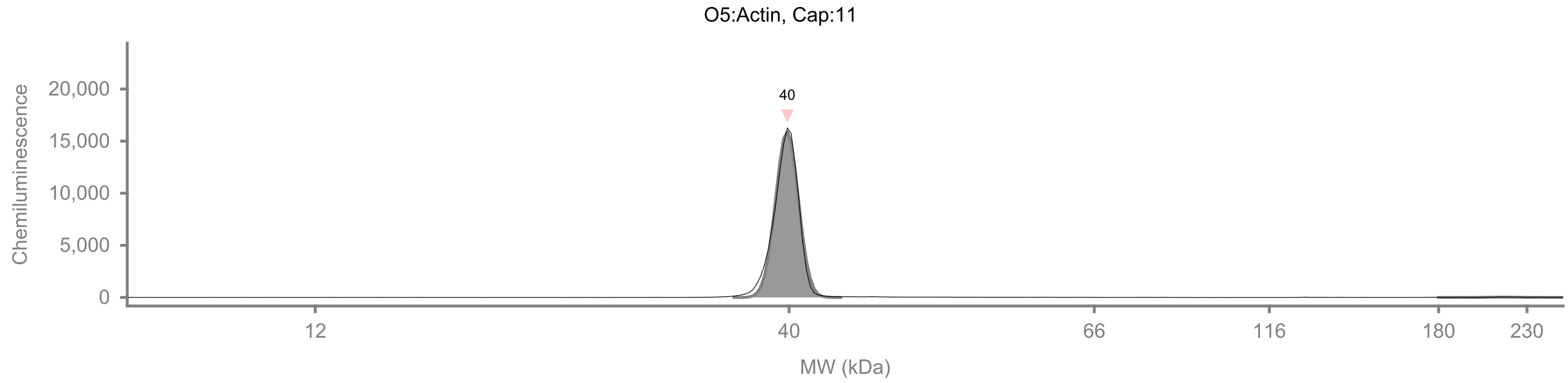


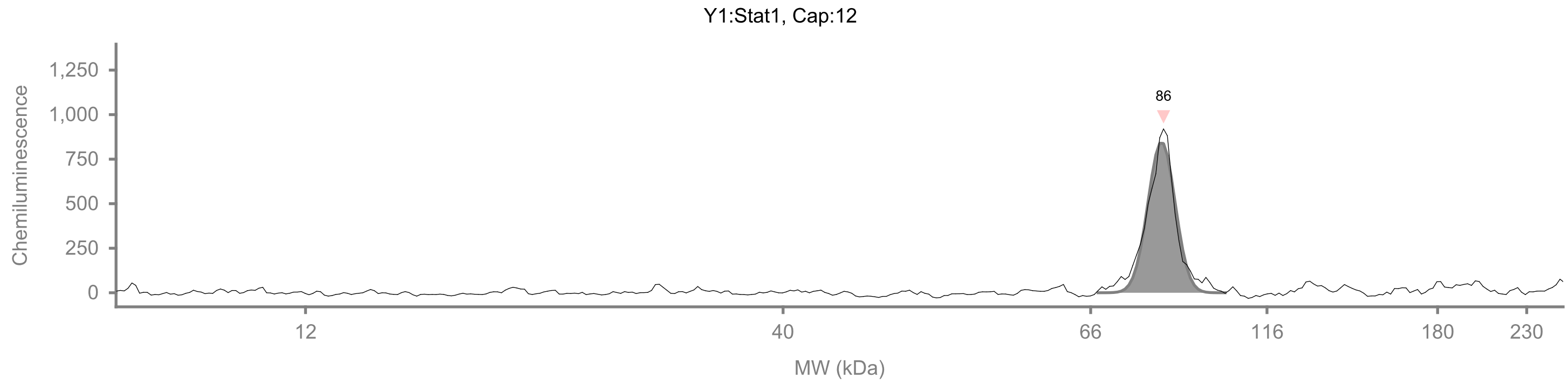


C:\Users\\OneDrive\Desktop\Stat1_Jess.cbz

# File: Stat1_Jess

Y2:Stat1, Cap:13

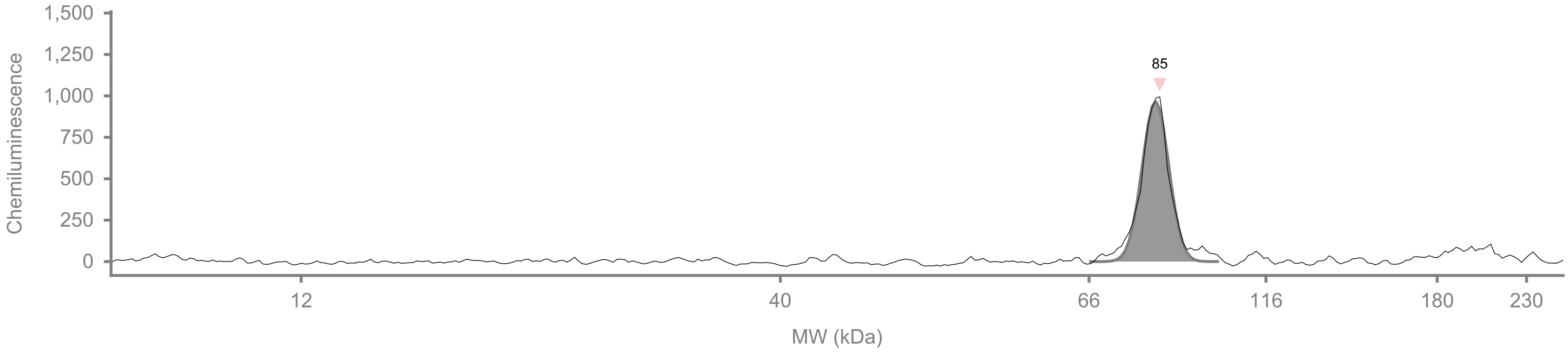


Y3:Stat1, Cap:14

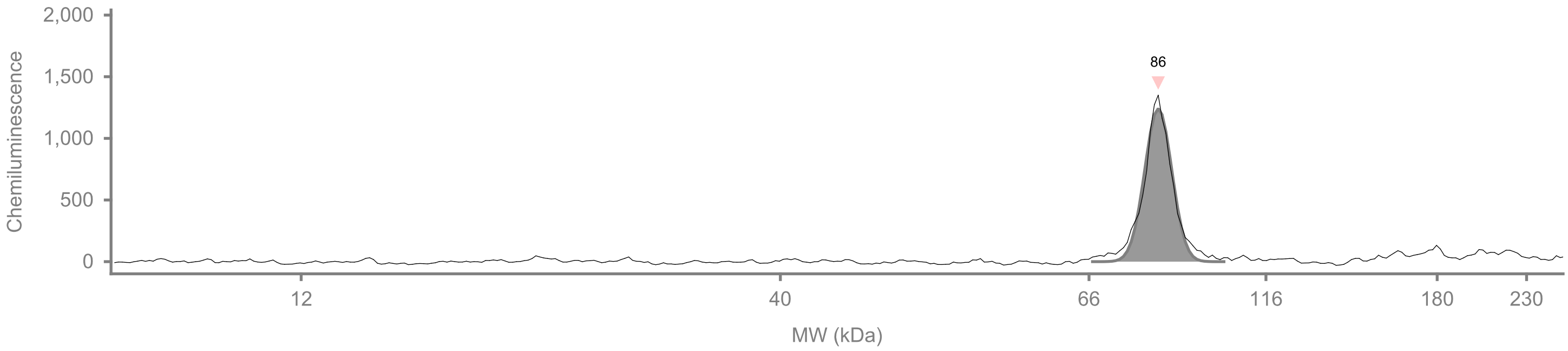


Y4:Stat1, Cap:15

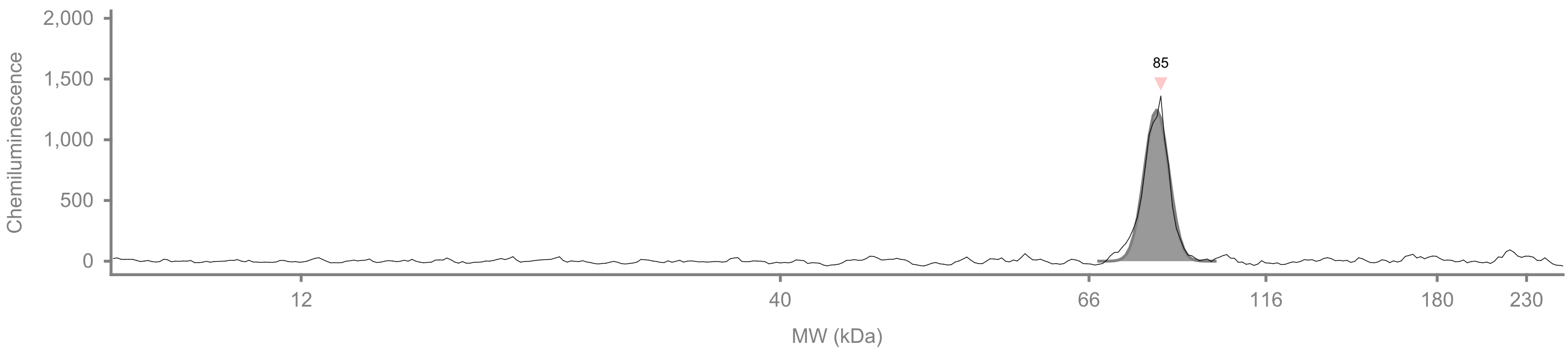


Y5:Stat1, Cap:16

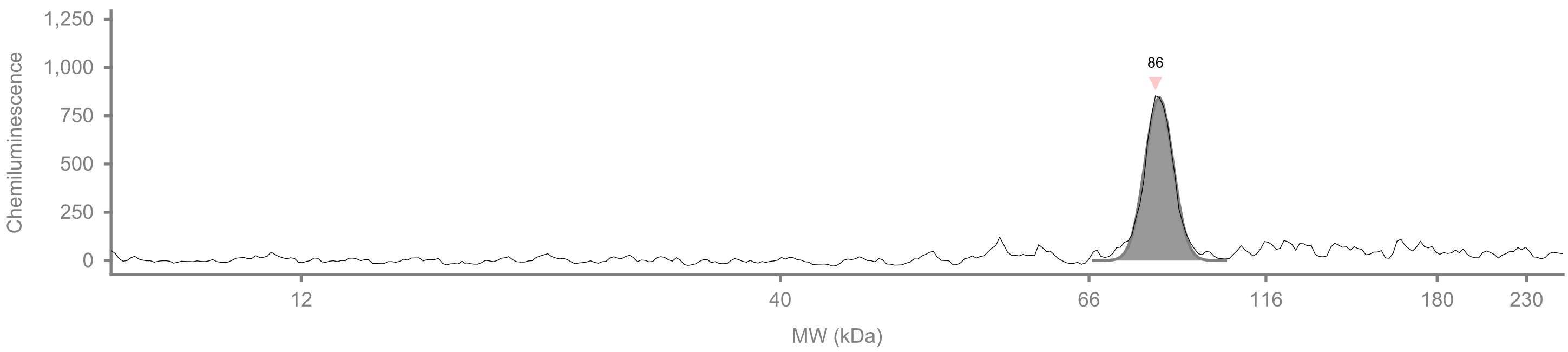

# File: Stat1_Jess

O1:Stat1, Cap:17

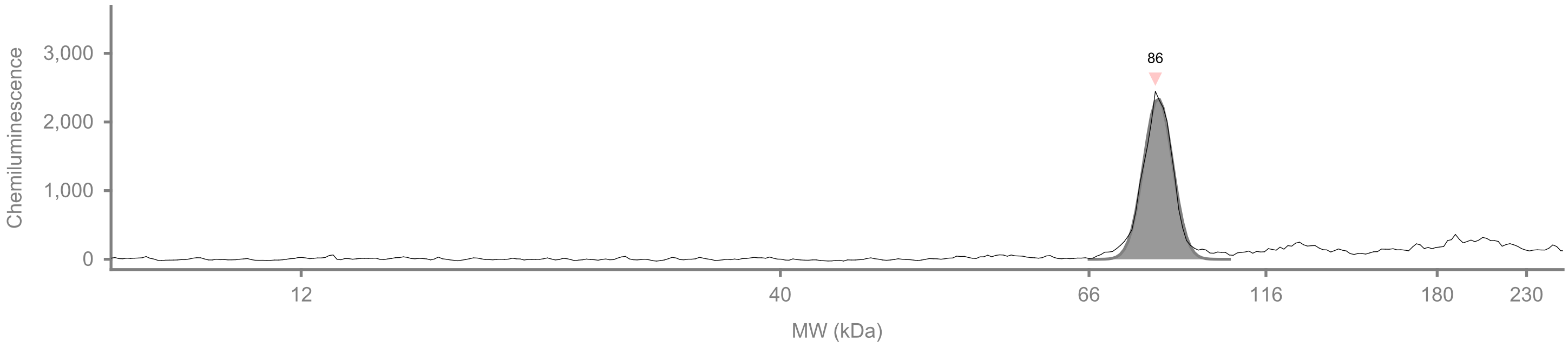


O2:Stat1, Cap:18

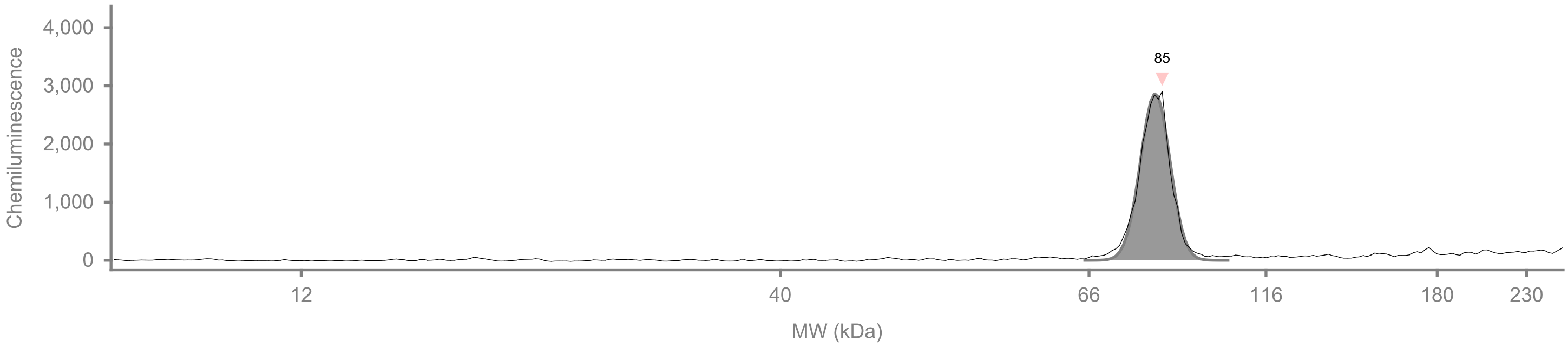


O3:Stat1, Cap:19

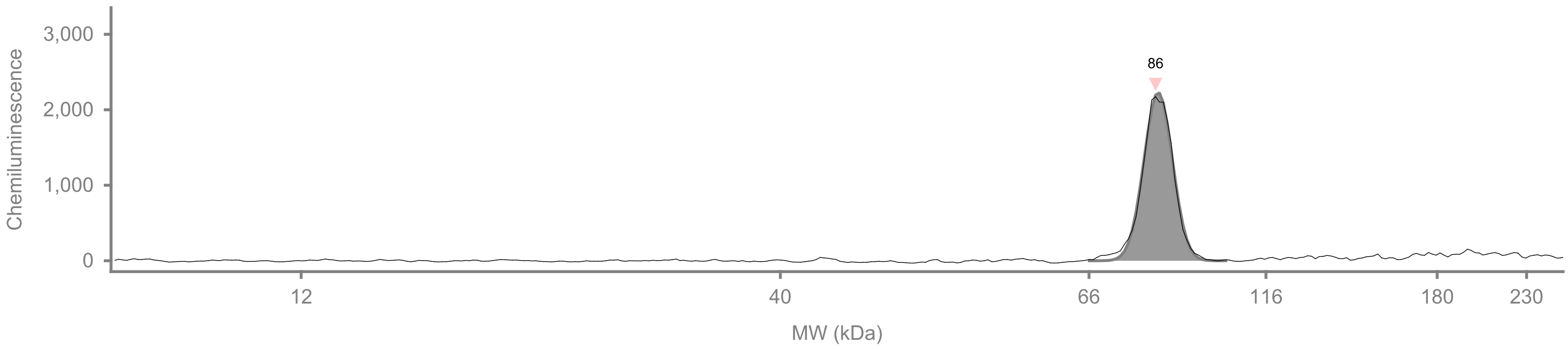


O4:Stat1, Cap:20

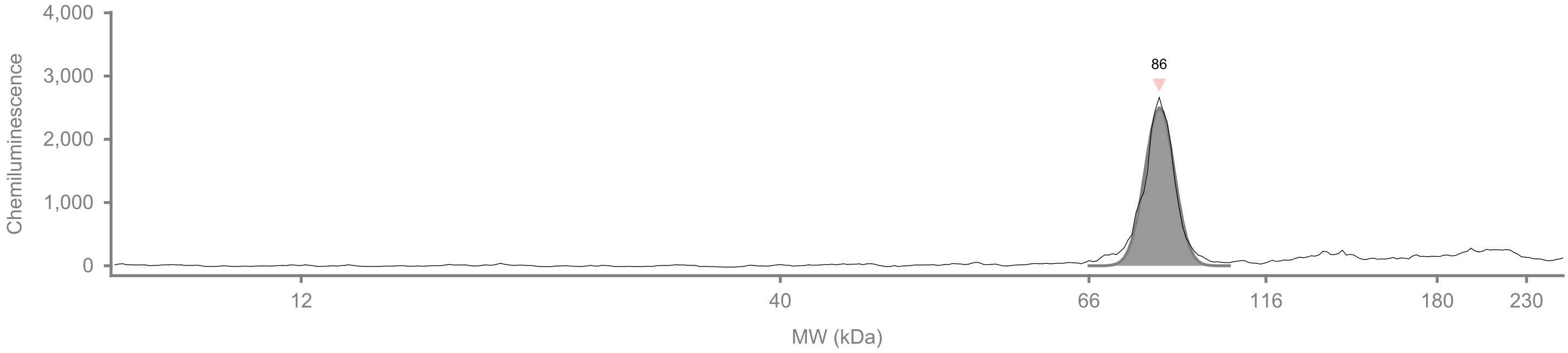

# File: Stat1_Jess

O5:Stat1, Cap:21

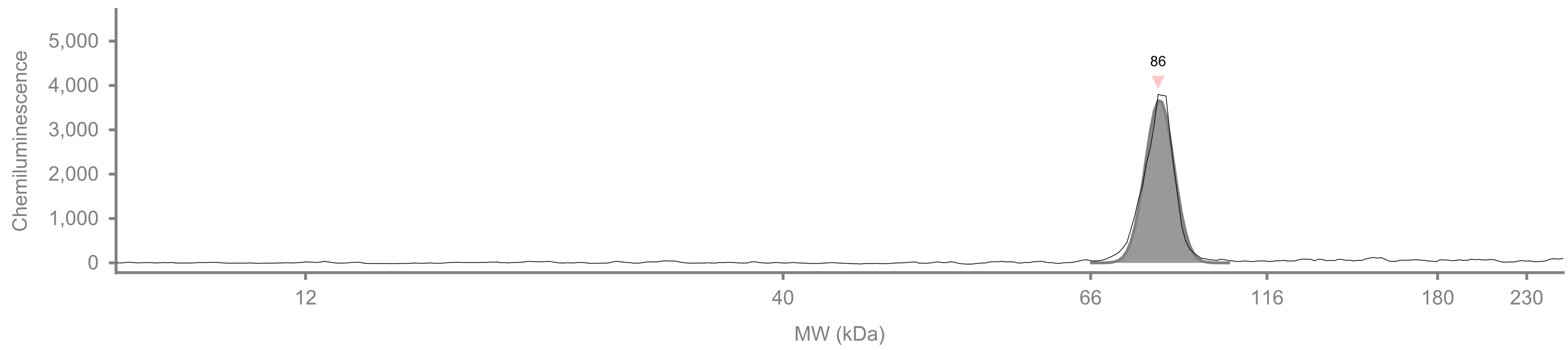


Sample, Cap:22

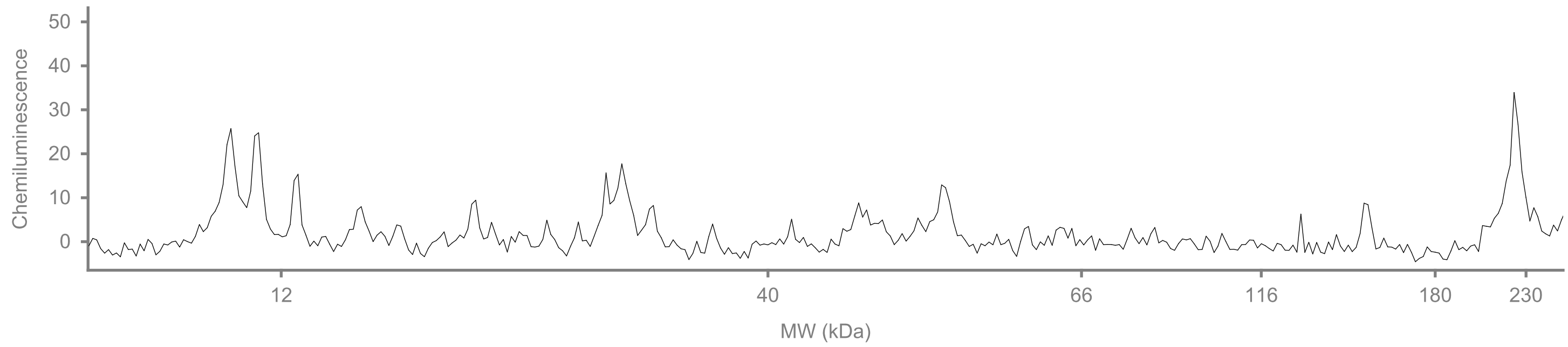


Sample, Cap:23

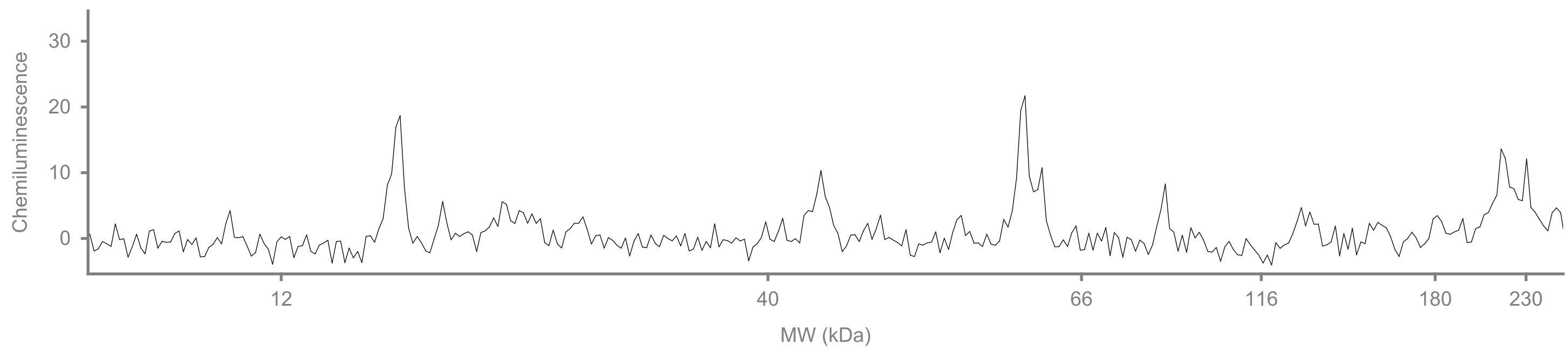


Sample, Cap:24

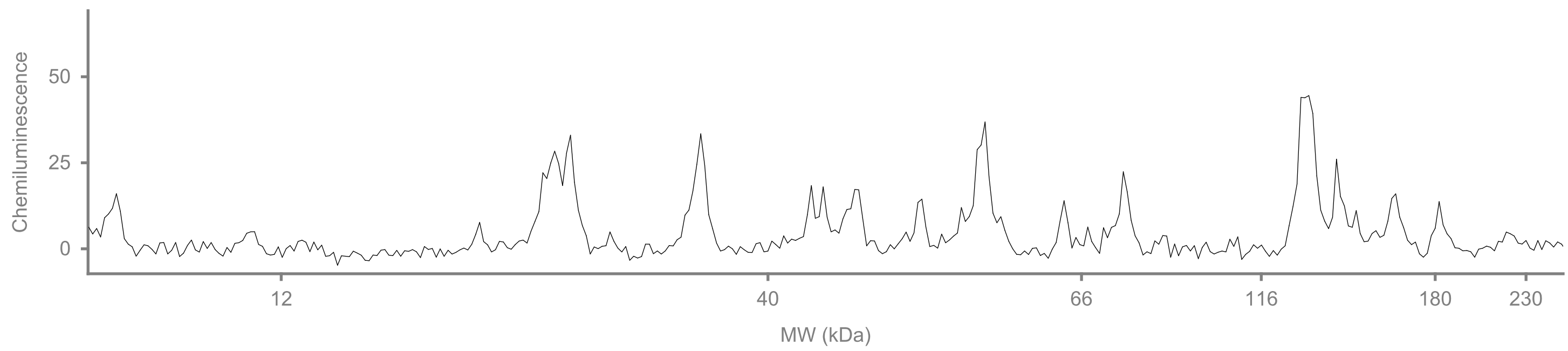


C:\Users\\OneDrive\Desktop\Stat1_Jess.cbz

proteinsimple
a biotechne brand

# File: Stat1_Jess

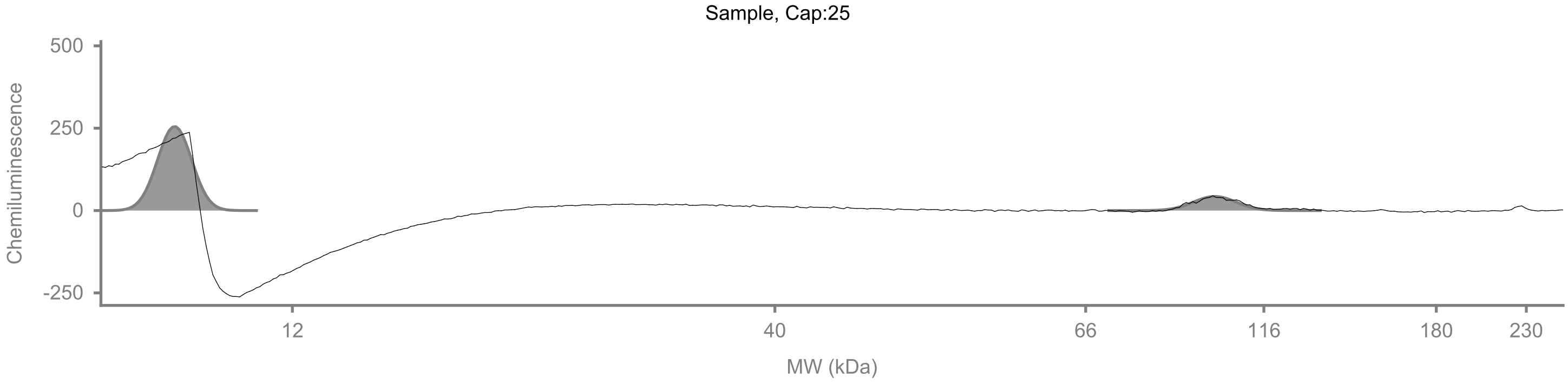

# CHEMI Sample Peaks

| | | | | | | | | | | | | | |
|---|---|---|---|---|---|---|---|---|---|---|---|---|---|
| Biot. Ladder | Antibody Diluent | Streptavidin HRP | 1 | 1 | Ldr 12 | 246 | 12 | 8068.4 | 131939.0 | | 15.4 | 2999.9 | 71.0 |
| Biot. Ladder | Antibody Diluent | Streptavidin HRP | 1 | 2 | Ldr 40 | 366 | 40 | 11172.4 | 153307.1 | | 12.9 | 4154.0 | 104.5 |
| Biot. Ladder | Antibody Diluent | Streptavidin HRP | 1 | 3 | Ldr 66 | 443 | 66 | 9975.6 | 116159.7 | | 10.9 | 3709.0 | 112.3 |
| Biot. Ladder | Antibody Diluent | Streptavidin HRP | 1 | 4 | Ldr 116 | 487 | 116 | 10472.4 | 128877.7 | | 11.6 | 3893.7 | 113.3 |
| Biot. Ladder | Antibody Diluent | Streptavidin HRP | 1 | 5 | Ldr 180 | 530 | 180 | 3824.8 | 57853.6 | | 14.2 | 1422.1 | 112.6 |
| Biot. Ladder | Antibody Diluent | Streptavidin HRP | 1 | 6 | Ldr 230 | 552 | 230 | 5481.5 | 71697.4 | | 12.3 | 2038.1 | 111.9 |
| Y1:Actin | Actin | Secondary | 2 | 1 | | 361 | 40 | 12942.1 | 87716.4 | | 6.4 | 6143.4 | 9.4 |
| Y2:Actin | Actin | Secondary | 3 | 1 | | 360 | 40 | 19847.8 | 138488.0 | | 6.6 | 8782.7 | 9.2 |
| Y3:Actin | Actin | Secondary | 4 | 1 | | 360 | 40 | 19395.6 | 141433.9 | | 6.9 | 9799.6 | 8.8 |
| Y4:Actin | Actin | Secondary | 5 | 1 | | 358 | 40 | 14431.7 | 102572.0 | | 6.7 | 6554.4 | 13.0 |
| Y5:Actin | Actin | Secondary | 6 | 1 | | 358 | 40 | 11227.8 | 77824.8 | | 6.5 | 5280.0 | 14.0 |
| O1:Actin | Actin | Secondary | 7 | 1 | | 360 | 40 | 10362.6 | 71587.9 | | 6.5 | 3977.1 | 17.4 |
| O2:Actin | Actin | Secondary | 8 | 1 | | 359 | 40 | 19726.5 | 141612.1 | | 6.7 | 8679.5 | 13.6 |
| O3:Actin | Actin | Secondary | 9 | 1 | | 359 | 40 | 12484.4 | 89605.9 | | 6.7 | 5439.8 | 12.3 |
| O4:Actin | Actin | Secondary | 10 | 1 | | 357 | 40 | 24781.5 | 186160.5 | | 7.1 | 6980.7 | 23.4 |
| O5:Actin | Actin | Secondary | 11 | 1 | | 357 | 40 | 15963.2 | 117329.6 | | 6.9 | 8970.9 | 19.2 |
| Y1:Stat1 | Stat1 | Secondary | 12 | 1 | | 455 | 86 | 847.7 | 7389.6 | | 8.2 | 94.0 | 85.9 |
| Y2:Stat1 | Stat1 | Secondary | 13 | 1 | | 454 | 85 | 967.9 | 8252.2 | | 8.0 | 113.8 | 90.9 |
| Y3:Stat1 | Stat1 | Secondary | 14 | 1 | | 454 | 86 | 1242.3 | 10777.1 | | 8.2 | 144.2 | 92.2 |
| Y4:Stat1 | Stat1 | Secondary | 15 | 1 | | 454 | 85 | 1249.9 | 9815.5 | | 7.4 | 134.4 | 114.9 |
| Y5:Stat1 | Stat1 | Secondary | 16 | 1 | | 454 | 86 | 847.5 | 7592.8 | | 8.4 | 101.6 | 94.0 |
| O1:Stat1 | Stat1 | Secondary | 17 | 1 | | 454 | 86 | 2343.9 | 21625.2 | | 8.7 | 346.3 | 80.9 |
| O2:Stat1 | Stat1 | Secondary | 18 | 1 | | 455 | 85 | 2871.3 | 27064.7 | | 8.9 | 469.0 | 63.4 |
| O3:Stat1 | Stat1 | Secondary | 19 | 1 | | 455 | 86 | 2233.6 | 20219.5 | | 8.5 | 259.0 | 106.1 |
| O4:Stat1 | Stat1 | Secondary | 20 | 1 | | 456 | 86 | 2507.0 | 23449.4 | | 8.8 | 482.5 | 57.6 |
| O5:Stat1 | Stat1 | Secondary | 21 | 1 | | 454 | 86 | 3679.7 | 33472.2 | | 8.5 | 711.8 | 81.4 |

File: Stat1_Jess

# Standard Plots

C1:1

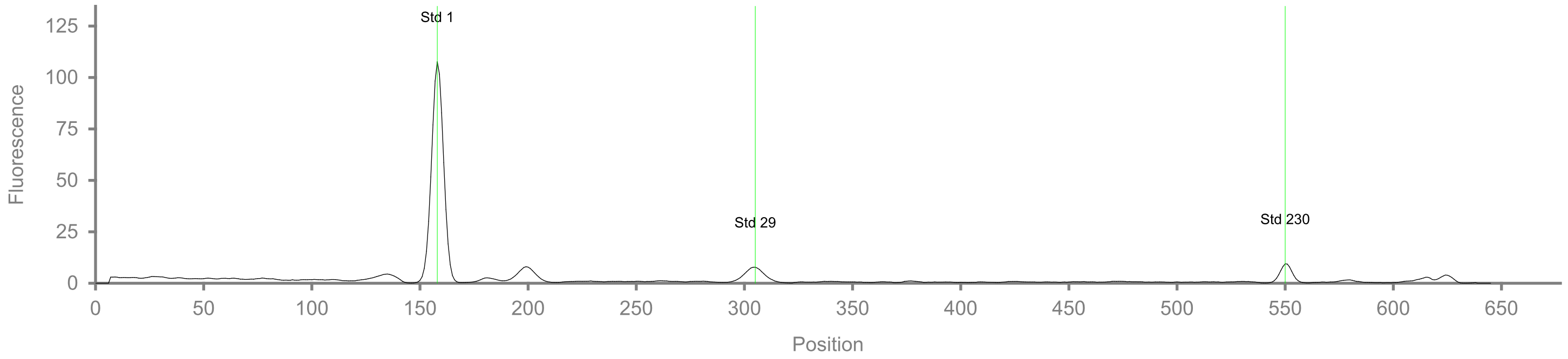


C1:2

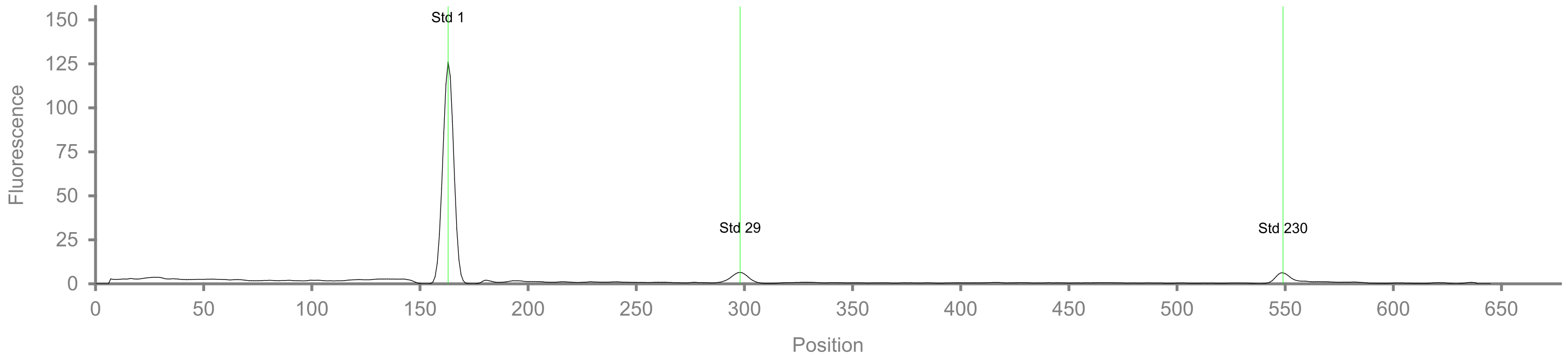


C1:3

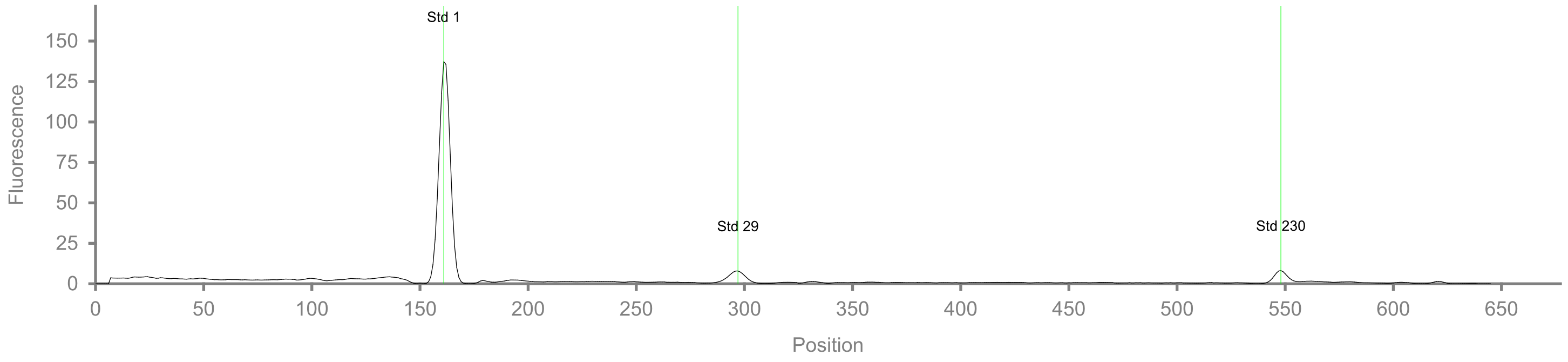


C1:4

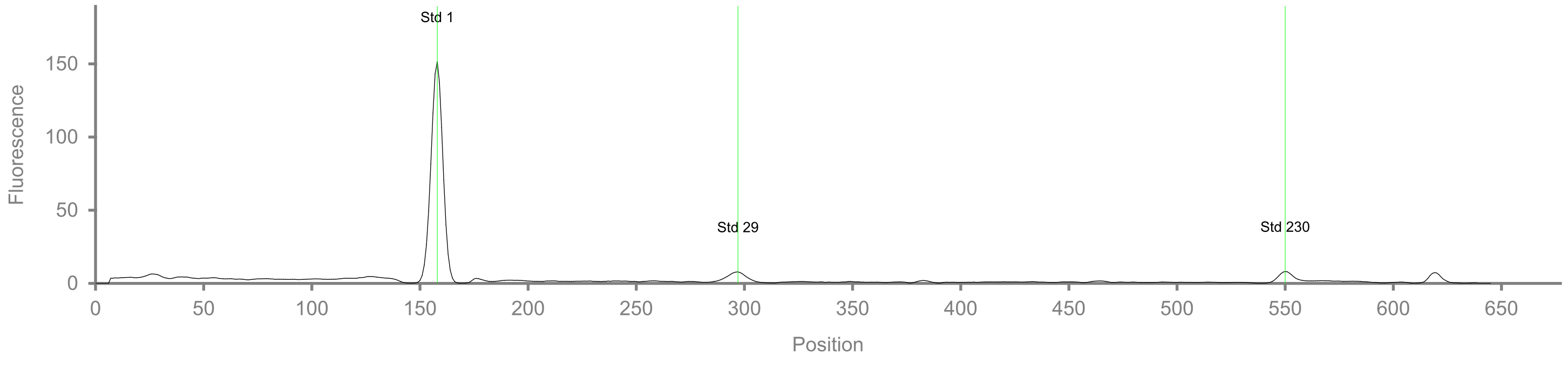

# File: Stat1_Jess

C1:5

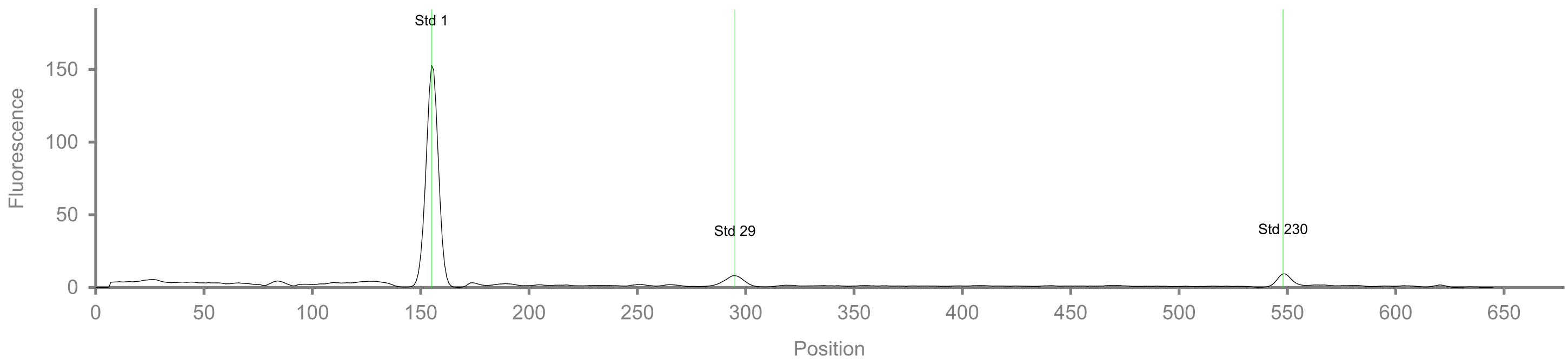


C1:6

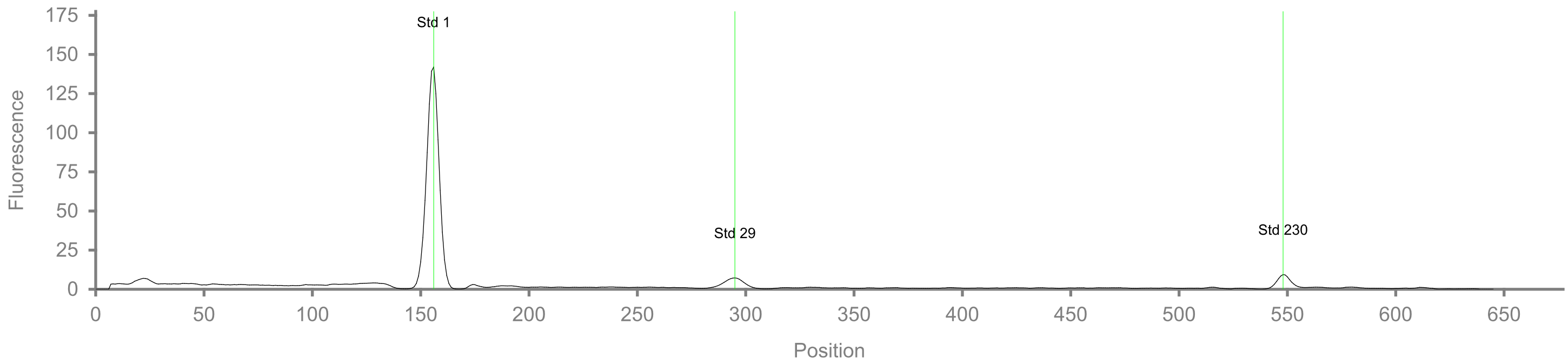


C1:7

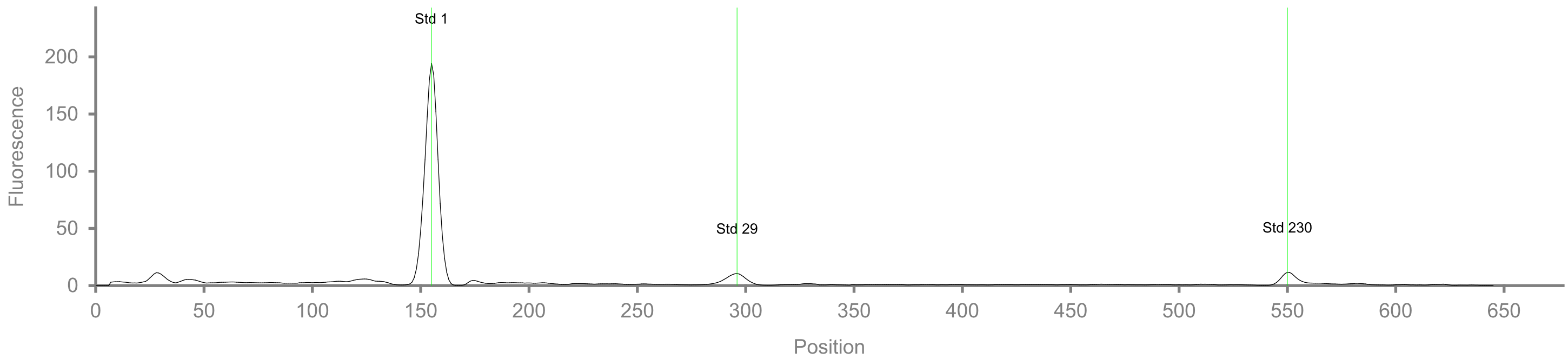


C1:8

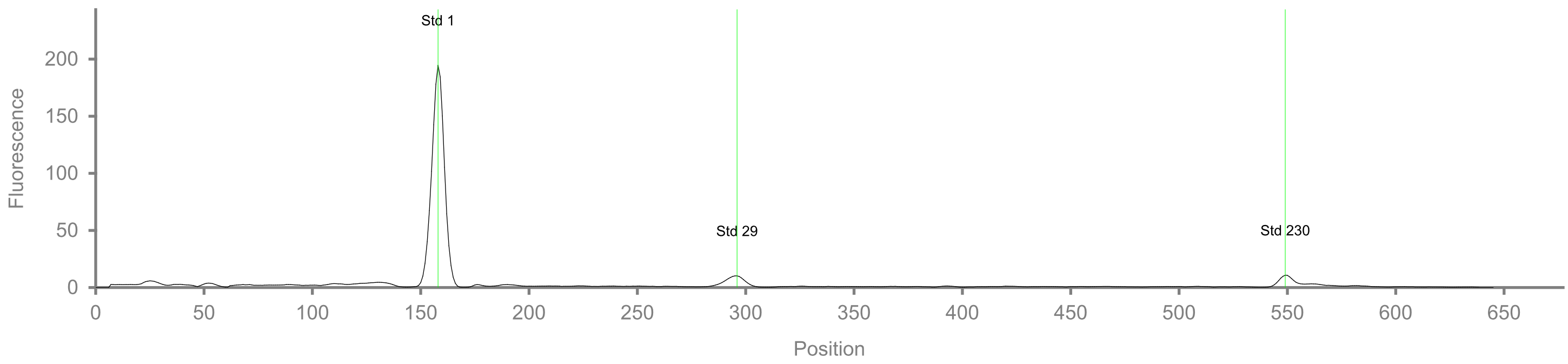


C:\Users\\OneDrive\Desktop\Stat1_Jess.cbz

# File: Stat1_Jess

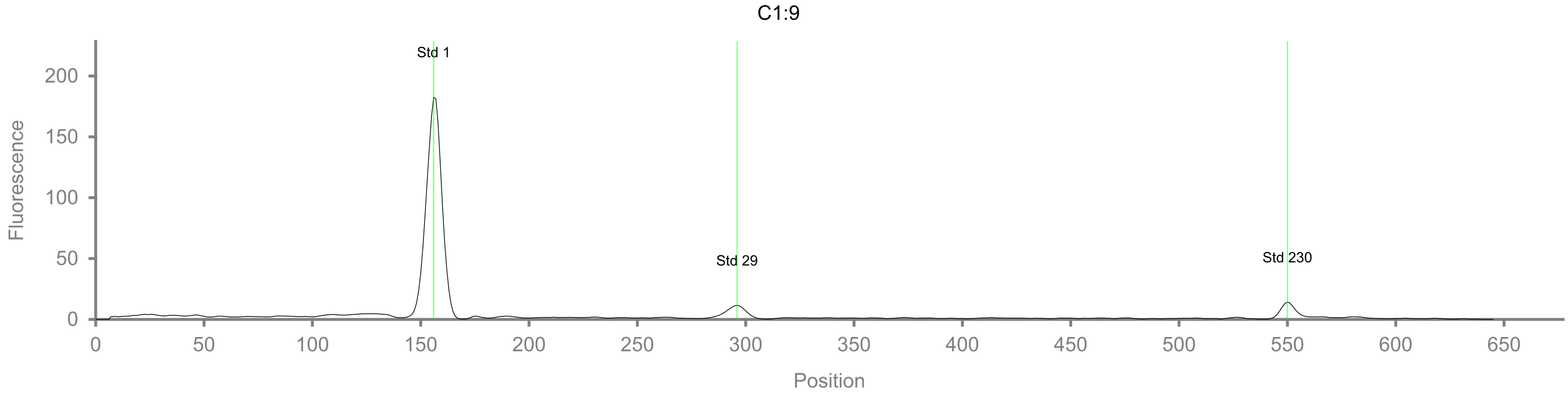


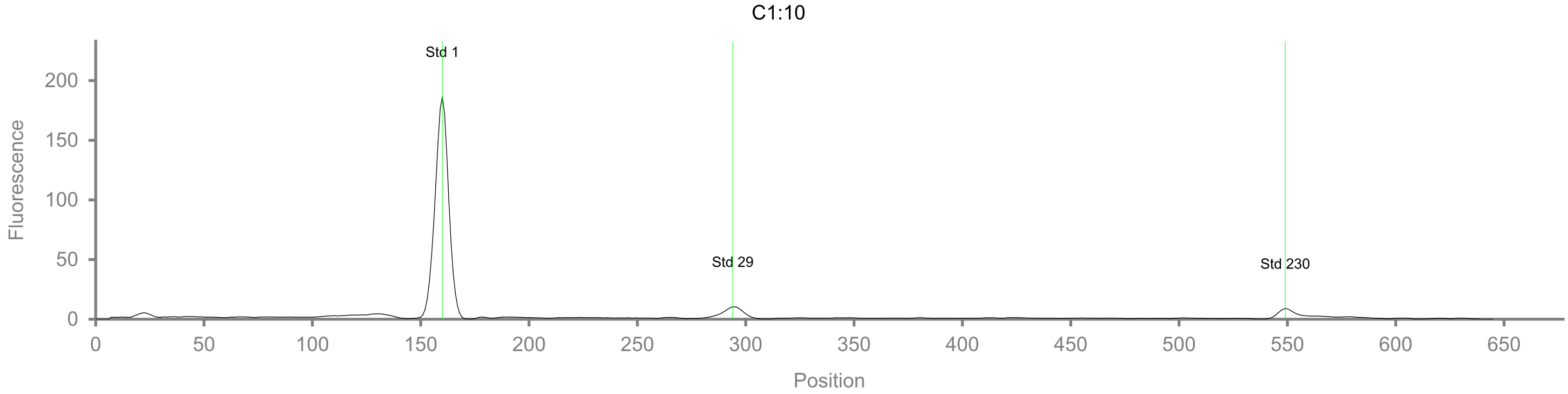


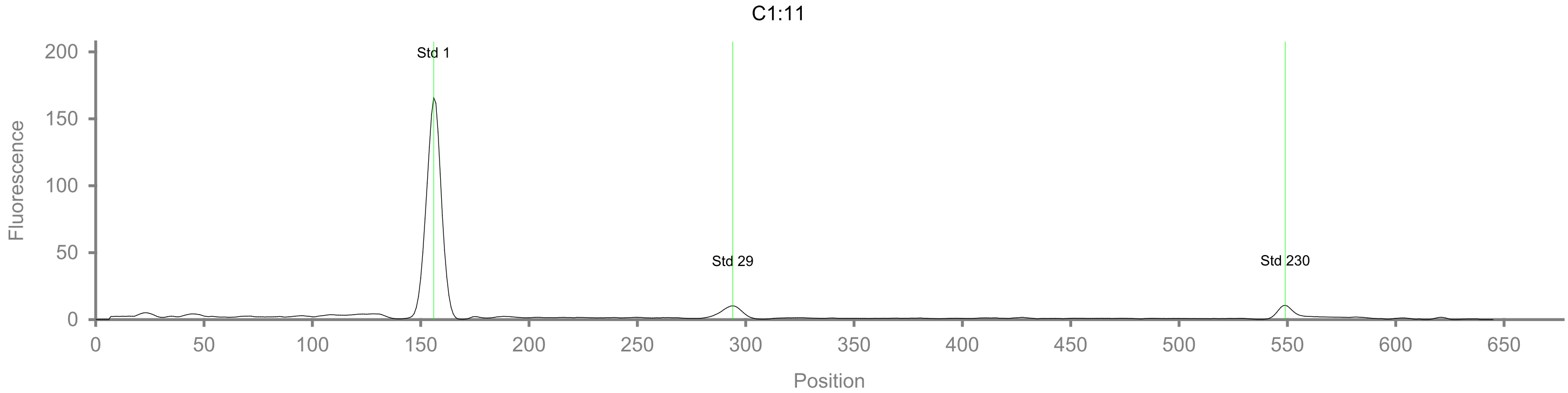


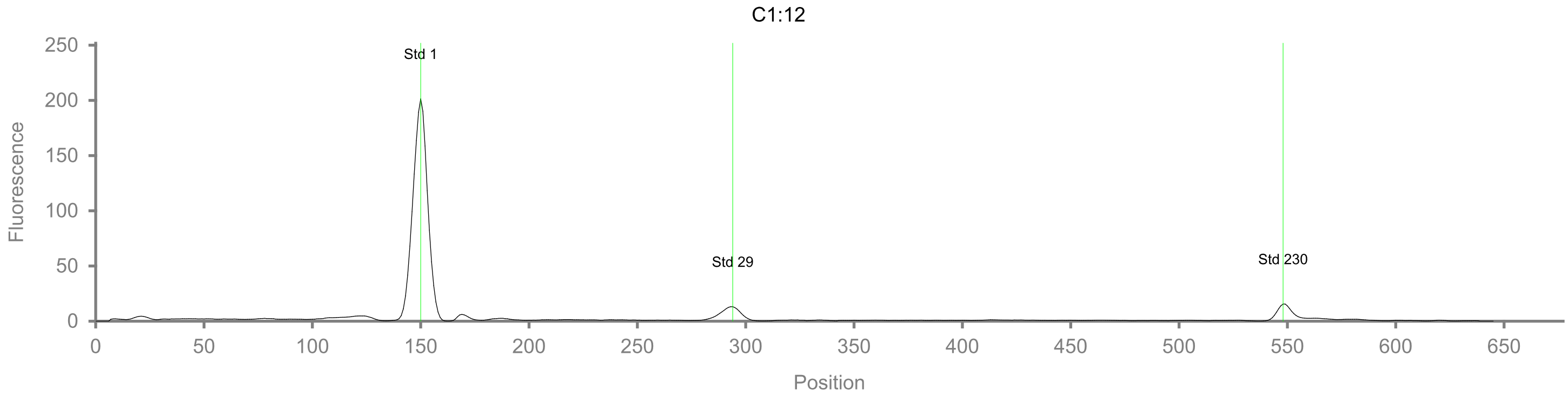

# File: Stat1_Jess

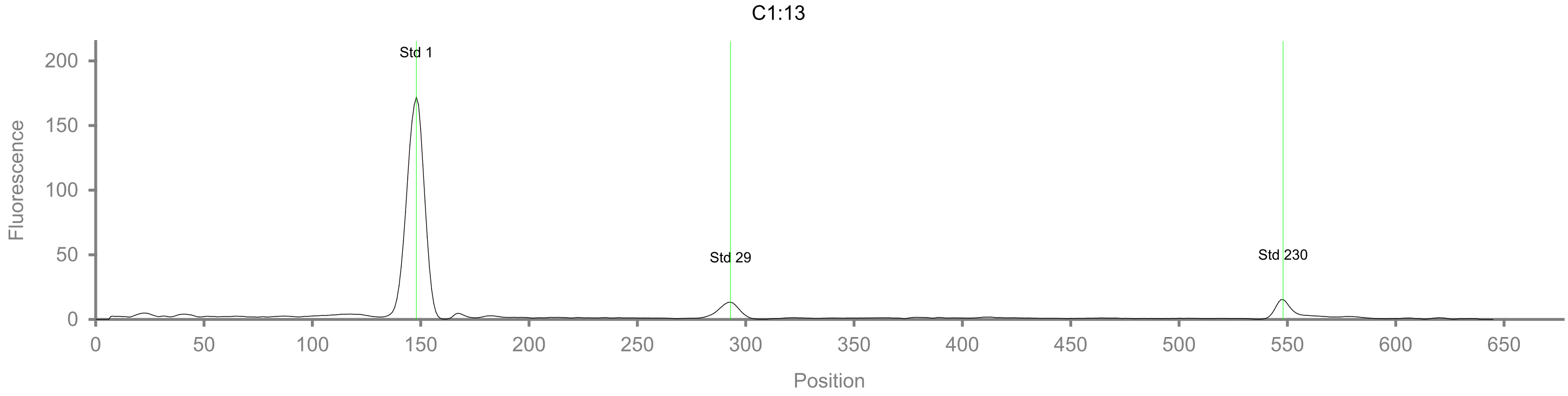


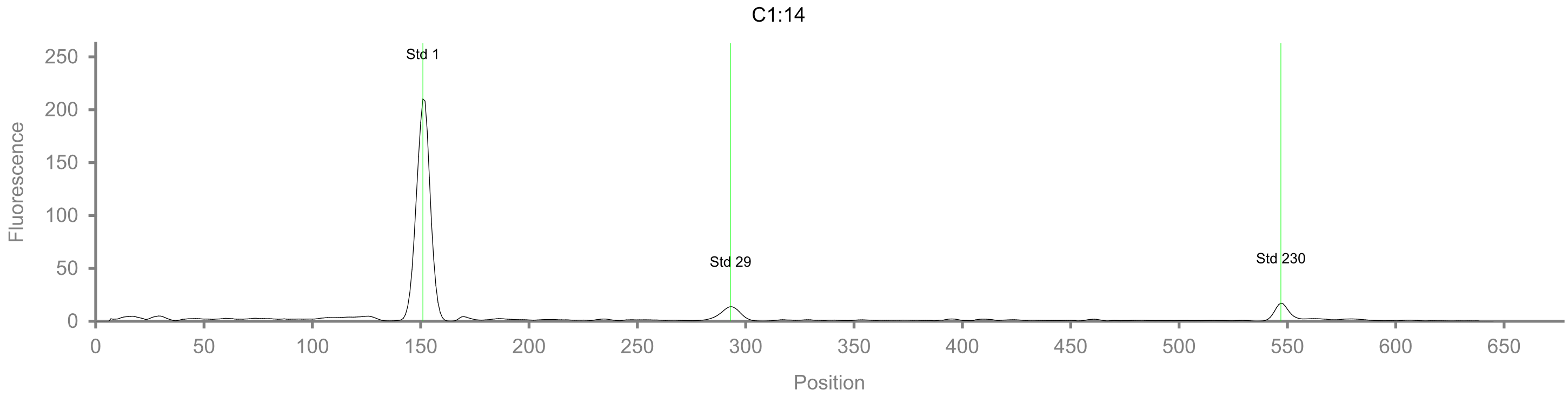


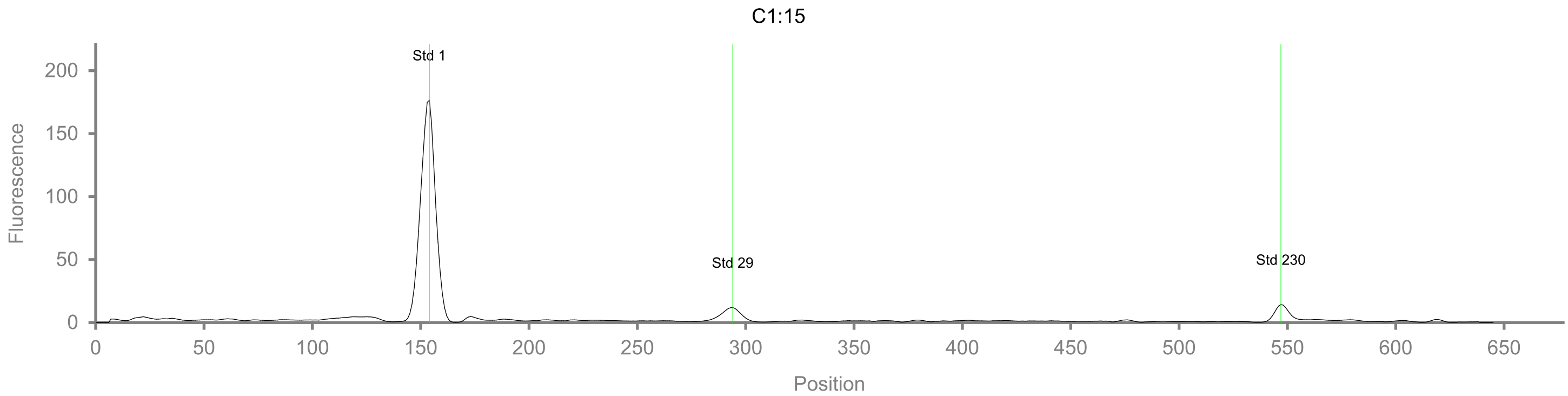


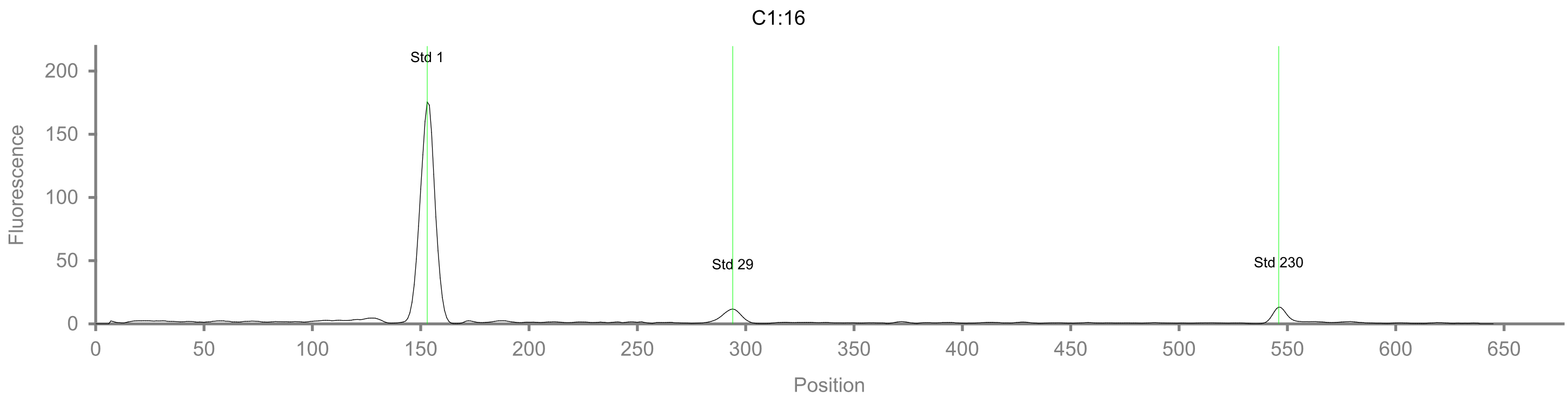

# File: Stat1_Jess

C1:17

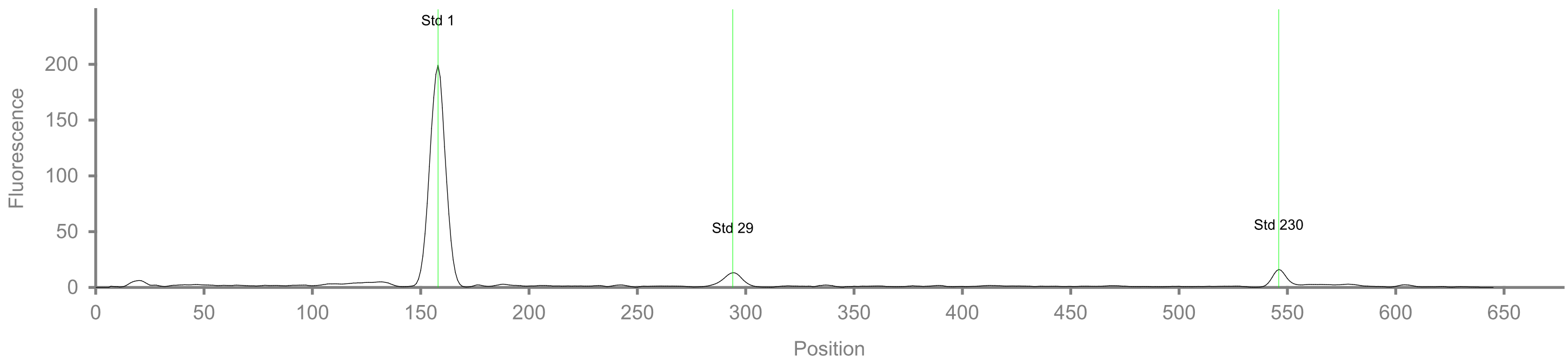


C1:18

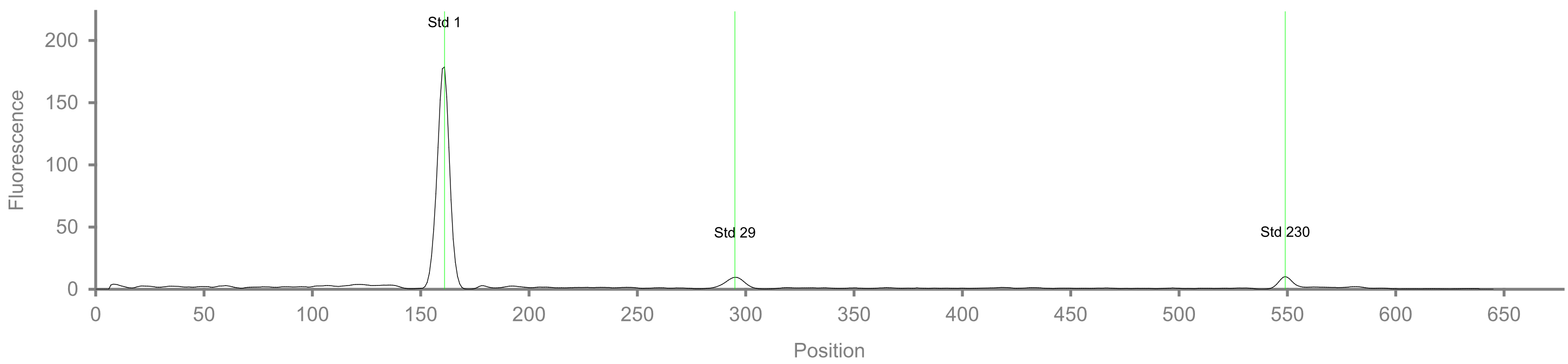


C1:19

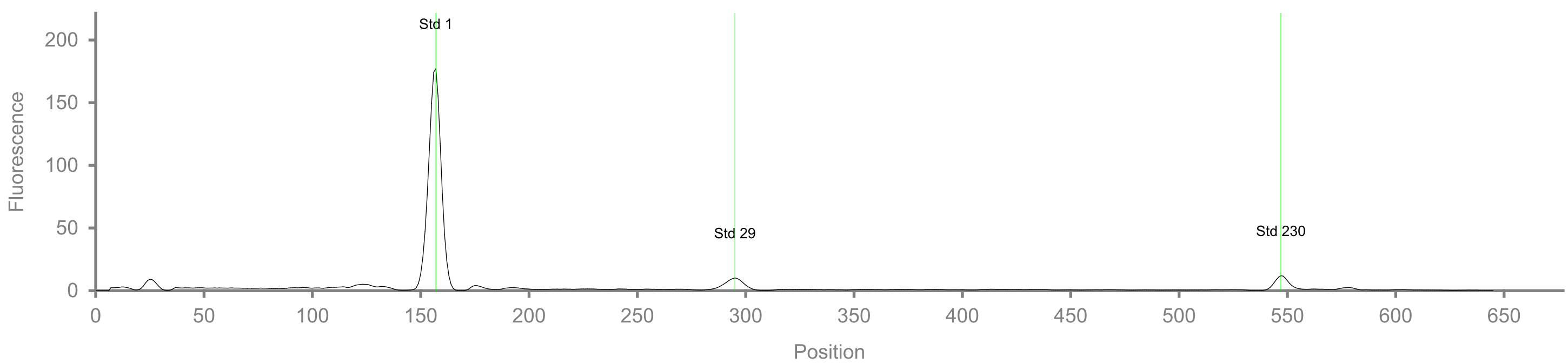


C1:20

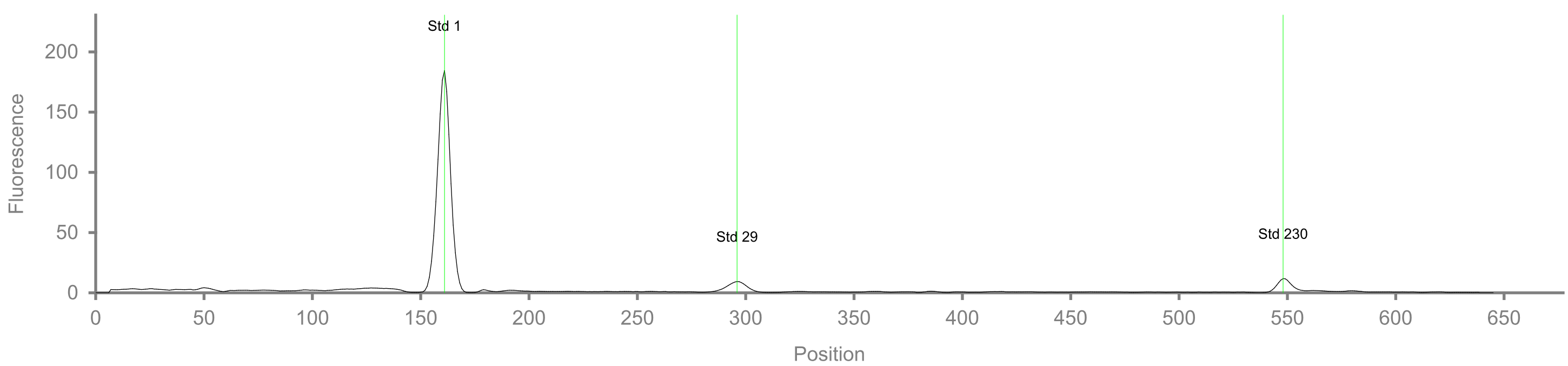

# File: Stat1_Jess

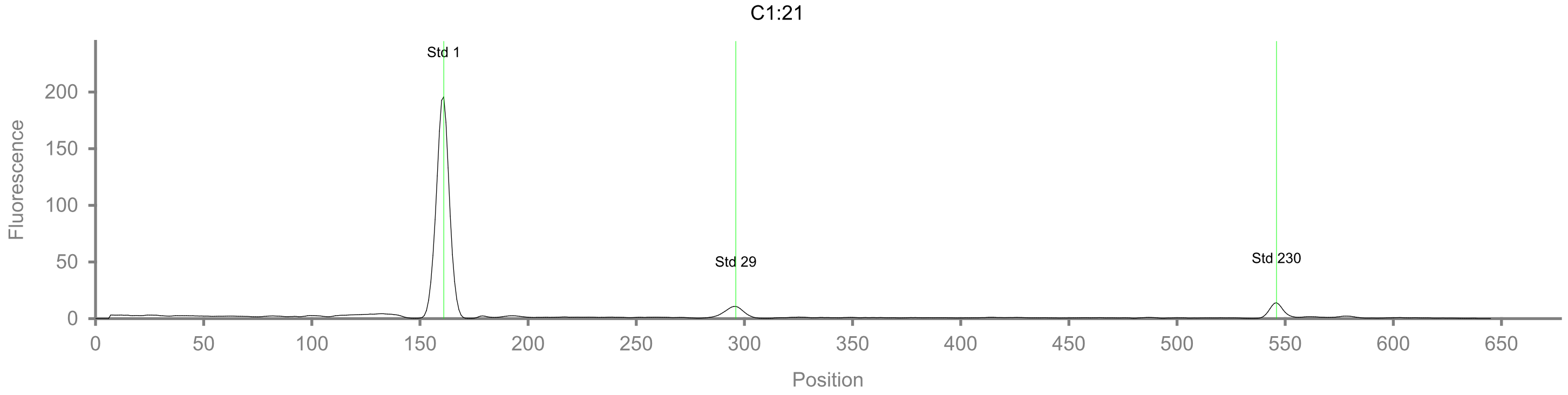


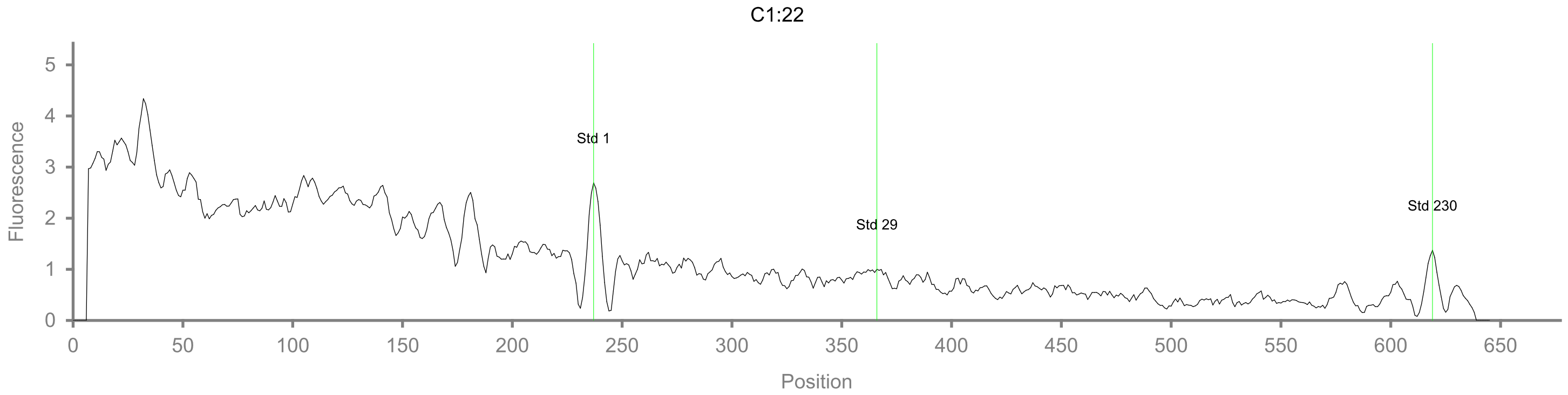


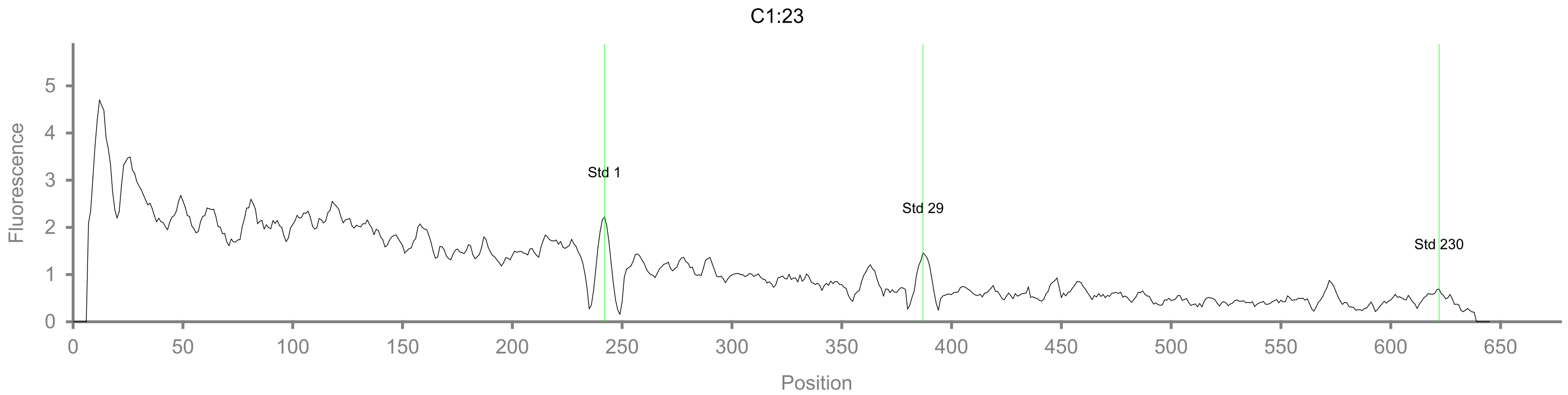


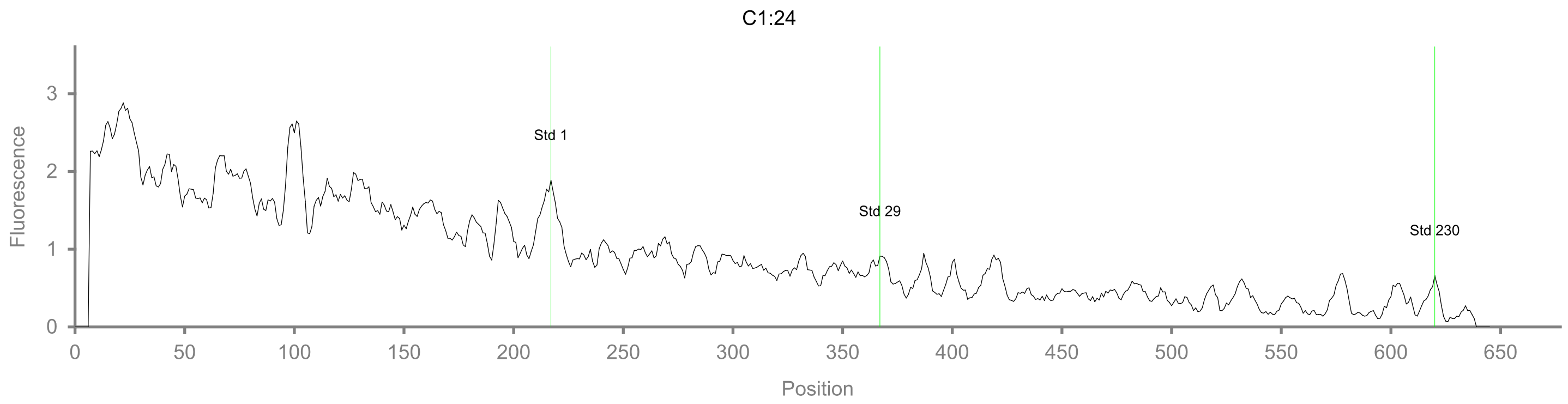

File: Stat1_Jess

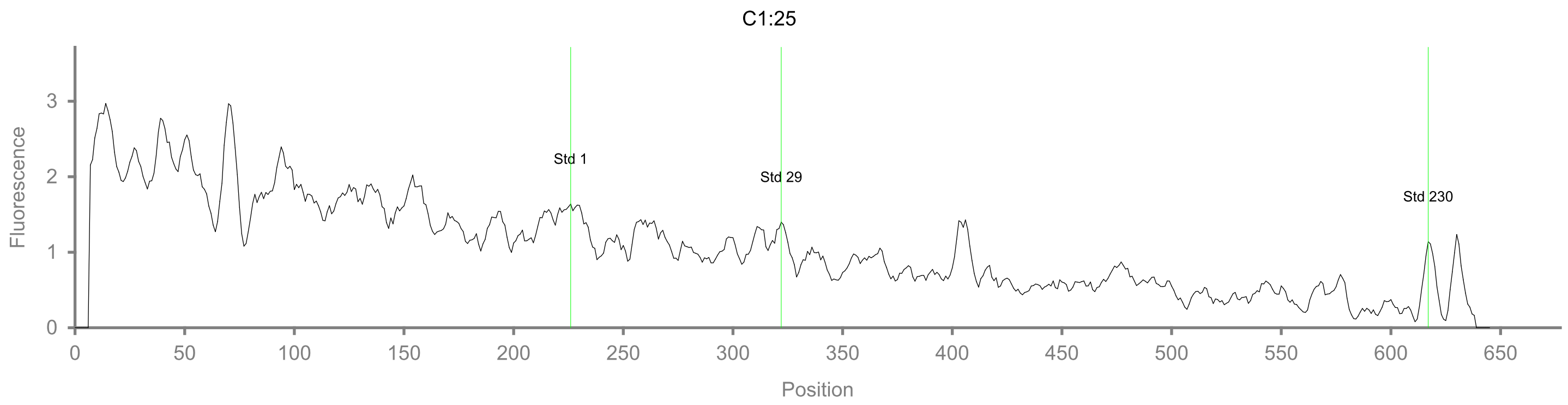

File: Stat1_Jess

## Standard Peaks (pixels)

| | | | | Std 1 | Std 29 | Std 230 |
|---|---|---|---|---|---|---|
| Biot. Ladder | Antibody Diluent | Streptavidin HRP | C1:1 | 158.1 | 305.0 | 550.0 |
| Y1:Actin | Actin | Secondary | C1:2 | 163.1 | 298.0 | 549.1 |
| Y2:Actin | Actin | Secondary | C1:3 | 161.2 | 296.9 | 548.1 |
| Y3:Actin | Actin | Secondary | C1:4 | 157.8 | 296.9 | 550.2 |
| Y4:Actin | Actin | Secondary | C1:5 | 155.1 | 294.9 | 548.2 |
| Y5:Actin | Actin | Secondary | C1:6 | 155.7 | 294.9 | 548.2 |
| O1:Actin | Actin | Secondary | C1:7 | 154.8 | 295.8 | 550.3 |
| O2:Actin | Actin | Secondary | C1:8 | 157.8 | 295.8 | 549.3 |
| O3:Actin | Actin | Secondary | C1:9 | 155.8 | 295.8 | 550.2 |
| O4:Actin | Actin | Secondary | C1:10 | 159.5 | 293.9 | 549.3 |
| O5:Actin | Actin | Secondary | C1:11 | 155.7 | 293.8 | 549.2 |
| Y1:Stat1 | Stat1 | Secondary | C1:12 | 149.4 | 293.7 | 548.3 |
| Y2:Stat1 | Stat1 | Secondary | C1:13 | 147.2 | 292.8 | 548.3 |
| Y3:Stat1 | Stat1 | Secondary | C1:14 | 150.7 | 292.9 | 547.3 |
| Y4:Stat1 | Stat1 | Secondary | C1:15 | 153.3 | 293.8 | 547.3 |
| Y5:Stat1 | Stat1 | Secondary | C1:16 | 152.8 | 293.8 | 546.2 |
| O1:Stat1 | Stat1 | Secondary | C1:17 | 157.7 | 293.9 | 546.3 |
| O2:Stat1 | Stat1 | Secondary | C1:18 | 160.5 | 294.9 | 549.2 |
| O3:Stat1 | Stat1 | Secondary | C1:19 | 156.5 | 294.9 | 547.2 |
| O4:Stat1 | Stat1 | Secondary | C1:20 | 160.7 | 295.9 | 548.3 |
| O5:Stat1 | Stat1 | Secondary | C1:21 | 160.7 | 295.8 | 546.1 |
| Sample | Primary | Secondary | C1:22 | 237.0 | 366.0 | 619.0 |
| Sample | Primary | Secondary | C1:23 | 242.0 | 387.0 | 622.0 |
| Sample | Primary | Secondary | C1:24 | 217.0 | 367.0 | 620.0 |
| Sample | Primary | Secondary | C1:25 | 226.0 | 322.0 | 617.0 |

File: Stat1_Jess

## Sample Images

CHEMI, 1s, Sample

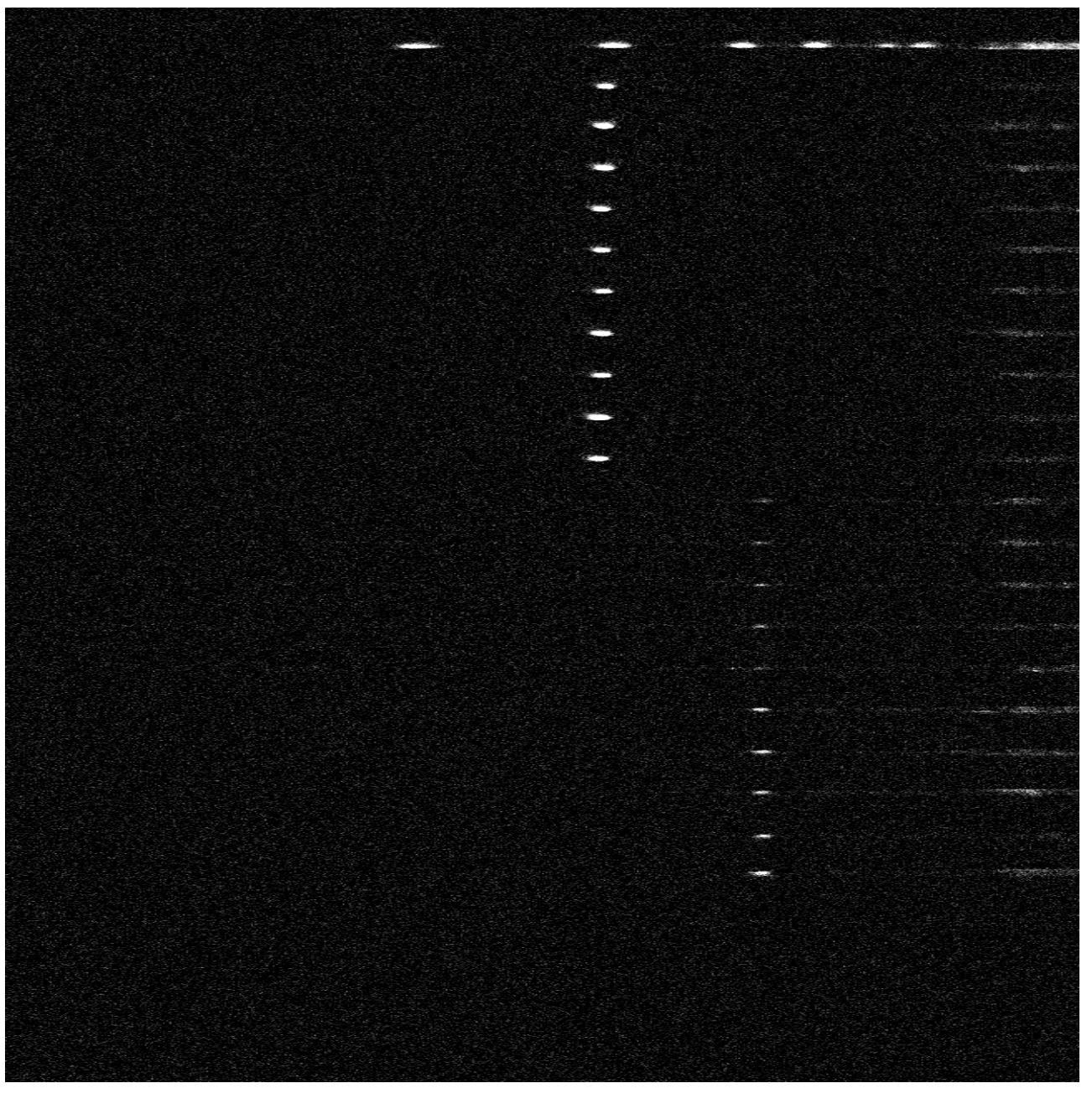

File: Stat1_Jess

## Standard Image

Std, 4s

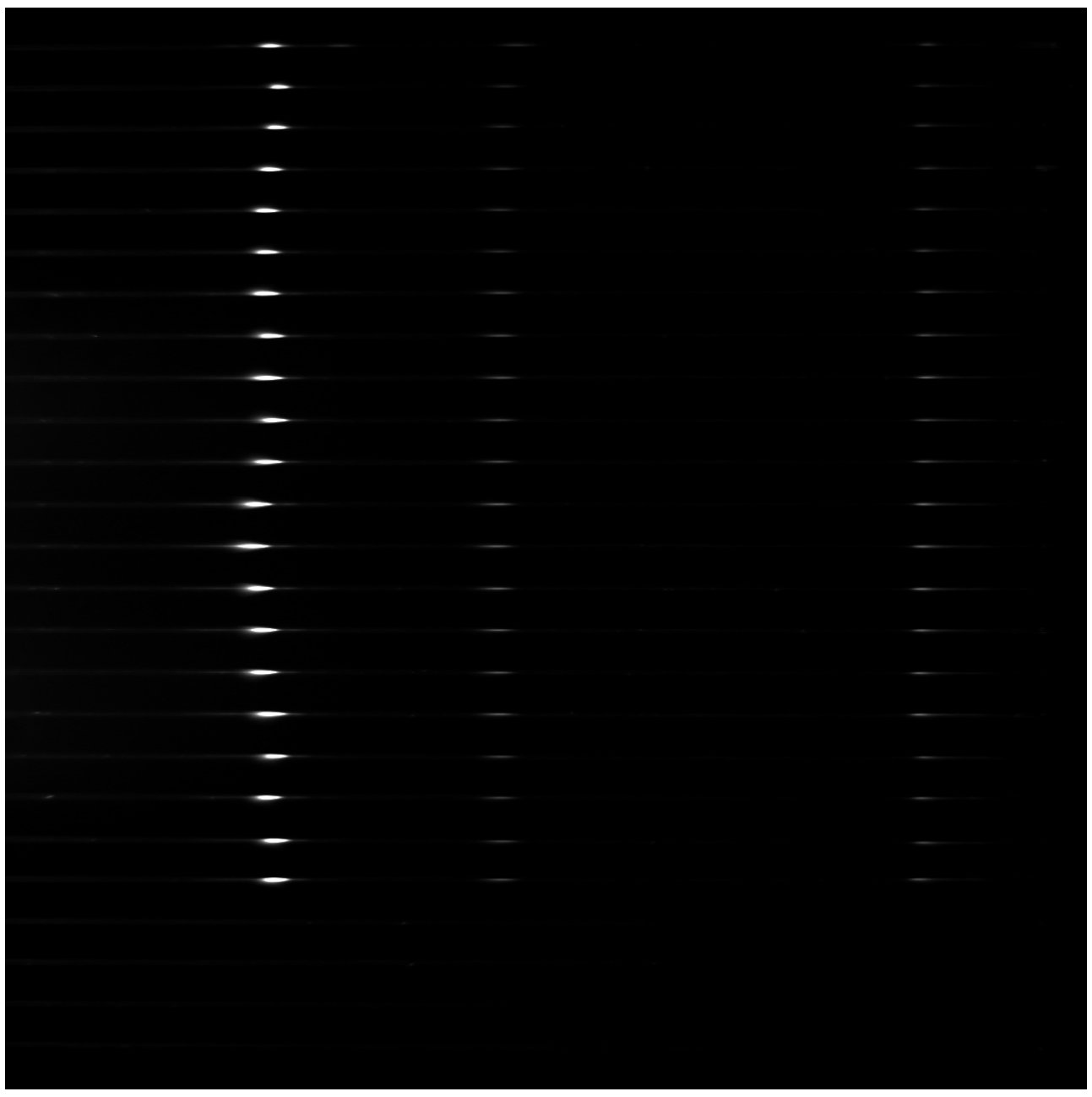

File: Stat1_Jess

# Analysis Log

| | | | |
|---|---|---|---|
| 2026-07-14 15:52:46 | | Started run: 2026-07-14_15-57-16_Jess  Assay: Jess.assay from Compass for SW v6.3.0-0719 | |
| 2026-07-15 09:41:09 | | Saved template changes from Compass for SW v6.3.0-0719 | |
| | | Changed well annotation at row A column 2: Name from Sample to Y1:Actin | |
| | | Changed well annotation at row A column 3: Name from Sample to Y2:Actin | |
| | | Changed well annotation at row A column 4: Name from Sample to Y3:Actin | |
| | | Changed well annotation at row A column 5: Name from Sample to Y4:Actin | |
| | | Changed well annotation at row A column 6: Name from Sample to Y5:Actin | |
| | | Changed well annotation at row A column 7: Name from Sample to O1:Actin | |
| | | Changed well annotation at row A column 8: Name from Sample to O2:Actin | |
| | | Changed well annotation at row A column 9: Name from Sample to O3:Actin | |
| | | Changed well annotation at row A column 10: Name from Sample to O4:Actin | |
| | | Changed well annotation at row A column 11: Name from Sample to O5:Actin | |
| | | Changed well annotation at row A column 12: Name from Sample to Y1:Stat1 | |
| | | Changed well annotation at row A column 13: Name from Sample to Y2:Stat1 | |
| | | Changed well annotation at row A column 14: Name from Sample to Y3:Stat1 | |
| | | Changed well annotation at row A column 15: Name from Sample to Y4:Stat1 | |
| | | Changed well annotation at row A column 16: Name from Sample to Y5:Stat1 | |
| | | Changed well annotation at row A column 17: Name from Sample to O1:Stat1 | |
| | | Changed well annotation at row A column 18: Name from Sample to O2:Stat1 | |
| | | Changed well annotation at row A column 19: Name from Sample to O3:Stat1 | |
| | | Changed well annotation at row A column 20: Name from Sample to O4:Stat1 | |
| | | Changed well annotation at row A column 21: Name from Sample to O5:Stat1 | |
| | | Changed well annotation at row C column 2: Name from Primary to Actin | |

File: Stat1_Jess

| | | | |
|---|---|---|---|
| | | Changed well annotation at row C column 3: Name from Primary to Actin | |
| | | Changed well annotation at row C column 4: Name from Primary to Actin | |
| | | Changed well annotation at row C column 5: Name from Primary to Actin | |
| | | Changed well annotation at row C column 6: Name from Primary to Actin | |
| | | Changed well annotation at row C column 7: Name from Primary to Actin | |
| | | Changed well annotation at row C column 8: Name from Primary to Actin | |
| | | Changed well annotation at row C column 9: Name from Primary to Actin | |
| | | Changed well annotation at row C column 10: Name from Primary to Actin | |
| | | Changed well annotation at row C column 11: Name from Primary to Actin | |
| | | Changed well annotation at row C column 12: Name from Primary to Stat1 | |
| | | Changed well annotation at row C column 13: Name from Primary to Stat1 | |
| | | Changed well annotation at row C column 14: Name from Primary to Stat1 | |
| | | Changed well annotation at row C column 15: Name from Primary to Stat1 | |
| | | Changed well annotation at row C column 16: Name from Primary to Stat1 | |
| | | Changed well annotation at row C column 17: Name from Primary to Stat1 | |
| | | Changed well annotation at row C column 18: Name from Primary to Stat1 | |
| | | Changed well annotation at row C column 19: Name from Primary to Stat1 | |
| | | Changed well annotation at row C column 20: Name from Primary to Stat1 | |
| | | Changed well annotation at row C column 21: Name from Primary to Stat1 | |
| 2026-07-15 09:44:17 | | Saved analysis and template changes from Compass for SW v6.3.0-0719 | |
| | | Added Peak Fit Apply Override "apply fit to Biot. Ladder" | |
| 2026-07-15 09:47:10 | | Saved analysis changes from Compass for SW v6.3.0-0719 | |
| | | Hide peak at position 445 in manual settings for capillary 2 CHEMI | |
| | | Hide peak at position 551 in manual settings for capillary 2 CHEMI | |
| | | Hide peak at position 419 in manual settings for capillary 4 CHEMI | |
| | | Hide peak at position 450 in manual settings for capillary 4 CHEMI | |
| | | Hide peak at position 472 in manual settings for capillary 5 CHEMI | |
| | | Hide peak at position 481 in manual settings for capillary 6 CHEMI | |
| | | Hide peak at position 531 in manual settings for capillary 6 CHEMI | |
| | | Hide peak at position 547 in manual settings for capillary 6 CHEMI | |
| | | Hide peak at position 514 in manual settings for capillary 8 CHEMI | |

File: Stat1_Jess

| | | | |
|---|---|---|---|
| | | Hide peak at position 463 in manual settings for capillary 10 CHEMI | |
| | | Hide peak at position 487 in manual settings for capillary 10 CHEMI | |
| | | Hide peak at position 535 in manual settings for capillary 10 CHEMI | |
| | | Hide peak at position 546 in manual settings for capillary 10 CHEMI | |
| | | Hide peak at position 544 in manual settings for capillary 11 CHEMI | |
| | | Hide peak at position 219 in manual settings for capillary 25 CHEMI | |
| | | Hide peak at position 528 in manual settings for capillary 25 CHEMI | |



proteinsimple a biotechne brand

File: Psmb9_Jess

# Run

| | |
|---|---|
| | 2026-07-14_23-53-50_Jess |
| | C:\Users\\OneDrive\Desktop |
| | Jess |
| | Regular: 12-230 kDa |
| | |
| | Jess : Jess JS5549 - js5549 |
| | 2.1.2021.09.01.00.26.14.bc9a11c63 |
| | |
| | 1144502151 |
| | 2026067640901026 |
| | 6-2026 |
| | 11:49  7 14, 2026 CST |
| | 2:34  7 15, 2026 CST |
| | None |

File: Psmb9_Jess

# Protocol

| | |
|---|---|
| Separation Matrix | |
| Well Row | |
| Load Time (sec) | |
| Stacking Matrix | |
| Well Row | |
| Load Time (sec) | |
| Sample | |
| Well Row | |
| Load Time (sec) | |
| Separation Time (min) | |
| Separation Voltage (volts) | |
| Standards Exposure (sec) | |
| EE Immobilization Time (sec) | |
| Antibody Diluent Time (min) | |
| Well Row | |
| Primary Antibody Time (min) | |
| Well Row | |
| Secondary Antibody Time (min) | |
| Well Row | |
| Detection | |
| Well Row | |
| CHEMI Detection Profile | |
| Exposure 1 (sec) | |
| Exposure 2 (sec) | |
| Exposure 3 (sec) | |
| Exposure 4 (sec) | |
| Exposure 5 (sec) | |
| Exposure 6 (sec) | |
| Exposure 7 (sec) | |
| Exposure 8 (sec) | |
| Exposure 9 (sec) | |
| Ladder Channel | |



proteinsimple a biotechne brand

File: Psmb9_Jess

## Plate Layout

| | 1 | 2 | 3 | 4 | 5 | 6 | 7 | 8 | 9 | 10 | 11 | 12 | 13 | 14 | 15 | 16 | 17 | 18 | 19 | 20 | 21 | 22 | 23 | 24 | 25 |
|---|---|---|---|---|---|---|---|---|---|---|---|---|---|---|---|---|---|---|---|---|---|---|---|---|---|
| A | B... | Y... | Y... | Y... | Y... | Y... | O... | O... | O... | O... | O... | Y... | Y... | Y... | Y... | Y... | O... | O... | O... | O... | O... | Sample | | | |
| B | Antibody Diluent | | | | | | | | | | | | | | | | | | | | | | | | |
| C | A... | Actin | | | | | | | | | | Psmb9 | | | | | | | | | | Primary | | | |
| D | S... | Secondary | | | | | | | | | | | | | | | | | | | | | | | |
| E | Luminol/Peroxide | | | | | | | | | | | | | | | | | | | | | | | | |

File: Psmb9_Jess

## Analysis Settings

| | |
|---|---|
| Hidden Capillaries | None |
| Images | |
| CHEMI Luminescence | High Dynamic Range 4.0 |
| Lane Contrast | Slider |
| CHEMI White Level | 16 |
| CHEMI Black Level | 9955.4 |
| Peak Fit | fit |
| Apply To | Default, Biot. Ladder |
| Range Min (MW (kDa)) | 1.0 |
| Range Max (MW (kDa)) | 250.0 |
| Range View | Analysis |
| Baseline Type | Spline |
| Baseline Threshold | 1.0 |
| Baseline Window (pixels) | 15.0 |
| Baseline Stiffness | 1.0 |
| Peak Find Threshold | 10.0 |
| Peak Find Width (pixels) | 9.0 |
| Peak Find Area Calculation | Gaussian Fit |
| Ladder Settings | Cap: 1, Chemiluminescent/Fluorescent Channel: CHEMI |
| Apply To | Default |
| Ladder Peak 1 (MW (kDa)) | 12.0 |
| Ladder Peak 2 (MW (kDa)) | 40.0 |
| Ladder Peak 3 (MW (kDa)) | 66.0 |
| Ladder Peak 4 (MW (kDa)) | 116.0 |
| Ladder Peak 5 (MW (kDa)) | 180.0 |
| Ladder Peak 6 (MW (kDa)) | 230.0 |
| Signal To Noise | |
| Algorithm | Signal-to-Noise Ratio |
| Region Spectrum | Full |
| Region Units | kDa |
| Standards | Biotinylated Ladder (12kDa-230kDa) |
| Apply To | Default |
| Standard 1 (MW (kDa)) | 1 |
| Standard 1 Position (pixels) | 120 |
| Standard 1 Fit | no |
| Standard 1 Registration | yes |
| Standard 2 (MW (kDa)) | 29 |
| Standard 2 Position (pixels) | 270 |
| Standard 2 Fit | yes |
| Standard 2 Registration | no |
| Standard 3 (MW (kDa)) | 230 |
| Standard 3 Position (pixels) | 530 |
| Standard 3 Fit | yes |
| Standard 3 Registration | no |
| Advanced | Advanced |
| Apply To | Default |
| Standards Peak Width (pixels) | 15 |
| Standards Allowable Drift (pixels) | 100 |



proteinsimple a biotechne brand

# File: Psmb9_Jess

| | |
|---|---|
| Sample Peak Fit Starting Width Ratio | 0.5 |
| Image Median Filter Threshold Ratio | 0.5 |
| Image Median Filter Threshold Limit (counts) | 1000 |

File: Psmb9_Jess

CHEMI

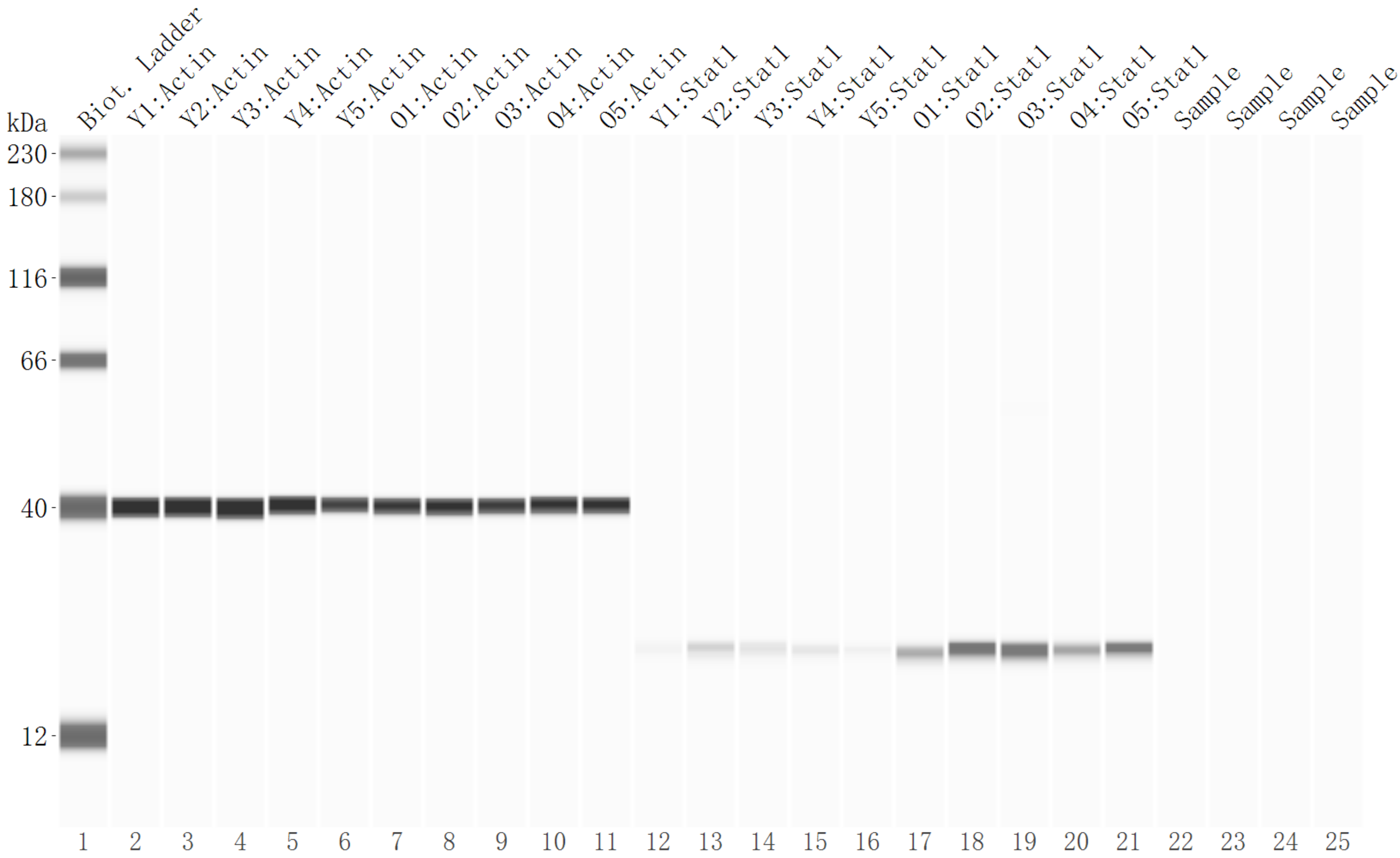


C:\Users\\OneDrive\Desktop\Psmb9_Jess.cbz

File: Psmb9_Jess

## Standard

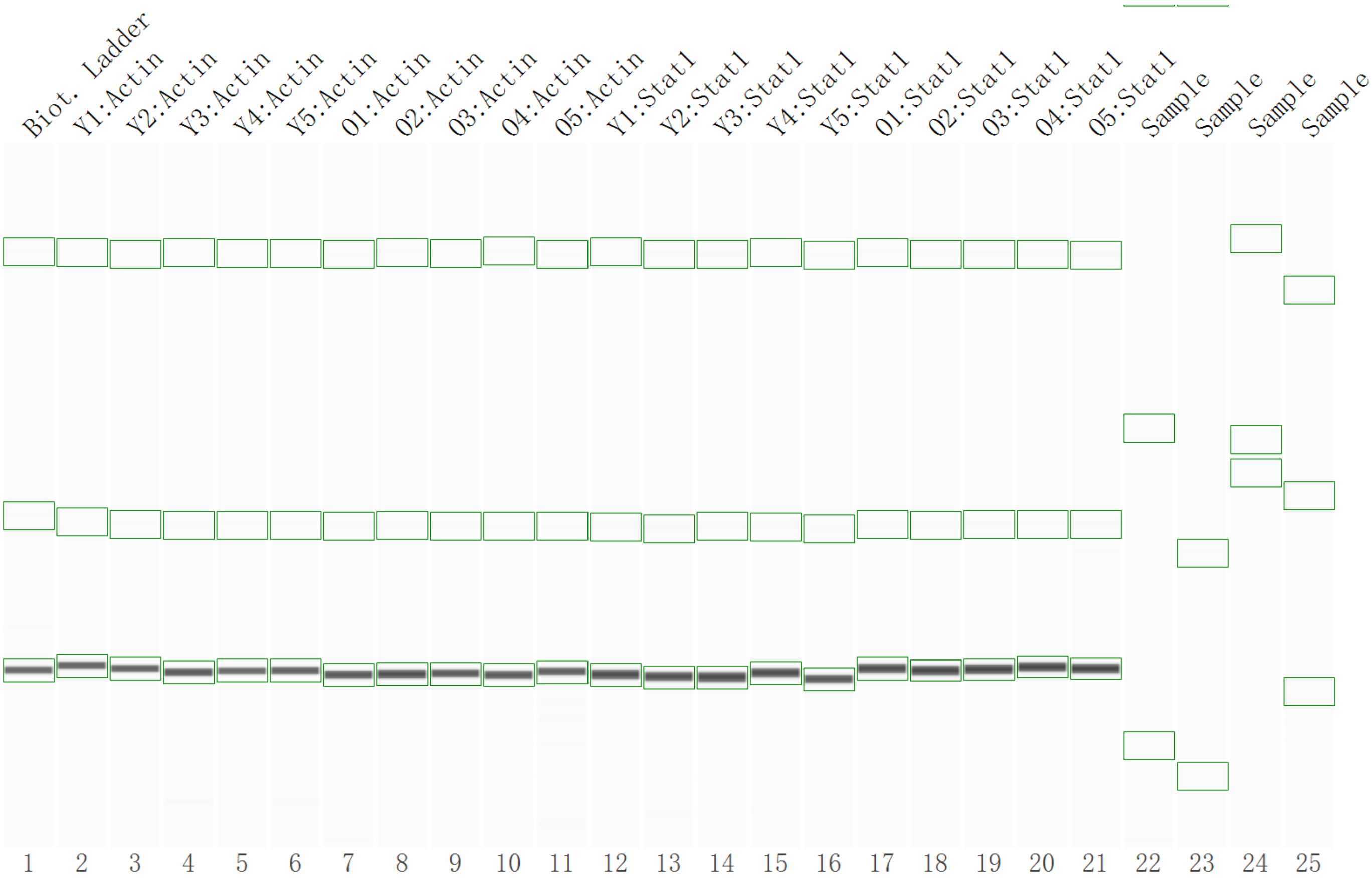

File: Psmb9_Jess

# IV Plot

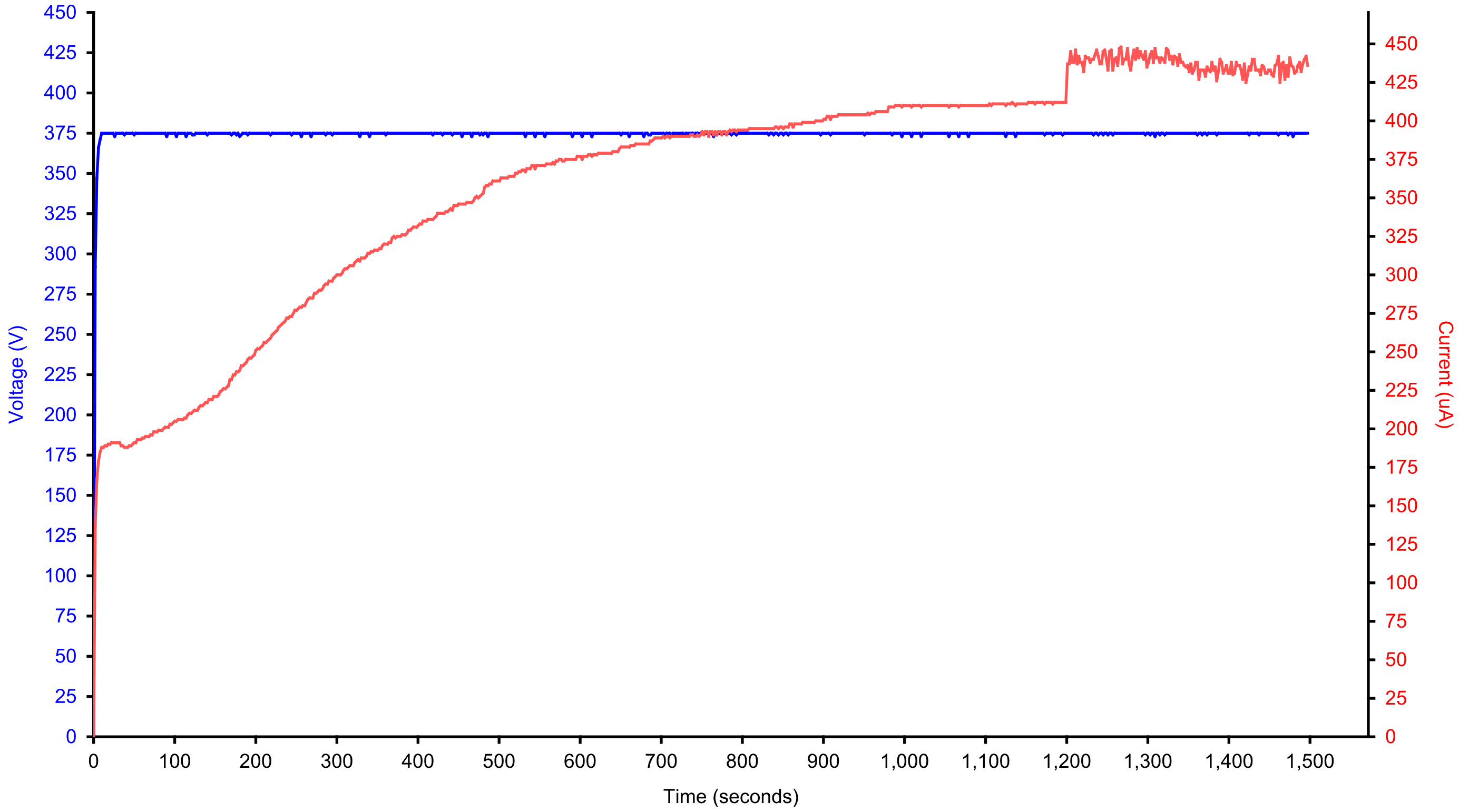

File: Psmb9_Jess

## CHEMI Sample Plots

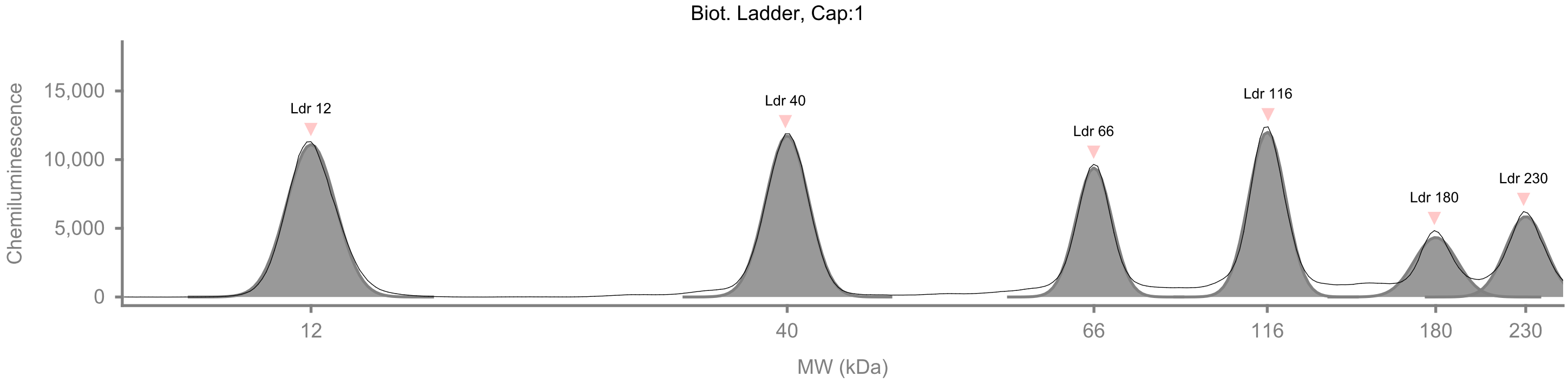


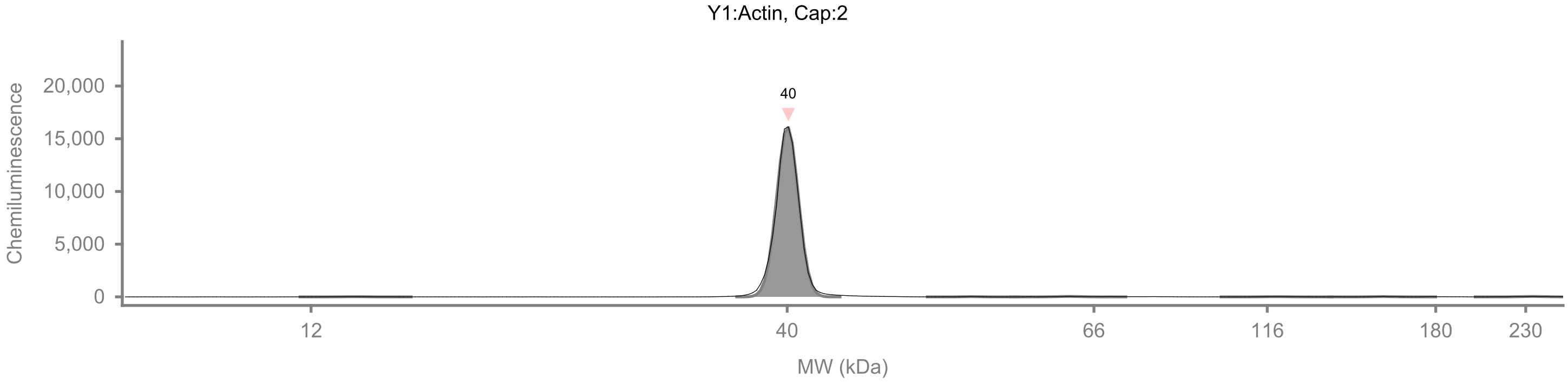


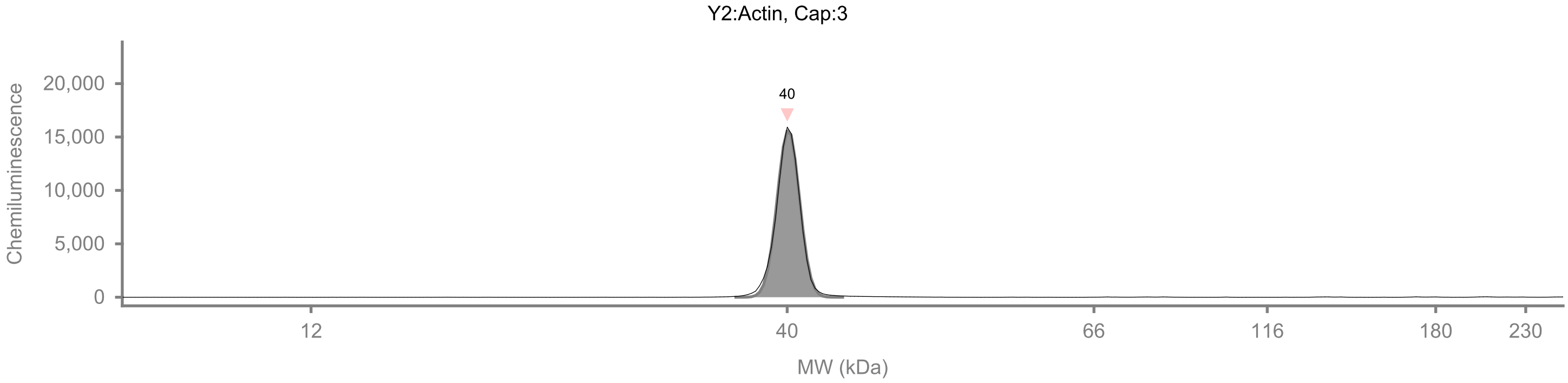


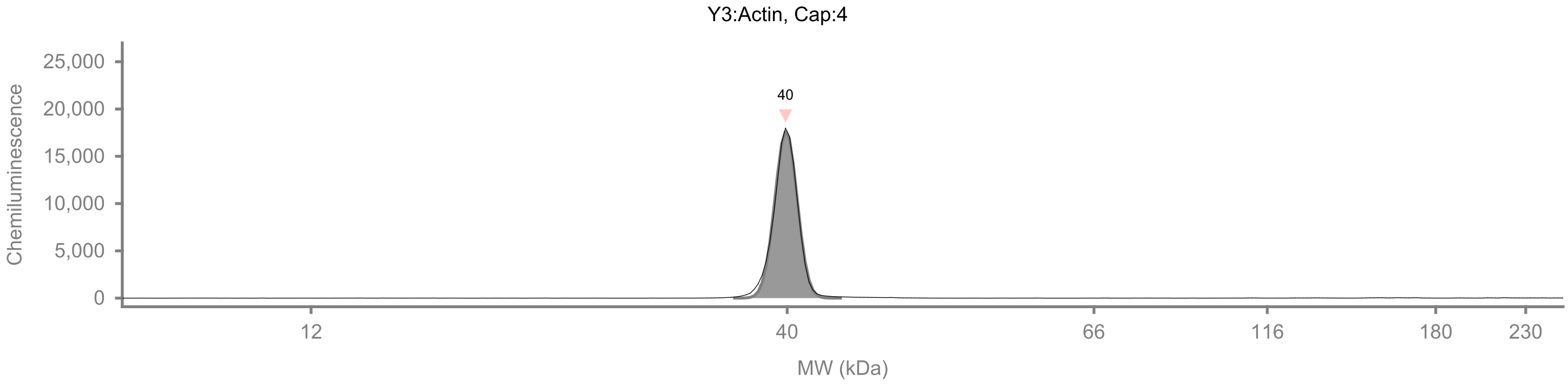

# File: Psmb9_Jess

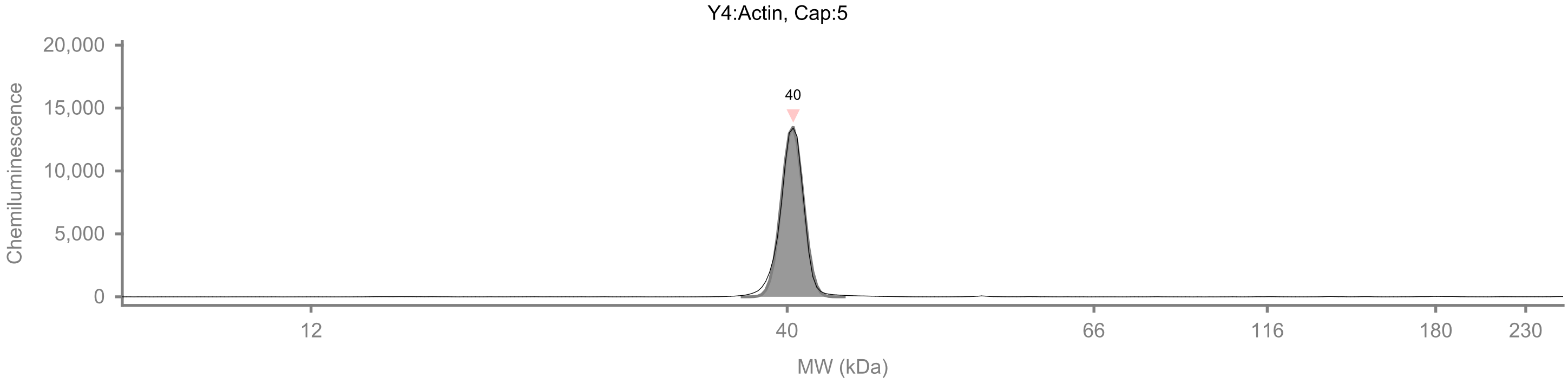


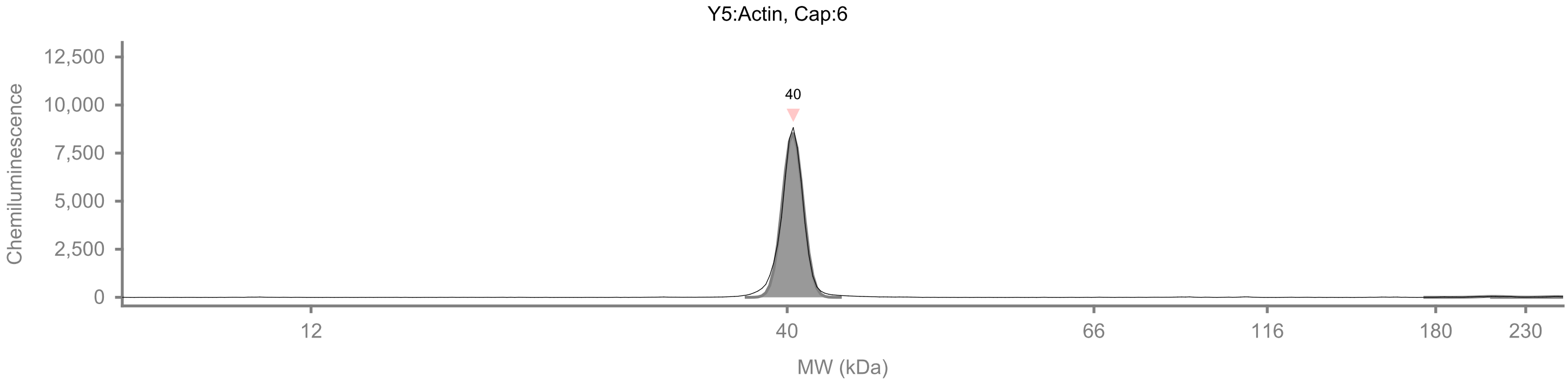


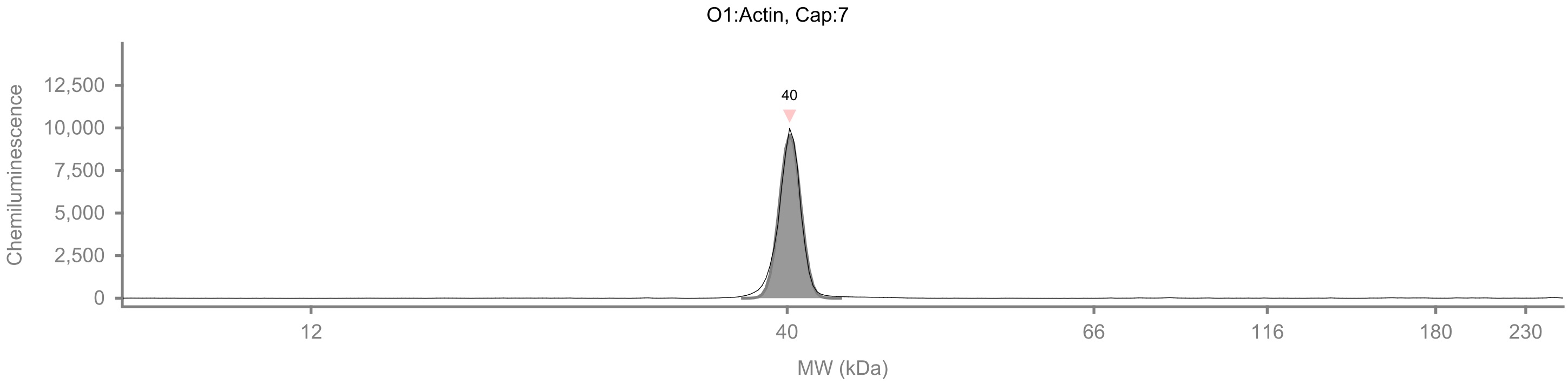


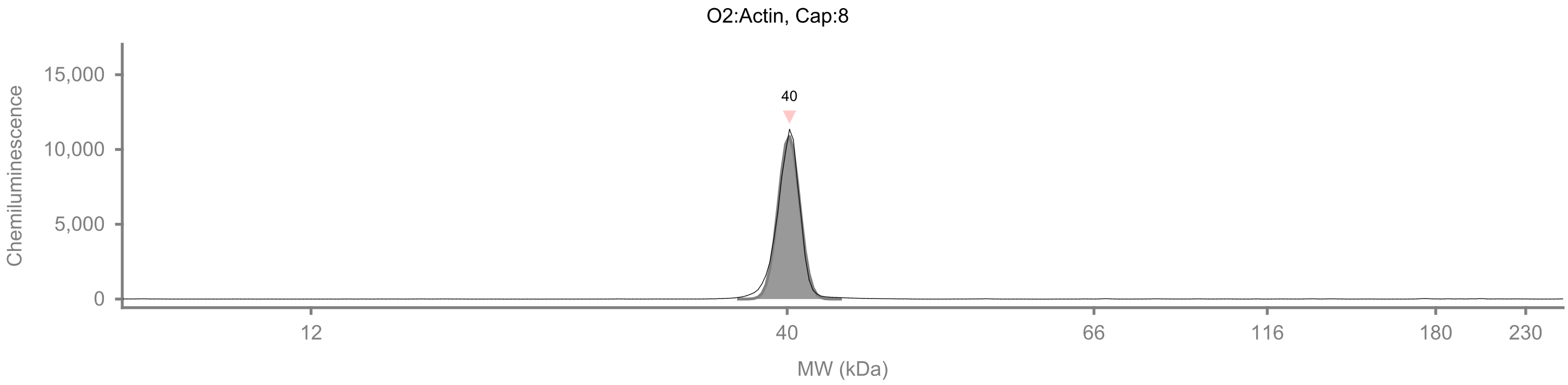




proteinsimple a biotechne brand

# File: Psmb9_Jess

O3:Actin, Cap:9

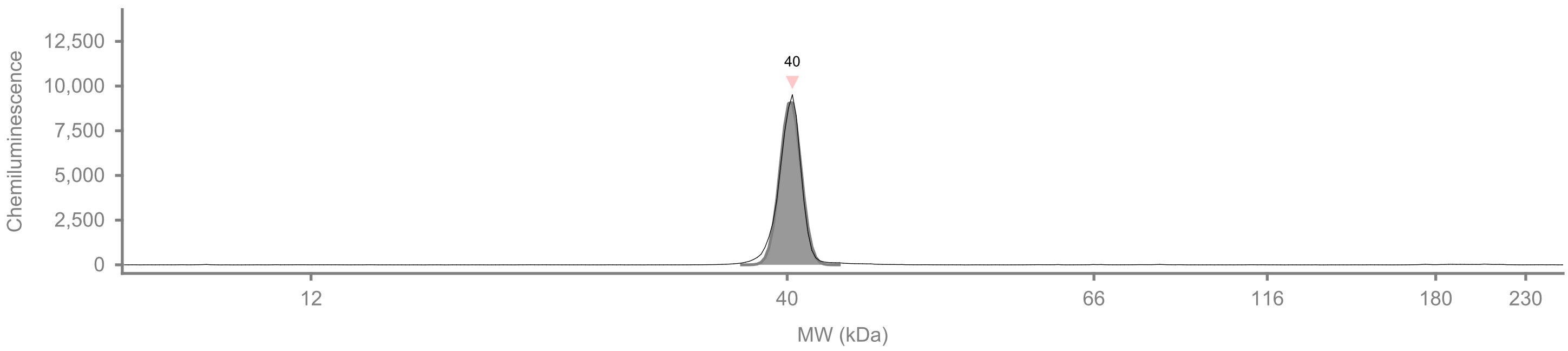


O4:Actin, Cap:10

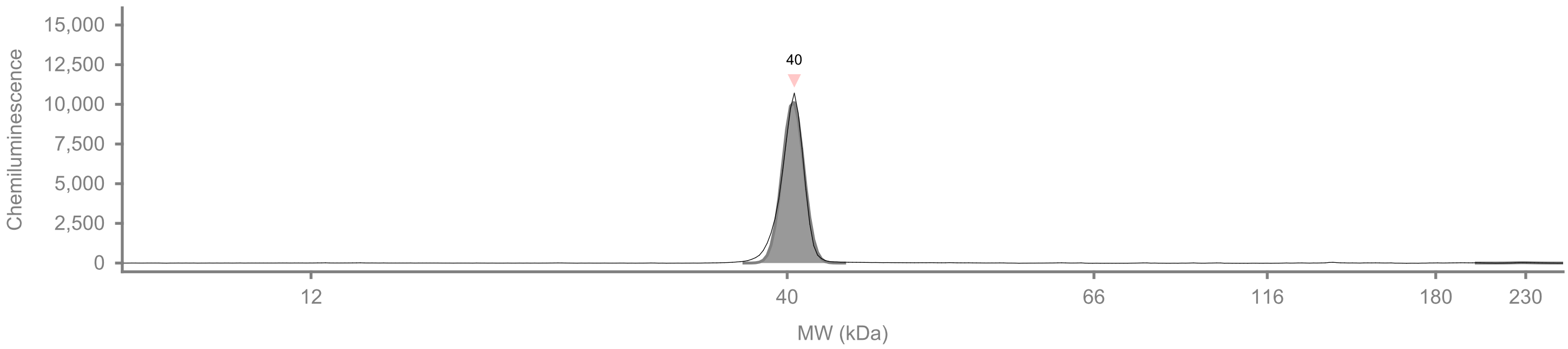


O5:Actin, Cap:11

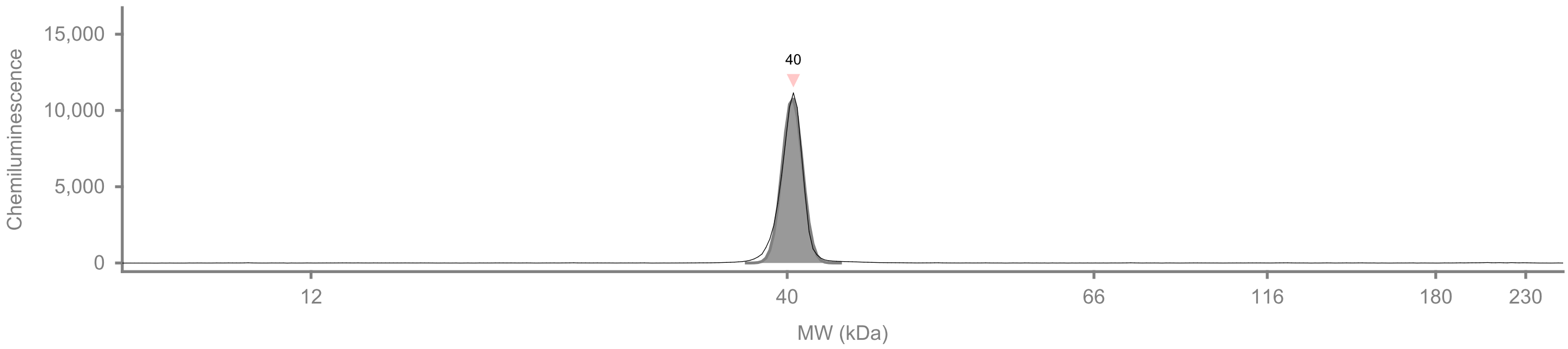


Y1:Stat1, Cap:12

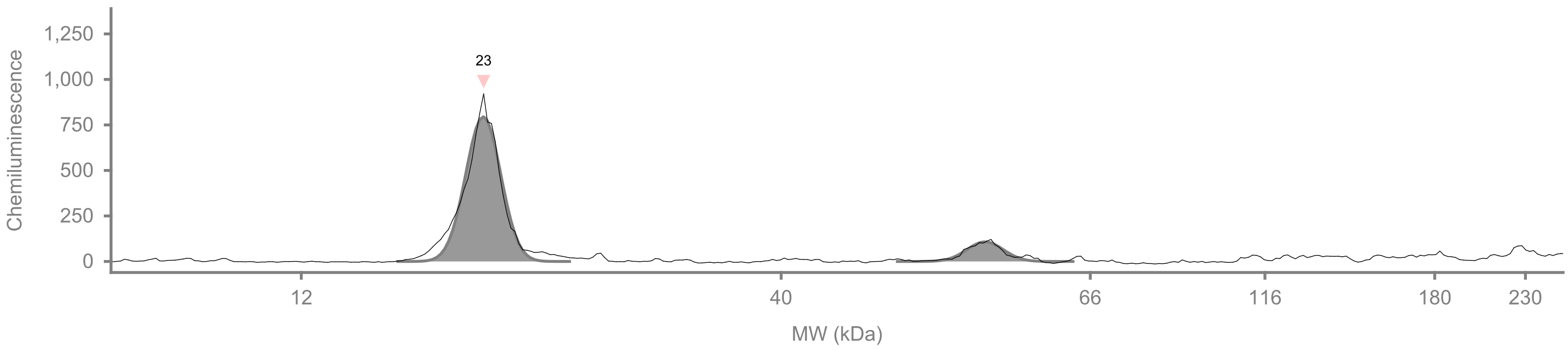

# File: Psmb9_Jess

Y2:Stat1, Cap:13

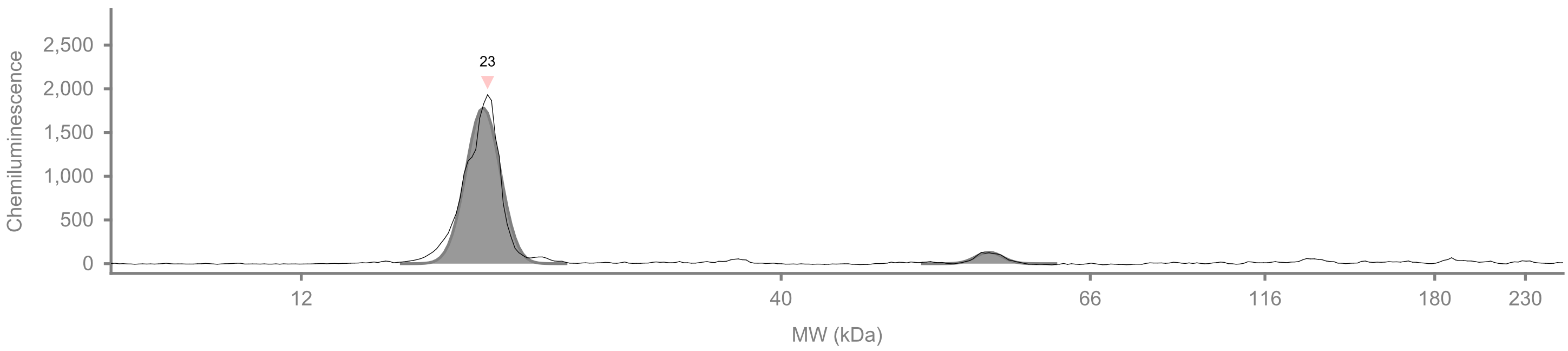


Y3:Stat1, Cap:14

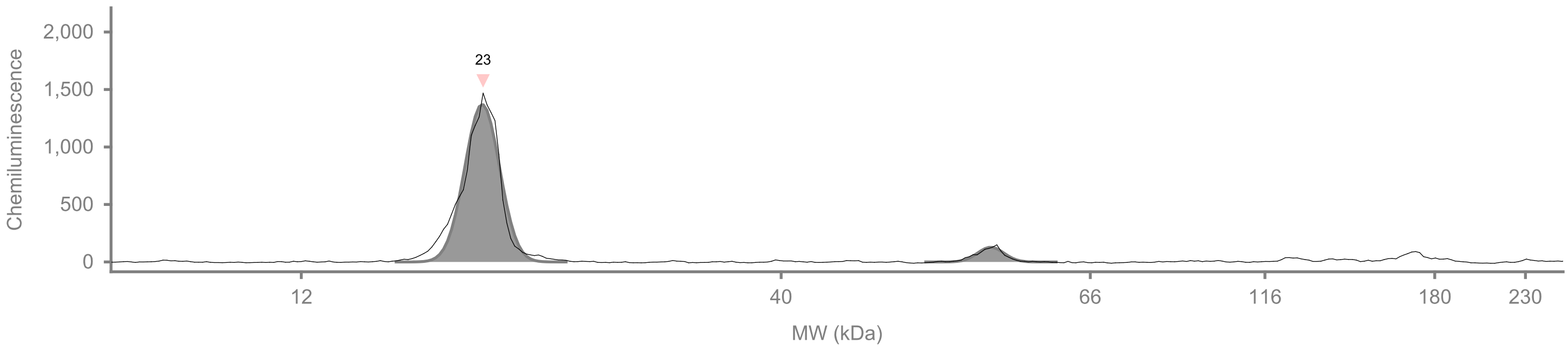


Y4:Stat1, Cap:15

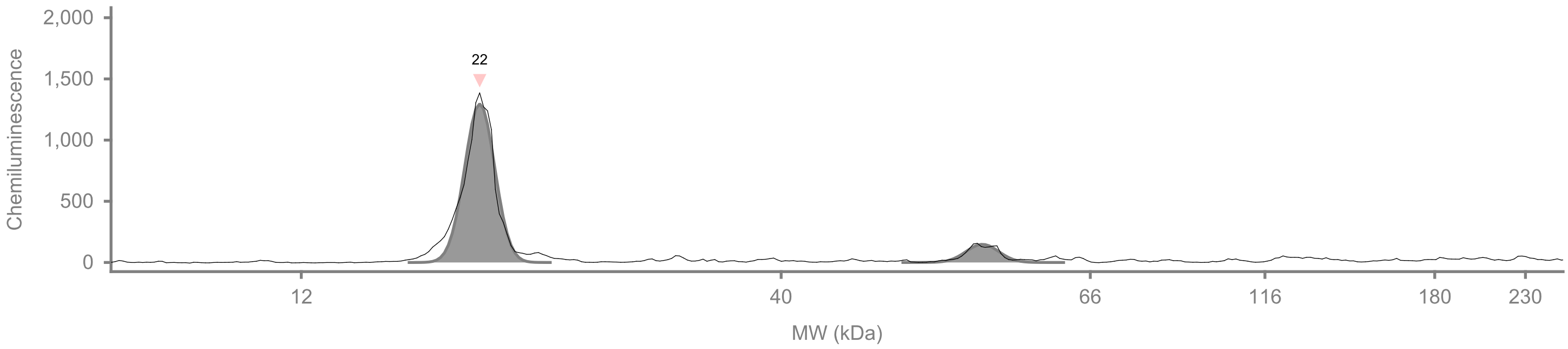


Y5:Stat1, Cap:16

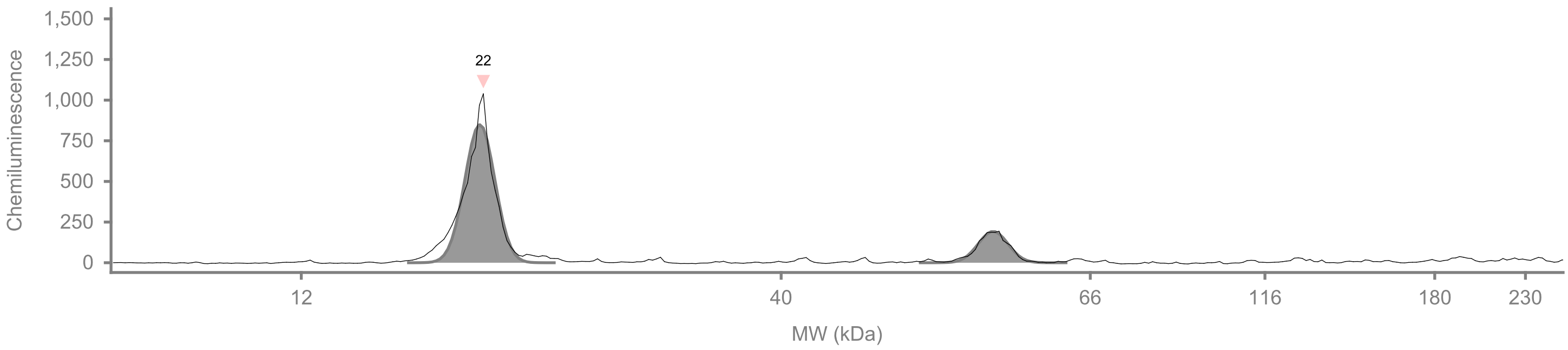




proteinsimple a biotechne brand

# File: Psmb9_Jess

O1:Stat1, Cap:17

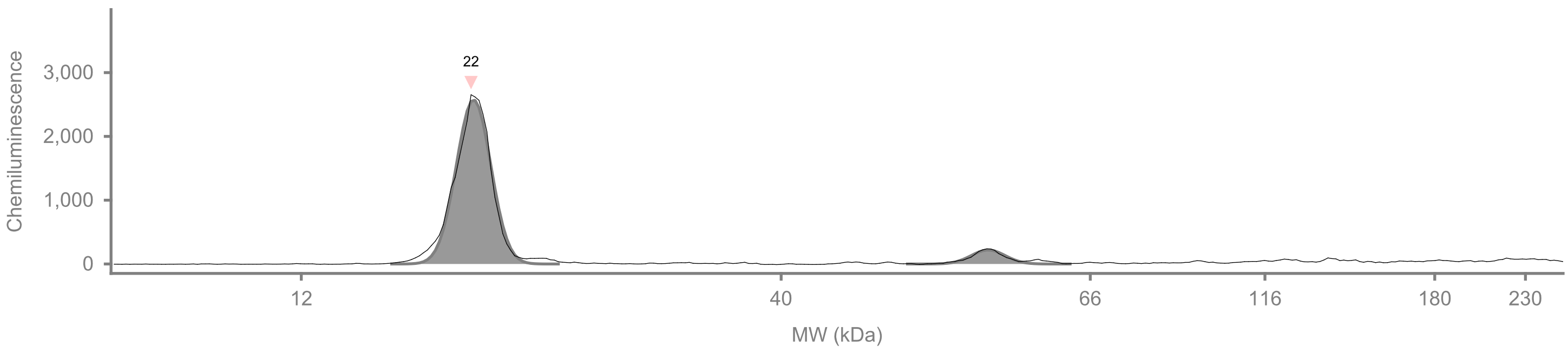


O2:Stat1, Cap:18

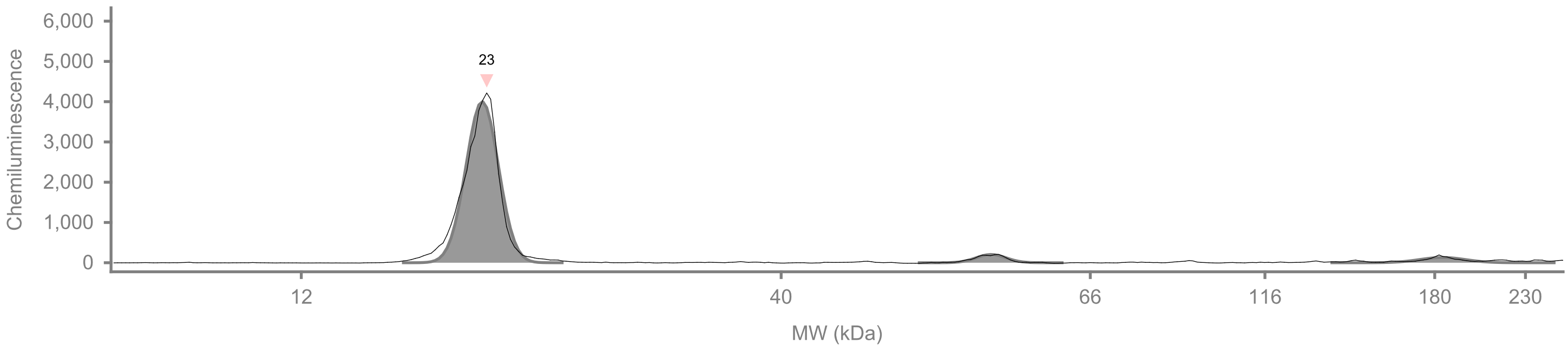


O3:Stat1, Cap:19

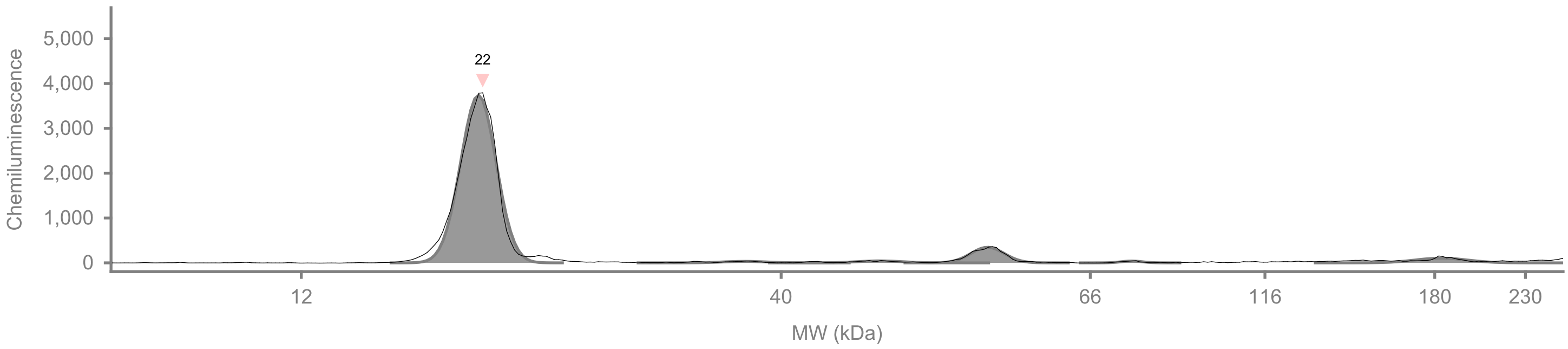


O4:Stat1, Cap:20

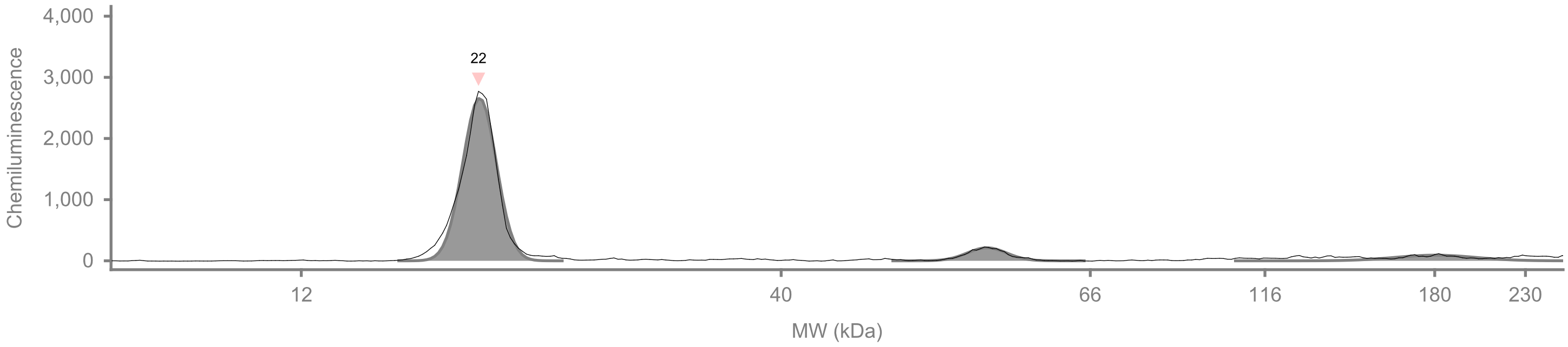

# File: Psmb9_Jess

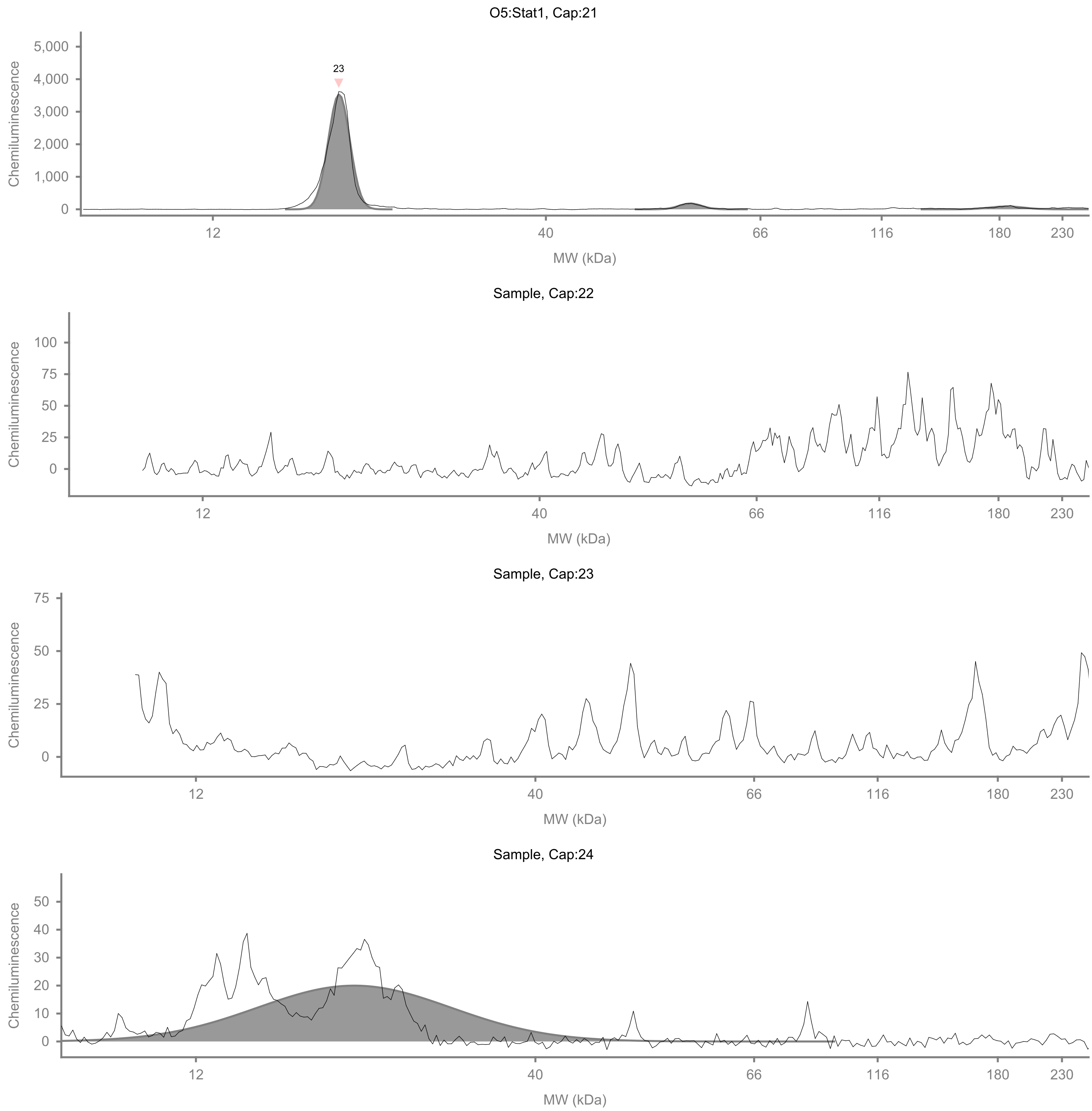


C:\Users\\OneDrive\Desktop\Psmb9_Jess.cbz

# File: Psmb9_Jess

Sample, Cap:25

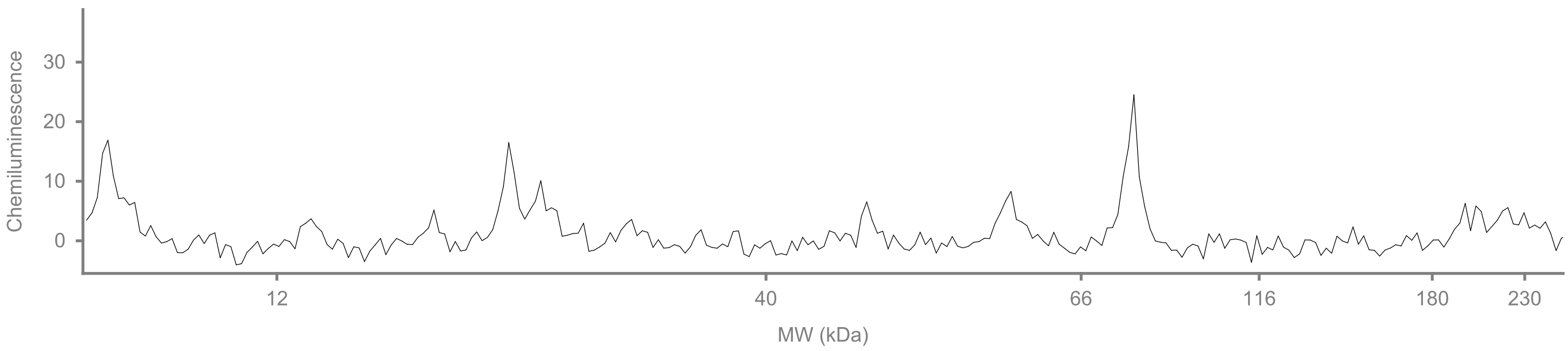




proteinsimple a biotechne brand

File: Psmb9_Jess

## CHEMI Sample Peaks

| | | | | | | | | | | | | | |
|---|---|---|---|---|---|---|---|---|---|---|---|---|---|
| Biot. Ladder | Antibody Diluent | Streptavidin HRP | 1 | 1 | Ldr 12 | 248 | 12 | 11098.3 | 168783.9 | | 14.3 | 3888.3 | 67.6 |
| Biot. Ladder | Antibody Diluent | Streptavidin HRP | 1 | 2 | Ldr 40 | 366 | 40 | 11751.2 | 155641.3 | | 12.4 | 4117.1 | 90.4 |
| Biot. Ladder | Antibody Diluent | Streptavidin HRP | 1 | 3 | Ldr 66 | 441 | 66 | 9405.2 | 103876.9 | | 10.4 | 3295.1 | 92.8 |
| Biot. Ladder | Antibody Diluent | Streptavidin HRP | 1 | 4 | Ldr 116 | 484 | 116 | 12015.3 | 137426.0 | | 10.7 | 4209.6 | 90.8 |
| Biot. Ladder | Antibody Diluent | Streptavidin HRP | 1 | 5 | Ldr 180 | 525 | 180 | 4334.8 | 57610.8 | | 12.5 | 1518.7 | 87.0 |
| Biot. Ladder | Antibody Diluent | Streptavidin HRP | 1 | 6 | Ldr 230 | 548 | 230 | 5857.7 | 73120.5 | | 11.7 | 2052.3 | 84.4 |
| Y1:Actin | Actin | Secondary | 2 | 2 | | 361 | 40 | 16158.6 | 108083.9 | | 6.3 | 8135.4 | 11.5 |
| Y2:Actin | Actin | Secondary | 3 | 1 | | 359 | 40 | 15715.9 | 108249.2 | | 6.5 | 7994.9 | 12.3 |
| Y3:Actin | Actin | Secondary | 4 | 1 | | 358 | 40 | 17698.6 | 123832.4 | | 6.6 | 6660.8 | 14.0 |
| Y4:Actin | Actin | Secondary | 5 | 1 | | 360 | 40 | 13561.6 | 90588.8 | | 6.3 | 6045.0 | 19.0 |
| Y5:Actin | Actin | Secondary | 6 | 1 | | 360 | 40 | 8570.6 | 54992.9 | | 6.0 | 4178.7 | 11.5 |
| O1:Actin | Actin | Secondary | 7 | 1 | | 358 | 40 | 9648.4 | 62823.9 | | 6.1 | 3580.7 | 16.8 |
| O2:Actin | Actin | Secondary | 8 | 1 | | 359 | 40 | 10931.2 | 72720.2 | | 6.2 | 3885.6 | 15.0 |
| O3:Actin | Actin | Secondary | 9 | 1 | | 358 | 40 | 9224.4 | 60209.5 | | 6.1 | 3625.0 | 12.4 |
| O4:Actin | Actin | Secondary | 10 | 1 | | 359 | 40 | 10215.6 | 69400.7 | | 6.4 | 4882.4 | 22.1 |
| O5:Actin | Actin | Secondary | 11 | 1 | | 359 | 40 | 10853.9 | 69026.3 | | 6.0 | 3271.8 | 15.6 |
| Y1:Stat1 | Psmb9 | Secondary | 12 | 1 | | 281 | 23 | 795.9 | 8908.8 | | 10.5 | 220.1 | 17.7 |
| Y2:Stat1 | Psmb9 | Secondary | 13 | 1 | | 280 | 23 | 1783.9 | 19243.3 | | 10.1 | 569.5 | 21.8 |
| Y3:Stat1 | Psmb9 | Secondary | 14 | 1 | | 282 | 23 | 1372.2 | 15003.2 | | 10.3 | 238.6 | 19.3 |
| Y4:Stat1 | Psmb9 | Secondary | 15 | 1 | | 280 | 22 | 1297.1 | 12351.8 | | 8.9 | 416.5 | 13.8 |
| Y5:Stat1 | Psmb9 | Secondary | 16 | 1 | | 279 | 22 | 851.5 | 8062.7 | | 8.9 | 238.6 | 14.6 |
| O1:Stat1 | Psmb9 | Secondary | 17 | 1 | | 282 | 22 | 2576.9 | 27878.2 | | 10.2 | 879.2 | 18.4 |
| O2:Stat1 | Psmb9 | Secondary | 18 | 1 | | 283 | 23 | 4008.0 | 40697.4 | | 9.5 | 1286.2 | 19.1 |
| O3:Stat1 | Psmb9 | Secondary | 19 | 1 | | 283 | 22 | 3740.6 | 41382.6 | | 10.4 | 1709.9 | 17.5 |
| O4:Stat1 | Psmb9 | Secondary | 20 | 1 | | 283 | 22 | 2661.9 | 27968.6 | | 9.9 | 1061.0 | 18.4 |
| O5:Stat1 | Psmb9 | Secondary | 21 | 1 | | 284 | 23 | 3535.5 | 34217.6 | | 9.1 | 1218.9 | 18.3 |

File: Psmb9_Jess

## Standard Plots

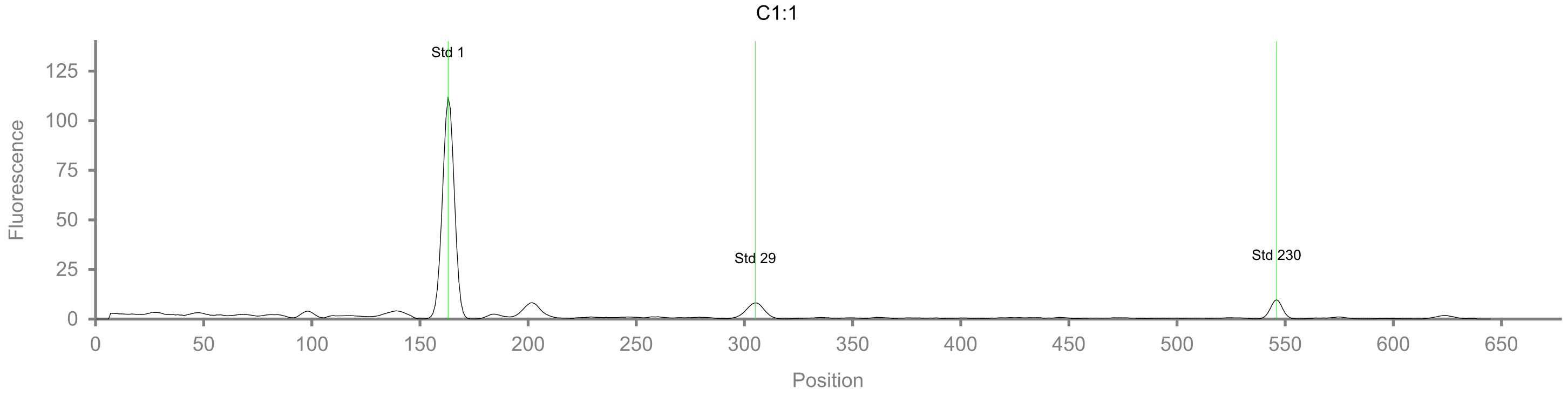


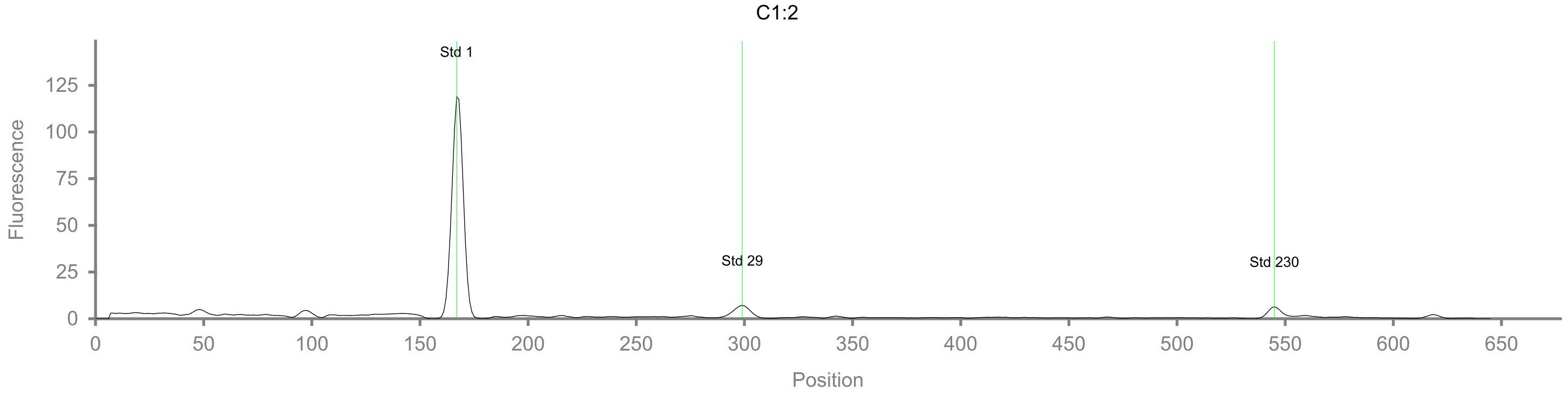


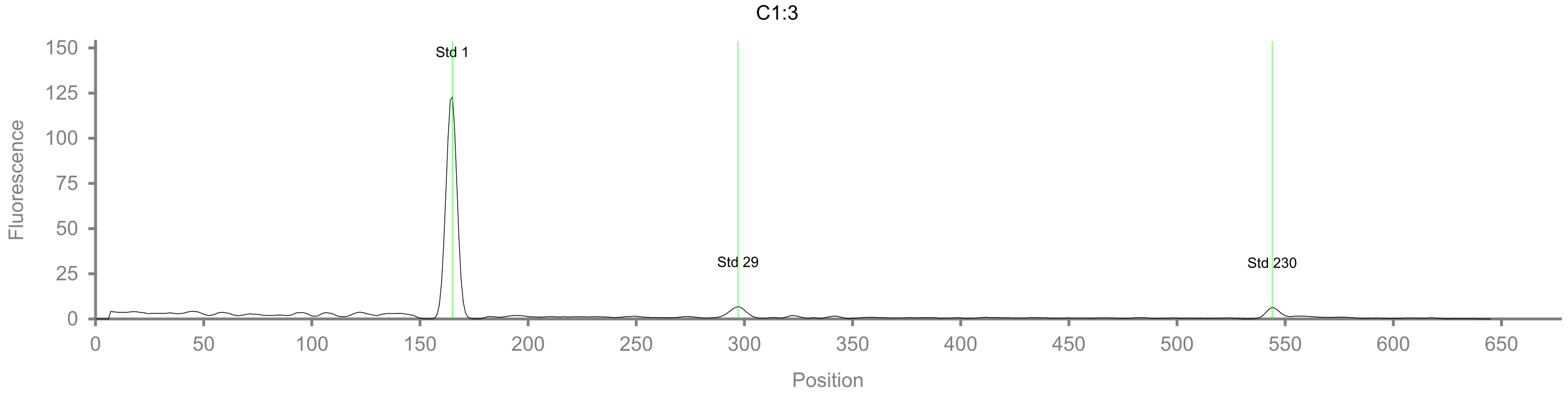


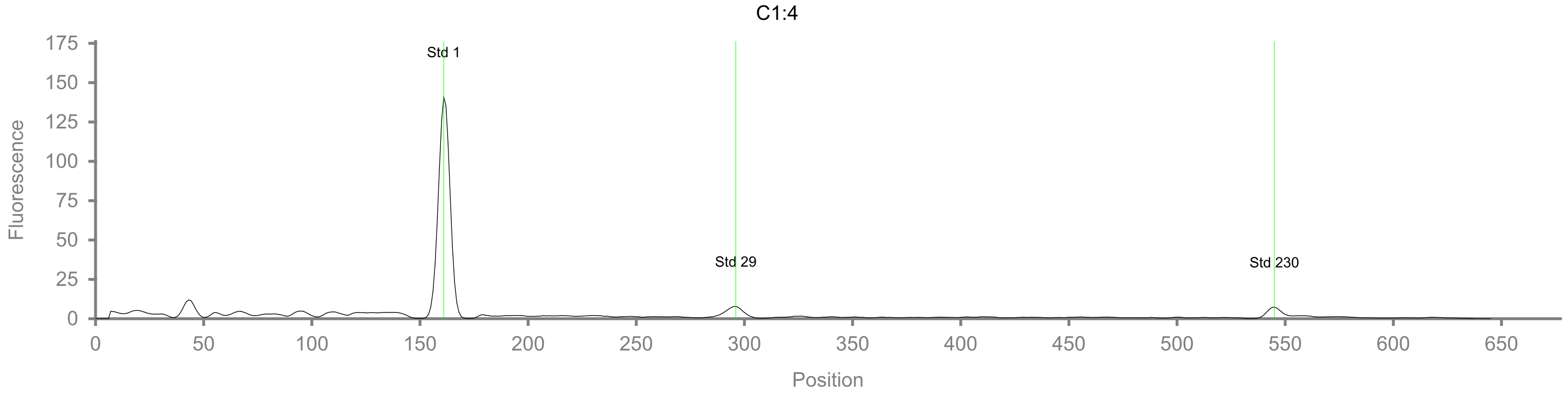

# File: Psmb9_Jess

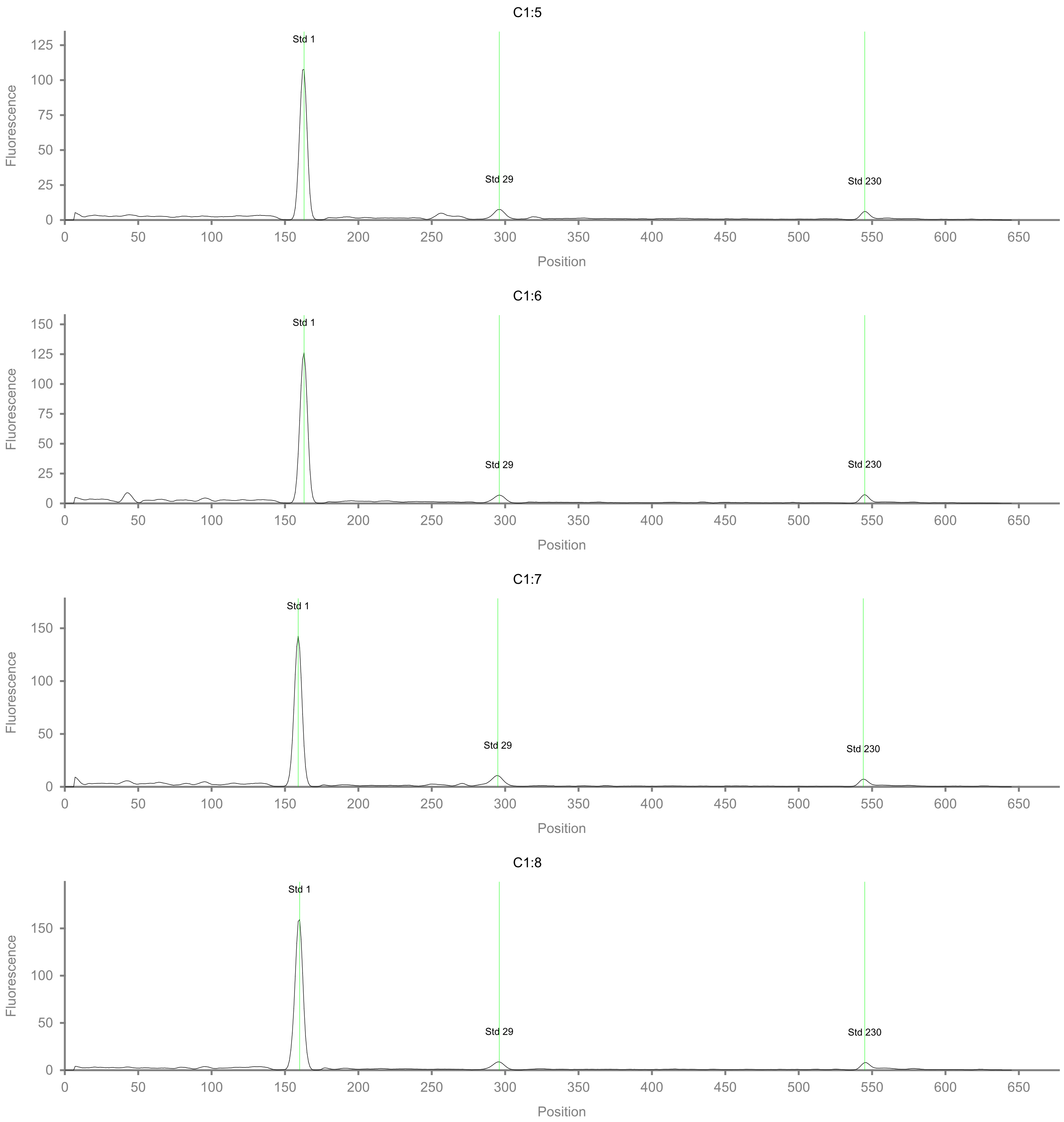

# File: Psmb9_Jess

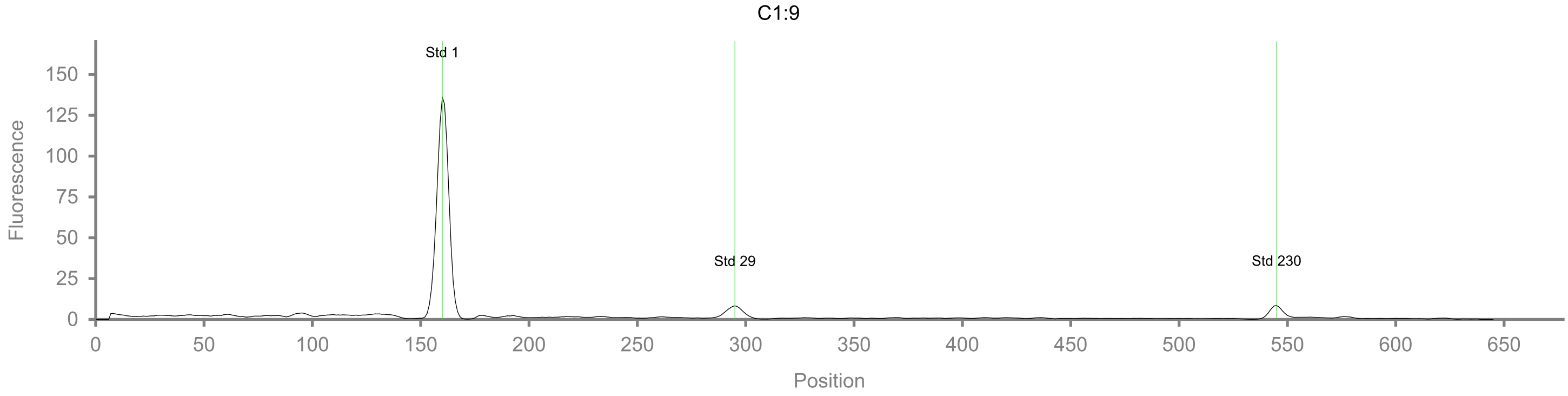


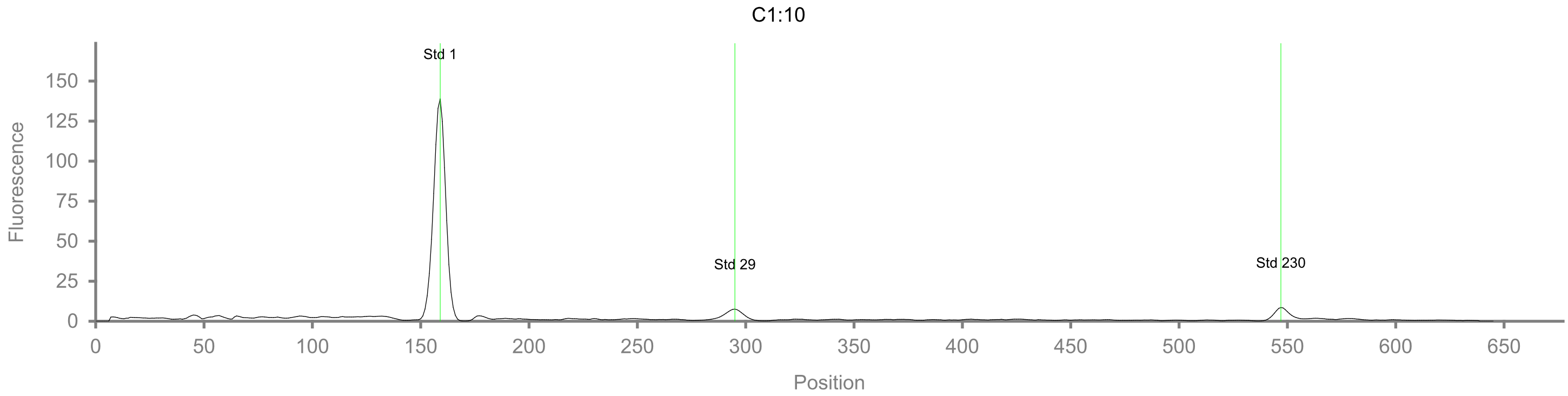


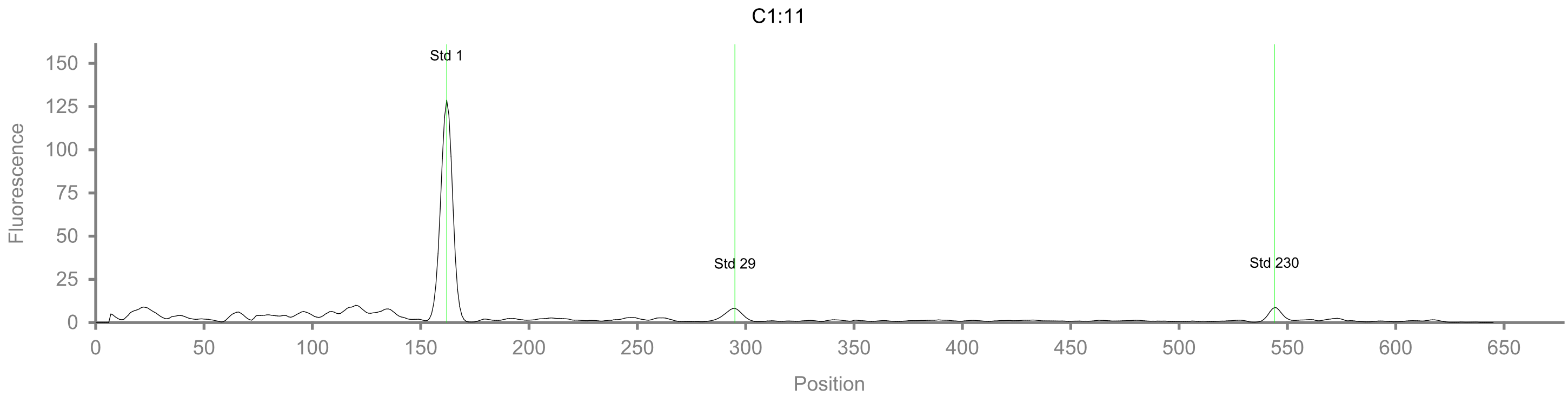


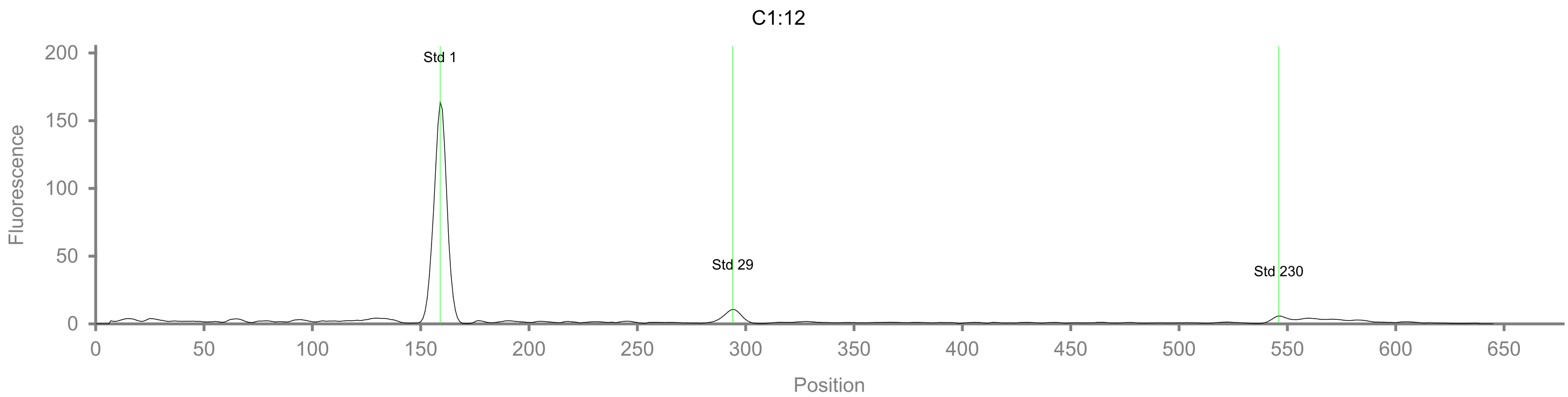




proteinsimple
a biotechne brand

# File: Psmb9_Jess

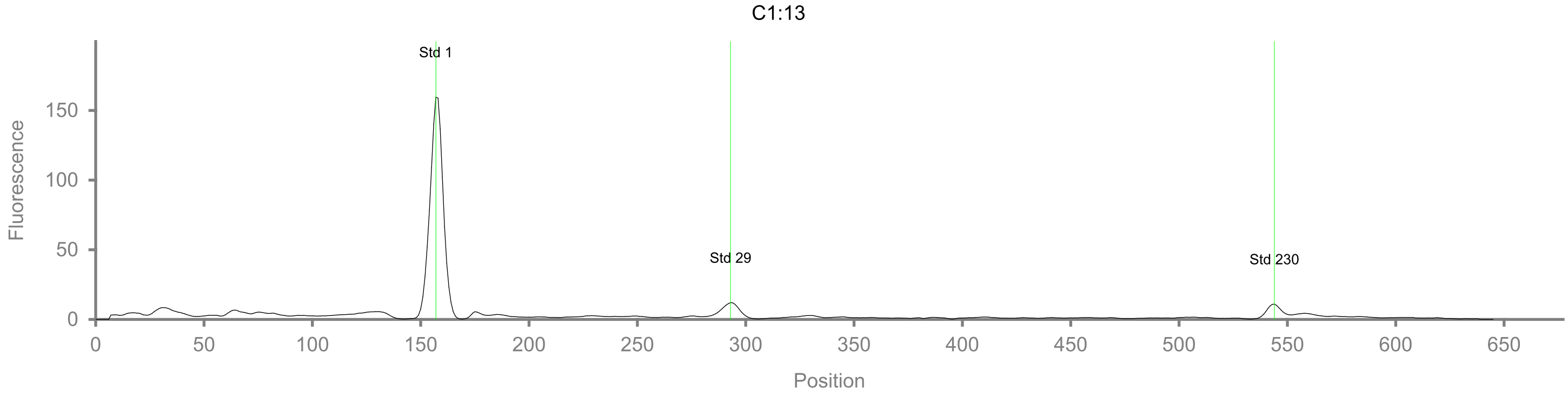


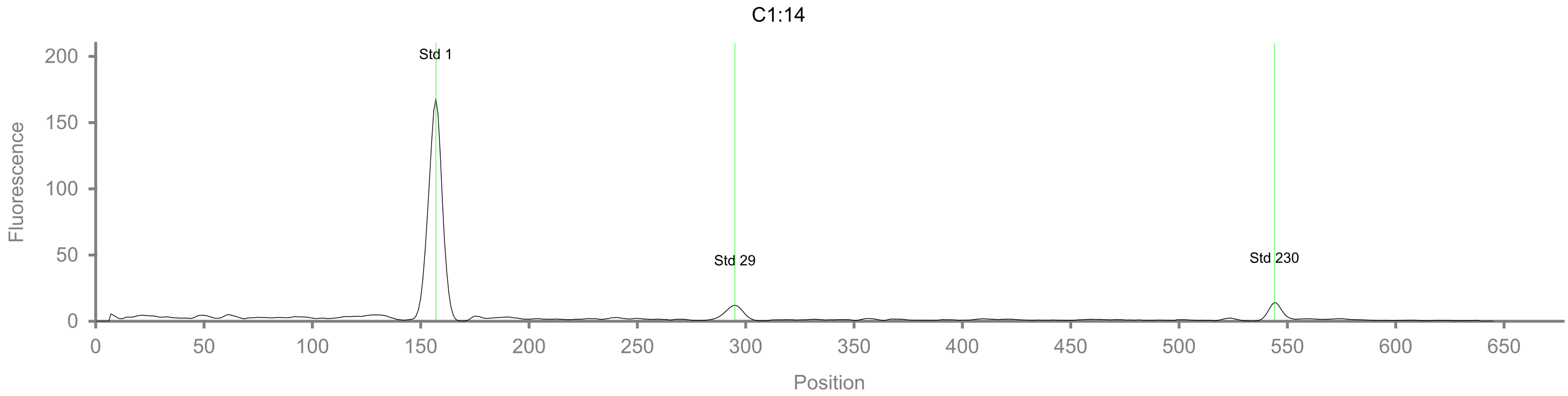


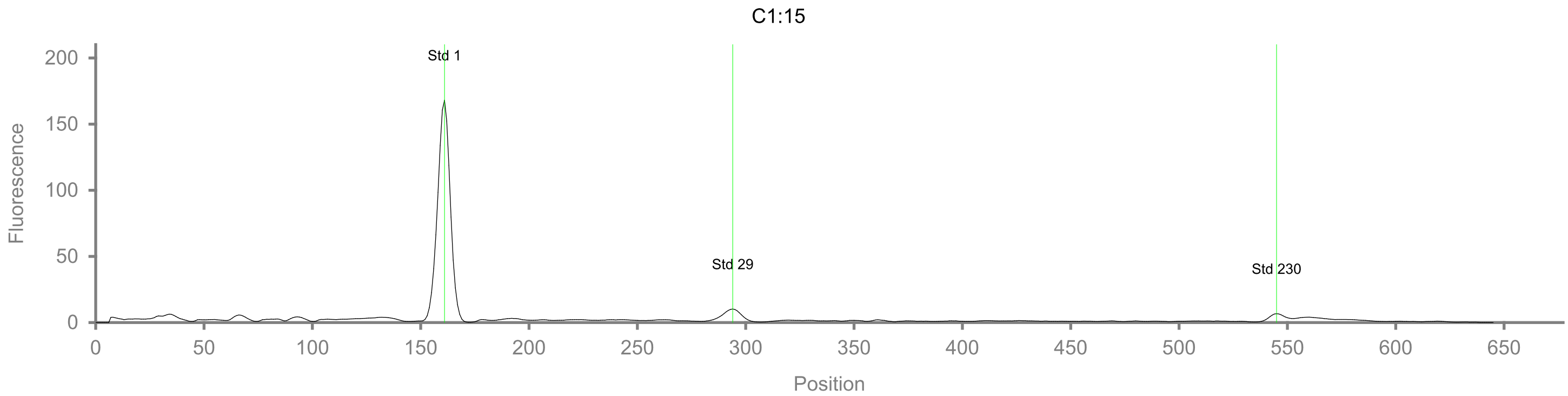


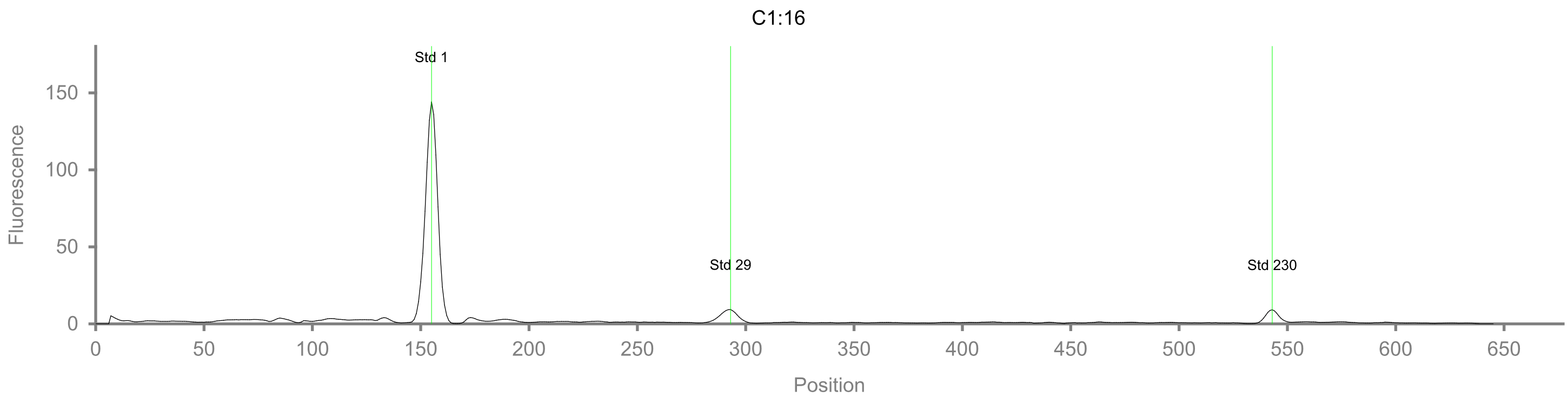


C:\Users\\OneDrive\Desktop\Psmb9_Jess.cbz

# File: Psmb9_Jess

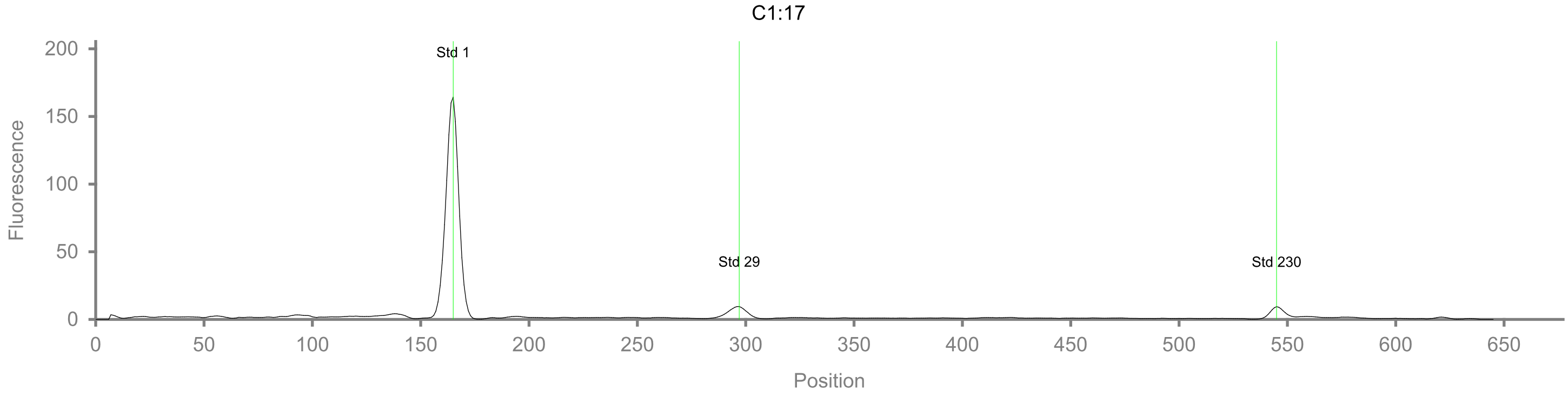


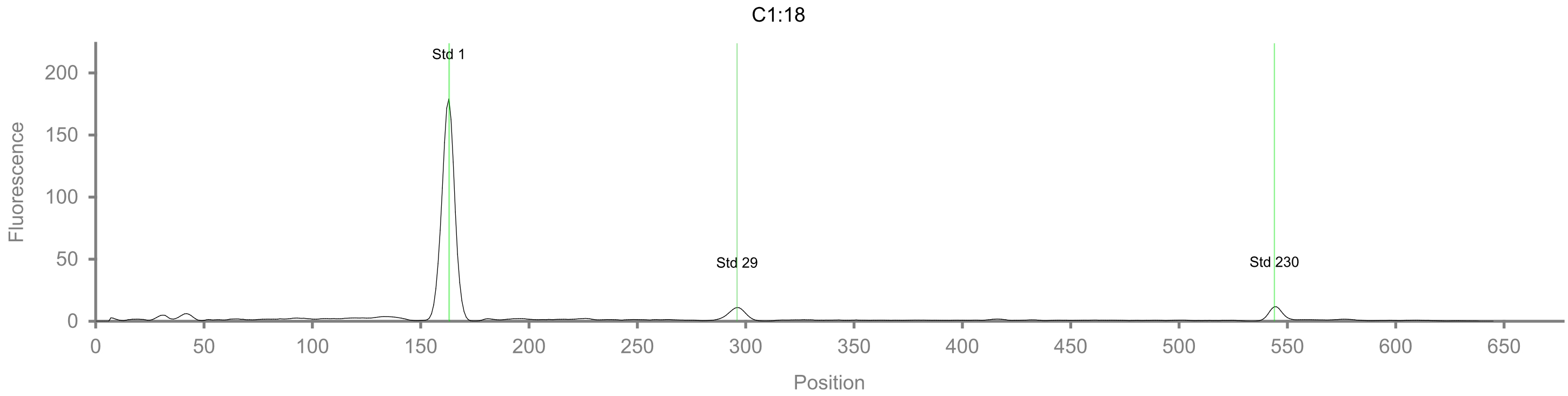


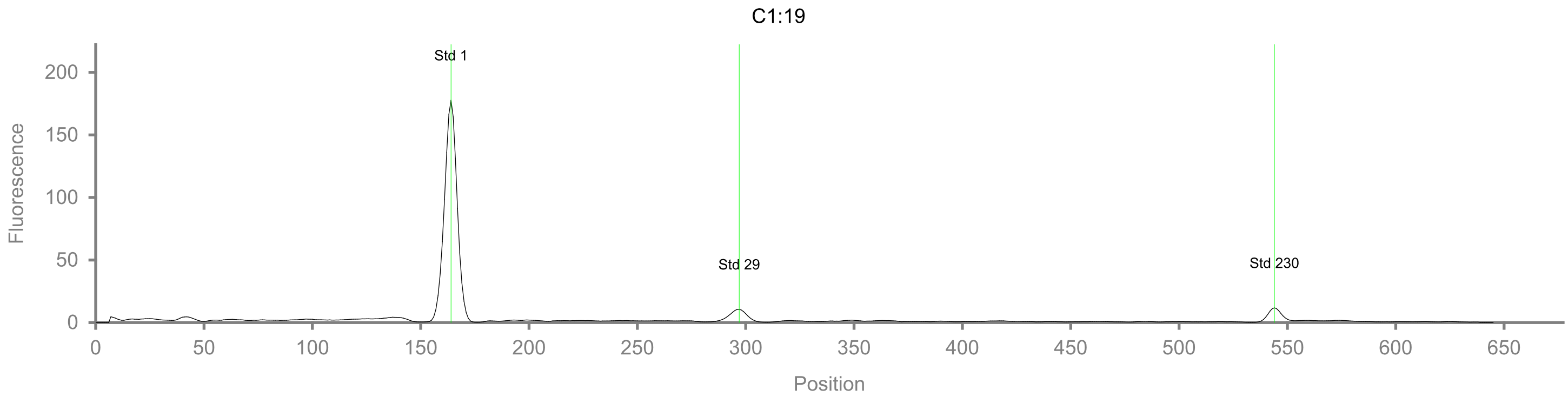


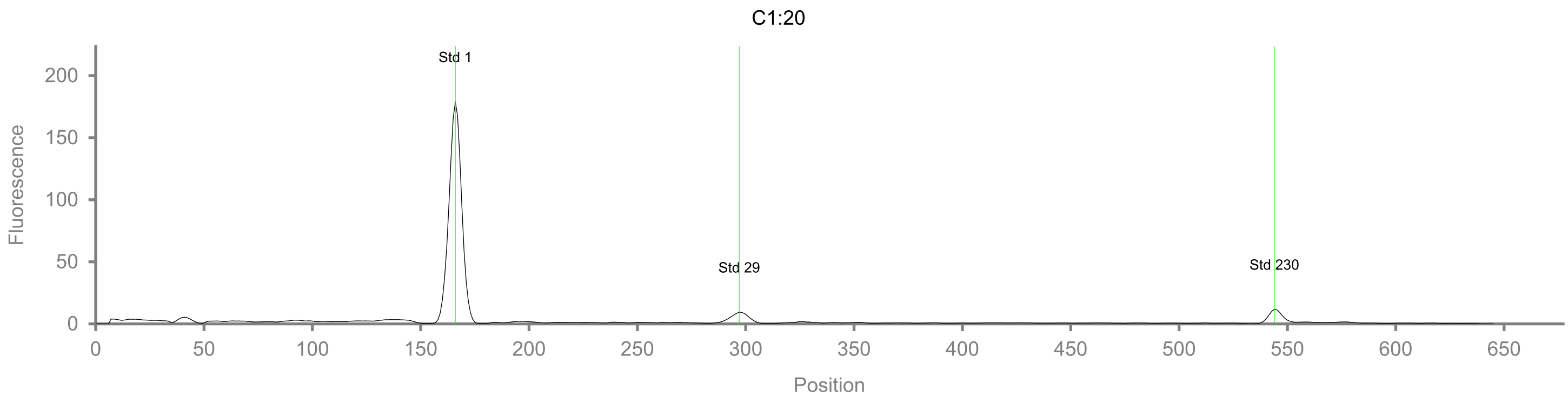

# File: Psmb9_Jess

C1:21

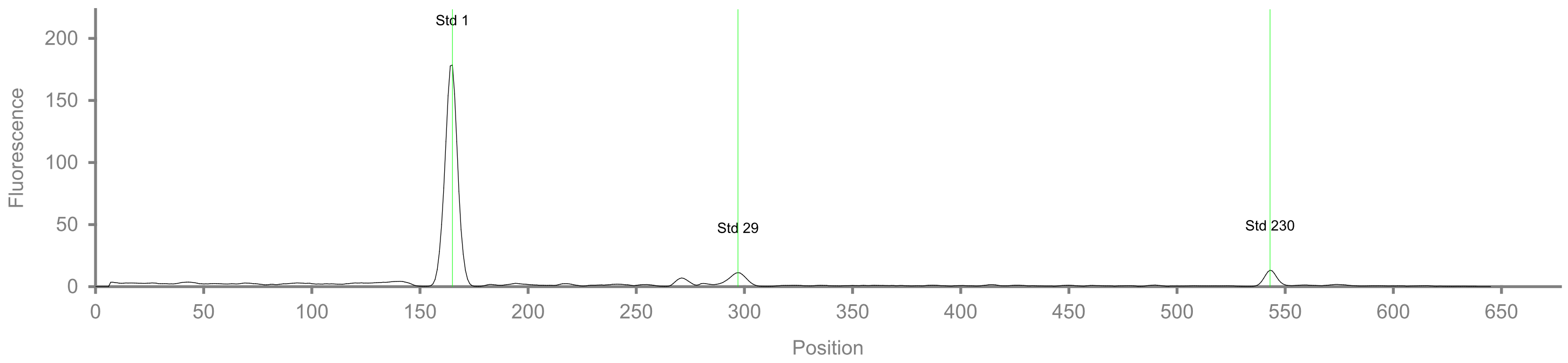


C1:22

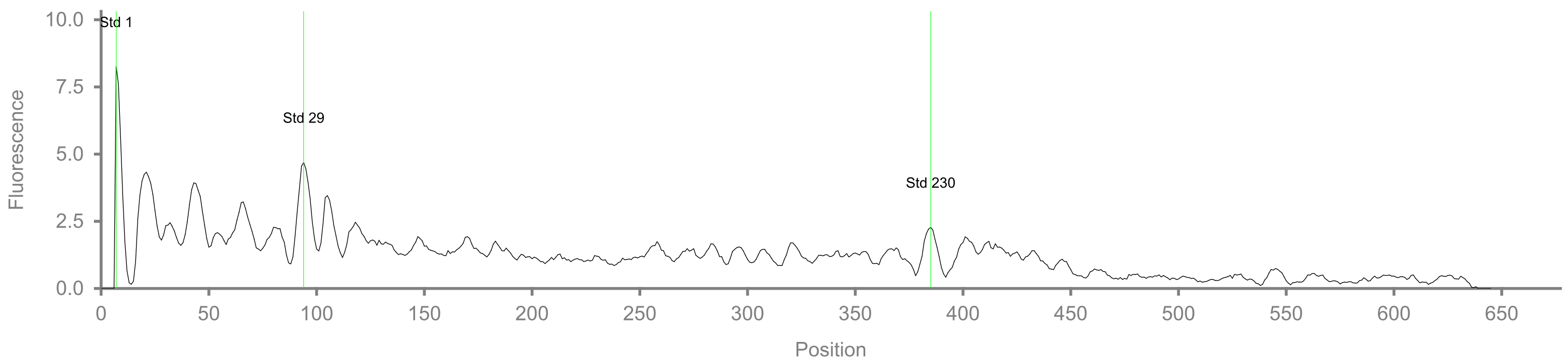


C1:23

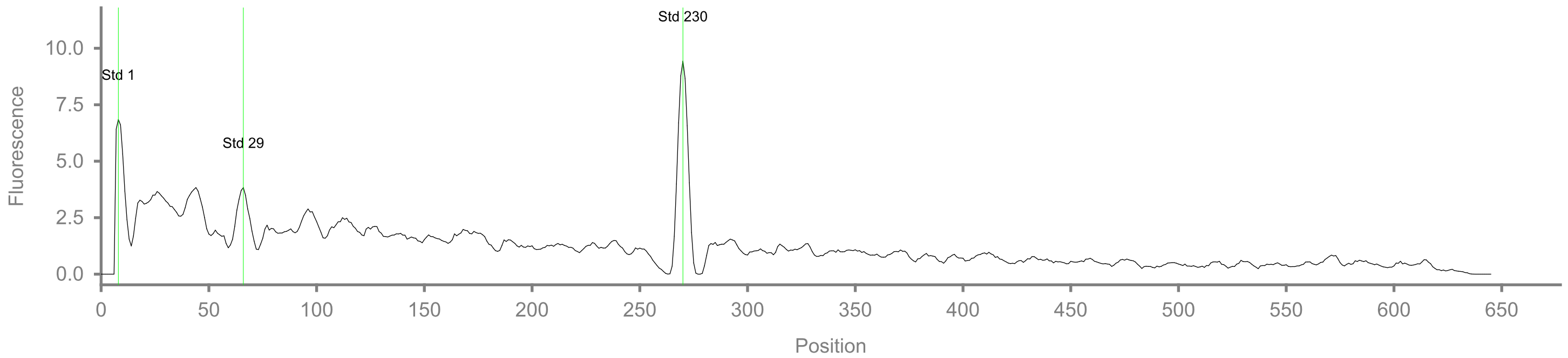


C1:24

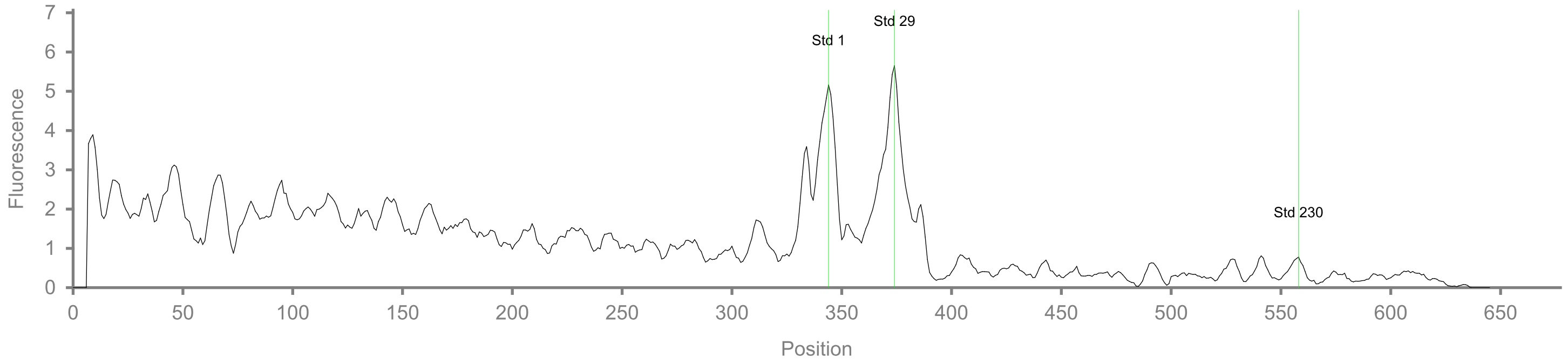


C:\Users\\OneDrive\Desktop\Psmb9_Jess.cbz

proteinsimple a biotechne brand

# File: Psmb9_Jess

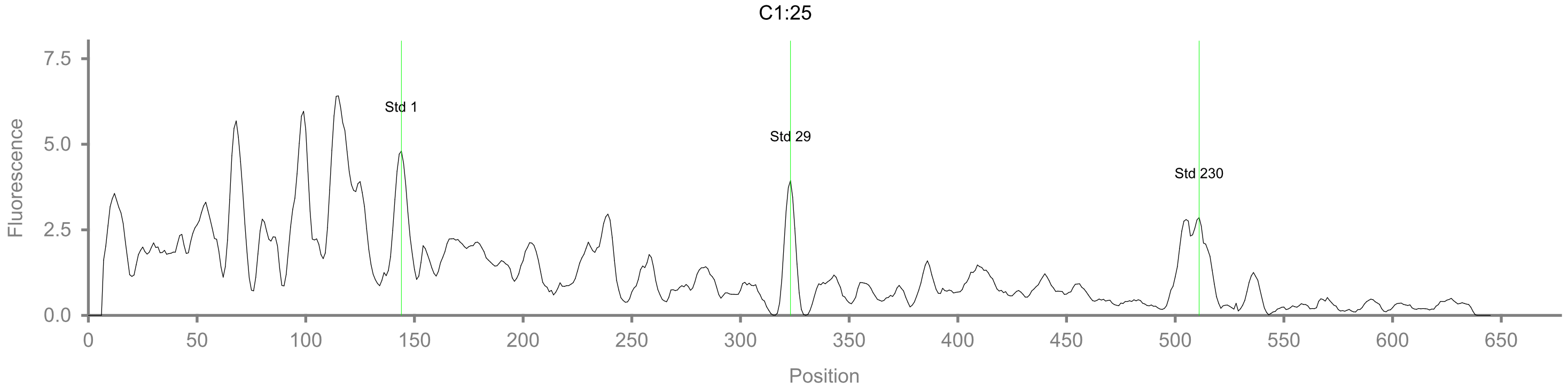

File: Psmb9_Jess

## Standard Peaks (pixels)

| | | | | Std 1 | Std 29 | Std 230 |
|---|---|---|---|---|---|---|
| Biot. Ladder | Antibody Diluent | Streptavidin HRP | C1:1 | 163.0 | 305.0 | 546.0 |
| Y1:Actin | Actin | Secondary | C1:2 | 167.2 | 298.9 | 545.1 |
| Y2:Actin | Actin | Secondary | C1:3 | 164.8 | 297.0 | 544.2 |
| Y3:Actin | Actin | Secondary | C1:4 | 161.1 | 295.9 | 545.2 |
| Y4:Actin | Actin | Secondary | C1:5 | 162.7 | 296.0 | 545.1 |
| Y5:Actin | Actin | Secondary | C1:6 | 162.8 | 295.9 | 545.1 |
| O1:Actin | Actin | Secondary | C1:7 | 158.8 | 294.8 | 544.2 |
| O2:Actin | Actin | Secondary | C1:8 | 159.5 | 295.9 | 545.2 |
| O3:Actin | Actin | Secondary | C1:9 | 159.8 | 294.9 | 545.1 |
| O4:Actin | Actin | Secondary | C1:10 | 158.5 | 294.9 | 547.2 |
| O5:Actin | Actin | Secondary | C1:11 | 161.6 | 294.9 | 544.1 |
| Y1:Stat1 | Psmb9 | Secondary | C1:12 | 158.6 | 293.9 | 546.3 |
| Y2:Stat1 | Psmb9 | Secondary | C1:13 | 156.8 | 292.9 | 544.2 |
| Y3:Stat1 | Psmb9 | Secondary | C1:14 | 156.4 | 294.9 | 544.2 |
| Y4:Stat1 | Psmb9 | Secondary | C1:15 | 160.5 | 293.9 | 545.3 |
| Y5:Stat1 | Psmb9 | Secondary | C1:16 | 154.7 | 292.9 | 543.1 |
| O1:Stat1 | Psmb9 | Secondary | C1:17 | 164.5 | 296.9 | 545.2 |
| O2:Stat1 | Psmb9 | Secondary | C1:18 | 162.6 | 295.9 | 544.2 |
| O3:Stat1 | Psmb9 | Secondary | C1:19 | 163.7 | 296.9 | 544.2 |
| O4:Stat1 | Psmb9 | Secondary | C1:20 | 165.9 | 296.9 | 544.2 |
| O5:Stat1 | Psmb9 | Secondary | C1:21 | 164.7 | 296.8 | 543.2 |
| Sample | Primary | Secondary | C1:22 | 6.8 | 94.0 | 385.0 |
| Sample | Primary | Secondary | C1:23 | 7.7 | 65.9 | 270.0 |
| Sample | Primary | Secondary | C1:24 | 343.9 | 374.0 | 558.0 |
| Sample | Primary | Secondary | C1:25 | 144.0 | 323.0 | 510.9 |

File: Psmb9_Jess

## Sample Images

CHEMI, 1s, Sample

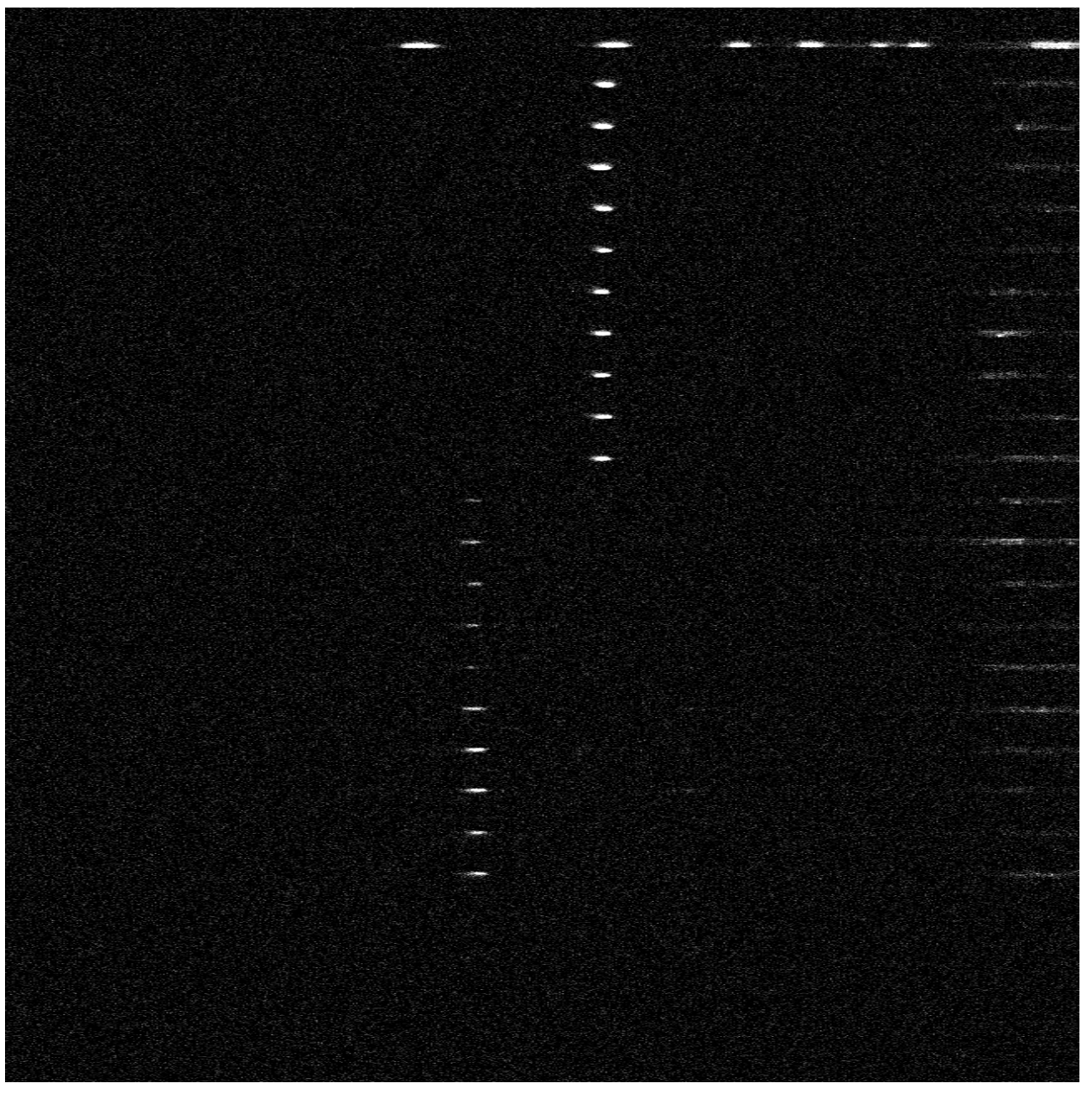

File: Psmb9_Jess

## Standard Image

Std, 4s

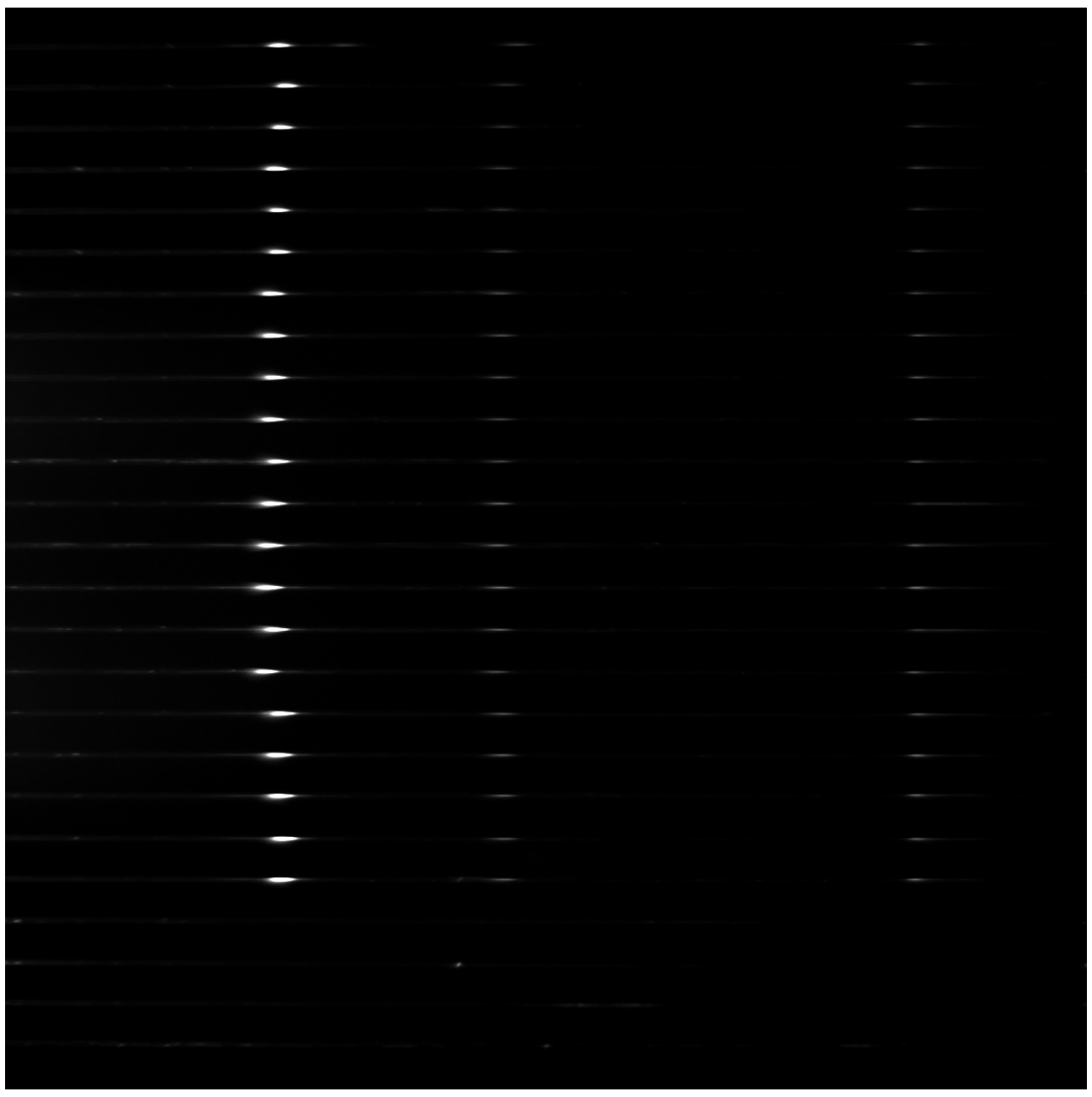

File: Psmb9_Jess

# Analysis Log

| | | | |
|---|---|---|---|
| 2026-07-14 23:49:19 | | Started run: 2026-07-14_23-53-50_Jess  Assay: Jess.assay from Compass for SW v6.3.0-0719 | |
| 2026-07-15 09:36:18 | | Saved analysis and template changes from Compass for SW v6.3.0-0719 | Stat1 |
| | | Added Peak Fit Apply Override "apply fit to Biot. Ladder" | |
| | | Changed well annotation at row A column 2: Name from Sample to Y1:Actin | |
| | | Changed well annotation at row A column 3: Name from Sample to Y2:Actin | |
| | | Changed well annotation at row A column 4: Name from Sample to Y3:Actin | |
| | | Changed well annotation at row A column 5: Name from Sample to Y4:Actin | |
| | | Changed well annotation at row A column 6: Name from Sample to Y5:Actin | |
| | | Changed well annotation at row A column 7: Name from Sample to O1:Actin | |
| | | Changed well annotation at row A column 8: Name from Sample to O2:Actin | |
| | | Changed well annotation at row A column 9: Name from Sample to O3:Actin | |
| | | Changed well annotation at row A column 10: Name from Sample to O4:Actin | |
| | | Changed well annotation at row A column 11: Name from Sample to O5:Actin | |
| | | Changed well annotation at row A column 12: Name from Sample to Y1:Stat1 | |
| | | Changed well annotation at row A column 13: Name from Sample to Y2:Stat1 | |
| | | Changed well annotation at row A column 14: Name from Sample to Y3:Stat1 | |
| | | Changed well annotation at row A column 15: Name from Sample to Y4:Stat1 | |
| | | Changed well annotation at row A column 16: Name from Sample to Y5:Stat1 | |
| | | Changed well annotation at row A column 17: Name from Sample to O1:Stat1 | |
| | | Changed well annotation at row A column 18: Name from Sample to O2:Stat1 | |
| | | Changed well annotation at row A column 19: Name from Sample to O3:Stat1 | |
| | | Changed well annotation at row A column 20: Name from Sample to O4:Stat1 | |
| | | Changed well annotation at row A column 21: Name from Sample to O5:Stat1 | |

File: Psmb9_Jess

| | | | |
|---|---|---|---|
| | | Changed well annotation at row C column 2: Name from Primary to Actin | |
| | | Changed well annotation at row C column 3: Name from Primary to Actin | |
| | | Changed well annotation at row C column 4: Name from Primary to Actin | |
| | | Changed well annotation at row C column 5: Name from Primary to Actin | |
| | | Changed well annotation at row C column 6: Name from Primary to Actin | |
| | | Changed well annotation at row C column 7: Name from Primary to Actin | |
| | | Changed well annotation at row C column 8: Name from Primary to Actin | |
| | | Changed well annotation at row C column 9: Name from Primary to Actin | |
| | | Changed well annotation at row C column 10: Name from Primary to Actin | |
| | | Changed well annotation at row C column 11: Name from Primary to Actin | |
| | | Changed well annotation at row C column 12: Name from Primary to Stat1 | |
| | | Changed well annotation at row C column 13: Name from Primary to Stat1 | |
| | | Changed well annotation at row C column 14: Name from Primary to Stat1 | |
| | | Changed well annotation at row C column 15: Name from Primary to Stat1 | |
| | | Changed well annotation at row C column 16: Name from Primary to Stat1 | |
| | | Changed well annotation at row C column 17: Name from Primary to Stat1 | |
| | | Changed well annotation at row C column 18: Name from Primary to Stat1 | |
| | | Changed well annotation at row C column 19: Name from Primary to Stat1 | |
| | | Changed well annotation at row C column 20: Name from Primary to Stat1 | |
| | | Changed well annotation at row C column 21: Name from Primary to Stat1 | |
| 2026-07-15 09:39:02 | | Saved template changes from Compass for SW v6.3.0-0719 | |
| | | Changed well annotation at row C column 12: Name from Stat1 to Psmb9 | |
| | | Changed well annotation at row C column 13: Name from Stat1 to Psmb9 | |
| | | Changed well annotation at row C column 14: Name from Stat1 to Psmb9 | |
| | | Changed well annotation at row C column 15: Name from Stat1 to Psmb9 | |

File: Psmb9_Jess

| | | | |
|---|---|---|---|
| | | Changed well annotation at row C column 16: Name from Stat1 to Psmb9 | |
| | | Changed well annotation at row C column 17: Name from Stat1 to Psmb9 | |
| | | Changed well annotation at row C column 18: Name from Stat1 to Psmb9 | |
| | | Changed well annotation at row C column 19: Name from Stat1 to Psmb9 | |
| | | Changed well annotation at row C column 20: Name from Stat1 to Psmb9 | |
| | | Changed well annotation at row C column 21: Name from Stat1 to Psmb9 | |
| 2026-07-15 09:50:10 | | Saved analysis changes from Compass for SW v6.3.0-0719 | |
| | | Hide peak at position 248 in manual settings for capillary 2 CHEMI | |
| | | Hide peak at position 406 in manual settings for capillary 2 CHEMI | |
| | | Hide peak at position 432 in manual settings for capillary 2 CHEMI | |
| | | Hide peak at position 484 in manual settings for capillary 2 CHEMI | |
| | | Hide peak at position 511 in manual settings for capillary 2 CHEMI | |
| | | Hide peak at position 548 in manual settings for capillary 2 CHEMI | |
| | | Hide peak at position 538 in manual settings for capillary 6 CHEMI | |
| | | Hide peak at position 555 in manual settings for capillary 6 CHEMI | |
| | | Hide peak at position 549 in manual settings for capillary 10 CHEMI | |
| | | Hide peak at position 409 in manual settings for capillary 12 CHEMI | |
| | | Hide peak at position 409 in manual settings for capillary 13 CHEMI | |
| | | Hide peak at position 410 in manual settings for capillary 14 CHEMI | |
| | | Hide peak at position 408 in manual settings for capillary 15 CHEMI | |
| | | Hide peak at position 410 in manual settings for capillary 16 CHEMI | |
| | | Hide peak at position 411 in manual settings for capillary 17 CHEMI | |
| | | Hide peak at position 411 in manual settings for capillary 18 CHEMI | |
| | | Hide peak at position 524 in manual settings for capillary 18 CHEMI | |
| | | Hide peak at position 382 in manual settings for capillary 19 CHEMI | |
| | | Hide peak at position 411 in manual settings for capillary 19 CHEMI | |
| | | Hide peak at position 447 in manual settings for capillary 19 CHEMI | |
| | | Hide peak at position 351 in manual settings for capillary 19 CHEMI | |
| | | Hide peak at position 525 in manual settings for capillary 19 CHEMI | |
| | | Hide peak at position 522 in manual settings for capillary 20 CHEMI | |
| | | Hide peak at position 410 in manual settings for capillary 20 CHEMI | |
| | | Hide peak at position 411 in manual settings for capillary 21 CHEMI | |
| | | Hide peak at position 525 in manual settings for capillary 21 CHEMI | |
| | | Hide peak at position 373 in manual settings for capillary 24 CHEMI | |
