# Supplementary material for: Multi‐Omics Reveals STAT1 as a Central Regulator of Oxidative Stress‐Induced Senescence in the Aging Retina
Source: Aging Cell. 2026 Nov 7;25(11):e70792. doi: 10.1111/acel.70792 (PMC13744170; doi:10.1111/acel.70792)
Supplement: Supplementary file 2 [file ACEL-25-e70792-s001.docx]

Supplementary Materials for **Multi-omics Reveals STAT1 as a Central Regulator of Oxidative Stress-induced Senescence in the aging Retina**

Yiran Fan^1,2^, Ying Li^1,2^, Xueying Wu^1,2^, Lin Cai^2^, Ruotong Yang^2^, Zhenhui Liu^1,2^, Yan Li^1,2^, Bingtao Hao^2,3^*, Bo Lei ^1,2^*, Xiuxiu Jin^1,2^*

^1^ **Henan Eye Hospital, Henan Provincial People's Hospital, Henan University People's Hospital, Zhengzhou, Henan, China.**

^2^ Eye Institute, Henan Academy of Innovations in Medical Science, Zhengzhou, Henan, China.

^3^ Department of Immunology, School of Basic Medical Sciences, Zhengzhou University, Zhengzhou, Henan, China.

***Correspondence should be addressed to:**

Xiuxiu Jin, Ph.D., E-mail: [jinxiuxiu@zzu.edu.cn](mailto:jinxiuxiu@zzu.edu.cn); Bo Lei, MD, Ph.D., E-mail: [bolei99@126.com](mailto:bolei99@126.com); Bingtao Hao, Ph.D., E-mail: [haobt123@163.com](mailto:haobt123@163.com)

**This PDF file includes:**

Supporting text

Figures S1 to S12

Tables S1-S7

Raw data

**Supporting Information Text**

**Figures**

**Figure S1 Proteomic analysis of lifespan-related genes in aging mouse retina**

**(A)** Heatmap of Z-score-normalized expression for differentially regulated lifespan-related proteins in young and aged mouse retinas. Rows represent proteins, columns represent samples, and hierarchical clustering of samples is indicated at the top. Proteins significantly altered with aging (*P* < 0.05) are indicated, and statistical significance is summarized by -log_10_(*P*-values). Lifespan-associated annotations were derived from the GenAge database, including proteins annotated as lifespan-decreasing (e.g., Arntl, Eef1e1, Hnrnpd, Rae1, Bub3, Clock, and Efemp1) and lifespan-increasing (e.g., Gsta4, Mif, Coq7, Ctf1, Akt1, Akt2, Gpx4, Surf1, and Igf1r). **(B)** Venn diagram illustrating the overlap between the total proteome (7,155 proteins) and the GenAge protein set (136 proteins), identifying 67 overlapping proteins. **(C)** Differentially expressed proteins (DEPs) among the 67 overlapping GenAge-associated proteins in aging retinas. Thirty-one proteins exhibited significant age-dependent changes, including 11 down-regulated and 20 up-regulated proteins.

**Figure S2. Workflow of screening and candidate transcription factor ranking analysis.**

Differentially expressed proteins (DEPs) identified from proteomic analysis of young (4 M) and aged (24 M) mouse retinas were annotated using AnimalTFDB and HOCOMOCO to identify candidate transcription factors. Target-gene sets for candidate transcription factors were obtained from TFlink and the Cistrome Data Browser. Candidate target genes were intersected with ageing-associated differentially expressed genes (DEGs) identified from transcriptomic analysis. Regulatory coverage was calculated as the proportion of overlapping genes between transcription factor target-gene sets and ageing-associated DEGs. Based on this workflow, STAT1 was identified as the top-ranked candidate transcription factor associated with retinal ageing.

**Figure S3 Genome browser visualization of Stat1 occupancy at representative aging-associated genes.**

Genome browser tracks showing enrichment of Stat1 binding at transcription start sites (TSS) of representative aging-associated genes (Psmb9, Tap2, Apoa2, and Ccnd3), based on publicly available Stat1 ChIP-seq datasets obtained from the Cistrome database.

**Figure S4 Quantification of Stat1, p-Stat1, and Psmb9 protein expression in young and aged mouse retinas.**

Retinal tissues from 4-month-old mice (young group, Y-1 to Y-5) and 24-month-old mice (aged group, O-1 to O-5) were analyzed by capillary-based Simple Western assay. Protein abundance was quantified based on integrated chemiluminescence peak areas generated by Compass for Simple Western software and normalized to the corresponding β-actin signal. Data are presented as mean ± SEM (n = 5 biological replicates per group). Statistical significance was determined using a two-tailed unpaired Student’s t-test. (*ns, not significant; **P < 0.01; ***P < 0.001)

**Figure S5 Quantitative analysis of protein expression in different senescence models.**

**(A)** Quantification of protein levels of P-JAK1, JAK1, STAT1, P-STAT1, P53, P21, and P16 in control (NC) and doxorubicin-induced senescent cells. **(B)** Quantification of protein levels of STAT1, P53, P21, and P-STAT1 in control (NC) and TBHP-treated cells. Protein expression levels were normalized to GAPDH and are presented as mean ± SEM from independent biological replicates. Statistical significance is indicated (*P < 0.05, **P < 0.01, ***P < 0.001).

**Figure S6** **Distinct GO biological process enrichment patterns in STAT1 overexpression and oxidative stress–induced senescence models**

Gene Ontology (GO) biological process enrichment analysis was performed using differentially expressed genes (DEGs) identified in STAT1-overexpressing (OE) ARPE-19 cells and TBHP-induced senescent cells. Significantly enriched GO terms are ranked by −log_10_(*P-*value). GO terms enriched in the STAT1 overexpression group (red) were predominantly associated with interferon signaling, defense response to virus, and interferon-gamma–mediated signaling pathway, whereas GO terms enriched in the TBHP-treated group (blue) were mainly related to extracellular matrix organization, cell adhesion, and extracellular structural components, reflecting distinct regulatory programs underlying transcription factor–driven versus oxidative stress–induced senescence.

**Figure S7** **Senescence-associated β-galactosidase (SA-β-gal) staining of ARPE-19 cells under STAT1 overexpression conditions**

Representative SA-β-gal staining images of ARPE-19 cells transfected with pcDNA3.1 or STAT1 overexpression (OE-STAT1). Vector control cells exhibited sparse and weak SA-β-gal staining, whereas OE-STAT1 cells showed markedly increased staining intensity and a higher proportion of SA-β-gal–positive cells, indicating enhanced senescence induction upon STAT1 overexpression. Scale bar: 200 μm. Figure S7 includes replicate SA-beta-gal staining images used for quantification, including the representative image shown in Figure 5F.

**Figure S8 Quantification of intracellular ROS levels in ARPE-19 cells following STAT1 overexpression.**

Intracellular reactive oxygen species (ROS) levels in ARPE-19 cells following STAT1 overexpression, demonstrating increased oxidative stress upon STAT1 activation. Data are presented as mean ± SD, and statistical significance was assessed using one-way ANOVA. Figure S8 includes replicate DCFH/ROS images used for quantification, including the representative image shown in Figure 5H.

**Figure S9 Integrated validation of STAT1-mediated regulation of senescence markers and downstream target genes**

**(A)** Quantification of protein levels of senescence-associated markers, including P53, P21, and ASC, in control (PC DNA3.1) and STAT1 overexpression (OE-STAT1) cells. **(B)** Quantification of protein levels of STAT1 signaling components and downstream targets in control (NC) and doxorubicin-induced senescent cells, including P-STAT1, STAT1, TAP2, ADIPOR1, PDXK, CCND3, and PSMB9. Protein expression levels were normalized to GAPDH and are presented as mean ± SEM from independent biological replicates. Statistical significance is indicated (*P < 0.05, **P < 0.01, ***P < 0.001; ns, not significant). **(C)**RT-qPCR analysis of gene expression changes in the STAT1 overexpression model and TBHP-induced senescent cells. Data are presented as mean ± SEM from three independent biological replicates. Statistical significance was determined using a two-tailed unpaired Student’s t-test (*P < 0.05, **P < 0.01, ***P < 0.001).

**Figure S10 Quantitative analysis of STAT1-regulated target protein expression.**

**(A)** Quantification of protein levels of STAT1 signaling components and downstream targets, including P-STAT1, STAT1, TAP2, ADIPOR1, PDXK, CCND3, and PSMB9, in control (PC DNA3.1) and STAT1 knockdown (si-STAT1) cells.**(B)** Quantification of the same set of proteins in control (PC DNA3.1) and STAT1 overexpression (OE-STAT1) cells. Protein expression levels were normalized to GAPDH and are presented as mean ± SEM from independent biological replicates. Statistical significance is indicated (*P < 0.05, **P < 0.01, ***P < 0.001).

**Figure S11 Validation of STAT1 knockdown and its effects on downstream gene expression**

**(A)** ARPE-19 cells were transfected with three independent siRNA sequences targeting STAT1 or a non-targeting control siRNA. STAT1 knockdown efficiency was confirmed at the mRNA level by RT-qPCR and at the protein level by Western blot. **(B)** The downstream molecular effects of STAT1 depletion were assessed by RT-qPCR analysis of six STAT1-associated target genes. Data are presented as mean ± SEM from three independent experiments. Statistical significance was determined by GraphPad Prism 10.(*P < 0.05, **P < 0.01, ***P < 0.001)

**Figure S12 Marker gene mapping across retinal cell types and intercellular expression correlations of STAT1 downstream targets**

**(A)** Dot plot showing the expression of canonical marker genes used for retinal cell-type annotation. Representative marker genes (e.g., Nrl, Opn1mw) are displayed across major retinal cell populations, including rods, cones, rod bipolar cells (RBC), cone bipolar cells (CBC), amacrine cells (AC), horizontal cells (HC), retinal ganglion cells (RGC), macroglia, microglia, and retinal pigment epithelium (RPE). Dot Color indicates normalized gene expression levels (scaled from −1 to 2), while dot size represents the proportion of cells expressing each gene within the corresponding cell type (0–60%). **(B)** Heatmap illustrating expression correlations between retinal cell types and STAT1 downstream target genes. The y-axis denotes retinal cell types, and the x-axis shows STAT1-associated genes (e.g., Ifit3b, Gsn). Color intensity represents Pearson correlation coefficients (ranging from −1 to 1), with blue indicating negative correlations and red indicating positive correlations. Distinct correlation patterns highlight cell-type–specific activation profiles of STAT1-regulated transcriptional programs across the aging retina.

**Tables:**

Table S1. Quantified transcriptomic profiles and gene expression data.

Table S2. Differentially expressed proteins (DEPs) identified in aging mouse retina.

Table S3. Differentially expressed genes identified across experimental conditions.

Table S4. Candidate transcription factors identified from DEPS using AnimalTFDB and HOCOMOCO annotation。

Table S5. Overlap and integration of STAT1 target genes from TFlink and Cistrome with ageing-associated DEGs.

Table S6. STAT1 target genes identified by ChIP-seq analysis from publicly available datasets.

Table S7. RNA-seq–derived differentially expressed genes (DEGs).

**Raw data:**

Raw data S1. Native Instrument-Generated Jess Simple Western Reports for Stat1, p-Stat1, and Psmb9 in Young and Aged Mouse Retinas.

This file contains the native, non-editable PDF reports generated directly by the Jess Automated Capillary-based Simple Western System (ProteinSimple, Bio-Techne). The reports were exported using Compass for Simple Western software and include the original instrument-generated analytical records, including sample information, electropherograms, virtual blot images, molecular weight determination, and chemiluminescence peak area quantification. Target protein abundance was quantified based on the integrated chemiluminescence peak area and normalized to the corresponding β-actin signal. These reports correspond to the Simple Western experiments presented in Figure 4C and the quantitative analysis shown in Figure S4.
